# Report on botanical nomenclature—Melbourne 2011. XVIII International Botanical Congress, Melbourne: Nomenclature Section, 18–22 July 2011

**DOI:** 10.3897/phytokeys.41.8398

**Published:** 2014-08-29

**Authors:** Christina Flann, Nicholas J. Turland, Anna M. Monro

**Affiliations:** 1 Species 2000, Naturalis Biodiversity Center, Leiden, 2333 CR, The Netherlands; 2 Botanischer Garten und Botanisches Museum Berlin-Dahlem, Freie Universität Berlin, Königin-Luise-Str. 6–8, 14195 Berlin, Germany; 3 Centre for Australian National Biodiversity Research, GPO Box 1600, Canberra ACT 2601, Australia

## Preface

This is the official Report on the deliberations and decisions of the ten sessions of the Nomenclature Section of the XVIII International Botanical Congress held in Melbourne, Australia, in July 2011. The Section took place on five consecutive days prior to the Congress proper. The Section meetings were hosted by the School of Botany at the University of Melbourne, Australia. Technical facilities included full electronic recording of all discussion spoken into the microphones as well as a series of screen shots capturing what was displayed via computers on the overhead screens. Text of all proposals to amend the *Code* was displayed on one screen, while the relevant text of the *Code* itself was displayed on another screen allowing suggested amendments to be updated as appropriate.

There was a strong female presence in leadership positions, despite the ratio of registered members still being skewed toward the male side (approx 33% of the registered members were women). The Section had the honour of being welcomed by the President of the Congress, Judy West, who was also a registered member of the Section and actively contributed to debate on the *Acacia* issue. The Secretary-General of the Congress, Karen Wilson, was very active during the entire Nomenclature Section, which is no surprise as she is secretary of the General Nomenclature Committee as well as acting as the spokesperson for the Special Committee on Electronic Publishing. Sandra (Sandy) Knapp did an exemplary job in the role of President of the Section. Her handling of the procedures, debates and personalities was an impressive feat and was all conducted in a very cheerful and positive atmosphere. She contributed to a complete record by keeping the use of microphones very much under control, admonishing those who attempted to speak without a microphone with a friendly but firm use of humour. Pauline Ladiges and her team from the School of Botany ensured that the entire complicated proceeding ran smoothly and comfortably.

**Figure:**
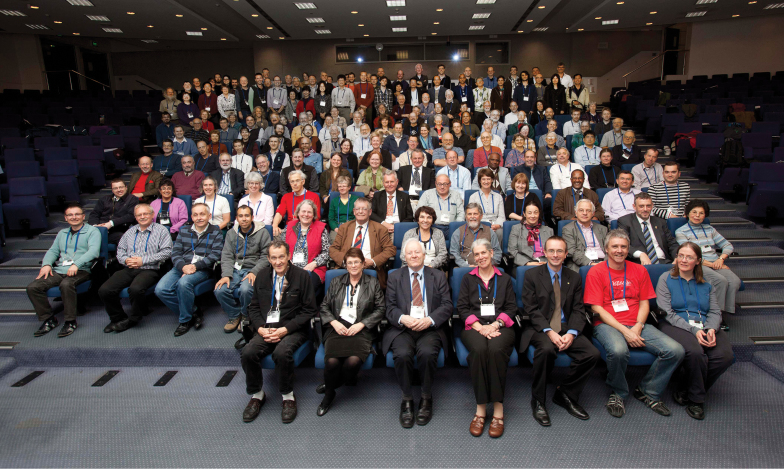
Nomenclature Section of the XVIII International Botanical Congress, Melbourne, Australia, July 2011. — Photograph by Michael Silver / Photonet; reproduced by permission of IBC2011.

A preliminary report of the Section meetings was published soon after the Congress (McNeill & al. in Taxon 60: 1507–1520. 2011). It includes a tabulation of the preliminary mail vote on the published proposals, specifying how the Section acted on each and detailing amendments and new proposals approved upon motions from the floor. It also includes the full report of the Nominating Committee as well as the Congress resolution ratifying the Section’s decisions, neither reproduced here. The main result of the Section’s deliberations is the new *Melbourne Code*, which was published as *Regnum Vegetabile* 154, on 19 December 2012 (McNeill & al. in Regnum Veg. 154. 2012)—a more slender volume than previous editions as it does not include the Appendices (except for App. 1 on the nomenclature of hybrids). It was also published online, on the same date (see http://www.iapt-taxon.org/nomen/main.php). The other Appendices will be published as a separate volume in 2014.

The present full Report of the Melbourne Nomenclature Section conveys, we believe, a true and lively picture of the event, retaining the flavour of goodwill and humour that permeated the Section. It is primarily based on the mp3 electronic recordings. Where necessary, in case of doubt, they were supplemented by the comment slips submitted by almost all of the speakers and scanned into PDF [portable document format] files. Access to the screen shots from the lecture theatre screens was also used to accurately record proposals from the floor that were rejected, as the text of these was often submitted electronically and not read out in full. With these sources combined, and with all motions and voting results double-checked through the soundtrack and published preliminary report of the Section meeting based on two parallel series of notes by the Rapporteur and the Recorder, we are confident that the record published hereunder is accurate and complete.

Before it was cast into its present, final form, this Report went through a succession of phases. Due to advances in technology, this involved fewer phases than the previously published Report of the Nomenclature Section of the St Louis Congress held in 1999 (Greuter & al. in Englera 20. 2000). It is worth noting here that, for various reasons, the Report of the Nomenclature Section of the Vienna Congress held in 2005 are still in progress and will likely be published after the present Report. The Melbourne Section was, as already noted, recorded electronically. This recording was then professionally transcribed by Pacific Transcription, Queensland, Australia and cross-checked and edited by Anna Monro. The edited version of the transcript was then heavily re-edited by Christina Flann, to convert it into a report format. At the same time some portions were rearranged to ensure that the Report reflects the sequence of relevant provisions in the *Code* even when the order of the debates differed. Deviations from the chronology of events are indicated in the text by italicized bracketed notes. The resulting report was then further edited by Nicholas Turland.

As in the case of previous nomenclature reports, which the present one follows in style and general layout, the spoken comments had to be condensed and partly reworded, rarely rather drastically. For this reason, indirect speech has been used consistently. Additions by the authors of this Report are placed between square brackets; they include explanatory or rectifying notes, records of reactions of the audience (to illustrate the sessions’ emotional background) and reports on procedural actions. As in previous reports, the index to speakers has been integrated with the list of registered Section members.

The Section in Melbourne was well attended, particularly given the distances most members had to travel to be present. There were 204 registered members in attendance carrying 396 institutional votes in addition to their personal votes, making a total of 600 possible votes representing 56 countries (detailed by McNeill & al. in Taxon 60: 1509, Table 2. 2011). There were 11 card votes, in which participation was high, the proportion of members voting ranging between 72.5% and 94.2% (the latter for a new proposal dealing with *Acacia*).

As is always to be expected, the geographical composition was uneven with a predictably relatively high representation of Australians (34%) and the familiar trend of strong (67%) representation of Anglophone countries (Australia plus United States, Canada, Britain and New Zealand). Mainland Europe was represented by 30 members (15%). China was represented by 10, Taiwan by 6 and India by 5 members. The low African (4%) and South American (2%) presence is far from ideal. As noted in the Preface of the St Louis Report (Greuter & al. in Englera 20: 7. 2000), these distortions are to some degree unavoidable and are somewhat compensated by a better balanced representation of institutional votes. However, African countries were represented by only 28 institutional votes (7%) and South American countries by 38 (10%). Despite its vast collections, Russia was represented by one member holding 10 institutional votes. For comparison, the Anglophone countries listed earlier had 158 institutional votes (43%) and mainland Europe 120 (30%). These inequalities have deep historical roots and are compounded by uneven access to funding to attend the Section meetings. Re-evaluation of the institutional voting system via the Special Committee on Institutional Votes created in Melbourne will hopefully offer some solutions at the next Nomenclature Section at the XIX International Botanical Congress to be held in Shenzhen in 2017.

It is worth noting that, despite the series of highly charged articles relating to the *Acacia* issue preceding the meeting, all debate on the issue was undertaken in a positive atmosphere, focussing on finding a solution to the dissatisfaction, and the results were graciously accepted by most.

Several significant decisions were made by the Melbourne Section, most visibly the change of title from the *International Code of Botanical Nomenclature* to the *International Code of Nomenclature for algae, fungi, and plants*. In addition to this change and the separation of the Appendices of conserved and rejected names, suppressed works and binding decisions, there were five other major changes to the rules of nomenclature adopted in Melbourne: the acceptance of certain forms of electronic publication as constituting effective publication; the option of using English as an alternative to Latin for the validating descriptions or diagnoses of new taxa of all organisms treated under the *Code*; the requirement for registration as a prerequisite for valid publication of new names of fungi; the abolition of the provision for separate names for fungi with a pleomorphic life history; and the abandonment of the morphotaxon concept in the nomenclature of fossils. It is worth stressing that these fairly momentous decisions were all taken in a very positive, good-humoured atmosphere. Thanks for that are due to Sandra Knapp and the team, who made it all run smoothly. We also thank Pensoft Publishing for agreeing to publish this Report as an issue of *PhytoKeys* and to sponsor its open access. Our thanks also go to the International Association for Plant Taxonomy for contributing the costs of producing this Report.

We would like to dedicate this volume to R. K. (Dick) Brummitt (1937–2013), whose tireless dedication to the world of botanical nomenclature was legendary. He was indispensable in his years based at the Royal Botanical Gardens, Kew, serving as secretary of the Nomenclature Committee for Vascular Plants (the former Committee for Spermatophyta). He trained generations of people in the art of botanical nomenclature and contributed in so many different ways to the advancement of the *Code*. His input, expertise and opinions will be sorely missed.

Christina Flann, Nicholas J. Turland & Anna M. Monro

**Note:** The figures given in parenthesis for each proposal in this Report correspond to the result of the preliminary mail vote (Yes: No: Editorial Committee: Special Committee).

## Eighteenth International Botanical Congress Melbourne 2011

### Nomenclature Section

Bureau of Nomenclature

*President*: Sandra Knapp

*Vice-presidents*: Barbara Briggs, Walter Gams, Dmitry Geltman, Werner Greuter, Gideon Smith

*Rapporteur-général*: John McNeill

*Vice-rapporteur*: Nicholas J. Turland

*Recorder*: Brendan J. Lepschi

## First session

Monday, 18th July 2011, 09:00–12:30

**Knapp** welcomed everyone to the University of Melbourne and opened the first nomenclature session by inviting the President of the XVIII International Botanical Congress to address the Section.

**West** welcomed the Section members to Melbourne and thanked Ladiges and all her helpers for the reception hosted by Botany at Melbourne University the night before. She noted the importance of the Nomenclature Section of the Congress as representing most of the world’s leaders in botanical nomenclature with an intricate knowledge of the *Code* as well as a lot of people who had not been to one of these Sections before. She highlighted the importance of the opportunity for early-career people to learn more about the *Code*, since it plays such an important role for taxonomy and our discipline as a whole. She felt that understanding the importance of the deliberations during the week offered a really good learning experience. She described being bamboozled for a while at the first Nomenclature Section she attended, but described it as a really good opportunity for people to learn from each other. She talked about coming together as a taxonomic community to make decisions that impact taxonomy in a collegial manner and encouraged taking that seriously and voting with thought. She mentioned key decisions to be taken, including *Acacia*, electronic publishing and mycological proposals, and wished a week of good discussion for the delegates present for the Nomenclature Section, which was, she added, one of the biggest.

**Knapp** went through various housekeeping details, thanking West and Ladiges, introducing the local team: Erin, Emma, Stephanie, Karen, Rose, Daniel, David and Trisha, mentioning the new aspects of a Twitter tag: #ibc18, Facebook page: facebook.com/ibc2011, wifi password and outlining the daily schedule: 9:00 a.m. to 5:30 p.m. with lunch and two tea breaks. The Bureau of Nomenclature was presented: President Sandy Knapp, Rapporteur-général John McNeill, Vice-rapporteur Nicholas (Nick) Turland and Recorder Brendan Lepschi assisted by Anna Monro. The appointment of Vice-presidents for the Section was announced: Barbara Briggs (Australia), Walter Gams (Netherlands), Dmitry Geltman (Russia), Werner Greuter (Switzerland) and Gideon Smith (South Africa). She explained that a Nominating Committee was required at each Nomenclature Section to prepare slates of candidates for election to the Permanent Nomenclature Committees, which do the work of nomenclature between International Botanical Congresses. The importance of these Permanent Committees was emphasized, as they consider and make recommendations on proposals to conserve and reject names, and serving on them is a big job. The following Nominating Committee was appointed: Barbara Briggs, Chair (Australia), Patrick Brownsey (New Zealand), Katherine Challis (U.K.), Ali Dönmez (Turkey), Renée Fortunato (Argentina), Hugh Glen (South Africa), Tseng-Chieng Huang (Taiwan), Paul Kirk (U.K.), Bob Magill (U.S.A.), John Wiersema (U.S.A.), Sebsebe Demissew (Ethiopia) and Chin-Sung Chang (China).

**McNeill** noted that the secretaries of the Permanent Nomenclature Committees had already prepared lists of their suggestions for membership of the Permanent Committees for the next six years, and invited any further suggestions to be passed to Barbara Briggs. He added that the role of the Nominating Committee was primarily to see that these suggestions were appropriate, that there was good geographic and disciplinary balance, and that things were being done properly and in order.

**Knapp** introduced the tellers appointed by the Bureau: Bronwyn Collins (Australia), Christina Flann (Netherlands), Lulu Rico Arce (U.K.) and Nadia Talent (Canada), who would count a card vote should any be needed. She reiterated the importance of the number of new participants present for botanical nomenclature as a dynamic area of endeavour. Regrets from some familiar faces from Nomenclature Sections past were noted. In particular Dick Brummitt, a person who had served nomenclature over many, many years in various capacities, unable to attend due to health reasons, was sent best wishes and hopes for a recovery very soon as his presence would be missed. An *in memoriam* booklet of botanists who had passed away since the last Botanical Congress was available at the registration desk.

Knapp went on to discuss practical issues given that it was one of the biggest Nomenclature Sections to date with a record number of proposals. The numbers registered in St Louis were 256, in Vienna around 250 and there were about 200 people registered for this Nomenclature Section. The number of proposals had also gone up—218 in St Louis, 312 in Vienna and 338 in Melbourne. She concluded that this meant more business to get through in the same amount of time and all members were requested to be brief and to the point so that the Section could do its business in the most efficient way possible.

Knapp proceeded with the following announcements:

- The proceedings would be recorded and those wishing to speak were requested to speak slowly and clearly.
- All speakers were required to wait for the microphone and preface their remarks with their name and home base.
- Contributors should be brief, concise, stick to the point and remember the aim was to facilitate botanical nomenclature.
- After speaking everyone was asked to submit a written version of each comment, either on comment forms or via e-mail ibc_recorder2011@csiro.au for those who could no longer actually write with a pen (Knapp gave herself as an example). She added that, as Greuter had said at St Louis, sometimes the difference between what was said and what was written down could be quite amusing.

Knapp finished with a personal plea to act as a community and show the outside world a commitment to have nomenclature serve the advancement of science. She exhorted everyone to come together and make the week something that would be remembered very positively once the IBC began the following week.

**McNeill** outlined the way in which a Nomenclature Section operates. As it was a very large Section with a lot of people who had not previously been at a Nomenclature Section meeting it was deemed worthwhile to take some time to go through a general outline of the process. The main business of the week concerned the consideration of the published proposals to amend the *Code*, a synopsis of which had been prepared before the Congress. The Nomenclature Section functioned as an integral part of the Congress. At one time the Nomenclature Section was comparable to a physiology section or a taxonomy section and met during the Congress. In the last 40 or 50 years the Nomenclature Sections had met separately in the week preceding the Congress. As an international body, the general procedures for meetings throughout the world were followed, but a Section was not governed by any particular national set of rules such as Robert’s Rules of Order. The rules were decided by the Section based on the broad principles that apply to general parliamentary debate and discussion.

All of the discussion dealt with proposals, whether procedural ones or proposals to amend the *Code*. A proposal had to be made and seconded by somebody. Modification of the proposal could be made by moving an amendment. Then discussion was confined to the amendment and the proposal to amend must be seconded. Only if seconded would the Chair allow discussion. Once seconded, discussion must be confined entirely to the amendment and not to the original proposal. If the amendment was defeated, that was the end of the amendment. If it was adopted then the amended proposal became the substantive motion. Then discussion could resume on the proposal and a final decision taken. Normally the majority was a simple majority of 50% but in certain situations a Section had adopted different procedures. There was a preliminary mail vote on all published proposals. This was described in the *Code* as being a guiding mail vote. One of the ways in which previous Sections had taken guidance from the mail vote is to agree that any proposal to amend the *Code* that receives 75% or more “no” votes is not discussed at the Section. It is ruled as defeated, unless somebody proposes that it be taken up and discussed and four other persons supported that request. He proposed that this Section adopt the same procedure, that any proposal to amend the *Code* that received 75% or more “no” votes in the mail vote would not be discussed unless moved by one person and supported by four others.

**Malécot** asked for clarification regarding the value of 75% because for Art. 7 Prop. D on the list 75% was given, but when computed it was 74.55%.

**McNeill** suggested that rounding up was inappropriate although understandable and it would be best to consider it when the proposal came up. [This was **agreed**.] He continued outlining the procedures, noting that previous Sections had adopted a rather similar procedure in a slightly different situation. As the published proposals had to be submitted by November of the previous year, things may have happened in the interval and new proposals could potentially justifiably be proposed during the meeting. Previous Sections had required that this also be supported by four other persons. This would also apply to any substantive amendment. It would not apply to ordinary amendments to proposals that had already been published, but if somebody wanted to make a dramatic change in a proposal, that would be another matter and would fall under the same rule.

Decisions would be voted on, normally by a show of hands, if the result was clear then that was resolved. If the vote was not clear from a show of hands, then people would be asked to show the cards that they had, to indicate institutional votes as well as individual votes. If a show of cards was not clear or if any member of the Section wished to have a formal vote, endorsed by the rest of the Section, then a card vote could be called for. All members of the Section were issued with a sheet with a number of numbered tags. In a card vote, one of these numbers would be designated for that particular vote. Cards that were issued to individuals for individual votes had a letter “P” associated with the number for personal votes. Those issued for institutional votes did not have any letter added. This was because there had been some question as to the role of institutional votes in decisions of Sections and this seemed an easy way to get data on the issue. Members would be asked to deposit their cards into a gorgeously coloured green box for a “yes” vote and a suitably red box for a “no” vote.

**Knapp** commented that for colourblind men, the boxes also had the word written on the side.

**McNeill** continued explaining that another general feature of most rules of procedure was that, for a proposal to be accepted or an amendment to the proposal, a majority must be in favour. It had been the practice of Nomenclature Sections to establish different requirements in certain situations. One of these was the percentage vote required for an amendment to the *Code* to be successful. In line with all recent previous Sections it was proposed that a 60% majority, a so-called supermajority, be required to approve any amendment to the *Code*. For all other matters, except as the Section may later decide, including any procedural matters, the normal, simple 50% majority would apply. He added that this included some situations when there may be a 60% vote for accepting a change to the *Code*, but there may be two alternative ways in which the same effect could be achieved. In a case of choice between alternatives, the Chair may rule that this choice only require a 50% majority. He reiterated that any change to the wording of the text of the *Code* would require a 60% majority for that proposal to be accepted.

The following procedural decisions were taken (**moved** by the Rapporteur, **seconded**, and **accepted** without objection):

- Any proposal to amend the *Code* that received 75% or more “no” votes would not be discussed unless it was moved by one person and supported by four others.
- A 60% majority would be required to amend the *Code* and a 50% majority for all other matters.
- New proposals and substantive amendments to alreadypublished proposals required a proposer and support from four additional people.

The final matter the Section had to consider was whether or not to make a special rule on the majority required in dealing with recommendations by the General Committee for botanical nomenclature on conservation and rejection of names. There had not been one procedure adopted by recent Nomenclature Sections. Prior to the Vienna Congress no special voting provision was ever made in such cases. Until Vienna, had there ever been a vote that needed to be counted, a simple majority would have applied. The Nomenclature Section in Vienna adopted the approach that the special status that the *Code* gave to General Committee recommendations in Art. 14.14 was such that their rejection might be looked on as analogous to reversing a previous decision of a meeting, something for which many rules required a supermajority. Members of the Section were reminded that there was a proposal to amend Division III of the *Code*. Namely Div. III Prop. C proposed that the *Code* require a 60% majority to approve a recommendation from the General Committee.

For the general benefit of people not as familiar with procedures of conservation and rejection of names as others, McNeill outlined that the Appendices to the *Code*, the lists of conserved and rejected names, were developed by a process set out in Art. 14 of the *Code* by which proposals must be published and then considered. They must be sent to the appropriate Permanent Nomenclature Committee for the particular group, the Committee for Algae, Fungi, *Bryophyta*, Vascular Plants, or Fossil Plants, and were then studied. Anyone who wished to make presentations did so and these Committees were required to have a 60% majority for recommending any either positive or negative action. The General Committee then reviewed these to make sure that that *Code* had been appropriately followed and made its recommendation. These recommendations were published and brought to the Section. Until Vienna no special voting provision was made for dealing with such recommendations, which meant that it was a simple majority. In Vienna the procedure adopted was that it should be a 60% majority to reject a recommendation from the General Committee. There was a current proposal to amend the *Code* to require a 60% majority to accept such a recommendation. There was no recommendation from the Bureau, and it was simply being brought to the attention of the Section so that if people wanted to make a proposal, one way or the other, they could do so. If not, like any other proposal it would simply require a 50% majority.

**Knapp** asked if there anyone who wanted to make a proposal on the percentage required to either accept or reject recommendations from Committees.

**Thiele** moved that, as in Vienna, a 60% majority be required to reject a General Committee report or recommendation on conservation and rejection of a name.

**Knapp** clarified that the motion on the floor was to require a 60% supermajority to reject a General Committee recommendation.

[The **motion** was **seconded** and **approved**.]

**Knapp** confirmed that the Section would adopt a 60% supermajority for any proposal to reject a General Committee recommendation and asked if there were any other proposals on the issue. She went on to say that to accept General Committee recommendations would require a simple majority.

**McNeill** clarified that this was not the case, that it would go through unless there was 60% against. What this meant was that if there was opposition to a proposal, then that opposition must require 60% of the votes to carry, otherwise the recommendation would be accepted by the Section of the Congress. He noted that there had only ever once been such a vote.

**Barrie** requested clarification that a 60% requirement would be for specific proposals, not for the report itself. Acceptance of the report would require 50% plus one.

**McNeill** explained that in terms of the normal practice with reports, some reports were simply reporting on what a Committee had done over the last six years. For example, the reports of the Permanent Committees on special groups did not come directly to the Section with particular recommendations unless they were procedural. This meant that the report was received and a 50% simple majority was required to accept it. The only situation under discussion was that those recommendations coming only from the General Committee, involving the conservation and rejection of names, would be accepted unless 60% of the Section voted against them. This would apply whether presented as a package or, should controversy be expected, presented individually.

**Luckow** questioned whether that meant that only 40% of the group had to approve something from a General Committee recommendation, that only 40% of the Section were needed for approval of a recommendation from the Committee.

**McNeill** corrected this to 41% and confirmed that this was the case.

**Luckow** added that this in essence meant that the people in this room were giving up their power to make decisions on nomenclature to a smaller committee.

**McNeill** refuted this and suggested that they were judging that the expertise of the Special Committee was such that it should only be overturned if a really large number of people at the Section decided to do so.

**Luckow** pointed out that there was no discussion of the proposal before we voted on it and she wanted to clarify so that people in the room knew that essentially the General Committee would be making decisions in most cases, not the Section.

**Van Rijckevorsel** added that there was a subtle distinction involved. First, there was a difference between recommendations and reports. A report of the General Committee was published. A recommendation was something put before the Section. Secondly, what had just passed without discussion was that the recommendations that were handed out in the package—so for this event only and for this report only—had been passed unless someone managed to raise more than a 60% majority. It was a unique procedure, only for this Section and without any direct reference to the report that was passed.

**McNeill** clarified that it was perfectly correct that receiving a report was simply a matter of confirming that it is a report of the Committee, that it is in order and that it is not just something the secretary wrote overnight and it has nothing to do with what the Committee did. It was a formality, and the Section would accept the Committee’s report in order to discuss it with a simple 50% majority, and it was unlikely to ever be voted on further. The issue was the matter of recommendations that had been made from the Committees for particular groups to the General Committee and then endorsed by the General Committee with an appropriate majority, 60%, and then presented to the Section. The judgements of each Section were independent, and the report from the General Committee later in the meeting would look then at the recommendations. There may well be more than 60% of people against them. He pointed out that this was not unique, it was exactly the procedure that was adopted in Vienna.

**Knapp** pointed out that a vote had already been taken and there had been an opportunity for discussion, but suggested that more discussion was possible if the Section felt that was needed. She wished to confirm that voting to receive reports required 50% and a 50% simple majority was required to accept the General Committee’s recommendations.

**McNeill** replied that this was not the case, the only situation the proposal from Thiele applied to would be those recommendations made by the General Committee that involved the conservation or rejection of names, material that would eventually appear in the Appendices of the *Code*. Luckow described it perfectly clearly and correctly that this was a situation in which there needed to be at least 41% of the people at the Section voting “no” in order for something to be stopped.

**Knapp** corrected him to 61% saying “yes”.

**McNeill** apologized and amended his comment to clarify that 41% needed to say that they did not want the name in question conserved or rejected otherwise it would be conserved or it would be rejected. [He presumably meant to say that 41% needed to say that they *did* want the name in question conserved or rejected otherwise it would *not* be conserved or it would *not* be rejected.]

**Barrie** offered to try to clearly state what was under discussion using the description of Van Rijckevorsel of the difference between a report and the recommendations. The report from the General Committee, of which everyone had a copy, was something the Section could accept with a 50% majority. After the report had been accepted, if there were any objections to any particular recommendation in the report, that would require a 60% vote in order to overturn the recommendation. This would not mean that 40% of the people are controlling the room, at least in terms of the report as a whole.

**Greuter** offered the point of view of someone who had worked for several periods both on a Special Committee and on a General Committee. The *Code* left matters concerning conservation and rejection of names to special bodies. These were nominated and given a mandate and sometimes recommendations by this Section. He described them as having expertise, doing their work skilfully and graciously, looking into every proposal critically and with considerable input of time. He felt it was one of the strengths of the botanical nomenclatural system. Art. 14.14 of the *Code* ensured that proposals approved by the General Committee were, for practical purposes, accepted under the *Code*. He thought that requiring a 60% majority to overturn a General Committee Recommendation by the Section was fair and logical. He argued that the Section would not have the knowhow or the necessary literature available to form considered opinions on individual cases and suggested that should a Section ever decide to take matters in their own hand, his prediction would be that skilled people would no longer be willing to serve on the Committees. The second point that he wished to make was that the discussion of an individual proposal in the Section was not in order. Had he been present in Vienna in his former capacities, he would not have allowed that because the *Code* does not provide for that. The *Code* provides for rejection, not for approval of the reports of the General Committee. He approved of the way it was going to be handled, in the sense that 50% to accept the report and 60% against to overturn a decision was acceptable, but disapproved of discussion of individual cases.

**Sebsebe Demissew** requested further discussion of the issue, as one of the problems with the *Acacia* issue was that this was not really clear in Vienna. His opinion was that, as in any democratic institution, the views of the Committee should be respected, but that a 60% majority for rejection was too much.

**Knapp** summarized that the General Committee report was received, with a 50% majority, a simple majority. In that report were recommendations. If an individual would like to overthrow that report, then by all rules of parliamentary procedure a higher majority was needed to overthrow something that had been accepted. To overthrow something in that report a supermajority of 60% would be needed.

**McNeill** closed the issue by pointing out that a vote had been taken and unless people who voted “yes” had changed their mind it was time to move on. He introduced the final, normally routine, procedural matter the Section had to deal with before considering proposals to amend the *Vienna Code*. The *Code* was prepared by the Editorial Committee appointed in Vienna charged with implementing the decisions that the Congress, through its Nomenclature Section, had made in Vienna. It was, of course, produced after the Congress, and the Section now had to ratify the *Vienna Code* as reflecting the decisions of the Vienna Congress before it became the basis upon which amendments could be proposed. He moved the **motion** that the Section ratify the *Vienna Code*; this was **seconded**.

**Gereau** called for more discussion as ratification of the *Vienna Code* without any further discussion would have completely ignored a number of issues that had been widely discussed and widely accepted as in need of discussion by a large number of people.

**Knapp** opened the floor for discussion.

**Luckow** felt that part of the reason there was a problem with ratifying the *Code* had to do with the *Acacia* issue. The argument had been made by Gerry Moore that parliamentary procedure was breached. She requested clarification as to whether ratifying the Vienna *Code* would include automatically accepting the *Acacia* decision, or whether that could still be brought up as an issue.

**McNeill** replied that ratifying the *Code* would mean acceptance of the *Code* as it is, including the conservation decision on *Acacia*. He went on to say that it did not rule out any amendments to the *Code* that could be proposed. Two such proposals had already been published: one by Dick Brummitt that had received very negative support in the preliminary mail vote; and another by Nick Turland that would require support by five people for it to be discussed. He was clear that socalled compromise proposals to amend the *Code* relating to *Acacia* were quite open for discussion. He was also clear that by accepting the *Vienna Code* as it stood, the Section would be agreeing that it was correct and *Acacia* was conserved with *Acacia penninervis* as type. He explained that the only argument against that would be that the Vienna Section acted improperly and took an invalid decision by adopting the procedure in question. He then pointed out that given what the Section had just adopted for the Melbourne Congress, it would be rather hard to argue this case. No Section would have the power to change a conservation proposal; once a name was conserved and in the *Code* it may never be deleted. The *Code* would have to be amended to alter this and it would apply to all names. He finished by saying that discussion of the so-called compromise proposals regarding *Acacia* was still open, but discussion on the matter of the conservation of *Acacia* was closed.

**Knapp** clarified that the ratification of the *Vienna Code* was the basis for the following debates with a simple majority vote as a procedural matter. A “yes” vote implied acceptance of the entire *Code*, including the voting procedure used to approve or reject the Committee reports presented in Vienna. A “no” vote implied the rejection of some part of that process.

**Van Rijckevorsel** wanted to make a small clarification regarding what he considered were a few historical inaccuracies in the explanation by Greuter. He accepted the accuracy of the broad picture, namely that there had never been any discussion on recommendations by the General Committee, but took exception to the suggestion that the power to approve had been transferred to those Committees. Historically he claimed this was inaccurate because the power to decide had always lain with the Section, despite it never having exercised it nor established any associated procedures. The matter before the Section was whether the printed book was an accurate representation of the decisions taken at Vienna, and many felt that it was not because it was handled improperly. He suggested the alternative that it was possible to accept the book minus the *Acacia* entry, as a phantom entry, something that was printed inadvertently.

**Knapp** explained that the current discussion was of the proposal to accept the *Vienna Code* as it was printed and that anything else would have to be a new proposal.

**Thiele** reiterated McNeill’s point that the principal objection to the *Vienna Code* had been that the method of voting that pertained in Vienna was incorrect, improper and invalid. Given that this Section had just made the same decision on the voting mechanism he agreed that it would be a tall order to try to argue that the Vienna process was invalid. He suggested that the Section should accept the *Code* as printed as the basis for the meeting.

**Rico** thought that the *Code* was correct with the exception of one single entry in Division III, which was the re-typification of *Acacia*. As she understood it, this was a provisional entry that needed to be ratified here. If the Section ratified the *Code* as it stood, this particular issue would be forgotten.

**Knapp** reiterated that voting “yes” on ratifying the *Vienna Code* meant acceptance of the entire *Vienna Code* and ratification of it as the basis for further discussion. Voting “no” meant an objection to some part of the procedural process.

**Gandhi** requested that the choices be shown on the screen.

**Knapp** accepted this suggestion. She explained that she was not ignoring people but trying to balance out the debate between those for and against ratifying the *Code* as it stood and asked if there was anyone who would like to speak for ratifying the *Code*. [No-one stepped forward.]

**Luckow** disagreed with Thiele in the sense that the situation for *Acacia* was different, as pointed out by Greuter, because the 60% vote was a particular case just for *Acacia*. She maintained that it was a very different situation and that what had just been voted on was not the same thing as in Vienna.

**McNeill** in turn disagreed with Luckow, stating that it was quite clear from the record that the proposal for the procedure with regard to a 60% majority to overturn any recommendation of a Permanent Committee [the General Committee] took place prior to any discussion. It applied to all the recommendations, not just to *Acacia*. The fact that *Acacia* was the only one that went to a vote was the only thing that was different. The same procedure would have applied for each of the other names had there been any questions so it was indeed the same.

**Knapp** decided to exercise her prerogative as Chair and pointed out that the Section had not convened to discuss the past. She repeated the importance of moving forward as a community. The issue under discussion was whether the Section would ratify the *Vienna Code* as a basis for the following debates with a simple majority vote as a procedural matter. Again it was explained that the proposal on the floor was that the Section either accepted the *Vienna Code* including the *Acacia* decision or did not accept the *Vienna Code* and then would move on to what to do next.

**Knox** questioned whether it would be possible to propose a modification for the ratification because the *Acacia* issue was obviously something that concerned many people, some were heavily invested in the debate. He identified a desire to avoid making a mistake at an early procedural stage that might somehow box in any possible later discussion. He stated that it was obvious that the Section needed to ratify the *Vienna Code* but wondered if it was possible to propose a modification that we ratify all of the *Vienna Code* except for the points pertaining to *Acacia*.

**Knapp** confirmed that this was possible but that no-one had made such a proposal.

**McNeill** observed that there would have to be some rationale for such a proposal explaining why the Editorial Committee failed in its duty.

**Funk** called the question.

**Knapp** explained that when someone called the question a supermajority of the Section, 60%, needed to agree that it was time to vote. [The Section voted on moving to a vote on the ratification of the *Vienna Code*.] She reported that there was a very clear majority for moving to vote on whether the Section ratified the acceptance of the *Vienna Code* in its entirety.

[A card vote was called for.]

**Knapp** repeated that the vote was a simple majority, 50% plus one for the motion to carry; “no” meant rejection of some part of the process used to arrive at the *Vienna Code*, “yes” meant acceptance of the entire *Vienna Code*. Ratification was presented as a formal **motion**, which had earlier been **seconded** and this was **approved** in the card vote [373: 172; 68%].

**Knapp**, during the count of the card vote, returned to one of the traditional pieces of business skipped at the beginning of the Section, the list of those botanists who had died in the period between the two congresses prepared by Dan Nicolson previously, and this time by Larry Dorr and Dan Nicolson. Unfortunately the list was far too large for every name to be read out. She expressed thanks to both Larry and Dan for putting in a huge effort and finding a lot of people who had passed away and who were not recorded at the last Congress. She took the opportunity to mention just a few people in particular from the list: Rogers McVaugh and Piers Trehane, who were contributors and editors on the *Code* and had contributed a great deal to nomenclature over the years, Santiago Castroviejo, who was a much-valued member of the IAPT Council, Armen Takhtajan, well known to students and an addition since the list closed on 30 June, and Paul Fryxell. Because the Section was in Australia and it was not possible to read all the names, she had identified all the Australians who had passed away: David Ashton, Nicholas Batianoff, John Stanley Beard, Roger Black, Jenny Chappill, George Chippendale, Lori Cobb, Betty Conabere, Ed Cross, Edgar Dell, Thamarapu Desikachary, Ludwik Dutkiewicz, Rica Erickson, Helen Hewson, Surrey Jacobs, Bob Royce, Dorothy Shaw, Mary Tindale and Bryan Womersley. She finished by adding to the list a muchvalued friend and colleague who could be considered an honorary Australian: Chris Humphries from the Natural History Museum.

**McNeill** then introduced another traditional motion: “that for the revised *Code* to arise out of this Congress, the Editorial Committee [yet to be appointed] be empowered to change, if necessary, the wording of any Article or Recommendation and to avoid duplication, to add or remove Examples, to place Articles, Recommendations, and Chapters of the *Code* in the most convenient place, but to retain the present numbering in so far as possible, and in general to make any editorial modification not affecting the meaning of the provisions concerned”.

[The **motion** was **seconded** and **approved**.]

**Knapp** noted that there were a number of compromise proposals about the typification of *Acacia* that had been put forward, one of which was heavily defeated in the mail vote, and another of which would need to be proposed from the floor. She suggested that the Section should decide whether to discuss these proposals early on or leave them until later.

**McNeill** mentioned that there had already been quite an extensive debate and unless the Section proposed otherwise this issue should be dealt with at an appropriate point in the proceedings. In planning the meeting it was felt that it might be better to concentrate on *Acacia* issues in the first session to look at the implications and decide what to do on a further day, but that was up to the Section to decide. He invited anyone who would like to consider the various published compromise proposals and any others that might arise, otherwise the normal procedure of proposals would begin, dealing with the general proposals first.

**Knox** proposed the motion that a fixed amount of time was set aside for an initial discussion on the compromise proposals to get everybody up to speed on the issues regarding the typification of *Acacia*, but decisions were deferred until the scheduled time in the meeting. This would allow an initial airing of people’s thoughts on the matters so that during lunch and other free times, people could reasonably discuss amongst themselves what some of the implications were.

[The **motion** was **amended** to specify that the limited amount of time would be 30 minutes and **seconded**.]

**Ladiges** queried when the decisions would be made.

**Knapp** explained this would be at the right point in the *Code*.

**McNeill** added that it would depend on the form that the first discussion took.

**Passalacqua** wished to return to the earlier debate and decide on rules in the *Code* for accepting or rejecting recommendations.

**Knapp** pointed out that this was not germane to the proposal to have a 30 minute discussion on the compromise proposals for the typification of *Acacia*.

**Passalacqua** apologized.

**Greuter** spoke against the amended proposal giving two reasons. First, he expressed a wish that the proceedings of the nomenclature of the Melbourne Congress would be published more rapidly than those of the Vienna Congress, and explained a procedural reason that it was easier for those preparing the proceedings if the proposals were discussed in the appropriate place where they appeared in the synopsis, and suggested that it was also easier for the participants to keep a record. The second reason he gave was that waiting may result in one or other of the proposers withdrawing their proposal.

**Glen** supported the proposal for a half an hour discussion because some of the Section members were still too jetlagged to make sensible decision on this important issue.

**Lewis** also supported the motion and suggested that the 172 votes against the earlier motion were probably from those who had a specific interest in the *Acacia* vote. He agreed with West that there was a need for the Section to be transparent, clear and honest. He expressed a worry that if the discussion was put off for too long, because of the underlying current of disagreement, the two sides looking at *Acacia* were going to continue to niggle at each other, putting various impositions on the whole proceedings. He argued that the Section needed to try to come to some sort of clarity and vote on the issue later.

**Knapp** reminded the Section of the proposal: to have a half an hour discussion of the *Acacia* compromise proposals now and to vote on those proposals at a later date in the week, when they come up at the appropriate time in the *Code*.

[The **motion** was **approved**.]

[*The general discussion on the Acacia issue took place here but has been moved to prior to Art. 29 in accordance with a logical order.*]

**Knapp** moved to the order of business usually undertaken in the Nomenclature Section, namely starting at the beginning of the *Code* with proposals to change the *Code*.

**Greuter** strongly suggested to the Bureau that they number the sheets handed out for writing up the comments. This was in their own interest as he felt those reporting on the Congress would be completely lost otherwise.

**Knapp** reiterated that the comment sheets needed to have name, institution and city, the date and time filled in including whether it was morning or afternoon so that the comments could be correlated with the recording.

### General proposals

* indicates that the Rapporteurs suggested a special meaning for an Editorial Committee vote in the preliminary mail vote.

**Prop. A** (87: 27: 4: 2).

**McNeill** introduced General Prop. A, from Hawksworth and others in *Taxon*. The proposal was to establish more clearly that the *Code* covered mycology, the study of fungi, as well as botany, commonly defined as the study of plants, by a series of insertions in the *Code*. He specified that it would involve inserting “and mycological” after “botanical” in the title of the *Code*, replacing “requires” by “and mycology require” and replacing the word “plant” by the words “plant and fungus”, and inserting “and mycologists respectively” after “botanists” in Pre. 1 and inserting in Division III, in the endnote [Div.III.1 footnote], “and mycological” after “botanical”. The aim was to make the *Code* a little more politically correct in referring to fungi as well as algae. He invited the proposer to speak.

**Hawksworth** gave background to the proposal. At the International Mycological Congress in Cairns in 2006, there was a strong feeling that the move should be made to a separate code for mycology quite independent of the botanical *Code*, and the same view had emerged at several other mycological meetings in recent years. He was not in favour of having a proliferation of codes, and therefore this proposal was put together to avoid having a separate code. It was voted on at the International Mycological Congress in Edinburgh in 2010, where there was strong support: 71% of the mycologists present were in favour of the proposal. If the proposal was not accepted by the Section meeting he suggested that a new code for mycology would be inevitable.

**McNeill** added that the proposal received 87 for and 37 [sic!] against in the mail vote, and it had been considered by the Nomenclature Committee for Fungi, which supported it with 78% for.

**Prud’homme van Reine** introduced himself as secretary of the Nomenclature Committee for Algae, and continued that they were very unhappy with this proposal. The proposal meant to establish more clearly that the *Code* covers mycology as well as botany, but he wanted to know why this was not extended to the algal groups in the kingdoms *Chromalveolates* or *Stramenopiles*, the *Excavates*, the *Rhizaria* etc., referring to a 2004 paper by Patrick Keeling, in the *American Journal of Botany* [DOI: http://dx.doi.org/10.3732/ajb.91.10.1481]. He pointed out that the fungi and the animals both belong to the unikonts, the only eukaryotic kingdom that included no algal groups, adding that the *Microsporidia* were closer to the fungi than any group of animals. Within the Nomenclature Committee for Algae there was very little enthusiasm about changing the name of the *Code* or replacing in the *Code* the word “plant” with “plant or fungus”. They maintained that there were already too many details in the *Code* that were only of interest of mycologists. The suggestion to add “mycology and phycology included” between brackets to the title of the *International Code of Botanical Nomenclature* did not get enough positive votes in the Committee for Algae to make it possible to add this suggestion as one coming from that Committee. However, he offered this suggestion as a individual. He summarized that every addition of mycology or mycologists and comparable terminology to plants in general is unacceptable for the members of the Committee for Algae. What they wanted was a simple, clear *Code* that covered all, not a complex, muddied *Code* that covered bits and pieces. This was the reason he gave to reject the proposal.

**Gams** was strongly in favour of the proposal to avoid having separate codes. He found the case for a BioCode very debatable, and a MycoCode even more. He observed that the fungi are heterogeneous, just like the algae, as Prud’homme van Reine had pointed out. They contain some biflagellate, zoosporic fungi as well as the *Chromista*, but for nomenclature, they were “the fungi”. He added that algae had always been taught in connection with botany, but fungi were increasingly being recognized as a separate group of organisms in their own right.

**Demoulin** outlined his background in both mycology and phycology and agreed with Prud’homme van Reine but felt that in the political context of the day, even if he considered it silly to make all the additions in the *Code* (he had abstained in both the Mycological Congress and in the mail vote on this issue), he agreed with Hawksworth and Gams that it was necessary to accept the proposals. This was to avoid more temptation of secession. He advocated moving to less and less diverse rule and hoped eventually for a BioCode.

**Gandhi** conveyed support for the proposal from the Harvard University Herbaria.

**Greuter** observed that this had been discussed before and at that time mycologists accepted that “botany” could cover them but not “plants”. He pointed out that there was something to the same effect already in the *Code*, a footnote to the Preamble that read: “In this *Code*, unless otherwise indicated, the word ‘plant’ means any organism traditionally studied by botanists”. He suggested that this might be slightly better worded to speak of organisms treated as plants in botanical tradition. Regarding the second proposal, to speak of plants and fungi, or plant and fungus, or fungus in all relevant parts of the *Code*, he suggested one might then substitute “plants” by “organisms treated as plants”, which would also cover all the algae, even the blue-greens, which are still dealt with under the botanical *Code* by a majority of those who worked with them, and would avoid proliferation or the lengthy expansion of the familiar title in botanical tradition, the *International Code of Botanical Nomenclature*.

**Hawksworth** remembered developing the footnote as a stopgap, but the reality was that was not acceptable to mycologists. He reiterated that if the situation was not changed, then there would be a parting of the ways.

**McNeill** asked Hawksworth if it would be feasible to avoid “plants and fungi” everywhere by using “organisms” more extensively.

**Hawksworth** agreed that “organism” would be an acceptable alternative instead of “plant and fungi” if it was made clear in the Preamble that “organism” covered the different groups.

**McNeill** asked if Hawksworth was prepared for his proposal to include the option for the Editorial Committee to seek to remove “plants” and replace it by “organisms”. [He was.]

**Glen** noted that “traditionally treated as plants” was all very well if you knew what the tradition was but felt that a generation or two in the future may no longer have that knowledge. On those grounds he would vote for the proposals because it offered a clear definition and would still allow generations in the future to understand it.

**Annette Wilson** agreed with Greuter that the footnote itself seemed rather clumsy in using the concept of tradition, and wondered if it would be possible to have a statement at the front of the *Code* setting out which organisms are covered, and then using the single word “plant”. She disliked the use of the word “organism”, because it could create confusion with zoology.

**Harley** suggested that the Preamble could include something to the effect that it covered all organisms not already treated in the *Zoological Code*.

**McNeill** pointed out that there was also a bacteriological or prokaryote *Code*.

**Gereau** explained that organisms covered by the *Code* were already defined in Pre. 7 and this could be editorially moved to a more prominent position. He felt that the issue was political. The mycologists wanted the changes in the *Code* in order to retain the use of the *Code*. If having mycologists adopt a second code was highly undesirable, he felt that the Section should vote for Gen. Prop. A, otherwise they should vote against it.

**Herendeen** disagreed that it was only a political thing. He claimed that the title of the *Code* was also biological and the *International Code for Botanical, Mycological and Phycological Nomenclature* was biologically sensible. He suggested staking out the ground covered in the title of the book, and then coming up with some more succinct terminology to use inside. He pointed out that some of the phycologists could already decide which *Code* they wanted to publish under.

**Kirk** explained that it was the “P” word that mycologists object to: “plant”. He supported Greuter’s suggestion to use “organism”. He offered the radical solution of removing the title, and just calling it the *ICBN*, without an expansion.

**Knapp** asked Hawksworth if he would consider that a friendly amendment.

**Hawksworth** thought too many mycologists would remember what the “B” stood for. [So would not.]

**Malécot** explained that in the *Zoological Code*, the first Article, 1.1.1, was “For the purposes of this *Code* the term ‘animals’ refers to the Metazoa and also to protistan taxa when workers treat them as animals for the purpose of nomenclature”. He felt that this would be desirable in the botanical *Code* and suggested moving Pre. 7 to Pre. 1, with some editorial modification to treat all of mycology and phycology.

**Wiersema** expressed the support of his mycological colleagues from the United States Department of Agriculture for including mycology in the title and asked for clarification as to whether the friendly amendment to change “plants and fungi” to “organism” had been accepted.

**McNeill** had the impression from Hawksworth that it would be acceptable if “plants and fungi” was replaced everywhere with “organisms”, having spelt out which organisms were covered by this *Code*. The issue of the title was a different matter.

**Wiersema** commented that the friendly amendment would have implications for other proposals such as the next one, Prop. C where the word “plant” appeared using “fossil plant” instead of “plant fossils”.

**Barkworth** wondered if Herendeen had formally proposed an amendment to add phycology as well as mycology to the title.

**Herendeen** was suggesting that revising the title to reflect the fact that this *Code* covered fungi and algae was a sensible thing to do. He formally moved a **motion** to amend the proposal to modify the title to be the “*International Code for Botanical, Mycological and Phycological Nomenclature*”. [The **amendment** was **seconded**.]

**Prud’homme van Reine** reported that the Nomenclature Committee for Algae was not in favour of putting phycology in the title although it was very much in favour of using “organism” instead of “plant”.

**Alvarado** also thought it would be a good thing to include phycological as well as mycological because, if people who work with fungi wanted to include their discipline, it was fair that people who work with algae should also be explicitly included. He elaborated that the three disciplines currently existed, even if the group that phycologists studied was polyphyletic.

**McNeill** asked for clarification from Prud’homme van Reine as to the rationale for the Committee for Algae being unhappy with including the word “phycological”.

**Prud’homme van Reine** repeated that the desire was for a clear *Code* that covered all, not a complex, muddied *Code* that covered bits and pieces. He did not think it necessary to enter all the divisions and four kingdoms of algae.

**McNeill** asked for further clarification as to whether the word “phycological” did not cover the four kingdoms of algae.

**Prud’homme van Reine** clarified that it did, but that the Committee for Algae thought it was unnecessary to make the *Code* thicker again, but that use of the word “phycological” was not wrong.

**Demoulin** requested clearly separating the sensitive issue of the title from the issue of what was going to be used in the rest of the *Code*. He thought there was some agreement about using “organism treated as plant” instead of “plant”, which for editorial purposes he thought would be much easier, but that this discussion should be left for later. He felt that it was politically important to add mycology in the title. Given that he thought the Section should do that, even though he thought it was silly, he wanted to know if the phycologists still considered they were covered by “botanical” and still did not want to be added, or, if it was inescapable to have “mycological” in the title, he wanted to know if they would prefer to also have “phycological” added.

**McNeill** thought Demoulin was asking for a representative from phycology to establish for the Section if it would be better from an algal perspective to have “botanical and mycological”, or “botanical, mycological and phycological”.

**Knapp** asked if there were any comments on that from the phycological community.

**Prud’homme van Reine** [who appeared to be the only phycologist present] accepted that, if there was nobody else, he would have to comment. He admitted that he made the same proposal himself in the Committee for Algae, but the Committee did not want it, although he himself wanted it, very much. He joked “if nobody tells the others, yes, do it!” [Laughter.]

**Head** supposed that he represented the palaeophycological community and felt that if the Section were to go with fungi specifically, then algae should also be included. He supported the amendment to include in the title both “mycology and phycology” because it explained what the *Code* covered concisely and truthfully.

**Knapp** checked there were no more comments and that the Section was ready to move to a vote on the amendment to the title. The proposal would read, instead of inserting “mycological” after “botanical” in the title of the *Code*, that “mycological and phycological” would be inserted after “botanical” in the title of the *Code*.

**McNeill** pointed out that this was not the final decision. This was simply moving from talking about “botanical and mycological”, to talking about “botanical, mycological and phycological” if the amendment was passed.

**Knapp** confirmed that everybody understood what was being voted on and that the Section would vote on the amended proposal afterwards. She noted that it would be a simple majority vote. [The **amendment** was **accepted**.]

## Second session

Monday, 18th July 2011, 14:00–17:50

**Knapp** took the opportunity after lunch to reiterate a couple of housekeeping points. Greuter had brought up an issue about the comment slips and she explained again that it was important to fill in the date and time. The Nomenclature Section photograph was scheduled for Tuesday afternoon at 2:30 pm, and she encouraged everyone to come “dolled up”. She reported the personal and institutional votes for the earlier vote ratifying the *Vienna Code* in an attempt to gather data about the influence of the different votes. The total was 373 “yes” and 172 “no”, a simple majority [68% in favour]. The institutional votes were 247 “yes” and 136 “no” [65% in favour]. The personal votes were 126 “yes” and 36 “no” [78% in favour]. She concluded that “no matter how you cut the pie, it came out the same way”. Then she returned the discussion to the proposal to change the Preamble. The proposal had been amended to read “inserting ‘mycological and phycological’ after ‘botanical’ in the title of the *Code*”. Hawksworth, who introduced the proposal, had also accepted editorially altering of “plants” to “organisms”.

**May** moved an **amendment** to Prop. A (iii) that the footnote on page one of the *Code* state “In this *Code*, unless otherwise indicated, the word ‘organism’ means any organism traditionally studied by botanists, mycologists or phycologists.” [The **amendment** was **seconded**.]

**Knapp** repeated that the proposal to be discussed was an amendment to change the footnote to say “plant means any organism traditionally studied by botanists, mycologists or phycologists” and then to replace the word “plant” throughout with “organism”.

**McNeill** clarified that, as qualified here, “organism” applied to all the organisms covered by the *Code*, as opposed to special use of the word. He pointed out that it was quite possible that in Prop. B retention of the word “plant fossil” would be acceptable and that would be for the palaeobotanists to decide.

**Greuter** wondered if this could just be referred to the Editorial Committee.

**Knapp** explained that an amendment had been proposed, which was being discussed, and would hopefully be voted on quite soon.

**Gams** suggested a **friendly amendment**. As the algae are usually much closer to plants than fungi he suggested reversing the sequence: “botanical, phycological and mycological”.

**Knapp** felt that this might be able to be dealt with editorially.

**McNeill** added that the alphabet dictated something different, so that view might not be accepted for that reason.

**Prud’homme van Reine** had been thinking that, since the higher plants are nested in the green algae—the “dry greens”—it would be possible to get rid of the “B” word—botany—and just call the *Code* the *International Code of nomenclature of mycology and phycology*. He followed this quickly with the comment that this was not a proposal.

**Herendeen** suggested that in some cases, strictly going with the change to “organism” all the time might be awkward and it might be easier to leave some of these editorial changes for the Editorial Committee to decide, based on what made most sense in terms of prose that worked. He recommended not getting too caught up in precise requirements to replace this word with that word throughout the *Code* and allowing the Editorial Committee to fine-tune it as appropriate.

**McNeill** thought that comment was very apposite to Prop. B, which would be discussed in a moment.

**Karen Wilson** requested that in part (iii) of Prop. A the words on the screen be altered to replace “plant” with “organism”.

**Ladiges** wanted to know if the very first sentence would have to be changed “The *Code* covers mycology, the study of fungi, as well as botany”.

**Knapp** elucidated that this was part of the proposal, not part of the *Code*. She moved to a vote on amending the footnote to read “mycological and phycological” after “botanical”. [The **amendment** was **accepted**.]

**Buck** called the question on the proposal, so that the Section could finish at least one proposal on the first day.

**Knapp** thanked him and called a vote on voting on the proposal. [There was a sufficient majority in favour of voting.] Then she proceeded to the actual vote on General Prop. A.

**Greuter** suggested a separate vote on item (i), for the title.

**Knapp** clarified that the Section had to vote on the whole proposal because the question had been called and a two-thirds majority of those assembled had said they wanted to vote. She read out the amended Prop. A, which was to insert “mycological and phycological” after “botanical” in the title of the *Code*, replacing “requires” by “mycology and phycology require” at the start of Pre. 1, replacing the word “plants” by the word “organism”, inserting “mycologists and phycologists respectively” after “botanists” in the footnote to Pre. 1 and inserting “mycological and phycological” after “botanical” in Div.III.1 footnote 1.

**Prop. A** was **accepted** as amended.

[*The following discussion of a new proposal by Norvell, to amend the title of the Code, took place during the Tenth Session on Friday afternoon.*]

**Norvell’s proposal**

**McNeill** noted that the Section had already decided to make changes to the title of the *Code* and understood there was some rethinking coming from the people who had proposed the changes and they were suggesting a modification of what had previously been agreed. [The **proposal** was **seconded** and **supported** by three others.]

**Norvell** recapped that the new title was the *International Code of Botanical*, *Mycological and Phycological Nomenclature*. She (in consultation with others) suggested that the *International Code of Nomenclature for algae, fungi and plants* would be easier as it covered everything that the *Code* covered.

**Demoulin** thought that this was interesting because it was parallel to what the bacteriologists had done with the change from *International Code of Nomenclature of Bacteria* to *International Code of Nomenclature of Prokaryotes* and it also avoided taking a stand in the controversy between “algological” or “phycological”.

**Alvarado** disagreed with the proposed name. He felt that the previous one was better because the three disciplines exist, but he felt that “algae, fungi and plants” excluded *Cyanobacteria*… [Audience dissent.]

**Knapp** instructed that there was to be “No violence in the Nomenclature Section!”

**Alvarado** continued that phycology included *Cyanobacteria* because phycology worked with algae and *Cyanobacteria*, but algae alone did not include *Cyanobacteria*. He preferred the first name and not the second one.

**Hawksworth** pointed out that the words were deliberately in lower case and reflect how they were used in the committee structure. He elaborated that fungi written like that included the slime moulds, for example, and other groups of algae that did not happen to have chloroplasts; and algae used in that sense meant things studied by algologists, including the *Cyanobacteria*. He thought it was very clear.

**Knapp** summarized that the vote was on changing the title of the joint *Code* from the *International Code of Botanical, Mycological and Phycological Nomenclature* to the *International Code of Nomenclature for algae, fungi and plants*.

**Norvell’s proposal** was **accepted**.

[*Here the record reverts to the normal sequence of events.*]

**Prop. B** (80: 25: 11: 2).

**McNeill** explained that “plant” would be replaced by “organism” not “plant and fungus”.

**Barrie** shared Herendeen’s concern that this would not be a rigid replacement. As a member of the Editorial Committee, he felt it could cause problems if forced to do this everywhere. In terms of keeping the text sensible and coherent he recommended some freedom and leeway and suggested referring this to the Editorial Committee.

**McNeill** clarified that this was an amendment to refer the proposal to the Editorial Committee. He felt that to some extent it was covered in the wording of the proposal: “where this is intended to include all organisms covered by the *Code*”. “Plant fossil”, for example, was not intended to cover all organisms.

**Herendeen** blamed living in Washington too long, but maintained that some could read it to be very strict instructions on replacing the wording in all cases by another word. He felt more flexibility was needed than what a strict reading of the proposal might suggest to someone.

**Knapp** reminded the Section about what was voted on in the very beginning session: that the Editorial Committee was required and requested by the Section to make sensible changes.

**Reveal** suggested a friendly amendment that, after “Editorial Committee to replace ‘plant(s)’”, the words “as appropriate” be added. [The **friendly amendment** was **accepted**.]

**Knapp** moved to a vote on Prop. B, to instruct the Editorial Committee to replace “plant(s)” where appropriate by the word “organism(s)” throughout the *Code*, where it was intended to include all organisms covered by the *Code*.

**Prop. B** was **accepted** as amended.

**Prop. C** (90: 7: 16: 7).

**McNeill** introduced a quite different world, that of fossil plants. Prop. C was part of a series of proposals by Cleal & Thomas designed to clarify that plant fossils were being considered. The proposers were not present. The proposal received very positive votes in the mail vote, 90 to seven. Furthermore, it was almost unanimously supported by the Committee for Fossil Plants.

**Herendeen** introduced himself as secretary of the Committee for Fossil Plants. He had discussed whether the wording was exactly right with Martin [Head], because fossil fungi and fossil algae were also considered. He wanted to modify the wording so that the text replacement would refer to plant, fungal and phycological or plant, fungal and algal fossils.

**McNeill** felt that this was editorial.

**Van Rijckevorsel**, in view of what had just been passed, suggested it may be possible in some cases to just use “fossil” rather than “plant fossil” as that would be much less clumsy.

**McNeill** thought that this was something that should definitely be considered by the Editorial Committee. The point of the proposal initially was to make a clear distinction that these are in fact plant fossils, not fossil plants, and there was a very important procedural and philosophical distinction being made in the proposal.

**Knapp** reiterated what the Section would be voting on: changing the words “fossil plant” to “plant fossil”, which was a conceptual difference between a fossil plant and a plant fossil, but that would be dealt with editorially.

**McNeill** clarified that there would be a recommendation that the Editorial Committee deal editorially with whether it should be “plant fossil” or “plant, fungal and algal fossil”.

**Funk** requested clarity on what was being voted on. She did not think that the changes being proposed were what the Committee for Fossil Plants discussed and came out in favour of.

**McNeill** explained that the proposal itself was quite clear. The substantive issue was changing the word “fossil plant”, which implied dealing with plants as whole organisms, to “plant fossils”, but, as Herendeen had pointed out in the light of what had just been decided with regard to fungi, the editorial part was whether “plant fossil” was used or “plant, fungal and algal fossil”.

**Funk** added the option of just “fossil”. If this is the *Code* of plants, algae and fungi, unless specifically talking about one of those three groups she suggested it was unnecessary to specify plant, fungal or algae.

**Herendeen** agreed completely that there would be places where “fossil” would be fine. To make the proposed change consistent with what had just been decided, he thought there was a need to consider broadening it as necessary. Some of the changes could be handled by the Editorial Committee.

**Malécot** noted that there already was a definition of fossil in the text, under Pre. 7 footnote 1: “In this *Code*, the term ‘fossil’ is applied to a taxon when its name is based on a fossil type, and the term ‘non-fossil’ is applied to a taxon when its name is based on a non-fossil type”. He argued that as “fossil” was already defined in the *Code* it would be possible to change all references from “fossil plant” into “fossil”.

**Knapp** repeated that the vote was on changing the words “fossil plant” to “plant fossil”, and, as editorially appropriate to “plant, fungal or algal fossils” instead of “fossil plants” in the *Code*. The Editorial Committee would be able to use their discretion.

**Prop. C** was **accepted**. [McNeill & al. in Taxon 60: 1511. 2011 noted this proposal as amended, but it was not officially amended according to the recording of the Nomenclature Section.]

### Preamble

[*For clarity, the normal sequence has been restored. The following debate, pertaining to Preamble Prop. A, took place during the Seventh Session on Thursday morning with discussion on Art. 45.*]

**Prop. A** (88: 17: 7: 2).

**McNeill** introduced Preamble Prop. A, that in paragraph 7 of the Preamble after “slime moulds” the following phrase, “but excluding the phylum *Microsporidia*”, be included.

**Redhead** explained that *Microsporidia* were a peculiar group with thousands of species and potentially hundreds of genera. The community that dealt with the *Microsporidia* had always considered them to be protists and assumed they were covered under the *International Code of Zoological Nomenclature* for many years. They continue to publish them as if they were covered under the *Zoological Code*, even though they may recognize that they may be fungi phylogenetically. Under that *Code* the names could be published without Latin. He reported that the entire community of people working with *Microsporidia* wished to be excluded from the *Botanical Code* and continue on as they had traditionally, being covered under the *Zoological Code*, and this had been cleared with the Commission for the *International Code of Zoological Nomenclature* before making the proposal.

**Prud’homme van Reine** spoke on behalf of the algal group who had been asked for a recommendation by mistake. The recommendation was not to accept this proposal. The reason he gave was that no group of organisms had ever been excluded from the *Code*, except perhaps in the Footnote 2 of Pre. 7 “For the nomenclature of other prokaryotic groups, see the *International code of nomenclature of bacteria*”, the *Code* of prokaryotes. The algal group felt that excluding a group of organisms from a code of nomenclature is confusing the independent functions of a nomenclature and classification. In the new prokaryotes *Code*, the first principle is “Nothing in this *Code* may be construed to restrict the freedom of taxonomic thought or action”. He thought that was also what the *Botanical Code* did. Moreover, as explained in the 17th Report of the Nomenclature Committee for Fungi about the microsporidians, it was stated that “Molecular phylogenetics supporting placement of the phylum *Microsporidia* within the fungi are recognized nomenclaturally in the *Vienna Code*, with most recent papers treating the microsporidians as fungi”. He exhorted the Section not to accept the proposal.

**David** pointed out that the Preamble actually said “The rules and recommendations apply to all organisms traditionally treated as plants…”. *Microsporidia* had not been traditionally treated as plants and had been under the *Zoological Code* up until very recently. He was against the proposal.

**Demoulin** noted that there was a difference in opinion between Redhead and himself about whether the *Microsporidia* are such a special group that they need a special rule, one of the reasons he made this general proposal was the reason that Prud’homme van Reine had given, no necessity of a special ruling for *Microsporidia*. He agreed that most people would probably use the *Zoological Code*, but felt there would be no harm in somebody wanting to transfer them to the *Botanical Code*. At Vienna it was thought that the issue had been taken care of, just like *Pneumocystis*, but there was a loophole that Redhead found. His opinion was that, now the loophole was fixed [see discussion later in proceedings: Art. 45 Prop. B], a special ruling for this organism was no longer necessary.

**Kirk** wondered if the Section was proposing to ignore the wishes of the people who studied *Microsporidia*. He added that it would lead to chaos because there would be two alternative naming mechanisms for *Microsporidia*. One group would recognize names as valid and the other group invalid and vice versa. The users of names outside systematics would further disregard the pronouncements made at these meetings in the future.

**McNeill** summarized the two separate arguments he had heard against the proposal. The one argument was from David, who felt that the *Code* already said that it covered organisms traditionally treated as plants and that clearly excluded the *Microsporidia*. But he also got the impression from the other proposers that they were saying that there was a mechanism by which, even if they wrote the description in English prior to 2012, under the provisions that had been approved with Demoulin’s proposal [Art. 45 Prop. B] a name would be validly published under the *Code*. They were suggesting that in fact it did not matter because the *Microsporidia* could be in one or the other *Code*. If this were the case, he felt that this would be an extremely dangerous proposal to vote down, because it would lead to a situation in which it was not known which generic names were homonyms under the *Code*. He had every sympathy with the argument that the proposal was not needed because the *Code* already said the *Microsporidia* were not covered, but if the argument was that they might become covered then that would be very dangerous and he suggested it may be prudent for the Section to support the proposal.

**Harley** felt that there needed to be some sort of liaison with those responsible for the *International Code of Zoological Nomenclature*.

**McNeill** assured him that that had already happened, and because *Microsporidia* were organisms that had fallen previously under the *Zoological Code*, that *Code* had a similar provision and they could continue to fall under it.

**Demoulin** suggested a friendly amendment to take care of what David had said: to add to the footnote of the Preamble something like “*Microsporidia*, having never been treated as plants traditionally, are not to be treated under this *Code*”.

**McNeill** suggested “excluding the phylum *Microsporidia*, never having been treated under this *Code*”.

**Demoulin** thought it was important to explain that it was from an already existing ruling, which is the one in the footnote.

**Redhead** thought this was very dangerous because then it allowed interpretation of other ambiguous groups. The rumen chytrid fungi were originally thought to be animal or protists, and then they were recognized as being chytrids and people could say “Well, traditionally they were not”. The same would be done with other groups of parasitic and microorganisms that turned out to be fungi, and sometimes they were being treated under the *Code* here as fungi. This case dealt with a very explicit exclusion, which was agreed upon by the group dealing with the highly characteristic group of intercellular parasites with a little coiled mechanism that do not look like anything else. Those who work on them have all voted for them to be excluded from this *Code*. They want it to be explicit and not as an example.

**McNeill** clarified that it was not being accepted as a friendly amendment and asked whether it was being proposed formally as an amendment.

**Knapp** wondered if it was an unfriendly amendment. [Laughter.]

**Demoulin** clarified that the amendment was to replace the set of proposals on *Microsporidia* by a second sentence in the footnote to the Preamble that said “For example, *Microsporidia*, having not been traditionally studied by botanists, are not covered by this *Code*. [The **amendment** was **seconded**.]

**Norvell** noted that the *Code* currently said “having never been traditionally studied”, but since Vienna for six years *Microsporidia* had been included in the *Code*. Also the Committee for Fungi originally did support the amendment.

**Prud’homme van Reine** also made this point.

**Malécot** found it strange because it was an inter-*Code* problem. He argued that currently the *Zoological Code* applied “to protistan taxa, when workers treat them as animals for the purposes of nomenclature”. If the decision was made that *Microsporidia* were not governed by the *Botanical Code*, it would mean people working on *Microsporidia* would have the *Zoological Code*, which said that you can use the *Zoological Code* or another *Code*. So there would be maybe some difficulties for these guys to decide which *Code* to use; maybe the bacteriological one.

**Demoulin** interpreted “traditionally” to mean more than six years, a longstanding use should be at least tens of years. He was happy if somebody wanted to amend the proposal by making it clear that tradition was something more than six years.

**Redhead** pointed out that 100 years ago or more the very first microsporidian was described as a yeast.

**Knapp** agreed this was a lovely point of fact and steered the discussion back towards comments or discussion on the amendment.

[The **amendment** was **rejected**.]

To **Harley** it seemed crazy not to include them as plants, or at least as fungi if they are actually such, because the same sort of problem could possibly occur with other groups in the future. He could see that people who had been used to using one sort of *Code* are not going to be very happy about changing, but suggested that this was a temporary problem. If there was no serious technical difficulty in changing from one *Code* to another, he was sure that the Section ought not to exclude them.

**McNeill** commented that the principles of both the *International Code of Botanical Nomenclature* and the *International Code of Zoological Nomenclature* were to keep the content as stable as possible, and neither *Code* considered that phylogenetic information on relationship should be the determinant as to what groups did or did not fall under the *Code*. This meant it was not simply a matter of people getting used to another *Code*, it was an issue of homonymy, which did not apply across the *Codes*. He made the strong point that it is very desirable for stability that the systematic content covered by each *Code* remain the same. This meant that the tradition was the important thing, and what was really under discussion was whether the exclusion of the *Microsporidia* should be explicit in the *Code* or was it implicit, and the recent amendment for implicitness had been defeated.

**Greuter** agreed that the *Code* was a code of nomenclature and, at least in theory, should exclude taxonomy and taxonomic opinion, although this was not always possible as shown by the case of *Microsporidia*. He wondered if it was necessary to state that they were a phylum and suggested taking out the rank designation as a **friendly amendment**. [This was **accepted** by the proposer.]

**Knapp** reiterated that the friendly amendment was to delete the word “phylum” in the proposal.

**Prop. A** was **accepted** as amended.

[Here the record reverts to the normal sequence of events.]

**Prop. B** (76: 3: 34: 1).

**McNeill** introduced Preamble Prop. B, by Gandhi and Reveal making clear that the Appendices were a part of the *Code*. “Names that have been conserved or rejected, oppressed publications, and a glossary of [terms used and] defined in the *Code* are given in the Appendices”. The Rapporteurs commented that it seemed appropriate so long as the wording was corrected to refer to Appendices II–VII [whereas the proposal referred to App. I–VII].

**Barrie** suggested changing “oppressed” to “suppressed”, because it was “oppressa” in Latin but it was “suppressed” in English. The publications were not being politically persecuted.

**Knapp** reiterated that the Section was voting to add an item to the Preamble: “Names that have been conserved or rejected, suppressed publications, and a glossary of terms used and defined in the *Code* are given in Appendices II–VII”.

**Prop. B** was **accepted**.

### Article 1

**Prop. A** (63: 8: 7: 25).

**McNeill** returned the proceedings to the more general portions of the proposals by Cleal & Thomas regarding plant fossils. He drew attention to the very positive support for Art. 1 Prop. A (63: 8) in the mail vote. The Nomenclature Committee for Fossil Plants had considered it together with Prop. B and again gave it strong support, about 80% being in favour.

**Greuter** had been working with fossil plants, not because he knew anything about them, but he knew something about fossil botanists and he had been learning a lot in the process. He outlined that efforts had always been made to understand what those who work with fossils wanted in the *Code* but felt that they did not always know it themselves. He suggested that this had changed given the very complex proposal in question, with large support in the fossil community. He felt there was still a number of difficulties in the proposals starting with Prop. A (i), last sentence: “fossil taxon comprises the remains … preserved in one or more preservational states, as indicated by the description or diagnosis of the taxon”. As a nomenclaturalist he automatically added “original” description or diagnosis, but looking at the Examples later, this was not the case. It was not what was indicated in the original, in the protologue, but the circumscription in terms of organs or whatever given by any worker at any time. Now, if that was what the plant palaeontologists wanted, he felt it should be expressed “depending on the circumscription of the taxon” instead of “indicated by the description or diagnosis of the taxon”. His second point related to “‘organ-taxa’, ‘form-taxa’, ‘autapo-taxa’, or conceptual whole-plant taxa”. Looking at the Examples, he maintained that these were three things and not four, because “autapo-taxa” was the same as conceptual whole-plant taxa. He suggested “autapo-taxa” should be dropped and just left at whole-plant taxa. He felt this was all too complicated for the Section to decide, so his proposal was that the whole Article be referred to the Editorial Committee to implement the necessary changes in close contact with the Committee for Fossil Plants, and hopefully with a representative of palaeobotanists in the Editorial Committee.

**Herendeen** pointed out that there were three proposals, the first two, Prop. A and B, were proposed by Cleal and Thomas, but the last part Greuter talked about was Prop. C, authored by Bateman and Hilton. The Bateman and Hilton proposal received a very negative vote in the mail vote and was not supported by the Committee.

**Greuter** stood corrected and limited his remarks to the first instance and not to the second one. He did not withdraw the proposal to refer these proposals to the Editorial Committee in close collaboration with the fossil plant community, and this was **seconded**.

**Redhead** requested that the Editorial Committee take into consideration any changes that may take place for Art. 59 and adjust appropriately Prop. A (vi), which wished to change Art. 11.1 so that it included “the use of separate names for the form-taxa of fungi is allowed”. He believed mycologists in general did not wish to use form-taxa even with changes in Art. 59.

**Prud’homme van Reine** pointed out an omission in Art. 1 Prop. A: in the emendation of Art. 1.2, “(diatoms excepted)” was left out after the first words, [in the current Art. 1.2] after “Fossil taxa”. He did not understand why this exception was deleted in the proposal by Cleal and Thomas and why the Nomenclature Committee for Algae had not been asked to give its recommendation here. He thought it was clear that the exception had to be kept for at least ten genera of diatoms, some rather large in number of species. He reported that none of the members of the Committee for Algae had voted in favour of the proposal by Cleal and Thomas.

**McNeill** noted this and did not think that it was at all deliberate, he apologized that the nomenclature editor of *Taxon* [himself] did not notice the issue as there was no intention of changing the status of diatoms.

**Herendeen** explained that the core of this proposal was to get rid of the concept of morphotaxa for fossil plants, which had been a very problematic concept for quite a number of years.

**Prop. A** was **accepted** on the understanding that fossil taxa excluded diatoms, and that this would be made clear by the **Editorial Committee**.

**Prop. B** (44: 5: 26: 25), concerning two Examples, was referred to the **Editorial Committee**.

**Prop. C** (17: 45: 12: 31) and **D** (12: 44: 18: 28) were **rejected**.

**McNeill** noted that the Committee for Fossil Plants had considered this and voted unanimously 0 to 11 to oppose Prop. C and D.

### Article 6

**Prop. A** (110: 5: 9: 0).

**McNeill** moved on to a set of proposals by Turland, Vice-rapporteur, dealing with a deficiency highlighted in the preparation of the Glossary.

**Turland** explained that when the Editorial Committee was preparing the Glossary in the *Vienna Code*, it was realized that some of the very familiar terms in the *Code*, such as nomen novum, new combination, new status, replacement name and avowed substitute, were only explained obliquely in the Articles of the *Code* and they were not really defined. The idea of the proposal was to explicitly define the terms in the status definitions in Art. 6, which would also simplify some of the later Articles, for example Art. 33.4, which had one of the oblique definitions in it.

**Alvarado** did not think it was necessary to define all the terms, because he felt that people who work with the *Code* were trained botanists and were already familiar with them. He did not think the *Code* was the place to explain the concepts, but rather to use them.

**Greuter** was much in favour of the definitions. However, he was not happy with one of the terms. He proposed an **amendment** to Art. 6.10 under Prop. A to replace “a nomen novum” by “a substitute name, nom. subst.”. This was firstly because the other terms, new taxa and new combination, were in English and nomen novum was Latin and secondly because the English translation of nomen novum was new name, and past Editorial Committees had successfully eliminated this term because it caused confusion. [The **amendment** was **seconded**.]

**McNeill** noted that this had been alluded to by the Rapporteurs in their comments because it was anomalous to have it in Latin as opposed to English.

**Turland** wished to clarify that Art. 6.9 would read “name of a new taxon”, Art. 6.10 would read “a substitute name” and the abbreviation, in parentheses, would be “nom. subst.” and Art. 6.11 would stay the same, as “new combination”. He wondered if nomen novum would be mentioned at all.

**Knapp** suggested that it should go in the parentheses.

**Gereau** opposed the amendment as he found nom. subst. more confusing and less informative than nomen novum.

**Applequist** noted that there was a great deal of existing literature that used nomen novum and thought it would be very confusing for people who had been brought up on that literature to see the term suddenly disappear.

**McNeill** clarified that there was no suggestion of the phrase disappearing from the *Code*.

**David** wanted to know if nom. subst. was supposed to be a Latin word, or two Latin words. Was it supposed to be “nomen substantivum” or some such? He felt that having nomen novum and nomen substantivum could be more confusing.

**Knapp** suggested that the Section was trying to wordsmith the *Code* as a committee and that this could be left to the Editorial Committee to make sure that the changes made sense.

**McNeill** explained that the amendment was emphasizing the use of “substitute name”, rather than how it should be abbreviated.

**Sennikov** thought that “replacement name” was already a familiar term, and substitute name was something not previously present in the *Code*, and suggested replacement name as the major term to be introduced.

**Greuter** had no strong preference but added that the difficulty was that the Latin abbreviation for nom. subst. existed but did not for “nom. repl.”

**Van Rijckevorsel** liked replacement name better than substitute name, and strongly suggested adding a note that in the literature the term nomen novum was used extensively for the same thing.

**Ford-Werntz** felt that the community understood nomen novum and favoured replacement name over substitute name, giving the argument that a substitute was often temporary while a replacement is something permanent.

**Greuter** accepted this as a **friendly amendment**, leaving open the question of what the Latin abbreviation should be.

**Turland** requested clarification as to whether the amendment from Greuter was to change all mentions of nomen novum throughout the *Code* to “substitute name” or “replacement name”. He wished to know if the convention when publishing nomenclatural novelties would not be to put “nom. nov.” any more but to put something different, possibly “nom. subst.”

**Alvarado** felt that nomen novum had been a tradition, it appeared in a lot of historical documents and it would be better to retain the term. He thought it was better to stick to nom. nov. in Latin rather than changing it into English.

**McNeill** clarified that the proposed amendment did not rule out maintaining nom. nov. as an abbreviation.

**Norvell** suggested it would be better to vote on this in two steps because some wanted to retain nom. nov. First nomen novum and replacement name and second, what Latin term should be used for replacement name.

**Knapp** clarified that the Section was currently voting on an amendment to replace nomen novum with substitute name or replacement name.

**McNeill** responded to Norvell’s concern by suggesting that the Section might want to recommend or decide on the Latin term to be used for the English replacement name.

**Knapp** returned the discussion to voting on the amendment.

**Turland** mentioned that part of his intention with the original proposal was to reduce the three different synonyms used for the same term in the *Code*, which was inconsistent. He recommended against introducing another alternative, so it was not obvious what the main term should be for nomen novum or substitute name.

**Levin** made a point of order that because this was a friendly amendment, the vote would be to replace nomen novum with replacement name, not substitute name.

**Gams** felt that the terms substitute or replacement were confusing, as it could refer either to a name replacing another one, or to the replaced name. Replaced synonym was already commonly used, and for substitute it was the same.

**Sennikov** stressed that nomen novum should be mentioned to avoid potential confusion if it were removed from the *Code*.

**Knapp** proposed moving to a vote on the amendment, to replace “nomen novum” with “replacement name”.

**McNeill** added that this was, as the English language term, the preferred term in the *Code*.

**Bill Barker** requested confirmation that nomen novum would remain in the *Code*.

**McNeill** confirmed that this was the case.

[A show of cards was requested and, as this was close, Ulloa called for an official card vote, which was **seconded**. The **amendment** was **accepted** on a card vote (325: 169; 65.8% in favour).]

**Knapp** tried to move to a vote on the proposal itself.

**Turland** first wanted to propose a slight amendment to the wording of the amended proposal. [Laughter]. He assured the Section that there was no need to panic. After the accepted amendment it stood as “a replacement name (nom. nov., avowed substitute)”. His proposed amendment was to simply put nomen novum inside the parenthesis before *nom. nov.*, so it would read “a replacement name (nomen novum, nom. nov., avowed substitute)”. He reiterated that “replacement name” would be the preferred term used throughout the *Code* but it would not mean the users of the *Code* could not use nomen novum as a term and journals publishing nomenclatural novelties could use nom. nov. as the abbreviation.

**Sennikov** pointed out that the proposal was regarding definitions, and in the third part, on Art. 6.11, there were two terms combined altogether without separate definitions: a new combination or a status novus. It was not clear to him from the definition what this was exactly, and he requested a phrase be added editorially to provide definitions, as a new combination or a status novus were two separate things.

**McNeill** agreed that this was an issue and noted that there was also another point that the Editorial Committee would need to look at, in that, whereas new combination, new name and replacement name all dealt with names, status novus was not actually a name, but a name with a new status.

**Greuter** suggested that the Editorial Committee could clarify this by means of Examples.

**Turland** suggested for Art. 6.11 that, where it read “i.e. new rank”, it could read “i.e. name at new rank”. He added another point that had come up during the coffee break: status novus is Latin. In view of the result of the card vote he proposed we simply change “status novus” to “new status” in English.

**Knapp** confirmed that the Editorial Committee would look quite closely at these issues. She reiterated that Prop. A was to add three new Articles to Art. 6 and adjust the Glossary as appropriate, meaning that the definitions would be in the Glossary as well.

**Prop. A** was **accepted** as amended.

**Prop. B** (106: 2: 8: 0).

**Knapp** introduced Prop. B: in Art. 6 Note 2 to insert “perhaps” before “by different authors” and in the entry for isonym in the Glossary.

**McNeill** added that this was intended to clarify a discrepancy in the definition of isonym.

**Turland** explained that it was to bring the wording in Art. 6 in line with that in Art. 14.

**Prop. B** was **accepted**.

[*A new proposal concerning Art. 6 was included as part of Wiersema’s set of proposals relating to illegitimate family names, and the discussion can be found under the Tenth Session on Friday afternoon.*]

### Article 7

**Prop. A** (103: 5: 8: 0).

**McNeill** introduced Art. 7 Prop. A, which was a simplification of Art. 7.3 and 7.4 that would utilize the terms that had been defined having adopted Art. 6 Prop. A and therefore made the wording simpler.

**Knapp** pointed out that as the Section had approved the amendment of Art. 6, those amended terms would be used in this proposal as well, editorially, so there was no need for further discussion of that nature.

**Turland** explained that Prop. A was not purely editorial because it was making Art. 7.3 parallel with Art. 7.4. One was discussing replacement names and the other one dealt with new combinations or new status, so it was quite logical that they be parallel in their wording. The proposal was to make them more consistent and simpler.

**Prop. A** was **accepted**.

**Prop. B** (15: 23: 73: 0) was referred to the **Editorial Committee**.

**Prop. C** (77: 29: 7: 0).

**McNeill** explained that this proposal was part of a series seeking to clarify the nomenclatural status of names of which there was more than one potential descriptive statement. It would simply make clear what most people had assumed, that a name validly published solely by reference to a previous effective publication was to be typified by an element from that publication. In other words, if there was a validating description in the protologue, there was no need to go back to any earlier ones. He drew attention to the positive response in the mail vote of 77 in favour and 29 against.

**Gereau** felt that the wording of the proposal was particularly unfortunate, claiming that the “solely” was completely unnecessary in this context. He maintained that if a name was published by reference to a previously and effectively published description or diagnosis, that was what it was published with a reference to. He also argued that “from the context of the validating description or diagnosis” was an exceedingly vague phrase that was possibly misleading.

**Demoulin** agreed. He did not understand the need for the proposal and opposed it.

**Soreng** felt the proposal needlessly restricted the elements that could be included in the typification, which he felt should be left up to the typifying authors to make a critical decision.

**McNeill** again explained that the proposal was intended to state what most people had assumed, that the Article concerned had always been interpreted as dealing with a situation in which a name was validated solely by a previously published description and at the place of valid publication there was no description. He maintained that “solely” was needed. He clarified that you would only apply Art. 7.7 when there was no description in the place of valid publication. That was not assumed by everyone, and it was possible to interpret the present wording as though you could go to any description. If the wording of the proposal was defective then the Editorial Committee would deal with that.

**Govaerts** wanted to know if the proposal would be retroactive, as it could have consequences for previous lectotypifications.

**Barrie** emphasized that the only difference between what was in the *Code* and what was being suggested was the word “solely”. If a name had been properly lectotypified he did not think this proposal should destabilize the type.

**Prop. C** was **accepted**.

**Prop. D** (16: 82: 11: 1).

**McNeill** pointed out that Prop. D was overwhelmingly defeated in the mail vote.

**Malécot** clarified that there was actually 74.55% who voted no in the mail vote, not 75%.

**McNeill** confirmed that the no vote did not quite reach 75%. The proposal was to change the phrase “context of” for material to “material associated with”, in terms of the type in Art. 7.7.

**Prop. D** was **rejected**.

**Prop. E** (65: 28: 17: 0).

**McNeill** noted that the Example associated with the proposal was very bizarre but the suggested addition was not unreasonable. He drew attention to the fact that it received substantive support in the mail vote of 65 in favour and 28 against.

**Greuter** warned that the proposal was a typical example of a proposed wording that, if adopted, could have quite negative sideeffects that were not necessarily obvious. He argued that Art. 7.7 only made sense when limited to names of a new taxon. The crossreference to Art. 32.1(d) meant that it also applied to new combinations and nomina nova that were not solely validated by reference back. This was because the typification was not relevant at that stage, but this became relevant for nomina nova validated only, as the case usually was, by an element from the original context. He suggested as a friendly amendment that the words “of a new taxon” be added after “a name” and before the newly inserted “solely”. [The **friendly amendment** was **accepted**.] He went on to deal with the second portion that appeared in bold in the proposal “or explicitly excluded part of the material associated” etc., arguing that this should not, in his opinion, apply to names above the rank of species because it would leave us with circumscription method procedure, which had been banished from the *Code* presumably since the type concept was introduced. His specific suggestion was, if the second boldface passage was deemed desirable, which he did not object to on principle, then it should be preceded by the specification “for names of a species or infraspecific taxon”.

**Prud’homme van Reine** commented that the Nomenclature Committee for Algae was unclear as to how to read the last sentence of the proposal, specifically “the indication or descriptive and other matter”, should it be “indication of descriptive”?

**McNeill** refocused the discussion on the proposal, as he felt Prud’homme van Reine had raised a general question on the wording of Art. 7.7 in the *Code*.

**Sennikov** returned to the issue Greuter raised concerning taxa above the rank of species, claiming that this exclusion was already effected by the presence of brackets “(but see Art. 10.2)” and felt no extra reference was needed.

**McNeill** asked if Sennikov felt that that excluded generic names.

**Sennikov** felt there was a hint in the brackets.

**Greuter** supposed that the Editorial Committee could clarify it as it deemed fit.

**Knapp** called for a vote on Prop. E on Art. 7.7 to amend it to “A name of a new taxon validly published”, to insert the word “entire” before “context” and to insert the words “or explicitly excluded part of the material associated with the validating description or diagnosis” after the word “type” but before “(but see Art. 10.2)”.

**Prop. E** was **accepted** as amended.

**Prop. F** (12: 33: 63: 0) was referred to the **Editorial Committee**.

**Prop. G** (80: 3: 29: 0).

**McNeill** introduced Prop. G as a modification to an existing Example in the *Code* and noted that it had received overwhelming support in the mail vote, with 80 in favour and two against. The Rapporteurs commented that the correction of a long-standing Example in the *Code* was a useful clarification.

**Veldkamp** introduced himself as a botanist in the Malesian area who had had a lot to do with the *Herbarium Amboinense* of Rumphius. It was a custom in the 19th and 20th centuries to base new names on collections that people attempted to identify with the *Herbarium Amboinense*. He noted that *Adenanthera bicolor* was from Sri Lanka and the plant described by Rumphius was from Ambon, so he felt it was rather dangerous to take the picture and the description of the *Herbarium Amboinense* species as the type. He advised against doing this. There was a specimen at Kew collected by Moon and labelled by him as such. This was an attempt to identify his specimen, and it was very likely that it was a different species to what Rumphius had in Ambon. Therefore he even suggested scratching the whole Example and certainly not accepting the proposal.

**Perry** outlined her reason for suggesting changing the Example was because many people were attempting to interpret it as meaning that the illustration was the type because it was cited by Moon, not that it was part of the validating description. She had not changed anything, just turned it round a bit to clarify. She noted that the Example had been in the *Code* since 1981.

**Barrie** suggested referring the issue to the Editorial Committee.

**Knapp** concluded that Barrie was getting tired.

**Reveal** very politely addressed “Madam Chairman” and suggested that the whole matter be addressed to the Editorial Committee with a purpose of resolving, with the help of others, the taxonomic status of the name and whether the Example should be accepted, modified or deleted.

**Prop. G** was referred to the **Editorial Committee**.

**Prop. H** (13: 74: 7: 8), **I** (38: 49: 6: 7) and **J** (16: 61: 15: 6) were all **withdrawn**.

[*These and others were replaced with a new set of proposals dealing with “sanctiotypification”. All of the relevant discussion can be found under the Tenth Session on Friday afternoon, including discussion begun during the Sixth Session on Wednesday afternoon.*]

**Prop. K** (6: 14: *86: 2).

**Turland** outlined that there was a slight problem with the proposal and read from the Rapporteurs’ comments: “However, the proposal stems from a slight misconception that was unfortunately reinforced in the editorial process. Article 9 (along with Art. 8) deals only with typification of names of species and infraspecific taxa, whereas Art. 7 deals with typification in general and so Art. 7.11 applies also to all typification including that of names of genera and subdivisions of genera. The doubt that the proposers perceived as to the requirements for typification on or after 1 January 2001 could be resolved by the addition of a parenthetical ‘(see also Art. 7.11)’ at the end of Art. 9.21. An ‘ed.c.’ vote will be so interpreted.”

**Prop. K** was referred to the **Editorial Committee**.

[*The following debate, pertaining to Art. 7 Prop. L, took place during the Seventh Session on Thursday morning, with discussion on Art. 37bis Prop. A.*]

**Prop. L** (39: 40: 12: 8).

**McNeill** introduced Art. 7 Prop. L as an additional proposal stimulated by the proposals dealing with the requirement to register fungal names prior to their being considered to be validly published. The proposal required the publication of fungal nomenclatural acts, such as lectotypification, to be recorded in a recognized repository and dealt with what was required for type designation to be effective. This was considered by the Committee for Fungi, which supported it 71% in favour (10: 3: 1).

**Gams** outlined that the proposal was strictly connected with Art. 37*bis* Prop. A, where the deposition and the registration of new names were made compulsory for validity. In this proposal the same requirement for validity was also postulated for all actions of typification. In another proposal this would only be a Recommendation. It was recommended that these typification actions be registered in MycoBank or a similar organization.

**Wiersema** relayed the fact that his mycological colleagues [at USDA] did not express support for the proposal.

**Kirk** pointed out that in order to be compatible with the proposal that had just passed [Rec. 37*bis* A.1 during the Seventh Session on Thursday morning] it should be “identifier” rather than “record number”.

**Gandhi** also opposed the proposal, preferring to see the type information cited within the protologue.

**Barrie** wondered if requiring more than the name to be registered might cause problems. The type already had to be attached to the name, so if the name was linked through the registration number he questioned whether it was desirable to add the extra requirement of having the type listed, because essentially that meant registering the type as well. Would the name then not be validly published if there was a glitch and the type did not get designated?

[One of the Microphone Runners had the temerity to suggest that someone had asked a question that the President had missed.]

**Knapp** set them straight, that it was she who got to choose who spoke, adding that no-one realized how much power she actually had. [Laughter].

**Sennikov** commented that the types were not automatically attached to the plant names in such a way that, should a plant name be published, you could easily find a type. He suggested that this was quite obvious for old names, and it was a real mess to find the first instance of lectotypification for many names, and many people had spent a lot of time doing so. There were many competing choices of lectotype selection, according to him, and it may well happen for any name in the future that it could appear to be lectotypified in some obscure source, and it was exactly the same issue as for valid publication of plant names. He felt that typifications, lectotypifications, neotypifications etc. should be registered and made available to the public in the same way as new plant names. He argued that it was exactly the same issue, as it was also a nomenclatural act; it must be registered and not be placed into the area of grey literature.

**Norvell** noted that this was supported by a 71% vote of the Committee for Fungi.

**Demoulin** agreed with Barrie that there were already enough requirements for valid publication and it added a risk of more names being turned invalid…

**McNeill** interrupted that it was not new names under discussion. This was regarding registration of a nomenclatural act. All it meant was that a lectotypification was not effected unless it was registered.

**Demoulin** continued that he believed there were people who would use the fact that the type was not registered and then the name would be considered invalid.

**McNeill** explained that this was in Art. 7, which was dealing not with valid publication, but with the effectiveness of a nomenclatural act—that is, a lectotypification or a neotypification. If a lectotypification was not registered then somebody else could come along and do it later. So this would not affect the valid publication of names.

**Demoulin** demurred.

**Prop. L** was **rejected** on a card vote (256: 187; 57.8% in favour).

[Here the record reverts to the normal sequence of events.]

**Prop. M** (46: 6: 58: 0) was **withdrawn**.

### Article 8

**Prop. A** (79: 17: 15: 0).

**McNeill** noted that Art. 8 Prop. A had fairly positive votes, 79 to 17, and had been proposed in association with other Articles to clarify that an illustration included in the protologue was part of the original material. It was an attempt to clarify what was meant, in botanical terms, by an illustration.

**Barrie** was concerned that the proposal seemed to indicate that photographs of a type specimen that accompanied a protologue would now be considered type material themselves. For example, a photograph of a holotype published with a protologue, whereas currently these are not considered type material.

**McNeill** felt that the issue of whether they were considered type material was dependent on the other linked proposal, Art. 9 Prop. E. He pointed out that some people would say that any illustration in the protologue was original material, but the literal wording of the current Note on that excluded them as being original material, which was what Prop. 9 was concerned with.

**Prud’homme van Reine** spoke on behalf of phycologists and hoped that the proposal would not be accepted because they were afraid that something would happen with all the old pictures used in phycology. He suggested inclusion of the text “all pictures recorded in the protologue are considered to be illustrations and will belong to the original material”. The reaction by the Rapporteurs, that the proposal was useful because it ruled out habitat photographs and the like was felt to be threatening. He reported that phycologists were particularly afraid of the restrictions about the use of illustrations because of Art. 39, which stated that validly published names of non-fossil algae must be accompanied by an illustration from 1 January 1955. If the definition of an illustration was to be changed now, he argued, this could lead to difficulties in older descriptions and typifications in algae.

**McNeill** asked Prud’homme van Reine if he considered that there were illustrations that would be the validating illustration for an algal new taxon that would be excluded by this definition.

**Prud’homme van Reine** confirmed that he thought this could happen quite often, especially for plankton, where photographs could include a number of things with only one of them pinpointed as the type. He also referred to the many newer underwater photographs of growths of algae that were more or less habitat photographs, but for phycologists they depicted the type of the alga.

**Turland** requested clarification as to whether Prud’homme van Reine was suggesting that such photographs would not depict a feature or features of the new taxon being described.

**Prud’homme van Reine** replied in the negative [presumably against the suggestion]. He reiterated that he felt the comments of the Rapporteurs were dangerous regarding ruling out habitat photographs and the like, as the definition of “the like” was not clear.

**McNeill** replied that in considering a proposal to amend the *Code*, the Rapporteurs do their best to interpret its intent, but the Rapporteurs’ comments do not appear in the *Code*. What appears in the *Code* is the wording of the proposal. He felt it would be surprising if an illustration that was considered a validation of a name of an algae did not depict a feature or features of the new taxon described, as that was the criterion [of a validating illustration]. When he mentioned a habitat photograph, he was thinking of vascular plants, where you might have a view of a meadow with absolutely no feature of an individual species visible. But presumably in no case would the illustrations in question not include a feature or features of the new taxon described. He wondered if names of algal taxa were really validated by a photograph of green scum, or something. [Laughter].

**Knapp** clarified for Prud’homme van Reine that the Rapporteur meant that he would find it very unusual if one would validate a new taxon on a photograph that did not show a feature of the plant that was being described.

**McNeill** added that the suggested change in wording would not affect these names.

**Prud’homme van Reine** reiterated that in his Committee everybody was very afraid that it would, because it was sometimes just one small dot or so [on a photograph] that was important, and specialists understood that, but others quite often did not and then they would say there were no data on the photograph. He preferred to be able to make the choice and not have the *Code* already do so in this respect.

**Sennikov** explained that which illustrations were acceptable or not for typification was defined in the definition of original material in Note 2 under Art. 9.2, so in principle the proposal was not so dangerous as it looked. He felt that it was highly desirable either to have explicit reference to the definition of original material in the way proposed in the proposal or these definitions should preferably be explicitly referred to each other or combined somehow so that they could not contradict each other. Therefore he suggested that the definition of original material should be taken in consideration while discussing this proposal.

**Buck** thought that part of the confusion was habit photo versus habitat photo and if that distinction was understood then there should not be an issue.

**Nic Lughadha** was concerned that, by the definition “work of art or a photograph”, this may inadvertently exclude some types of illustration, such as a nature print, which she would not consider a work of art because it was mechanical, but it was also not a photograph.

**Van Rijckevorsel** suggested adding the word “diagnostic” before “features” so as to emphasize that the photo should show something diagnostic.

**McNeill** felt that the *Code* did not require descriptions to be diagnostic and that that should be the same for an illustration, it just required that it show the organism. It should be diagnostic from a point of view of good taxonomy but he was not certain that it was required in the wording of Art. 37.

**May** explained that it was the lack of the word “diagnostic” that protected the concerns of the Committee for Algae, as that allowed leeway so that if the illustration was a bit vague and fuzzy, but at the time considered adequate, it was not possible to retrospectively decide there was nothing diagnostic about it.

**Greuter** encouraged the Committee for Algae and others to submit examples to the Editorial Committee so that they could illustrate what was and was not an illustration in definite cases. He felt that the wording was fine but not clear enough, citing the example that the wording would encompass, without any doubt, the photograph of metaphasic fig because it was a feature, not a morphological feature but a feature and this would be useful to have as an example.

**Annette Wilson** was concerned about the wording “work of art” because she did not find it an adequate description of a very broad range of illustrative material. It seemed to her to encompass anything that was not a photograph, some of which were very obviously not artistic. She pointed out that there were some awfully bad drawings out there.

**Alvarado** suggested replacing “work of art” with something like “image”, because images can be of any kind and not just artistic.

**McNeill** suggested this was like saying that “Here and elsewhere in the *Code* an illustration is an illustration, featuring a…”.

**Knapp** warned that the discussion was erring on the side of wordsmithing the *Code* again and alerted the Section to the fact that this could take rather a lot of time.

**Marhold** suggested, instead of “work of art”, “drawing” might be better.

**McNeill** added that it could also be a painting. He felt that if the proposal were to be approved the Editorial Committee would ensure that what had been expressed was covered in terms of making sure that nothing fell between the gaps in the set of definitions without changing the intent or the meaning.

**Prop. A** was **accepted**.

**Prop. B** (17: 44: 16: 23).

**McNeill** introduced the proposal, which dealt with problems that existed with microfossils in that the material was very often on a slide and unlocatable. The author was trying to find some way in which an illustration could originally be the type and suggested in the proposal that it would be a surrogate for the type. The proposal received a negative vote: 17 in favour and 44 against and a substantial number (23) wanted to send it to a Special Committee. This was considered by the Committee for Fossil Plants, which again was negative but not to the same degree as in some other cases. It was split at four in favour, six against, so a 55% “no” vote. The Rapporteurs wondered if the proposal was a little convoluted and if there was an option in this situation of invoking what was currently in the *Code* for algae and fungi for which there were technical difficulties of preservation, i.e. Art. 37.5, which was one of the situations in which an illustration could be the type.

**Herendeen** commented on behalf of the Committee for Fossil Plants, which was against the proposal, that there were a number of problems with fossil plants. Illustrations were not allowed to serve in the place of a specimen as type. Traverse, the author of the proposal, proposed the idea of a surrogate for the type if the specimen was no longer able to be found or was degraded, but introducing the new concept of epitype was found to be very problematic. The epitype would only work as long as the type itself existed, so an epitype would not work for this problem. The Committee felt that the proposal addressed a real problem but the suggested solution was something that the Committee did not favour.

**McNeill** wondered if the Committee had considered the Rapporteurs’ suggestion of extending Art. 37.5 to cover this situation. That related to technical difficulties of preservation, where it was impossible to preserve a specimen for recent microalgae and microfungi and it was possible to have an illustration as type. He noted that it was the only situation in the *Code* in which an illustration was allowed as type [of the name of a new taxon published currently].

**Herendeen** responded that the Committee had discussed that and it was not approved. They did not want to open the door to illustrations serving in place of a specimen, even if that specimen was impossible to locate.

**Prop. B** was **rejected**.

**Prop. C** (20: 84: 8: 0) was ruled as **rejected**.

[*The following discussion, pertaining to a new proposal by Prud’homme van Reine concerning Art. 8.4, took place during the Tenth Session on Friday afternoon.*]

**Prud’homme van Reine’s proposal**

**McNeill** introduced a new proposal on Art. 8.4 by Prud’homme van Reine regarding type specimens of names of taxa being preserved permanently. [The **proposal** was **seconded** and **supported** by three others.]

**Prud’homme van Reine** explained that the proposal was to make explicit what was already implicit by adding: “to remain alive in the inactive state”. He added that it was easy to kill a culture by deep-freezing but that was not what was desired, it was necessary to be able to work with the cultures later and they needed to be living cultures.

**Alvarado** thought this was a good amendment and noted that in the Prokaryotic *Code* it was essential to keep a sample of a living organism in a state as it was stated there. He felt it was very important to also include that for *Cyanobacteria* and agreed that the proposal made it explicit.

**Reveal**, having a little knowledge of history, called the attention of the Section to the herbarium in Berlin and several other herbaria in Europe during the Second World War in the 1940s. He agreed that what was being mandated was perfectly reasonable, providing electricity worked all the time and everything was done perfectly over an extremely long period, because if something was destroyed, for example 50 years from now due to war or accident, then there would not be any types. He thought it was necessary to put something into Art. 8.4 that took into account that an inadvertent accident would not invalidate the type. He did not know how to deal with the problems regarding an unintentional accident.

**Prud’homme van Reine** responded that if the culture was lost then there was no type anymore. [Audience muttering.]

**Unknown speaker** noted that if herbarium specimens were lost, there was no type any more either.

**May** interpreted the situation that if the culture died it was just then like a specimen.

**Hawksworth** pointed out that if they were lyophilized they were not dependent at all on electricity. He explained that these were little vacuumsealed tubes that could be put in herbarium packets in a herbarium, so there was no basic difference at all. He added that it was only the deepfrozen ones that could encounter the issue, but in practice what people did with cultures like this was send duplicates, isotypes, to a number of different collections and normal practice was to send them to at least three.

**Barrie** wondered if the suggested wording was really any different to what was already in the *Code* and was just adding something else in. It seemed to him to repeat what “metabolically inactive state” meant, because that implied “you can be metabolically inactive and alive, you can be metabolically inactive and dead, but…” [Laughter.] He concluded that it did imply that these were alive.

**Boyne** was wondering if it was desirable to keep the cultures alive in an inactive state so that they could be revived and studied later. Would there be a limit as to how much of that inactive culture could be used, because if a person decided to take the entire culture and revive it, would that no longer be considered the type? Or do they have to take a sample of the revived culture and then refreeze it, for example, and then would that be considered the same type?

**Demoulin** noted that there had been extensive discussion on the problem of a culture as type during preceding Congresses and he did not think it was appropriate at the end of this one to reopen the general discussion. He felt this was a minor amendment that may be considered slightly superfluous but did no harm. His opinion was that the amendment should be accepted and the general discussion should stop, despite it being certainly very important, and he felt it was not the intention of the proposer to open it again.

**Kirk** felt there was ambiguity here as to what were the consequences of this live culture when it died. He interpreted the current *Code* that however many bits of the type were preserved in the metabolically inactive way, when one was revived and it became living, it was no longer the type because the *Code* specifically said the type could not be living.

**Herendeen** called the question. [There was a sufficient majority in favour of voting.]

**Knapp** moved to a vote on the addition of the words “to remain alive in that inactive state” to Art. 8.4. She reported that the count was 46 for and 33 against, which did not reach the 60% supermajority rule for inclusion in the *Code*, and so deemed that the proposal was rejected.

**Prud’homme van Reine** called for a card vote. [Audience groaned.] He apologized.

**McNeill** asked if his Committee really thought it was that important.

**Prud’homme van Reine** confirmed it was.

**Knapp** reported that the results of the card vote, to include the phrase “to remain alive in that inactive state”, was 290 “yes” and 145 “no”, i.e. 66.67% “yes”, so the proposal to include that phrase had been passed.

**Prud’homme van Reine’s proposal** was **accepted** on a card vote (290: 145; 66.7%).

[*Here the record reverts to the normal sequence of events.*]

### Recommendation 8A

**Prop. A** (38: 52: 18: 1).

**McNeill** explained that Rec. 8A Prop. A sought to redefine the definition of protologue, so as to make explicit that material that was not effectively published was excluded. That would include electronic supplements to hardcopy papers. He added that it received somewhat negative responses, 38 in favour and 52 against.

**Van Rijckevorsel** confirmed that the proposal was intended to explicitly exclude electronically published material. He wondered if perhaps a more explicit wording would be more popular, something to the effect that prior to a certain date electronic supplements were not part of the protologue.

**Barrie** was concerned that putting “effectively published” was going to threaten things like uncited illustrations used for type material and uncited syntypes. Introducing this concept “as far as effectively published” could cause problems for the typification of some names.

**Redhead** agreed with Barrie in worrying about effective publication of these illustrations when he considered that in the past illustrations that had been deposited in herbaria but were not effectively published may have been cited and could be used as types.

**McNeill** did not think that would be affected by the proposal because it was the citation of the illustration or the citation of the herbarium specimen in the protologue.

**Greuter** noted that in Art. 9, the type was given as an element selected from the original material. The type could be an unpublished illustration so he argued that the proposal would introduce conflict between definition of a type and definition of original material if it were passed.

**McNeill** felt that this was the point that Barrie raised.

**Redhead** remained worried about the issue, despite the comments from the front.

**Prop. A** was **rejected**.

**Prop. B** (8: 82: 14: 2) was ruled as **rejected**.

**Prop. C** (8: 45: 52: 0) was ruled as **rejected** as it was an editorial change related to Art. 32 Prop. I, which was rejected.

**Prop. D** (34: 44: 30: 0).

**McNeill** outlined that Prop. D recommended greater precision in the designation of type.

**Barrie** relayed comments from the collections manager at the Field Museum Herbarium, Christine Niezgoda, on the proposal: many herbaria do not have barcodes or accession numbers on their specimens, so suggesting putting these on may be a little bit too restrictive.

**Alvarado** commented that sometimes a description was written while the material being described had not yet been processed and the numbers of the specimen had not yet been assigned. He felt that it was a bit of a problem adding an accession number before or at the time of description because sometimes herbaria work very slowly.

**Thiele** responded by mentioning the experience that many herbaria probably shared where an unmounted specimen was not yet processed into the collection but used as type material. He felt this was a very dangerous thing to do as there were many occasions where the material subsequently got lost.

**Knapp** felt that this was a very interesting point but that it was not germane to the proposal.

**Kellermann** wondered whether this had to be in the *Code* or whether it was more a matter of a best practice of how to publish taxa and how to best write descriptions and cite material.

**Kirk** interjected that that was what the Recommendations were for.

**Knapp** forbade talking without the microphone and threatened to send Kirk into the time-out corner.

**Prop. D** was **rejected**.

### Recommendation 8B

**Prop. A** (88: 0: 11: 7).

**McNeill** introduced Rec. 8B Prop. A, a proposal regarding a culture being designated as a type. He noted that cultures that were permanently preserved were permitted to be designated as types and the Recommendation was that the status of the culture should be indicated, including the phrase “permanently preserved in a metabolically inactive state” or an equivalent. He added that the proposal affected algae and fungi and received substantial support in the mail vote with no negative votes at all and was supported both by the Committee for Algae and the Committee for Fungi by about 80% majorities in favour in both cases.

**Prud’homme van Reine** was in favour of this Recommendation but the Committee for Algae proposed an amendment to replace “designated” with “intended to serve” and to add “under Art. 8.4” after “as a type”. The reason given was that taxonomists may forget to write down that material was permanently preserved in a metabolically inactive state or they may do it incorrectly so the material was not really metabolically inactive despite intending it to be type material. The amendment would clearly add to the Recommendation that the type was according to Art. 8.4 and therefore “must be preserved permanently and may not be living plants or cultures. However, cultures of fungi…”

**Buck** spoke strongly against the amendment arguing that intent is not the same as designation, and it may not even have been mentioned in the publication. He recommended overwhelmingly turning down this Recommendation because it was not possible to know what somebody intended to do if they did not publish it.

[The **amendment** was **rejected**.]

**Prud’homme van Reine** relayed a comment from Robert Anderson, a member of the Nomenclature Committee for Algae, who had been involved for 15 years in the study of algal cultures in the Provasoli-Guillard National Center of Culture of Marine Phytoplankton, a well-known institute in the United States of America, that the phrase “metabolically inactive state” was not precise.

**Knapp** highlighted that the proposal said “or equivalent”.

**Prud’homme van Reine** continued that he [Robert Anderson] assumed that the phrase intended that a metabolic inactive culture could be made metabolically active again. He reported that this was only possible by cryopreservation and lyophilization, and the latter was not used for algae because there was incredible loss of viability of 5 to 20% per year. However, cryopreservation only worked at temperatures below the glass transformation temperature, which was approximately minus 135 degrees centigrade. At temperatures warmer than minus 135 degrees centigrade ice crystals continued to expand and contract, cutting up cellular material including DNA. If the intent of Art. 8.4 was that organisms remain alive in an inactive state then it should say this, and thus he returned to his earlier proposal…

**McNeill** pointed out that this was a totally new proposal that had nothing to do with the proposal under discussion, except that both apply to “permanently preserved”. He suggested it should be deferred to the end of the sessions with other business and Prud’homme van Reine concurred.

[*The discussion can be found after Art. 8 Prop. C.*]

**Prop. A** was **accepted**.

### Article 9

**Prop. A** (25: 38: 43: 0).

**McNeill** explained that Art. 9 had a series of proposals, Prop. A to D, designed to address what was perceived as a conflict between Art. 9.1 and 9.2. The proposals received mixed support, all were to a degree negative, some very substantially so.

**Prado** spoke on behalf of the proposers, who had detected a conflict between Art. 9.1 and 9.2, because Art. 9.1 mentioned the material “used by the author, or designated by the author” as a holotype, whereas in Art. 9.2 this phrase disappeared and the Article mentioned only a holotype being “indicated”. The intention was to make the two Articles parallel. He did not feel that the suggestion would change the meaning or definition of the holotype.

**McNeill** clarified that Prop. C particularly addressed that issue, as opposed to Prop. A and B, which were more generally editorial. Regarding Prop. C, the issue seemed to be how broad the word “indication” was considered to be. A holotype was defined as a specimen, the one specimen used or designated by the original author. He found it hard to see how to know that a specimen was a type if there was no such indication.

**Buck** contributed some hearsay evidence that, after having talked to Moran [senior author of the proposal] about the issue, when it came to an institutional decision about how to vote, he himself voted against these. [Laughter].

**Gereau** agreed with the Rapporteur-général that Prop. A and B attempted somewhat clumsily to do the same thing that Prop. C actually accomplished. He did not see any further clarity in Prop. A and found the phrase in the Note “and its duplicates (if any) accepted as isotypes” particularly unclear in its definition and application. He thought the consistency brought about by Prop. C was very useful and should be accepted, but that Prop. A and B should be rejected.

**Prop. A** was **rejected**.

**Prop. B** (11: 30: 65: 0) was referred to the **Editorial Committee**.

**Prop. C** (33: 50: 27: 0) was **rejected**.

**Prop. D** (4: 33: 67: 0) was referred to the **Editorial Committee**.

**Prop. E** (80: 20: 4: 0).

**McNeill** introduced Art. 9 Prop. E, which returned to the definition of illustration discussed earlier on. The proposal addressed the question of whether an illustration published as part of the protologue was original material. This had been assumed by many people to be the case, but according to the definition of original material—“those specimens and illustrations (both unpublished and published [either prior to or together with the protologue]) upon which it can be shown that the description or diagnosis validating the name was based”—it was only an illustration that was used by the author that could be original material. Very often both the description or diagnosis and the illustration were produced based on the same plants, in which case this definition would not apply.

**Gereau** emphasized that he felt this proposal was very dangerous. He felt that illustrations known not to have been used by the author being candidates for typification was contrary to all definitions of original material.

**McNeill** wondered if Prud’homme van Reine wished to comment, as he knew that the Committee for Algae was very upset at any suggestion that illustrations should not be original material.

**Prud’homme van Reine** did not have a problem with this proposal.

**Prop. E** was **rejected** on a card vote (222: 246; 47.4%).

**McNeill** commented that this meant essentially that the situation still existed where it was very ambiguous if an illustration associated with the protologue was part of the original material, unless it was possible to establish that the author used the illustration, which he pointed out was a rare event.

**Prop. F** (29: 17: 54: 2) was referred to the **Editorial Committee**.

**Prop. G** (45: 29: 26: 1) was referred to the **Editorial Committee**.

**Prop. H** (37: 33: 22: 12).

[*The following debate, pertaining to Art. 9 Prop. H, took place during the Ninth Session on Friday morning.*]

**Knapp** suggested the proposal could be sent to the Editorial Committee.

**Redhead** requested clarification whether this was intended to mean just the illustrations, the specimens that the validating author had and not anything in any earlier synonymy or cited publications.

**Perry** clarified that it was only meant to reflect what was already in Art. 7.7, second sentence.

**McNeill** suggested it would seem to imply what is actually available to the validating author or indicated by them. He added that these were names relative to later startingdate works. The proposal had gone to the Committee for Fossil Plants for their comments and they supported it.

**Demoulin** thought it would be very bad if this passed because he felt it was premature and dangerous as the later starting-point for blue-green algae was not yet decided. He felt it was important to maintain the link between original author and revalidating author or sanctioned author in the mycological system.

**McNeill** responded that it was a Note, so if it did make a change to Art. 7.7, then the Editorial Committee could clearly modify the wording to make sure it did not depart from the intent of Art. 7.7. He added that the Committee for *Bryophyta*, the Committee for Fossil Plants and the Committee for Algae all voted in favour, respectively 91%, 79% and 87%.

**Demoulin** was referring to the blue-green algae, not the fossils or the bryophytes. He felt that the *Code* should not insist on this definition of original material.

**Perry** clarified that, as in Art. 9 Note 2, it would only apply to “normal names”, not names typified under Art. 7.7 or 7.8. The aim was to try to rectify an oversight.

**Gereau** felt that a “yes” or “no” vote was needed. A “yes” would mean the Editorial Committee would see it anyway, but he thought clear indication from the Section of its will on this issue was necessary.

**Prop. H** was **accepted**.

[*Here the record reverts to the normal sequence of events.*]

**Prop. I** (33: 54: 7: 5), **J** (31: 48: 10: 7), **K** (30: 42: 19: 6), **L** (29: 41: 21: 4) and **M** (30: 42: 18: 7) were all **withdrawn**.

**Prop. N** (10: 38: 56: 3) was ruled as **rejected** as it was an editorial change related to Art. 32 Prop. I, which was rejected.

**Prop. O** (93: 6: 7: 0) was **accepted**.

**Prop. P** (92: 4: 12: 0) was **accepted**.

**Prop. Q** (78: 9: 23: 0).

**Barrie** had a problem with the proposal regarding people who published names with long lists of specimens where they were explicitly citing some as paratypes and saying others were not. The proposal would mean that all material would be paratypes.

**McNeill** agreed that this would mean it was not possible to exclude material.

**Barrie** was concerned that this may restrict people’s freedom of choice in the way material could be written up in protologues and descriptions.

**McNeill** thought that was open to question. The present wording permitted a specimen to be cited in the protologue that was neither a holotype nor an isotype and that was not stated to be a paratype, but the *Code* made it explicit that nevertheless it was a paratype.

**Prop. Q** was **accepted**.

**Prop. R** (6: 51: 50: 0).

**McNeill** noted that proposal R was rather misleading, but the Rapporteurs had commented that the Examples given would be quite useful to be included. That led in the mail vote to a very substantial vote against and a similar vote to refer it to the Editorial Committee. The Note would only be accurate if there was no autonymic infraspecific taxon recognized. Whereas that was quite common in earlier publications, it was much less so later on and this was not realized by the proposer.

**Gereau** found the proposal to be exceedingly dangerous and potentially destabilizing as it was quite contrary to the current understanding of what a syntype was. To say that all specimens cited under all infraspecific taxa were syntypes of the species being described was felt to be very bad practice and should be completely rejected.

**Veldkamp** thought that there was a confusion between taxonomy and nomenclature. It was clearly against the intention of the original author, who distinguished between a typical infraspecific taxon and atypical ones. Therefore, the proposal should not be sent to the Editorial Committee; it should be rejected.

**Sennikov** found the proposal to be absolutely erroneous because it is always assumed that it was a typical variety of a species name that was typified and named varieties that were included under that species name had separate types. Those syntypes that were included under varieties were part of the original material of those varieties, not of the typical variety, which was established by valid publication of those named varieties.

**Greuter** suggested that all Examples suggested for addition, deletion or amendment, except voted Examples, be referred systematically to the Editorial Committee, who were free to use them or not. The Editorial Committee vote was just that the Editorial Committee should take into account the Examples in the proposal and the comments on the proposal were negative.

**Prop. R** was **rejected**.

**Prop. S** (6: 37: *63: 0) was ruled as referred to the **Editorial Committee**.

**Prop. T** (49: 20: 37: 0).

**Van Rijckevorsel** pointed out that this could have been handled editorially and he had pointed it out before the meeting of the Editorial Committee of the Vienna Congress, but it had not been not corrected. So he had taken the opportunity to rewrite it, to do justice to the case.

**Prop. T** was ruled as referred to the **Editorial Committee**.

**McNeill** clarified that the only case where the Section would have to actually vote something to the Editorial Committee was a voted Example, other than that it was undesirable to formally vote that something should go to the Editorial Committee because all Examples were automatically considered by the Committee and they had no obligation to either include or exclude, unless it pertained to a voted Example.

**Prop. U** (28: 38: 21: 18) was ruled as **rejected** as it was a corollary to Art. 8 Prop. B, which was rejected.

**Prop. V** (74: 6: 11: 7) and **W** (73: 6: 13: 7) were **withdrawn**.

[*The discussion preceding the withdrawal of Prop. V is located with the set of proposals relating to fungi with a pleomorphic life cycle in the Eighth Session on Thursday afternoon.*]

**Prop. X** (19: 8: 77: 0) was referred to the **Editorial Committee**.

**Prop. Y** (10: 8: 88: 0) was ruled as referred to the **Editorial Committee**.

**Prop. Z** (12: 85: 10: 0), **AA** (21: 84: 3: 0), **BB** (18: 88: 4: 0), **CC** (17: 86: 6: 0), **DD** (15: 92: 6: 0) and **EE** (7: 104: 3: 0) were all ruled as **rejected**.

**Prop. FF** (36: 5: *71: 0).

**McNeill** introduced Prop. FF, which was supported reasonably strongly in the mail vote, 36 to 5, with 71 wanting it to go to the Editorial Committee. It regarded inserting a new Note, to introduce into the *Code* the terms isolectotype, isoneotype and isoepitype. He thought that the reason the terms were not in the *Code* was because they did not have any nomenclatural significance. He noted that duplicates of lectotypes, neotypes or epitypes did not have any status under the *Code*. If a lectotype was lost, it was not the isolectotype that replaced it. A new lectotype must be selected from amongst the original material that was eligible in the usual order. That was the reason they never appeared in the *Code*, but he understood that this had created problems for overzealous editors and others who had not wanted to include the term in publications because it was not in the *Code*, although they were perfectly clear and explicit terms and they appeared in the glossary that Hawksworth had recently produced and in many other glossaries, but the desire was to include it as a Note to give it some authenticity.

**Turland** referred to the Rapporteurs’ comments where it was noted that “Prop. FF could endow the three terms in question with the desired formal status, although it would not be appropriate to include them in a Note because their application is not already implicit in the *Code*. It would be much more suitable to include them as a Recommendation following Art. 9. An ‘Ed.C.’ vote would be so interpreted”.

**Reveal** noted that forty-two years ago he had proposed this as a snot-nosed graduate student and was thoroughly abused in Seattle. He found it a comforting thought to see that it had come back and was keen to let his colleague defend his actions.

**Gandhi** had also made the same proposal in the *Vienna Congress* because there were a number of editors from different countries, including his own departmental journal who would contact him as they were uncomfortable using the term just because it was not in the *Code*. The proposal was for other people who were doubtful.

**McNeill** reiterated the Vice-rapporteur’s point that it would make good sense if it appeared as a Recommendation in the *Code* that duplicates of these particular categories of type be so designated. He felt that it did not really represent a Note, because a Note was something that reflects a rule that was not explicit in the rule itself, but was implicit in the rule. There was nothing implicit in any of the rules about what created those terms. On the other hand he felt that, as they were in wide use and had obvious meaning, they were useful terms. Including them in a Recommendation, if it would meet the needs of editors and others, would be the best way forward. The Rapporteurs proposed an amendment that the content of the Note be included as a Recommendation.

[This was accepted as a **friendly amendment**.]

**Prop. FF** was referred to the **Editorial Committee** as amended.

**Prop. GG** (4: 98: 8: 0) and **HH** (18: 82: 8: 0) were both ruled **rejected**.

**Prop. II** (12: 15: 78: 0) was referred to the **Editorial Committee**.

**Funk** moved that the Section adjourn.

**Knapp** did so and thanked everyone for being brief, to the point and germane. She added that if anyone had coffee cups that they brought into the hall, could they please take them out, because if they did not, she and Ladiges would have to stay behind and do the tidying up and they would be cross!

## Third session

Tuesday, 19^th^ July 2011, 09:00–12:30

**Knapp** welcomed everyone back and expressed pleasure at the number of people who had returned. She went on to say that later in the week, there would be a discussion about Art. 59, an Article concerning fungi. As the issues that needed to be discussed were potentially not that familiar for those who normally dealt with vascular plants, there were a number of documents available for members of the Section to familiarize themselves with the arguments and the ideas proposed. Three documents were available: Declaration on Amsterdam, a Critical Response to the Amsterdam Declaration and a letter of concern about amending the *Botanical Code*. They could also be downloaded from www.mycotaxon.com. Lorelei Norvell was identified as available for further information.

**Kellermann** requested a half-hour discussion as for *Acacia* because some of the mycology issues were so controversial.

**Knapp** noted that this had been discussed and was planned for when the issues arose.

**Kellermann** continued that it was likely that the issues would come up late in the proceedings and suggested the half an hour be planned for Wednesday, instead of Friday.

**Knapp** confirmed that the timing would be looked into and that it had already been agreed with the mycological community that there would be time for discussion. On another practical matter she noted that the synopsis with the proposal under discussion would be visible on one screen and the electronic copy of the *Code* available on another.

### Article 9 (continued)

**Malécot’s proposal**

**McNeill** explained that although the day before had concluded Art. 9, there was an additional proposal linked to what had been discussed and Malécot would present it.

**Malécot** summarized that, the day before, Prop. FF had been accepted as a Recommendation dealing with isoepitype, isoneotype and so on. This was remaining from the Vienna Congress, when there were four terms referred to the Editorial Committee to be introduced into Art. 9 as a Note. These words were the three in Prop. FF and the fourth word was “paralectotype”. Thus he proposed an amendment to Art. 9 in order to introduce a new Recommendation that would read: “A paralectotype is a specimen cited in the protologue that was not subsequently chosen as a lectotype and that is not an isolectotype nor a paratype. Syntypes not chosen as a lectotype become paralectotypes after lectotypification”.

**McNeill** requested clarification as to whether Malécot was implying that they would cease to be syntypes and would now become paralectotypes, so therefore would not be in the normal sequence for future lectotypification.

**Malécot** replied that this would be from the time of lectotypification.

**McNeill** pointed out that the *Code* specified that if a lectotype was lost or destroyed then there was a sequence in which material should be selected, and what was suggested was not part of that sequence. He wanted to know if they would lose their status as a result of this.

**Malécot** replied that they would not lose their status.

**McNeill** was not convinced and suggested that some other proposals would be required to ensure that they did not lose their status.

**Malécot** had checked when there was a problem with a lectotype, and concluded that it did not seem to be a problem.

**Knapp** asked if there were five people to second the new proposal [for a new Art. 9.6bis]. [There were; the **proposal** was **seconded** and **supported**.]

**Redhead** was totally confused by what the status would be.

**Gandhi** encountered such items frequently as part of the International Plant Name Index project and his understanding was that in the past there was a debate whether to call the residues paralectotype or lectoparatype. After discussion with his colleagues, both at Harvard and New York, they believed that whatever was left after selection of the lectotype should continue to be called syntypes.

**Bill Barker** hoped that these would remain syntypes, just as a lectotype was a syntype. He felt that the solution to the problem that had just been raised was to change the proposed wording to: “a paralectotype is a syntype”. This was also considered useful instead of “other syntypes” in publications. Having a category to put on a det. slip, which says “this is a paralectotype” by the person lectotypifying at the time, would be very useful.

**Buck** thought “once a syntype, always a syntype”, unless it was raised up. He pointed out that a paratype was not a real type and that paralectotype, adding the prefix ‘para’ to a real type, was a source of confusion.

**Barrie** agreed with Bill Barker that this was not a good idea and that adding a new term was only going to confuse people. He felt this attempted to change the status of specimens that already had a proper name. He had experience with people trying to tell him that they had to change their syntypes to paralectotypes now, when the term did not even exist in the *Code*. He also pointed out that people had tried to introduce the term as far back as the ’70s. He thought it was better to leave it out and keep calling them syntypes. He also made the point that the addition would have to be an Article because it involved putting a new definition into the *Code*. It was proposed as an Article but Malécot had suggested making it a Recommendation, in which case it would have no force anyway and it would just result in more confusion.

**Marhold** was not sure that paralectotype was the right term but definitely wanted some term for what remained from syntypes after lectotypification, as they were no longer syntypes.

**McNeill** stated that they were.

**Marhold** repeated that they were not.

**McNeill** elaborated that under the *Code* they were syntypes and remained syntypes and stressed that this was important from the point of view of how a lectotype was chosen, should the existing lectotype be lost or destroyed. His concern was that this proposal would change that sequence and therefore introducing it would require some other alteration elsewhere although Bill Barker had pointed out a way in which this could be obviated.

**Marhold** did not feel that this interpretation was unanimous because he had encountered several opinions, as editor of a journal, that if a lectotype had been chosen, there were no longer syntypes in the true sense.

**Sennikov** found paralectotype to be a confusing term, for two reasons. First, as had been pointed out already, he felt that “para” belonged to quite a low level of type; a paratype was not a real type. Second, several people he knew believed that after lectotypification the other syntypes lost their value so much that they could even be thrown away without any danger to future nomenclature, because they were deemed to have lost their importance at the moment of lectotypification. He argued that lectotypifications can be overturned very easily, should some mistake be found, and such mistakes were not that rare. For example it may be found that the original material was heterogeneous and so the syntype that was selected as lectotype was not the best choice. He felt that, in principle, lectotypifications could be subject to revision and the suggested new terminology may exacerbate this erroneous practice.

**Janssen** requested that the relevant text in the *Code* be shown as there seemed to be confusion about what happened to the remaining syntypes after lectotypification. [Art. 9.4 was displayed.]

**McNeill** confirmed that there was no suggestion that a syntype ceased to be a syntype because of some other action.

**Lendemer** called the question.

**Knapp** asked the Section to vote on whether or not to go to a vote. [There was a sufficient majority in favour of voting.]

**Malécot’s proposal** was **rejected**.

**Knapp** had forgotten to mention a proposal that the Bureau wanted to put to the Section to automatically refer to the Editorial Committee, without discussion, any proposal that dealt entirely with an Example, unless there was any objection to the motion.

**Redhead** suggested flagging voted Examples so they did not go to the Editorial Committee.

**Knapp** corrected the proposal to all Examples that were not voted Examples.

**Talent** queried what would happen when there were two proposals, one of which was really an Example: could the Example be discussed with the paired proposal?

**Knapp** clarified that this would only apply to proposals that were solely to do with Examples.

[The **motion** was **approved**.]

### Recommendation 9A

**Prop. A** (86: 12: 13: 0).

**McNeill** introduced the first proposal by Prado and Moran under Rec. 9A, which addressed a Recommendation that was a hangover from pre-Tokyo *Codes*. It should have been deleted editorially at St Louis, but the authors had suggested a constructive change to the Recommendation, which received very positive support in the mail vote, 86 to 12. It was essentially saying that where no specimen was designated as type but a gathering was cited, then it was recommended that the specimen housed in the institution where the author was known to have worked be selected as the lectotype, unless there was some evidence that other material was primarily used.

**Buck** suggested that the Editorial Committee should deal with “housed in the institution where the author is known to have worked” as these herbaria are often in different institutions, not where the author actually worked but where the herbarium ended up.

**McNeill** took the point.

**Bill Barker** had had the experience where syntypes that were in other institutions were more representative of the protologue and added that some characters may not be represented in the specimen at the home institution. He felt that where the author worked was just one of the considerations, but that other considerations may take priority. He was against the Recommendation as worded.

**Gandhi** noted that Rec. 9A seemed to be applicable to names published prior to 1990. From 1990 a name would be invalid unless the institution housing the holotype was designated as cited.

**McNeill** agreed that was correct, but thought the first phrase of the Recommendation made that clear, because it was not possible to simply cite a single gathering but no specimen after 1990.

**Demoulin** had voted against the proposal [in the mail vote] and would keep voting against it because there were two points that he felt were not satisfactorily dealt with. First, he did not see why it could not be a holotype and had to be a lectotype. He gave a definite example of a potential problem that he felt was more important: the author whose types were in different institutions. He had a paper in press in *Flora Malesiana*, a report of a past symposium, where he had studied the typification of Corner’s polypores and clearly shown that some of the collections of holotypes were in Edinburgh, as many people had assumed, but several other holotypes, not lectotypes, were in Singapore. It was not always easy to find out what Corner has done when his herbarium was split between Europe and Singapore. He would prefer to have the whole thing deleted.

**McNeill** commented that if they were indeed holotypes, this would not be applicable and they would not just be a gathering with no institution specified. They would have to be only a unicate, or else with an institution specified, or they would not be a holotype.

**Wiersema** suggested that if the changes were not accepted, then the situation would return to what it had been.

**McNeill** refuted this, as the clause would be deleted editorially because it was contrary to the *Code* and the proposal was a suggestion to take what was there and make it meaningful. He assured Wiersema and the Section that if the proposal was defeated, then the Editorial Committee would simply drop it as a Recommendation as it would no longer be relevant.

**Knapp** reiterated that the vote would be to change the wording, amending the text of Rec. 9A.4: a “yes” vote would change the text, a “no” vote would mean that the Recommendation was deleted from the *Code*.

**Prop. A** was **rejected**.

**Prop. B** (47: 55: 10: 0).

**McNeill** moved on to Rec. 9A Prop. B, which, along with the following proposal, was providing and recommending more precise bibliographic information on type selection. The Rapporteurs had commented that those who feel this is appropriate as a Recommendation in the *Code* can support it. If you think it has too much detail, you will oppose it.

**Gereau** felt that the level of specificity in Recommendations was really trying to do the work of editors and authors for them. He encouraged leaving the *Code* as a functioning document that did not spell out every element of good practice, and assumed that editors could hold authors’ feet to the fire and make these things happen.

**Gandhi** recounted a recent situation when an author published a new species designating a holotype without having seen it but clearly noting that it had not been seen, although the isotypes had. He felt that even though this proposal was quite rigorous, he was not commenting that it should be accepted, but the concept seemed to be good.

**Prop. B** was **rejected**.

**Prop. C** (59: 49: 7: 0) was **rejected**.

**Prop. D** (16: 88: 11: 0) was ruled as **rejected**.

### Recommendation 9C (new)

**Prop. A** (39: 46: 7: 10) was **withdrawn**.

### Article 10

**Prop. A** (12: 22: *72: 0).

**McNeill** introduced the first proposal under Art. 10, to which the Rapporteurs had made the comment that it would be perfectly logical to go further than the proposal. The proposal was to delete the second part of clause (a) in Art. 10.5 but, as the Rapporteurs had pointed out, the whole clause was redundant except in a case where a type of a name of a genus or a subdivision of a genus was “otherwise chosen” [under Art. 10.2]. He elaborated that if no species name was included in the protologue, then it was impossible for an element [a type] to be in conflict with the protologue, because the only elements eligible as types are those that are included in the protologue. He went on that, in the case where a type was “otherwise chosen”, e.g. a specimen that was the type of a species name, as opposed to a type of a species name that was included in the protologue, then if this was in conflict with the description it must be superseded, and he mentioned that this was already in the *Code* [in Art. 10.2]. The Rapporteurs had suggested that an Editorial Committee vote would be interpreted as favouring deletion of the whole of the clause, and the result of the mail vote on this particular proposal was 12 in favour, 22 against and 72 in favour of referring to the Editorial Committee. He concluded that the mail vote had therefore taken the view that the clause might be entirely deleted.

**Greuter** thought that the comments of the Rapporteurs were not quite accurate because Art. 10 dealt with generic names, and in generic names the elements available as types were the names cited in the protologue. He pointed out that these names could be misapplied by the original author and gave the example of a very famous case, *Pseudolarix*, where designating a type under Art. 10 did not apply because there was only a single binomial in the protologue. In that case, he argued that there was no choice but to accept the binomial and its type, even if contrary to the explicit intent [of the author?] and to the whole remainder of the protologue. He went on that if there were several species mentioned by binomial when a new generic name was validated, and one of them was blatantly misapplied and did not fit the generic concept in all evidence and happened to be chosen as a lectotype, in those cases, the present provision made sense.

**McNeill** requested clarification as to whether that was a vote against the proposal or against the Rapporteurs’ editorial extension, or both.

**Greuter** clarified that it was against the Rapporteurs’ comments.

**McNeill** disputed the interpretation because he felt that if a person cited a name, the fact that he may have misapplied the name did not mean he had not cited it, therefore it was indeed part of the protologue, so he disagreed with the former Rapporteur [Greuter], but took his point that it was arguable. He suggested that the Section may rather just vote for the proposal and not the extension the Rapporteurs had proposed. He proposed a vote first on the Rapporteurs amendment to the proposal [This was **seconded** and supported by four others.] The amendment was to delete the whole of clause (a) in Art. 10.5.

**Knapp** clarified that this meant deleting “it can be shown that it is in serious conflict with the protologue and another element is available which is not in conflict with the protologue”.

[ The **amendment** was **rejected**.]

**Knapp** moved to a vote on the original Prop. A, as published in the synopsis, which was to delete the second part of clause (a) in Art. 10.5: “and another element is available which is not in conflict with the protologue”. [A show of cards was called for after the initial vote.] The Vicerapporteur pointed out that, because the previous two times when there had been a show of cards and subsequently a card vote, the result of the latter had been quite different to what was seen with the show of cards, and so he proposed to go to a card vote. [The Section voted to do so.]

**Prop. A** was **accepted** on a card vote [290: 147; 66.7% in favour.]

**Prop. B** (3: 77: 1: 24).

**McNeill** pointed out that the next proposal, Art. 10 Prop. B, was a proposal for a voted Example, which would be discussed. The Rapporteurs had cautioned that it could be very nomenclaturally disruptive, were it to be accepted. The mail vote, to some extent, reflected that, in the sense that there were only three in favour, and in fact there was a 77% “no” vote. However, he felt that it was more complicated because there was a genuine problem that had led to the proposal: to know exactly what works were covered by the provision of the existing voted Example in Art. 10, dealing with largely automatic methods of selection of a type.

The Rapporteurs had suggested that if the proposal was not acceptable to the Section, as appeared unlikely given the mail vote, then it would be useful to set up a Special Committee to try to clarify this by means of appropriate lists, or whatever way the Committee chose. He suggested the Section consider the proposal first and, if it passed, that was the end of the matter but, if it was defeated, there would be an additional proposal to consider whether or not to appoint a Special Committee.

**Gandhi** elaborated a little on the proposal by saying that many generic names were typified by Britton and Brown in their 1913 work [*Manual of the flora of the northern States and Canada*, ed. 3] and that was what was listed in ING [Index Nominum Genericorum: http://botany.si.edu/ing/]. Unless people read the *Code* very carefully, they would take for granted that whatever was listed in ING displayed the correct type species information. He went on that within that 1913 work it was clearly stated that the authors were following the *American Code of Botanical Nomenclature*, which was purely mechanical as far as the type species designation was concerned. He reported that Britton did not always say anything about what kind of nomenclature he was practising and, although everyone knew Britton was a promoter of the *American Code*, in a given work he might not have cited such a statement. In the absence of citation of such a statement, should we have to take it for granted that Britton was following the *American Code* or was it left to our discretion? Gandhi also mentioned that it was problematic whether what was cited in ING had to be followed or not. His belief was that, as long as Britton did not state that he was following the “American Code”, whatever typification he made was acceptable. He and Reveal had had extensive discussions with both McNeill and Greuter to come up with some solution, whether to reject all the typifications by Britton, or use some exceptions.

**McNeill** felt that that was a very cogent argument for a Special Committee.

**Barrie** thought that the proposal identified a real problem but did not think it would solve it. He was in favour of having a Special Committee, especially if it was going to come up with specific works that could be listed, and then the issue would be removed from the *Code*, so that the lists could be ruled as mechanical whether or not they actually were, and it would solve the confusion. He felt it was a problem that ING accepted Britton and Brown typifications as they ended up in TROPICOS, so a lot of people looked at them as being acceptable when actually, under the *Code*, later typifications were correct.

**Reveal** [one of the proposers] urged the Section to vote “no” on the proposal and then immediately vote for a Special Committee. He called the question. [There was a sufficient majority in favour of voting.]

**Prop. B** was **rejected** and a new **Special Committee** was established to deal with the issue [the Special Committee on Publications Using a Largely Mechanical Method of Selection of Types (Art. 10.5) (especially under the *American Code*)].

**Prop. C** (34: 48: 7: 11) was **withdrawn**.

### Article 11

**Prop. A** (70: 8: 32: 0) was ruled referred to the **Editorial Committee**.

[*The following discussion of a new proposal by Herendeen on Art. 11.8 took place during the Tenth Session on Friday afternoon.*]

**Herendeen’s proposal**

**McNeill** introduced a new proposal by Herendeen on Art. 11.8: “Delete in Art. 11.8 all reference to subfossil and delete ‘subfossil’ from the Glossary”.

**Herendeen** added that it was a simple housekeeping matter to simplify the *Code* as “subfossil” had no nomenclatural purpose and he argued that mention of it in the *Code* was not useful. The suggestion was to get rid of it and simplify the text. [The **proposal** was **seconded** and **supported** by three others.]

**McNeill** interpreted this as there being no doubt in the minds of scientists working on Pleistocene material that they were dealing with a fossil. He asked if it was perfectly clear when a thing was a fossil and when it was not a fossil.

**Herendeen** confirmed that it was.

**Knapp** moved to a vote on deleting the word “subfossil” from Art. 11.8 and the Glossary. As the “ayes had it” she concluded that there were no longer any subfossils. [Laughter.]

**Herendeen’s proposal** was **accepted**.

[*Here the record reverts to the normal sequence of events.*]

### Article 13

[*The following debate, pertaining to Art. 13, took place during the Seventh Session on Thursday morning with discussion on Art. 45.*]

**Prop. A** (83: 16: 5: 3).

**McNeill** introduced Art. 13 Prop. A, which dealt with the groups that fall under the *Code*, making it explicit in discussion of startingpoint dates. He suggested that the only issue for debate was whether it was necessary.

**Dorr** felt it would be better to state “names of *Microsporidia*”, rather than turn *Microsporidia* into an adjective.

**Knapp** suggested that could be dealt with editorially and felt it was a good suggestion.

**Buck** wondered if the *Code* had any authority to tell those who worked on *Microsporidia* what to do considering that the group was now excluded from the *Code*.

**Knapp** considered that the proposal was being helpful, and being helpful was always nice.

**Barrie** pointed out that, since the Preamble had been changed, this would be a Note.

**McNeill** felt perhaps a footnote might be the best way to deal with it in an Article regarding the starting points of groups falling under the *Code*, but that would be decided editorially.

**Alvarado** thought it would be fine to add the words “Since 1 January 2013 microsporidian names are governed by the *International Code of Zoological Nomenclature*”, because before that time they were still governed by this *Code*, at least since the Vienna Congress, so if that was not added he wondered if it would mean that all the names that had been published in the six years under this *Code* would be invalidated.

**McNeill** indicated they would not.

**Knapp** clarified that the vote was on Art. 13 Prop. A to add at the end of Art. 13.1(d) the following sentence: “Names of *Microsporidia* are governed by the *ICZN* (see Pre. 7)” as a Note or a footnote.

**Prop. A** was **accepted** as amended.

[*Here the record reverts to the normal sequence of events.*]

**Prop. B** (43: 20: 13: 24).

**McNeill** introduced a proposal from Silva to eliminate the later starting-point dates for blue-green algae, *Cyanobacteria*, cyanoprokaryotes. The proposal had received quite respectable support in the mail vote: 43 in favour; 20 against; 13 that it go to the Editorial Committee, which was deemed a little difficult by him; and 24 that it go to a Special Committee. However, he reported that it had also been considered by the Nomenclature Committee for Algae, which was much less enthusiastic and in fact voted three in favour and nine against the extension, 60% “no”. The Rapporteurs’ comments had summarized what the proposal was: it pertained to a long-standing issue and the arguments were presented that any later starting-point dates created problems because an arbitrary line was being placed across a period in which binomial nomenclature was used, whereas in the case of starting with Linnaeus, there had been no previous binomial nomenclature. He reported that a second problem in this particular case was that the startingpoint work included a large number of names that were not validly published, on the grounds that they were not accepted by the author in the original work, and it was very hard to know when they were later published. He suggested that that was presumably why there was good support from the mail vote but very little support amongst the specialists and the Committee for Algae.

**Prud’homme van Reine** spoke on behalf of the Nomenclature Committee for Algae, which was against the proposal despite it being proposed by its chairman. He noted that there was supposed to be a Special Committee on the harmonization of cyanophyte nomenclature, which had encountered delays. The formation of the Special Committee was very slow, but now a list of suggested members existed and that was in report no. 10 of the Committee for Algae in February 2011. He suggested it would be better to wait another six years until there was agreement with the prokaryote people.

**Barrie** provided a little background to what Prud’homme van Reine had said. In Vienna there was a proposal to have a Special Committee look at reconciling this issue along with some of the bacteriologists, but the problem was that no one signed up for it in Vienna, so the Committee was never formed. Prud’homme van Reine sent him a list later on but the Committee still ended up never being formed, so what was needed was to reform the Special Committee, which already had members set up to be enlisted. He suggested letting them do their work so that they could report again to the next Congress.

**McNeill** thanked Barrie for mentioning that, because he was just going to raise that next. That Committee was to report to the Melbourne Congress; the Committee automatically ceased to exist after the Melbourne Congress, so it would be necessary to appoint a new Special Committee on this topic, as was clearly desirable.

**Demoulin** was also chairman of a committee [the Committee for Fungi], and was not always in agreement with the majority of the Committee, and he sympathized with Silva, who had given great attention to this problem, which he knew very well. His experience was with the situation in the fungi, but half of his life was devoted to algae and mostly blue-greens, *Cyanobacteria*. He hoped that the system of later starting points that he felt was impossible to work with would be abandoned where possible. He suggested that Silva had already acted as if there were no later starting points, giving his work for the Indian Ocean as an example of his use of 1753 as the starting point.

**Redhead** noted that there was some overlap with the mycological literature for these groups, for some of the names for things that were tremellaceous. He supported the proposal.

**McNeill** did not think this would affect the names, because the starting point would be determined not by the work it was in but by the identity of the type, so it would have a 1753 starting point, even if it was in one of those works.

**Knapp** moved to a vote on eliminating later starting points for the cyanoprokaryotes.

**Prop. B** was **rejected**.

**McNeill** explained that the next vote would be to reestablish the Special Committee that failed to report to this Congress, for good reasons as described, to deal with the harmonization of the nomenclature of *Cyanobacteria* or blue-green algae.

**Redhead** requested a card vote, seeing as so few people voted.

**McNeill** noted that it was very clearly defeated nearly two to one and could not see any basis for the proposal being successful, he did not expect a big bias in the institutional votes but was willing to go to a card vote if Redhead insisted.

**Bill Barker** explained that the reason he did not vote was because it was being moved to a Special Committee. He thought he could leave it to people who knew more about it, and then it would be resolved if it was rejected by people who knew.

**Knapp** proposed a vote on establishing a Special Committee on this so that the Section would be sure that the issue had been moved to the people who knew.

The **motion** was **seconded** and a new **Special Committee** was established [the Special Committee on Harmonization of Nomenclature of *Cyanophyta/Cyanobacteria*].

**Knapp** hoped that people would sign up this time.

**Prop. C** (18: 81: 5: 6) was **withdrawn**.

### Article 14

**Prop. A** (79: 21: 7: 1).

**McNeill** introduced Art. 14 Prop. A, which was seeking to resolve what had been a matter that many had overlooked, that the *Code* permitted the conservation of names only at the ranks of family, genus and species. It did not permit conservation of names at infraspecific level, or of a subdivision of a genus. Consequently, if a generic name was proposed for conservation with a type different from what would be the type under the other provisions of the *Code*, and that generic name was based on the name of a subdivision of a genus, then automatically the typification was broken, because the type of the name of the subdivision of the genus must remain what it was under the *Code*. Changing that would be tantamount to conserving a subgeneric name or a sectional name. The proposal was seeking to overcome this difficulty so that such basionyms could be treated essentially as conserved, whereas the broader issue of conserving names of all ranks had generally not received much support at previous Sections. The mail vote was quite positive: 79 in favour, 21 against.

**Wiersema** added that there were cases like this with the conserved generic names in App. III and he knew of at least one case in the species name conservations. He felt that the proposal was needed because there was ambiguity about how to interpret the types of the basionyms in these cases, whether they would be the same as the conserved type or not. He was strongly in favour of the proposal.

**Gereau** felt that the Section really needed to take a hard look at the whole philosophical direction the *Code* was going and not just this particular adjustment. He asked whether it was desirable that the *Code* was a set of basic principles from which any intelligent person who cared to master it was able to apply the rules or a body of special legislation on individual cases, so that the *Code* continued to grow in length and complexity. He argued that opening the door to conservation at ranks other than of genus and species was an undesirable move in the latter direction and should be roundly defeated.

**Sennikov** disagreed, saying that it was not a bad door to open the way to conserve names of infraspecific ranks, for example, or sectional ranks. He felt that his was the way to legally retain the original material and original types of conserved names in cases when they were originally published at ranks different to those allowed for conservation. He suggested taking this case as an exception and making a rule that would allow retaining the original material and the history of those names already in conserved entries in the *Code*.

**Alvarado** noted that sometimes genera were quite subjective and from an evolutionary point of view the point at which a clade was named as a genus could be a little bit arbitrary.

**Knapp** pointed out that this was a point about science rather than the current proposal.

**Alvarado** continued, claiming to be talking about the proposal as well, because sometimes what was called a subdivision of a genus would, in another group of organisms, be a genus itself. His point was that he thought that subdivisions should have the same right to be conserved as genera.

**McNeill** clarified that the *Code* did not provide for that and there was no proposal at the moment to do so. He also encouraged speakers to address whether the proposal was one that was a step in what they would consider the right direction or not.

**Hawksworth** wondered whether it was time to reconsider whether this should apply at all ranks or at least family and below, because there were certainly going to be cases, for example *Aspergillus*, where the community would definitely want to conserve subgeneric names.

**McNeill** did not consider that that would be an amendment to this proposal, as it would be much too extreme to be treated as an amendment. He pointed out that Hawksworth was free to make a proposal later in the sessions to be dealt with in other business.

**Hawksworth** thought it would be good to get a general feeling of whether people would welcome opening that up further.

**Knapp** suggested that the vote on the proposal in question may show the general feeling.

**Hawksworth** disagreed as he felt that was a different problem.

**McNeill** preferred to first consider the proposal and, if the Chair was agreeable, then have a quick show of hands on the broader issue afterwards, which may provide guidance as to whether it was worth preparing a proposal.

**Hawksworth** did not want to waste the Section’s time but thought it was something worth revisiting.

**Wiersema** wanted to underscore that the proposal would not “open the door”. It had been pointed out that perhaps some people wanted that door opened, but this was not opening the door to conservation at infraspecific or infrageneric ranks. It would still only be possible to conserve at species rank, or generic rank with this provision, it just resolved some of the typification ambiguity when that was done.

**Knapp** thanked him for the clarification.

**Van Rijckevorsel** wanted to point out that there already were conserved names at other ranks, namely in Art. 14.10, which said “A conserved name, with any corresponding autonym, is conserved against all earlier homonyms”. So he felt there was a precedent, the rule was that there were only listings at the primary ranks but conserved names at other ranks already existed.

**McNeill** requested clarification as he did not understand what was meant, as a homonym would necessarily be at the same rank.

**Barrie** thought that the argument was that the autonym itself was also conserved, along with the conserved name, although he was not sure that he read it that way.

**McNeill** apologized as he had misheard homonym and not autonym. He felt it was an interesting point but was not sure that it was immediately relevant to the proposal.

**Knapp** reiterated that Art. 14 Prop. A was to add a new penultimate sentence to Art. 14.1 to solve the problem that McNeill had been discussing.

**Prop. A** was **accepted**.

**Prop. B** (88: 11: 11: 0).

**McNeill** explained that Prop. B was also making clear something that most people had assumed but was not spelled out in the *Code*, and that was that “The listed type and the spelling of a conserved name may not be changed except by the procedure outlined in Art. 14.12”. He elaborated that meant that both the orthography and the type were already indicated as being conserved *ipso facto*, even if not being explicitly conserved. Sometimes a name was conserved with a conserved type explicitly, in other cases the name was conserved for other reasons and the type may be the type that would be correct under the *Code*. But the *Code* made clear that that listed type was *ipso facto* conserved by the very conservation process. This was a proposal to clarify that this also applied to the spelling of the names, something that most had assumed. The mail vote was positive: 88 for and 11 against.

**Demoulin** for once did not agree with the Rapporteur, because he felt that, had this provision existed 30 years ago, it would have been impossible to implement the change in starting point in fungi that was made at the Sydney Congress. He thought some flexibility should be retained in editing the list of conserved names, because when there was a change in the *Code*, where new evidence was discovered, it should be possible to correct an entry, not to reverse an explicit decision but for many, many things with inherited entries that may sometimes need to be adjusted or corrected. After the Sydney Congress, as the responsible person in the Editorial Committee for the conserved names of fungi, he worked hard to get the list of conserved names in agreement with the new rules, of course with the control of the Committee for Fungi. All the proposed additions were submitted to the Committee. He suggested that if they had had to use this very precise and strict rule, they would have had to publish it in *Taxon* and wait maybe two, three years before all the changes would have been discussed in Committee.

**Greuter** agreed and also supported what was being proposed, in principle. He **moved** an **amendment** to add the words “except in the case of correctable errors”, with perhaps a cross-reference to Art. 60. What prompted him to do that was that in many cases, such as had happened in the past and he felt would happen again, the rules in Art. 60 were modified. It could happen, for instance, that some Congress, however ill-advised, decided to eliminate the hyphen. Proposals would then have to be made to modify the entries in App. III or IV to take away all the hyphens that had been taken away by a former vote on Art. 60 if this was unamended. He reported that there had been cases in which there had been a misprint in App. III and the *Code* had been approved by the following Nomenclature Section as a whole so the misprint became the conserved name and, unless there was the possibility to correct these things, a proposal would have been necessary to correct that misprint. [The **amendment** was **seconded**.]

**Reveal** gave the example of one instance in App. IIB, where there was a mistake made in the editing so that the same place of publication was given for two entirely different names and it was simply an entry error. This was known and had been corrected and he argued that it had to be possible to correct editorial errors.

**McNeill** confirmed that was perfectly correct, but that this particular proposal would not cover it. It would cover the issue of the misspelling, and there was at least one name in the *Vienna Code* that was misspelled that went back a little before the *Vienna Code* and had now been corrected. He agreed that it would not apply to the type of case that was described because that was not the spelling of the name.

**Reveal** wanted to know how a simple error that was inadvertently made could be corrected if there was no provision in this that allowed corrections of an editorial error.

**Knapp** reminded the Section that it was the amendment to add the phrase “except in the case of correctable errors” under discussion.

**Wiersema** was not sure that a reference to Art. 60 was desirable, because there were other correctable errors that might come into play if the gender was changed. If the gender of a generic name was conserved and the ending of the species epithet that might be the type had to be corrected, this would need to be possible.

**Knapp** asked if there was a friendly amendment to Werner’s amendment.

**Wiersema** suggested taking out the reference to Art. 60. [The **friendly amendment** to the **amendment** was **accepted** by the proposer Greuter]

[The **amendment** was **accepted** as amended.]

**Wiersema** just wanted to confirm that by deleting Art. 60, an inadvertent typographical error would be covered by correctable error.

**Knapp** confirmed that it would.

**Prop. B** was **accepted** as amended.

**Knapp** asked for a straw poll for Hawksworth as an indication of whether he should spend any time working on his suggestion to extend the provision for conservation and rejection to all ranks. She reported that there were slightly more against than in favour and suggested that Hawksworth could do what he wanted. [Laughter.] She asked those people who wanted Van Rijckevorsel’s printout of the various proposals for *Acacia* to raise their hands to receive a copy and when they ran out added that more copies would be made in the next break.

**Prop. C** (74: 2: 15: 10) was **withdrawn**.

**Prop. D** (63: 16: 26: 0).

**McNeill** introduced Art. 14 Prop. D addressing the names in App. IIB, which differed from the other conserved names, as it was the list of conserved names of spermatophyte families that were conserved not against any specific name but were a list of conserved names. This was a proposal that not only should the names be conserved but also the authorship and place of publication; always assuming that they were validly published there. This was long thought to be the case and indeed there was provision in the *Code* for it, but the footnote protecting that was deleted at St Louis [*Tokyo Code* Art. 14 Note 1 footnote], and this had meant that a great deal of work had had to be done on names that were thought fixed and established. It seemed to him that if names were conserved and if they were in fact validly published, it should not be necessary to continually change the authorship and place of publication by looking at obscure late 18th Century works. The proposal posited that there should also be preservation of the place of publication and the authorship in addition to the conservation of the name and type, implicit in the family name. This meant an earlier isonym would not displace the name as listed in App. IIB.

**Turland** added a few figures after the introduction to the proposal. Prior to the St Louis Congress the authors and places of publication of the names on App. IIB were protected by a footnote in Art. 14, which gave temporary protection but with no end date to that protection. That footnote was deleted at the St Louis Congress and consequently the names were subject to being corrected if earlier isonyms were found. Of the 466 conserved names on App. IIB, something like 40% were potentially published earlier, and sometimes by different authors to what was stated in the Appendix.

He and Barrie, as a subset of the *St Louis Code* Editorial Committee, extensively reviewed these suggested earlier isonyms to see whether they were indeed validly published and whether they were ranked as families. Consequently the result was that 102 entries, about 25% of the entries in App. IIB, were changed, so the names remained the same and the types of the names remained the same, but the authors and places of publication and dates of publications changed.

At the *Vienna Congress* there was a proposal to reintroduce the 1789 startingpoint date for suprageneric names that had been in effect until the St Louis Congress, which meant that some of the names that were pre-1789 in App. IIB in the *St Louis Code* had to be changed to the first available validation after 1789. That was usually the actual starting-point work, Jussieu’s *Genera Plantarum* of 1789, so 35 names that were attributed to Adanson, for example, in the *St Louis Code* App. IIB were changed, mostly to Jussieu.

In the intervening six years between the St Louis Congress and the Vienna Congress more and more suggested earlier isonyms also came to light. These were checked thoroughly by the Editorial Committee again, and also by the then Special Committee on Suprageneric Names. This resulted in a further 70 entries being changed in App. IIB. This has led to tremendous instability between the *Tokyo Code*, the *St Louis Code*, and finally the *Vienna Code* in the names in App. IIB.

In practical terms this meant that the names still had the same types so their application was not affected. The only major difference really in a few cases was whether the date of publication was changed, and there were two competing synonyms, both conserved in App. IIB. It had necessitated about half a dozen “superconservation” proposals where a widely used family name was actually predated by an earlier family that was a synonym, or considered a synonym under certain circumscriptions. So the superconservation proposal that had been necessary was to conserve the commonly used later name over the less wellknown earlier name.

He read a paragraph from the proposal. “In conclusion, I cannot help but feel a little sad about all the hundreds of personhours spent on this purely academic exercise that might have been better applied to, say, science and conservation. Anyway, what is done is done. What we could still do, however, is prevent a similar waste of time and money in the future.” So his proposal was to extend the protection currently afforded to App. IIB to include the authors together with the places and dates of publication, which would be deemed to be correct even if an earlier place of valid publication, by the same or a different author, was discovered.

**Barrie** added a little more background. The first list in App. IIB of the conserved family names went into the *Code* in 1959 in Montreal. When this issue was looked at in St Louis eventually he had gone back and started looking at the original writings in *Taxon* by the authors who came up with the list and some of the discussion in the report. It was pretty clear from that that their intentions were it would be a list where not only the names themselves, but their authors and place of publication would be conserved. They looked at it as a relatively small list, well-defined, fewer than 500 names, that they could all agree on would be used in this way.

Unfortunately that intention was never written into the *Code*, if it had been put into the introduction to App. IIB then there would be no problem. The family names became considered similar to the conservation of generic names where authors and place of publication were not conserved. The way the *Code* was written they had to be treated that way but the true intent of the list at the beginning was that everything would be conserved, and it would just be a practical tool for people to use.

**McNeill** added that Bullock, the primary person [involved in compiling the original list], chose the starting-point date of Jussieu’s *Genera Plantarum* simply because it was so difficult to determine when things might have been first published that he thought that taking it as an arbitrary starting point, and having the list as one that was maintained regardless of earlier names that might come to attention, would be appropriate.

**Wiersema** could not support the proposal as it was. He could support preserving the place of publication, but felt that inconsistencies in the *Code* over time between some of the Appendices could result from applying this to the authors and dates. If there were valid reasons, e.g. that the authorship of some part of a work was attributable to someone else, and there were conservation proposals for genera or species in that same work, then the authorship in App. III could end up being different from the authorship in App. IIB. There could be a similar situation with dates.

He thought that as long as the place of publication could be salvaged or preserved, that was the most critical thing. He did not want to see the possibility of introducing inconsistencies in the *Code* Appendices from one Appendix to the other over time if the proposal was accepted.

**McNeill** interpreted that Wiersema was suggesting something similar to what he had proposed for another Article, where corrections of errors would be excluded so that the place of publication was maintained, but any error relating to that place of publication, most notably the date and possibly the authorship, would be correctable.

**Turland** explained that his intention with the proposal was to go somewhat further than that, but he conceded that it could indeed create inconsistencies, for example, if a book was discovered to have been published two years earlier than was thought, a name in App. IIB would have a fixed date, and then everywhere else where a name from that same book was cited, the date would be the actual known date, which might be different. Or it could be the author that was different. His question was why that would be a problem, he wondered what problems it would cause.

**Wiersema** thought that people, himself included, looked to the Appendices to get answers about the proper dates of publication, or the proper authorship. In family names he did not think authorship was terribly important, and most people did not cite them anyway. But he did think the place of publication was something that needed to be worried about. In the proposal it was suggested that because of all the careful work that had already been done the chances of these things causing problems in the future were much diminished. He preferred to be able to correct things that needed correcting over time. He suggested an **amendment** that the proposal only be concerned with preserving the place of publication and allow correction of the authors and the dates.

**Turland** added that this would presumably also include errors of bibliographic citation, although he thought that because App. IIB had been gone over so many times the chances of any bibliographic errors remaining was small, but if, for example, the page number was wrong, that would also be correctable.

**Knapp** summarized the suggestion to amend the proposal with the exception of correctable errors, as was done in another amendment. [This was not accepted as a **friendly amendment**. It was **seconded**.]

**Reveal** found that he was apparently responsible for what he thought was described as a waste of time and energy… [Laughter.]

**Turland** confirmed that was indeed what he had suggested.

**Reveal** continued that he happened to disagree with that characterization of what he had done with Ruud Hoogland and Werner Greuter and John McNeill and others, including Nicholas Turland, for the last 20 years. He did not think it had been a waste of time. It indicated the need for critical detailed review of what was done. Therefore, he supported what Wiersema was suggesting by adding the amendment with the exception of correctable errors, with “correctable errors” being understood to mean typos, or inadvertent errors that were discovered. As for bibliographic citations, there were going to be some, he gave the example that when somebody discovered when Dumortier’s 1829 work was actually published in 1830, which it was, then that would change.

**Gandhi** agreed with Wiersema and gave an example from a few months previous when he was reviewing a generic treatment for *Flora of North America*. He had come across a situation where in both ING and the *Code* a conserved generic name had incorrect authorship because everyone just looked at the title of the book and assumed that the relevant person printed on the title page was the author of the whole treatment, but for the particular family, a different author was cited who was responsible for all the novelties. So for *Flora of North America* and IPNI he had been able to correct the authorship and so forth and ING would be correcting the details soon. He expected the same thing for the *Code*.

**Barrie** had a couple of issues: one was, what constituted a correctable error? The other was, how many errors would there still be in the list? First there was Bullock’s list, which was fairly well done, and then he knew that Jim Reveal and Ruud Hoogland had spent a lot of time beavering away at this, looking at these names, where they were published, who published them and worked really hard to get them accurate.

He thought the list itself was for the most part probably pretty accurate. He felt that any errors that remained were probably going to be going to be pretty infrequent but he did not think they should be left and ignored. He suggested they could probably be solved by putting a corrective conservation proposal in. Because he thought there would be very few of them and it would not be an issue very often, he supported the original proposal.

**Harley** felt that it was important to think of the future when it was possible that people may not be quite so careful, and therefore the fact that changes may need to be made should be allowed for. He was very much in favour of correctable errors.

**Reveal** responded to Barrie by pointing out that the wording in the proposal was “treated as correct in all circumstances and consequently are not to be changed”, therefore, a conservation amendment to make a change by this provision was prohibited.

**Van Rijckevorsel** understood that the last remark was not addressed to the amendment but he was thinking of using Art. 14.8 as a model. So if there was something very important, there was a mechanism to…

**Knapp** recommended keeping that comment for discussion of the proposal, because the current discussion was on the amendment. She then moved to a vote on the acceptance of the amendment to strike out the words “authors and dates of publication”, and leave only “places”, and to add “with the exception of correctable errors” to Prop. D at…

**McNeill** requested clarification from the proposers of the amendment as he had the impression that there were alternatives rather than both the deletion and the addition.

**Wiersema** clarified that he was proposing the deletion but was okay with the addition at the end.

**McNeill** felt that the point was that they were alternatives and not both going in[to the *Code*]; it was one or the other.

**Wiersema** clarified that it was just the last.

**Knapp** confirmed that the proposer was not suggesting that the authors and dates of publication be deleted from the proposal.

**Turland** felt that that would be meaningless.

**Knapp** had understood that Wiersema was suggesting that both the deletion and the addition were the amendment.

**Turland** summarized that if you found an earlier place of publication, it would be a correctable error, so the rule would be meaningless.

**Wiersema** was not sure correctable errors would allow you to correct the authors as in the *Code*, there was nothing specified as correctable that dealt with authorship unless the whole of Art. 46 was considered to require correction of something having to do with the ascription or some… but he wondered if those instances were considered correctable and asked if the date of publication was a correctable error. He ended by concluding that the part at the end was not necessary.

**Knapp** reiterated that the Section was voting on the amended amendment, which was to just strike out “The authors together with” and “and dates”, so this left “the places of publication” that were conserved.

[The **amendment** was **accepted**.]

**Greuter** felt that the amendment was fine. However, he had noted reading the synopsis a fact that was not unusual: that was disagreement between a proposer and the Vice-rapporteur. The fact that was unusual was that they were the same person in this case. [Laughter.]

**Knapp** asked Turland if he had a split personality.

**Greuter** explained that in the proposal, Turland had suggested and intended that the citations should be maintained irrespective of whether the name was validly published in that place. In the comments he had contradicted this together with the Rapporteur. Greuter suggested that there were two ways out: let the original proposer’s intention win, and in that case in those two places cited in the Rapporteurs’ comments [Art. 6.3 and 12.1] just add in parentheses “but see Art. 14 etc.” or let the present, strict interpretation stand. In that case, the reverse would be done. His opinion was that the original intent of the proposal was a better one, because if the entries of names not validly published in the conserved place were to be altered, another problem that had not been named would be encountered. There may be a validly published name in that place but it may not be the name of a family. This had been a frequent problem in the past, where they were orders or subfamilies, so they were not the validly published name of a family where they were conserved. His opinion was that things should be corrected completely and the present entry should win, notwithstanding possible errors in rank or valid publication or ambiguity.

**McNeill** confirmed that Greuter was proposing an amendment, that this would also cover what turned out not to be validly published names by appropriate qualification in the Articles that were referred to.

**Greuter** added that this would be inserted by the Editorial Committee.

**Turland** had originally intended to do that until the Rapporteurs had their Rapporteurs’ meeting in the previous year where the Rapporteur had pointed out that what the Vice-rapporteur was proposing was impossible. [Laughter.]

**McNeill** objected that he hadn’t said it was impossible…

**Turland** was keen to accept the change as a **friendly amendment** if it was deemed to be possible by the Rapporteur.

**McNeill** clarified that the amendment was to add “including names which were not validly published” and instruct the Editorial Committee to modify the Articles that would preclude this. Essentially the difficulty was that a conserved name was being discussed. He added that if a name was not validly published of course it was not a name under the *Code*, but that “name” could be qualified by making this an exception in the definition of that term.

**Reveal** highlighted that the purpose that Greuter had assigned to him and Ruud Hoogland in the late ’80s was, among several things, to determine the validity of names. He believed that there was not a single family name in which the validity was questioned.

**Turland** confirmed that there was not a single case that he was aware of, and he could not actually remember any [past] cases. Maybe there were one or two cases from the *Tokyo Code* where a name was found that was not validly published. He thought they had found a few cases where they were, as Greuter had pointed out, at a different rank.

**Reveal** believed that since St Louis that had been one of the things that they had “wasted their time” to resolve. [Laughter.]

**Turland** agreed.

**Reveal** continued that if the Section wanted to vote on the amendment, that was fine, but he would be surprised if there was any single name in App. IIB that was not valid.

**Turland** would also be surprised, but was not willing to bet his life savings on it not being the case. [Laughter.]

**Redhead** suggested changing the friendly amendment to say “including names that otherwise would not be validly published”, because it was certainly not desirable for names to continue to be invalid. [This was accepted as a **friendly amendment** to the **friendly amendment**.]

**Knapp** moved to a vote on the amended proposal to add a new Article to Art. 14: “The places of publication cited for conserved names of families in App. IIB are treated as correct in all circumstances and consequently are not to be changed, including names that otherwise would not be validly published”.

**Prop. D** was **accepted** as amended.

**Prop. E** (45: 16: 45: 0).

**McNeill** introduced Prop. E, which was just an editorial consequence.

**Turland** confirmed that Prop. E was a necessary consequence of what had just been passed, whereby the *Code* said that a name may not be conserved against itself, and the proposal simply allowed an exception to that rule, which would occur in the case of the Article that had just been passed.

**Knapp** moved to a vote on Prop. E, which she described as the necessary corollary to the amended proposal that had just passed.

**Prop. E** was **accepted**.

**Prop. F** (54: 34: 18: 0).

**McNeill** noted that the next proposal, Prop. F, continued in the same theme to extend the same principle agreed to for App. IIB to the other Appendices in the *Code*: authors together with places and dates of publication cited for conserved names of families, genera and species in App. II, III and IV. Although App. IIB had been dealt with, App. IIA had not been considered: it dealt with family names in certain other groups that were conserved in the normal way against some other name(s).

**Turland** added that it seemed logical to amend the proposal to bring it in line with what had just passed with similar wording to Prop. D.

**McNeill** understood that the proposer was going to change the proposal so that it was of comparable wording to what had been approved for App. IIB.

**Gereau** felt that App. IIB was a far different beast than the other Appendices as the amount of careful scholarship that had been expended—not wasted—upon it had produced a very impressively clean and correct document. He thought that the other Appendices had had a great deal of work put into them but nothing comparable to App. IIB, and the current proposal went far too far and would constitute nothing more than the possible enshrining of a lot of errors that should be corrected. So he felt it should be rejected.

**Demoulin** agreed that the Section could not accept the proposal for the same reason already discussed on the former proposal, which finally was accepted but in an amended form.

**Redhead** was also worried that there had not been the intense examination of the names on the lists, and that enshrining them in such a way would fix a lot of errors that were potentially in there.

**Reveal** suggested that because of Prop. D passing that the words “of families”, need not be included in the…

**McNeill** interrupted that that was not the case, as he had pointed out, there was App. IIA, which had not been covered.

**Reveal** understood that and questioned whether the intention was to extend this down to all families that were conserved in App. II.

**Turland** confirmed that was the case.

**Reveal** suggested that what had been done for Prop. D should be deleted…

**McNeill** felt that could be dealt with editorially. The point was, he felt, whether the Section wanted to support a proposal of this sort, the final wording would be dealt with at the Editorial Committee and the two proposals would be integrated. One had already been accepted; the one under discussion was much broader and would, of course, include the other, and that would be dealt with editorially. The order was chosen simply because there was a perception that there would be more support for the first proposal than there may be for the one under discussion.

**Wiersema** did not support the proposal for the same reason as the last one, but also for the fact that it said “correct in all circumstances and consequently are not to be changed”. It would not allow for the possibilities under Art. 14.12 of amending a conservation entry through a proposal. There were cases where that had been done.

**McNeill** had not appreciated that point when he read it initially and thought it a valid one that the proposer might want to address. He noted that the thought that this would exclude action under Art. 14.12, a proposal to change the type of a name, to modify a name through the due process, was certainly not in the Rapporteurs’ comments. This could simply be avoided by adding “are not to be changed except under the provisions of Art. 14.12”. [ Turland **accepted** that as a **friendly amendment**.]

**Barrie** wanted to know if including names that otherwise would not be validly published had implications for names of genera and species that it did not have for names of families.

**Knapp** interpreted that he was asking whether including the clause for names that were otherwise not validly published would have implications for names of genera and species that were not applicable to names of families.

**Barrie** agreed as he had the feeling that there might be consequences for species names that were not relevant for family names, but he was not sure, that was why he was asking if anyone had thought about it.

**McNeill** could not envisage it.

**Reveal** suggested that the similar wording “except under the provisions of Art. 14.12” also be added to Prop. D.

**McNeill** thought that he was assuming that would permit names that were not validly published to be retained, just as had been done for family names in App. IIB.

**Turland** queried as to whether Reveal was proposing an amendment to what had already been passed.

**McNeill** thought that Reveal was suggesting this should be entirely parallel to what had already passed, that he wanted clarification as to whether Prop. F included the clarity that Greuter suggested, that it was clear that names that would otherwise be not validly published were treated as such, because this would then also be an exception to Art. 6.

**Turland** confirmed that this was his intent, although he thought that this was covered at the end.

**Knapp** agreed it was included at the end but thought that Reveal was proposing something different: making a new amendment to the proposal that had already passed and she thought that could be dealt with editorially.

**Reveal** was just suggesting that when the Editorial Committee dealt with the two items that they make sure that, if Prop. F was turned down, that the provisions from Art. 14.12 went into Prop. D.

**Norvell** was concerned that correcting a citation in the *Code* for a conserved species name when it was found that an author and date of a work should be changed would no longer be possible. Obviously the place of publication also would be changed.

**McNeill** confirmed that it would mean if an earlier place of publication was found, that would become irrelevant. Whatever was accepted as the place of publication of the conserved name would be retained.

**Norvell** referred to a case where all three items—the authors, the place of publication[, the date]—everything had changed because an earlier combination had been found…

**McNeill** explained that the whole point was to prevent what was perceived by the proposer and by many others to be unnecessary changes. The work had been done in conserving a name. What was really wanted was to be able use the name. It did not really matter where it was first published once it was established that this was a name that was going to be used.

**Norvell** was opposed to the proposal.

**Pennycook** knew of a case of a fungal genus name that had been conserved with a fictitious publication, based on a total misunderstanding of the literature, and wished to know if this would be preserved for all posterity without any opportunity to change it.

**Turland** requested confirmation that the publication did not exist.

**Pennycook** confirmed this.

**Turland** thought that in that case he did not see how it could be protected because it did not exist.

**Sennikov** added that the proposal was very much needed, and it was a good complement to the principle of conservation, because should the authorship or the place of publication be changed to something earlier it would be a totally different name with different original material, probably a different type. He felt the proposal was good even though it might not be fair in all cases to the history of botany, to the earlier authors, so that this history would not be retained; but conservation was not fair to history.

**Redhead** felt that, given that the Appendices in question had not been as thoroughly vetted as the family lists in App. IIB, perhaps the application of the proposal could be delayed, which would allow a few years for people then to go through and vet them and, once they were established, then say “You had your chance”. He proposed an **amendment** to add a date effective from 1 January 2015. [The **amendment** was **seconded**.]

**Turland** suggested 2016. [Laughter.]

**Redhead** reiterated that this would just build in a chance for people to try and correct what was there and motivate them.

**McNeill** commented that there would presumably need to be some mandate for some person or persons to carry out the verification. If there was simply a date and if nobody did any checking then it would automatically come into effect on that date.

**Barrie** pointed out that the list was looked at by the Editorial Committee each time the *Code* was published, but not a thorough, monthslong inspection of each and every entry. He invited people to send corrections in to the Editorial Committee. He felt that the list was reasonably clean, probably not flawless, but not riddled with errors.

**McNeill** thought that one of the things that probably concerned people other than vascular plant taxonomists was that, whereas the whole list of conserved names going back to 1905 was very thoroughly reviewed by Rickett and Stafleu in the ’60s and ’70s, no correspondingly asthorough coverage was carried out for some of the other groups. He thought that Isoviita had done quite a lot for the bryophyte lists at different times and, at the time of the changes to starting-point date, the fungal names were reviewed as well. He agreed that it was something that was continually ongoing, and suggested that the Editorial Committee be charged to take account of any information provided.

**Applequist** wondered how any corrections made after the *Melbourne Code* was published would be inserted into the Appendices before this provision took effect, since the next Congress was not until 2017. She asked whether publication of a list of errors in *Taxon* would suffice.

**McNeill** thought it would depend very much on how the next few proposals were dealt with, as the next two proposals suggested electronic publication of the Appendices.

[The **amendment** was **rejected**.]

**Prop. F** was **rejected**.

**Reveal** requested a point of clarification: inasmuch as the proposal deleted all references to family, he wanted to know if that still applied to Prop. D.

**Knapp** explained that Prop. D had passed.

**McNeill** had thought that Reveal was about to raise the issue that Prop. D include the reference to Art. 14.12.

**Prop. G** (59: 43: 5: 0).

**McNeill** moved on to Art. 14 Prop. G–I, which were seeking a mandate from the Congress to, if necessary, terminate hardcopy publication of App. II–V. He was not sure to what extent the proposer was seeking to preclude inclusion [of these Appendices in the *Code*] or merely to permit it to be done electronically.

**Redhead** did not have much of an agenda regarding the proposal but had just noticed that in carrying this around… [he brandished his copy of the *Code*—Laughter.]

**Knapp** warned him that he was going to break it.

**Redhead** pointed out that the part he was holding in the middle were all the Appendices, and the rest of it was the *Code* itself and the indexes. He could only envisage it getting larger and larger and larger, and particularly with molecular taxonomy research that the Appendices could be cumbersome. He did not know how much it cost for the IAPT to publish the *Code*, but it seemed ridiculous to him in this day and age to be publishing such an immense thing. He was really putting forward this proposal so that everyone could just decide whether they wished to continue spending money or whether it was possible to facilitate it electronically. He did not actually wish to run it—someone else would have to do it—so the idea was just out there to try and save money and make things more efficient.

**McNeill** requested clarification of the phrase “Periodic publication of comprehensive hardcopy of the Appendices may be made”, as he felt that would presumably mean that some *Code* might want to include them, whereas later on the implication seemed to be that the Appendices may not be included with the *Code*.

**Redhead** had been trying to build in flexibility for whatever the Congress decided, but he just wanted to have the opportunity to try and prevent downing of more trees.

**Stuessy** suggested having a two-volume *Code*, the first with all the rules and so on, and the second would be the Appendices, and you could buy one or the other if you wish.

**Prud’homme van Reine** was in favour of a single book with the full *Code*, as he felt that many people would not have access if it were only electronic. He found it better to have a nice book at least once every six years and it did not matter if it was thick or thin, you could have the book on your shelf and you could use it.

**Annette Wilson** spoke to the business of trees by saying that she thought requiring it to be published in *Taxon* would equate to more trees. She did not think that hard copy publication of the amendments and additions was going to actually solve anything. Speaking as an editor, she suggested that making changes to the typefaces and layout would probably cut the Appendix sizes in half. She was not in favour of taking the Appendices out of the *Code*, although she did not see any problem with having it online as well, but she certainly supported editorial manipulation to make them shorter.

**McNeill** assured her that this was already in mind for the next *Code* and had been discussed.

**Greuter** was in favour of the proposal, but there was something in it that was completely inappropriate and unacceptable in his mind, and he hoped it could be taken care of by the Editorial Committee if the Rapporteurs approved. Because the *Code* had authority over names but no authority over either the editorial policy of *Taxon* or of the IAPT, he felt it was inappropriate in the *Code* to put rules on what was published in *Taxon* and supported by IAPT and suggested that this aspect should be neutralized.

**McNeill** thanked Greuter and summarized that he was supporting the principle of separate publication electronically without necessarily endorsing the precise wording of the proposal being made.

**Demoulin** loved the solution that Stuessy had mentioned and he would certainly buy the two copies because it was very useful to be able in some circumstances, like at the Congress, to just to have the rules, but at home he absolutely wanted to have the printed copy of the Appendices. Those who preferred to work on the computer screen could already do it, so he supported continuing to have nice books for important things like this.

**Van Rijckevorsel** thought that there were two separate issues: firstly, how the *Code* should look and secondly if there should be a provision in the *Code* on that. There had never been anything in the *Code* that prescribed how it looked, there was no requirement for hard copy and up until 1983 the *Code* was produced in three languages, although there was no provision for that. Before that, there was a discussion to publish only a supplement to the *Code*, and even before that there was a *Code* that existed only as a supplement. He felt that the issue was whether there should be a provision in the *Code*, and he thought not. Secondly, a suggestion that had not been made was to put in the *Code* only the changes to the Appendices, which would slim it down a lot, and a hard print book would still be available. He opposed the proposal but not the thought.

**McNeill** felt that it sounded from the debate that there was a lot of support for two things. First of all, making it clear that the Appendices were indeed part of the *Code* by some inclusion in the Preamble, and that would come in other proposals before the Section. But more importantly, that the concept of electronic publication of the Appendices, as being a component separate from the main work that should be updated on a regular basis, also appeared to have some support, whereas the actual wording of enshrining this in the *Code* had had some negative comments, which he found justifiable. He suggested taking a vote on the principle behind this, separate from the wording, and then perhaps the proposer could redraft it as he had said he was not too concerned about the exact wording, just the principle.

**Redhead** was basically interested in the principle and was happy to leave it completely in the hands of the Editorial…

**McNeill** interrupted that he thought there were two elements to the principle: the one element was that there should not be hard copy except periodically, whereas the other principle was that there should be electronic copy but the hard copy would continue with each issue, but perhaps in a separate volume. He asked if Redhead was seeking to limit the hard copy production.

**Redhead** said that he was because he felt that it would impede people carrying it [the *Code*] around if it was all bound together, and he also anticipated that there would be an awful lot more conservation proposals in the future to deal with the results of molecular taxonomy and phylogenies, and the Appendices would swell. His proposal was basically a sort of philosophical idea, but the exact wording could be altered: *Taxon* and IAPT could be taken out if it was inappropriate. Essentially it was just an idea that he had thrown out to the Congress to give it an opportunity.

**McNeill** reiterated that there were two issues and suggested that the precise wording was not up for debate. What was being debated was the proposal of Redhead, the principle that the Appendices should not normally be included as hard copy with the *Code*, so the proposal was that in future the Appendices would normally be published electronically and the Editorial Committee was charged to make appropriate reference to this in the *Code*.

**Herendeen** felt that occasionally publishing hard copy of the Appendices, not specifically mentioning *Taxon*, was a good thing; he supported occasional publication where necessary. He believed that it would be very useful to move to electronic publication of the Appendices, remove them from the *Code* itself and if there was a groundswell of support for publishing the Appendices from time to time, then so be it, but as a separate publication.

**Knapp** thought that that was essentially the proposal that McNeill had made, pulling all three proposals together. She pointed out that Prop. I was to basically let the Editorial Committee decide what the wording should be. She interpreted what people had said to mean that what needed to be decided on was whether the Appendices should be largely electronic and occasionally published in hard copy or whether they would always be hard copy and published at the same time as the *Code* every six years. She also mentioned the possibility of publishing the Appendices as a separate volume, but highlighted that this was not really something for a Section to decide.

**McNeill** felt that was what was needed was the authority not to publish it with the hard copy of the *Code*.

**Stuessy** suggested that the discussion was off track a little. He emphasized the desire to have the *Code* published in whatever form possible to ensure as full a dissemination of the *Code* as possible. He supported publishing the *Code* (with Appendices) in both hard copy and electronic versions.

**Kirk** felt that Redhead’s point about greening the *Code* and not chopping down trees missed the point of the cost of shipping those bits of trees around the planet. He suggested that the way to solve that problem was an electronic version with the ability to download a PDF in page format to print locally, which should be very simple from an IT point of view. This would mean that every six years when it was updated people who could not afford the book and did not want to increase CO_2_ in the atmosphere by shipping a book around the planet could print it locally at local costs, which would appeal to people in developing countries.

**Sennikov** added that those who argued for the nice book and the availability of that book maybe did not realize that the cost of the nice book was still high enough that many people hesitated to buy it. He also felt that the market of the *Code* was such that it was sold from few places, not in many countries, and often had to be purchased from abroad, which was not very convenient for many people actually in the Third World, Speaking for Russian taxonomists, he knew that very few taxonomists in Russia had an original copy of the *Code*. He maintained that the major availability of the *Code* was exclusively because it was available on the Internet. He argued that it must be available electronically, and deciding to save paper resulting in trees being not chopped was a nice thing but most importantly that electronic availability of the *Code* was a really great thing that helped people.

**Gandhi** had a minor comment regarding the term “periodic publication”, as he felt it may require a definition. Periodic, in the sense of what? Once in 10 years, once in…

**Knapp** refocused the discussion on the principle rather than the wording.

**Glen** as somebody working in a developing country had not used a paper copy of the *Code* in the last six years plus. His unit did not even own a copy of the *Vienna Code* on paper, they only used the electronic copy, which was wonderful particularly because it was searchable, which meant he could find what he was looking for. [Laughter.] Glen requested that whatever was decided, it should be published electronically. He felt that if somebody was really that addicted to ink on paper they could always print it out at a printondemand place and get it bound. He supported this proposal and added that the lists could be updated every five minutes if desired; there was no need to wait for six years for the new one.

**Miller** thought having the *Code* and its Appendices available electronically was a wonderful goal, but there was no need to amend the *Code* to do that. He interpreted the proposal as addressing the question of whether to separate the Appendices from the printed copy or not, and he did not want that done.

**McNeill** agreed that was a very good point. He emphasized that what this proposal was permitting the Editorial Committee to do was to cease to publish the Appendices with the *Code*. All the other things mentioned could already be done and some had been done in the past, as had been pointed out.

**Knapp** exercised her Chair’s prerogative to suggest moving to a vote on this proposal. She reiterated that voting “yes” for this proposal would allow the Editorial Committee to decide to not print the Appendices as part of the *Code* and to have them as solely electronic copy while voting “no” would mean that the printed Appendices were always part of the printed *Code*.

**McNeill** added that voting “yes” did not mean it would not be possible to have a separate printed volume of the Appendices. He explained that, as it had been rephrased it was a procedural motion as it no longer amended the *Code*, so the vote required only 50%.

[The **motion** was **approved**.]

## Fourth session

Tuesday, 19th July 2011, 13:30–18:00

### Article 14 (continued)

**Knapp** decided to exercise her Chair’s prerogative [again] to revisit something that had happened before lunch. She thought that there was a bit of ambiguity about a vote, and perhaps a perception that there might be ambiguity about what type of majority was needed to do it. What she thought had been voted on—and the vote would be taken again—was that the Editorial Committee had the option to produce the Appendices in only electronic form.

**Nic Lughadha** had understood that the Editorial Committee had the option to publish a hard copy of the *Code*, without the Appendices being included.

**Knapp** thought that sounded like the same thing, that the Appendices would be electronic and only the *Code* would be in hard copy. She pointed out that there was no specification in the *Code* that the *Code* be hard copy. The proposal was from Redhead and he had said he was happy for anybody to do whatever they wanted really, which made things a bit complicated. As the proposal did not involve an amendment to the wording of the *Code*, it was felt that a 50% simple majority was the correct majority for the vote. It could be so interpreted that this would cause a change in the physical manifestation of the *Code* and therefore a supermajority would be required. She asked the Section to choose between the two options of simple majority or a supermajority for voting on the issue. The vote itself would be a 50% majority vote one way or the other, because it was a choice between two options. Then the Section would move to a card vote, as was suggested by several people around the room, on “The Editorial Committee has the option to produce Appendices in only electronic form”.

**Funk** was under the impression that the Section was empowering the Editorial Committee to decide how they wanted to deal with the Appendices, and one option was that they were published electronically, but this did not prohibit the option that it could be hard copy.

**Knapp** agreed that this was what the word “option” meant.

**Funk** objected to the use of “only”.

**Knapp** suggested deletion of the word “only”, so the proposal would become “The Editorial Committee has the option to produce the Appendices in electronic form”. She moved to the first vote, a simple majority vote as to whether 50%, a simple majority was needed for this, or a supermajority. [A 50% simple majority was **accepted**.] She moved on to the card vote.

**Annette Wilson** wanted to know if the Section was still voting on Prop. G or not.

**Knapp** clarified that it was not a vote on Prop. G. She outlined what the Section would be voting on. “The Editorial Committee has the option to produce the Appendices to the *Code* in electronic form”. The vote was a simple majority vote card vote. A “yes” vote meant that the Editorial Committee would have the option to produce the Appendices to the *Code*…

**Ulloa** still did not understand what was being voted on, she wished to know if an amendment to Prop. G had been proposed from the floor.

**Knapp** explained that because of the way Prop. G, H, and I were presented and the way in which they were discussed, when the Section voted before lunch it voted on the principle that the Editorial Committee would have the option of producing the Appendices in electronic form—a principle, not something that would go into the wording of the *Code*. It was an instruction to the Editorial Committee for how they would perhaps work on producing the Appendices.

**Ulloa** wondered if it was legal to vote on a principle that was not written.

**Knapp** pointed out that the *Code* was not a legal document but a code of practice.

**McNeill** clarified that it was a guidance to the Editorial Committee and that was the reason why it was a simple 50% majority. It was not an amendment of the *Code* but it was very cognate to the *Code* and it was perfectly proper for the Section to give instruction to the Editorial Committee as to what options they would like the Editorial Committee to have regarding the form of the *Melbourne Code*. He highlighted that it was not binding because if there was some financial crisis in IAPT and it was not possible to publish the *Code*, then it would not be published. The vote was a guide, an intent on the will of this Section as far as the production of the *Melbourne Code* was concerned. He felt that this was very valuable to have, and was certainly most relevant to the work of the Section.

**Knapp** thought it gave the Editorial Committee guidance to what might be done in the future.

**Redhead** was the person who proposed these things, and he had published them as proposals to change the *Code* because it added substance and got it out there for debate, but he completely agreed with what was taking place now and it was just principle, so the Section was not specifically voting on Prop. H or I or J. It was the principle that was instilled in that.

**Buck** wondered what would be added by voting on this because the Appendices as well as the entire *Code* were already online electronically.

**Knapp** understood what he was saying but still felt that there was a certain amount of misunderstanding about what was being voted on, and what percentage, and perhaps some disquiet about what percentage should have been used to vote. So she was suggesting that everything be very, very clear and open. She felt that it did not matter what it added; what it allowed was some latitude for the Editorial Committee to have the option to produce the Appendices in electronic form.

**Buck** reiterated that they already had that option and had done it. He likened it to closing the barn door after the horse had left.

**McNeill** added that there was a situation in which since de Candolle’s *Lois* there had been a printed copy of the *Code*, and as soon there became Appendices these were published with the *Code*. What this was saying was that the Editorial Committee was free to publish both in hard copy; there was a clear guidance, if the vote was “yes”, that electronic version of the Appendices in particular would be what people would like to see become established, but that there was no longer the requirement that we produce a printed copy of the *Code* with the Appendices.

It was quite clear to him that a vote “yes” merely gave the Editorial Committee the authority to do what had been done before, but given the initial proposal, a vote “no” would be a much firmer instruction because that would tell us “you’ve got to do what you’ve always done and you’re not allowed to separate the Appendices from the body of the *Code*”. It was to allow the flexibility to move more strongly into the electronic direction, always recognizing that there had to be a final copy.

There would always have to be a definitive copy, and what type of electronic storage that would be in, what format, would have to be negotiated in due course. It could well be that in this instance, for the *Melbourne Code*, there might be a printed copy of the Appendices as the archival copy of the main copy, but in the future it would be a more permanent electronic storage that might be the archival reference copy.

**Levin** was, quite frankly, getting more and more confused. He requested clarification on whether voting “no”, would mean that the Editorial Committee does not have the option of making an electronic version at all, because that was the implication as it was presented.

**Knapp** clarified that if the Section voted “no”, it meant that however the *Code* produced, the Appendices were also produced in the same way, the Appendices would stay as part of the *Code*.

**Levin** suggested clarifying that instead of giving the Editorial Committee the option to produce the Appendices electronically, they have the option to publish them separately as he felt that that was the core of the issue.

**Knapp** repeated that the option already existed. She summarized again that a “yes” vote meant that the Editorial Committee would have the option to pursue different things, like producing two volumes as suggested, a “no” vote meant sticking with what had been done and not pursuing alternatives. It was a principle, so the words were not actually that important. “The Editorial Committee has the option to produce the Appendices in electronic form”.

**Thiele** suggested clarifying these confusions by saying that we were voting that “The Editorial Committee has the option to produce the Appendices to the *Code* in electronic form only”.

**Knapp** pointed out that that was what she had initially suggested and it was amended by someone to take the word “only” out.

**Thiele** felt that in the conversation that had just occurred that that was where the confusion lay. Of course the Editorial Committee currently had the option to produce the Appendices electronically but what was actually being talked about was whether the Section was empowering the Editorial Committee to have the option to not produce them in hard-copy form, to have it electronic only.

**McNeill** thought that was probably the thrust of Redhead’s proposal.

**Redhead** concurred.

**McNeill** requested the proposer to comment on whether that encapsulated the principle that he had in mind. [It did.]

**Knapp** recapped that the vote was about an option, a “yes” vote meant exploring the option of producing the Appendices in electronic form only, and a “no” vote meant not.

**Kellermann** thought that there were two different points. Firstly, in electronic form only; secondly, separately from the main part of the *Code*, so whether the Appendices were published at the same time or at different times…

**Knapp** interrupted very politely to say that there already was the option to produce them separately.

**Kellermann** added that the option to produce them electronically existed as well.

**Knapp** disagreed.

**Kellermann** said it was so.

**Knapp** clarified—not “only”. She reiterated that the vote was looking for guidance for the Editorial Committee and would not be written in the *Code*.

**Kellermann** still thought that people were talking about the two issues and either mixing them up or talking about them at the same time.

**Knapp** agreed.

**McNeill** suggested going with what had been suggested, provided that that was what the initial proposer felt represented the principle behind his proposal. He agreed with comments from the floor that the detail in the original proposals was inappropriate, but the principle of moving towards greater electronic publication of the *Code* was very desirable and in particular the electronic publication of the Appendices. The Editorial Committee had freedom, but IAPT in particular could not necessarily provide the funds to do different things, so the *Code* may be constrained to operate in a particular way.

However, he felt that it was important to do what the botanical community wanted to have done, and really that was what the vote was about; to get a feeling from the Section as to what strategy—if economically and in other ways feasible—should be adopted for the publication of the *Melbourne Code*.

Many people felt like Scott did, that the sheer bulk of the Appendices in the *Vienna Code*, even if by better editorial production they could be more concise, was still excessive and would increase. Therefore, to split them off and to perhaps have the Appendices entirely electronic was a good idea, but many people have said they like this nice book and they would like to keep it as it always was and thus guidance was necessary.

There was no way to force the Editorial Committee or IAPT to do something, but a very strong indication of the way in which the publication of the *Melbourne Code* should proceed could be given. He was quite happy with the more general broad principle that was talked about before lunch and was supported, but if it was deemed important to get a card vote then some words were needed. He thought that would give the indication of where the Section stood vis-à-vis electronic publication as an option, or insisting on hard copy.

**Knapp** asked the original proposer if he wanted the word “only” in or not.

**Redhead** suggested that the word “option” meant that either one or the other could be done and decided that taking out the word “only” offered more flexibility.

[Aside discussion and objections.]

**Redhead** was also fine with leaving the word “only” in there.

**McNeill** was of the opinion that it would be meaningless without “only”.

**Knapp** exercised the Chair’s prerogative again, deciding to leave the word “only” in. She clearly outlined that a “yes” vote would imply that the Editorial Committee would have the option to produce the Appendices in electronic form only, and a “no” vote would mean that they did not. She moved to a card vote on the issue.

[Knapp thanked the tellers for taking all the card votes and, while they counted, the Section moved on to the next items of business.]

**Prop. G** was voted on in principle and **accepted** in a card vote (368: 157; 70% in favour).

**Prop. H** (60: 35: 9: 0) and **I** (57: 34: 17: 0) were referred to the **Editorial Committee**.

**Knapp** offered reassurance that although the Section had given instructions to the Editorial Committee to explore the issue, it did not mean there would not be hard copy of the Appendices of the *Melbourne Code*.

[*The following debate, pertaining to a new proposal by Hawksworth to amend Art. 14, took place during the Tenth Session on Friday afternoon.*]

**Hawksworth’s proposal**

**McNeill** introduced a proposal from the floor from **Hawksworth** on Art. 14.1 He explained that conservation of names was only permissible at the ranks of family, genus, and species, and this was a proposal to extend it to all ranks. [The **proposal** was **seconded** and **supported** by three others.]

**Gereau** felt that the Section was quickly becoming a race of lawyers rather than scientists, making rules and going through lists, and on every issue micro-legislating all activities, instead of applying sets of basic principles so that it was possible to just get on with our work. He wondered how many Appendices there would be. He wanted to know if there would be an App. VIII on conserved and rejected names of subtribes and an App. IX on conserved and rejected names of subformae. He felt it was becoming absurd and would lead to a *Code* in six volumes in little time if the trend continued. He argued that it was time to get rid of this once and for all. [Applause.]

**Knapp** instructed the Section fiercely that there was to be “No clapping!”

**Talent** had a problem with giving special status to subspecies when variety was the common rank in many groups.

**Funk** called the question. [There was a sufficient majority in favour of voting.]

**Hawksworth’s proposal** was **rejected**.

[*Here the record reverts to the normal sequence of events.*]

[*New proposals concerning Art. 14 were included as part of Redhead’s set of proposals relating to fungi with a pleomorphic life cycle, and that discussion can be found under the Eighth Session on Thursday afternoon; there was also Lendemer’s proposal as a consequence of the other changes accepted, and that discussion can be found under the Tenth Session on Friday afternoon.*]

### Article 15

**Prop. A** (80: 5: 8: 6).

**McNeill** introduced the next item of business, which was Art. 15 Prop. A by Demoulin, adding that sanctioned names had special status similar to that of conserved names, and the proposal was that the spelling used by a sanctioning author was treated as conserved except if it was to be corrected or standardized under Art. 60. The proposal was quite independent of the sanctioned names discussion so did not need to be deferred. It was to make more explicit what many people assumed was the case.

**Demoulin** confirmed that the proposal was just to make clear something that had always been the policy of the Fungal Committee, but also add something that needed to be spelled out that Luis Pérez had suggested to him: that sanctioned names could be corrected under Art. 60. The good support in the mail vote made him think that it was not necessary to elaborate much.

**McNeill** agreed that both the mail vote and the opinion of the Nomenclature Committee for Fungi were very positive with regard to the proposal.

**Prop. A** was **accepted**.

**Prop. B** (38: 23: 28: 8) and **C** (34: 40: 12: 12) were **withdrawn**.

### Article 16

**Prop. A** (87: 12: 7: 0).

**McNeill** moved on to Art. 16 Prop. A, which was also proposed by Redhead although quite independent. This was to deal with a slight anomaly in the sense that any name above the rank of family could be treated as a descriptive name, so that even if it was not based on an included legitimate family name it could still be legitimate, but then the type was open to question. The proposal offered a mechanism for slightly broadening the basis of names above the rank of family that were derived from a generic name in order to make them more frequently legitimate. The Rapporteur had commented that it would clarify that typification.

**Redhead** explained that one of the reasons for his making the proposal was that, due to more modern phylogenetic research, taxonomists were now approaching things from the opposite end to traditional taxonomy, which used to build things up from species to genera and then to family and so on. He noted that people were now approaching it from kingdom on down and were proposing new orders and forgetting the fact that there were legitimate names of families or other higher levels, and yet they seemed to be based on generic names, so this was an attempt to standardize the automatic typification of names that were obviously based on generic names but at a higher level so they skipped one or two ranks.

**McNeill** noted that the proposal had good support, 87 to 12 in the preliminary mail vote.

**Greuter** favoured the proposal in principle, but was not satisfied with the way it was presented. On the one hand it had a drawback in that it was ambiguous. He wondered what would happen if a suprafamilial name that included the type of a legitimate name of a family was nevertheless called after a genus that was not the type of the family as this would create an ambiguous situation. He felt that the proposal was just too complicated.

He suggested a friendly amendment, that the proposal could be reworded and simplified by just substituting what was now under (a), automatically typified names formed from the legitimate name of an included genus by adding to the genitive singular inflection the appropriate termination. The exact wording would then be parallel to what was in 18.1 for family names. It was rather lengthy but would be taken care of by the Editorial Committee. He pointed out that the simplification would also have the effect of making Prop. E, to which the Rapporteurs had rightly objected but which had quite some merit, acceptable.

[The amendment read: “(a) automatically typified names, formed from the genitive singular of a name of an included genus by replacing the genitive singular inflection (Latin *ae*, *i*, *us*, *is*; transliterated Greek *ou*, *os*, *es*, *as*, or *ous*, and its equivalent *eos*) with the appropriate termination; or (b)”.]

**McNeill** summarized that instead of determining legitimacy of a name above the rank of family on the basis of an included legitimate family name, Greuter was proposing bypassing that rank and determining the legitimacy of a name above the rank of family on the name of an included genus.

**Greuter** had suggested the amendment on the understanding that the effect would be exactly what Redhead desired, but it would be simpler and would not have ambiguity included.

**McNeill** could not immediately remember it, but he had a funny feeling that there was a further Article that depended on family names as being the basis.

**Redhead** assessed the amendment as still allowing a bit of flexibility, whereas his intent was to ensure that those names that were based on generic names were automatically typified. The way he read the changes, it appeared that such names may be either automatically typified names or they could still be called descriptive names. He felt it did not pin it down.

**Greuter** added that under (b) it said “descriptive names not so formed”, so if they were formed from a genitive singular of a generic name they were not descriptive names.

**Redhead** agreed that if that was the way other people read it that would be acceptable because that was the intent. He took a minute to decide whether the meaning of the amendment was what he intended and then accepted it as a friendly amendment.

**Reveal** wondered why anyone would have an ordinal name without an included family.

**Redhead** responded that he would not but that there was a growing group of people who were publishing order names in the absence of family names and, possibly, other novel names because they were using phylogenetic analysis and working the way down from kingdom, not grouping species or genera together.

**Malécot** made an editorial comment on the amendment, that, instead of “with the appropriate termination”, the proposed amendment should be “with the appropriate termination denoting their rank (preceded by the connecting vowel o- if the termination begins with a consonant) as specified in Rec. 16A.1–3 and Art. 17.1”.

**Reveal** encouraged the Section to vote “no” as he felt the proposal was encouraging bad taxonomic practice and did not see why it would be desirable to put bad taxonomic practice in the *Code*. He added that it was long known that you have species within genera not species floating so wondered why you would have a floating order without any families, but it may have genera in it.

**Redhead** began to explain that there were examples, particularly in the fungi, when people had published orders…

**Knapp** admonished the microphone runner for supplying Redhead with an unauthorized microphone!

**Redhead** continued …and afterwards published families.

**McNeill** commented that there was a world of difference between the need for a genus and a generic name for any species and the higher category. It was conventional that the family had become very important in certain groups of organisms, most notably the vascular plants, but there was no reason why it was necessary to have entries in all the other higher categories.

**Knapp** concluded that there was a clear difference of opinion and moved to a vote.

**Prop. A** was **accepted** as amended.

**Prop. B** (30: 4: 71: 0) was **withdrawn**.

**Prop. C** (28: 4: 73: 0) was referred to the **Editorial Committee**.

**Prop. D** (36: 42: 24: 0).

**McNeill** noted that this proposal was defeated 36 in favour to 42 against in the mail vote. He added that it was making explicit what was otherwise quite clear in the *Code*—that autonyms do not exist above the rank of family. He felt that the proposal may be seen to be useful, but it was not in necessary because autonyms were defined in the *Code* very specifically in terms of the ranks in which they occur and they were not present above the rank of genus.

**Prop. D** was **rejected**.

**Prop. E** (15: 87: 6: 0).

**McNeill** mentioned that Prop. E had more than 75% “no” votes, but that there was some suggestion that someone might want to raise discussion on it.

**Greuter** had foreshadowed the issue when speaking on Prop. A, and felt that the “no” votes were completely justified in view of the also justified negative comments of the Rapporteurs, which had however become irrelevant because of the changed format in which Prop. A had been voted on. He therefore proposed that the Section discuss the proposal. [This was **seconded** and supported by three others.] He continued that the merits of the proposal were that names of orders and suborders that were descriptive names were not only dropping out of use, but that those that were not were a nuisance. He gave the example of *Leguminales*, which was not based on a generic name but was sometimes used and would be outlawed by the new provision, and others, like *Liliiflorae* or *Glumiflorae*, which were heritage of the past from the old Engler system or the Principles of Linnaeus. He felt that these descriptive names that had been used at those ranks were no longer in use, so it was no worry to lose them formally, which was why he quite liked the idea of having the proposal accepted.

**McNeill** made two points: Greuter had rightly commented that the main opposition that the Rapporteurs drew to the proposal had been dealt with as a result of the acceptance of the earlier proposal; however, the second reason against the proposal was still applicable—why it was thought necessary to rule out the possibility of using these names when in fact they were already becoming less used. A point he felt was more important, advised by mycologists to some extent, was whether this would make names like *Ascomycota* and *Basidiomycota* not validly published.

**Greuter** pointed out that the proposal dealt with names of orders and suborders, not all names above the rank of family.

**McNeill** apologized as he had thought it was all names above the rank of family.

**Demoulin** had already mentioned that he taught botany to people who were not botanists and found descriptive names were extremely useful in this context. He used *Leguminales* and hoped his successor would keep doing that. He felt that in a number of situations it was much better to have something descriptive, whatever its rank, because then “you know what you’re talking about”. He expressed surprise that Greuter was now against descriptive names because he always remembered what he had told him one day, that “with a name like *Ascomycetes* you know what you’re talking about but when you’ve got something like *Pezizomycetes* you never know how inclusive it is”.

**Prop. E** was **rejected**.

**Prop. F** (22: 13: *72: 1).

**McNeill** moved on to Prop. F, which was substantially editorial. He outlined that there were a number of cases in the *Code* in which Articles that were mandatory or rules that had mandatory effect were represented by text that was in Recommendations, and this was one of them. The suggestion was to move this from a Recommendation into an Article of the *Code*, which would not change the application of it. He mentioned that it was referred strongly to the Editorial Committee in the mail vote.

**Wiersema** did not think that the proposal was entirely editorial because Brummitt had dropped the last sentence out of the existing Article, under the premise that his other proposals on valid publication of suprafamilial names would be accepted. He also mentioned a housekeeping detail that would delete Rec. 16A.1, 16A.2 and 16A.3, not just Rec. 16A.1.

**McNeill** agreed that the first point had been made by the Rapporteurs, that those who favoured the proposal but recognized that the precise wording was dependent on the acceptance of subsequent proposals if those were rejected, should vote “ed. c.”. In other words, that that sentence would not be removed in this situation. He also agreed that the housekeeping would certainly be editorial.

**Unknown speaker** wondered if the Section was allowed to vote to the Editorial Committee.

**McNeill** explained that in the mail vote, in order to allow an expression of the desire for a principle with some modification, the convention of referring to the Editorial Committee was established. He went on to say that the Editorial Committee will automatically look at those components where the proposer had suggested removing some Recommendations but not all that were needed, but the Section as a group had to vote for the proposal as now modified by retaining portions that the proposer wished to omit because of a presumption of crossing something out.

**Knapp** introduced the vote for Prop. F in the way that McNeill just described, which she felt she could not possibly repeat.

**Prop. F** was **accepted**.

**McNeill** noted that Prop. G in Art. 16 related to quite an extensive set of proposals, which were really interesting but unusual. He suggested that, as the core of the proposal set appeared in Art. 32, it would be better to consider this along with the others with Art. 32.

**Prop. G** (30: 62: 13: 0) was ruled **rejected** as it was a corollary to Art. 32 Prop. A, which was rejected.

**Van Rijckevorsel’s proposal**

[The proposal was submitted electronically and read:

“*Note n*. Given that names above the rank of family are often used by different authors for taxa with quite different circumscriptions, it often is desirable to indicate in what sense the name is used.

*Ex. n*. Differently circumscribed taxa which may be indicated by the same name, and which may be recognized by citing the author(s) who circumscribed the taxon: *Magnoliidae* sensu Dahlgren, 1980 [= dicotyledons]; *Magnoliidae* sensu Cronquist, 1981 [= basal angiosperms]; *Magnoliidae* sensu Chase & Reveal, 2009 [= angiosperms]; *Magnoliidae* sensu Stuessy, 2010 [= magnoliids].”].

**Van Rijckevorsel** wished to put forward a new proposal. He quite liked the basic premise of Brummitt’s piece, which was that names above the rank of family were often very different in circumscription depending on the author who used them. As circumscriptions differed very strongly he felt it would be useful to put a Note and an Example to that effect in Art. 16. [The **proposal** was **seconded** and **supported** by three others.]

**Gereau** pointed out that it was already possible to indicate the circumscription in which the use of the name was understood by saying “*sensu* some author”. He saw absolutely no need to write it into the *Code* as it was already common practice, well understood and it operated at all ranks. It had nothing to do with being above the rank of family, so he thought the proposal was trivial and should be rejected.

**McNeill** noted that it would actually be a Recommendation rather than a Note and that could be above the rank of family.

**Van Rijckevorsel’s proposal** was **rejected**.

### Recommendation 16A

**Prop. A** (83: 17: 7: 4).

**McNeill** outlined that the proposal was, in addition to having the endings *phyta* and *mycota*, that *phycota* should be included in Rec. 16A. He reported that the Rapporteurs thought it desirable and the mail vote was 83 to 17 in favour.

**Prop. A** was **accepted**.

### Recommendation 16B

**Prop. A** (47: 55: 8: 0) was ruled **rejected** as it was a corollary to Art. 32 Prop. A, which was rejected.

### Article 18

**Prop. A** (10: 89: 9: 0) was ruled **rejected**.

**Prop. B** (8: 9: 91: 0).

**McNeill** noted that Art. 18 Prop. B was substantially editorial, offering a clear and more structured wording for a rather lengthy Art. 18.1, and could be referred to the Editorial Committee. He reported that that was generally the feeling of the mail vote, which was 91 votes in favour of referral to the Editorial Committee. It involved some wording from Prop. A, which had just been rejected, but he assured the Section that that would be taken care of by the Editorial Committee.

**Prop. B** was referred to the **Editorial Committee**.

**Prop. C** (37: 48: 20: 0).

**McNeill** noted that Prop. C paralleled one that had already been rejected, under Art. 16 Prop. D.

**Reveal** reminded members of the Section that autonyms were not permitted above the rank of genus.

**McNeill** agreed that they were not allowed because there was no provision in the *Code* to use them, so they did not exist.

**Prop. C** was **rejected**.

**Prop. D** (11: 75: 21: 0).

**McNeill** noted that the proposal had received quite a substantial “no” vote, 11 for and 75 against, in the mail vote. It was proposing to move into the body of the *Code* material from the introduction to App. IIB and, as the Appendices were indeed part of the *Code*, he suggested that it might be unnecessary. The Rapporteurs had suggested that, if it was deemed desirable to move it, the placement was inappropriate, but that was an editorial matter and need not affect discussion.

[Aside discussion about memory sticks, file names and there being no need to panic.]

**McNeill** continued that the issue was that, whereas the family names in IIB were not conserved against any other particular name, there were situations in which some of these names competed, and there the process of conservation was essentially the same as would be the case for other names where they were conserved against a particular name. Just the method by which this was indicated in App. IIB was slightly different, in the sense that there was a note under the two names indicating which one was to be given precedence.

**Alvarado** felt that, as the Appendices would no longer be together with the *Code*, at least in the printed form, it would be a good idea to give some information about the Appendices within the printed *Code*.

**Prop. D** was **rejected**.

**Reveal** wondered how the Editorial Committee proposed to handle the situation where a person might have a small copy of the *Code* and then an independently published, or perhaps only electronically published, Appendix with the information only there.

**McNeill** acknowledged that this was a real issue and thought that the simplest route given the vote “no” would be to reprint the introduction to the Appendices as an appendix to the main body. But he noted that this was something that could be looked at and it was just an off-the-top-of-his-head response.

**Reveal** felt that the Section had just voted that very idea down.

**McNeill** disagreed, explaining that what the Section had voted down was the inclusion in Art. 18 of that particular clause in a particular place. If it were felt that there was information that would be lost by the Appendices being in a separate work he suggested one possibility could be to reprint what appeared as the introduction as a small appendix to the main body.

**Knapp** welcomed everybody back after afternoon tea with a couple of little announcements. She reported that Larry Dorr had fortunately finally arrived after a rather epic journey, and he had brought the nice copy of the *in memoriam* book, which he and Dan Nicolson had prepared. She thanked Larry for the much nicer bound copy than the kind of rather lame printout that she had produced on the first day and let the Section know that it would be on the registration desk for people to look at and asked that any additions be sent to the e-mail address in the introduction at the Hunt Botanical Institute.

**Prop. E** (31: 83: 2: 0).

**McNeill** noted that Prop. E was the core part of a package of three proposals [Art. 18 Prop. E–G]. He reported that they had received rather negative votes in the mail vote: 72% “no”; 72% “no”; 71% “no”, but as they were less than 75% they were open to discussion. He summarized that the proposal was essentially a mechanism by which the names of long standing, *Compositae, Gramineae, Leguminosae* etc., would no longer be permitted. He highlighted that the mechanism proposed was quite elegant, in that it retained the priority that rested in names such as *Gramineae* and *Umbelliferae* and conferred this onto *Poaceae* and *Apiaceae*, priority that they would not otherwise have had. So he felt that it was workable. He explained further that the proposal was to ban the use of the names of long standing, as opposed to the situation where either was allowed and in which there was in many parts of the world a great tendency to form names on the basis of the genus that included the type, therefore *Poaceae* or *Brassicaceae* rather than the names of long standing. The proposal intended to ban those names of long standing and to stick with the *aceae* alternatives rather than permit gradual atrophy or an ability to choose.

**Gereau** invoked Principle IV of the *Code*: “Each taxonomic group with a particular circumscription, position and rank can bear only one correct name, the earliest that is in accordance with the Rules” and then what he felt was a weak little “except in specified cases”. He felt that the Section really needed to ask, why specify these cases? He recounted that every time he taught botany in Africa, students asked him “You told us groups can only have one name, but why were there two names for this?”. He said “Well, because these were all traditional names that we keep around because of tradition”. They said “Why don’t you get rid of them?”. He said “We keep trying”. He argued that it was desirable to have monophyletic families and wondered why it was not possible to make them mononomic as well.

**Alvarado** thought that it was important to keep names like *Gramineae* because there have been more than 200 years of botanical nomenclature in which those family names have been preserved. He worried that generations in the future would not understand clearly what was meant when they read the works of the past. He felt it was important to keep the two variants. He also made the point that the golden age of alpha-taxonomy happened during the time when the names in question were used and therefore thought it would be better to retain them.

**Demoulin** was surprised the proposal had been rejected with such a wide majority but felt it should still be discussed. He had just observed the beautiful big book named *Compositae* [edited by Funk & al., published by IAPT in 2009] during the tea break, which meant it was not only the time of the ancient people that used the alternative names.

**Funk** pointed out that over 70% of the mail vote went against the proposal. She felt that meant most people who taught liked the names and wanted to keep them and she did not think it was up to the Section to shove such a change down the throats of people who did not want it. Personally she also thought it made a great exam question for taxonomy! She warned that it would be extremely confusing to get rid of at least two of the names, the *Leguminosae* and the *Compositae*, because they were frequently used. Especially in the legumes because it really was confusing in that family without a *Leguminosae* to clearly indicate all three of the subfamilies. She used *Compositae* frequently, never using *Asteraceae*, and she felt there were a lot of taxonomists who liked the name *Compositae* as it was descriptive and easier to explain to a student than *Asteraceae*.

**Sennikov** added his opinion as someone who had worked with *Asteraceae* for 25 years and never used *Compositae* instead. He did not see any harm for the two variants to be legally allowed and made a plea to leave people to choose between them. He understood that it was highly convenient, for example, for databasing to have a single variant and to have standardized endings. He suggested allowing that practice make the choice.

**Lewis** thanked Funk and fully supported *Compositae* as well. He continued in a “beancentric” vein for a minute, outlining that it had always been argued that *Leguminosae* was a preferable name to the alternative *Fabaceae* for the reason that *Fabaceae* was very ambiguous. He explained that when people referred to the legumes, there were three families—*Papilionaceae* was one of them, but the alternative was *Fabaceae*. When they refer to those three units together, an alternative of *Leguminosae* is again *Fabaceae*. So *Fabaceae* was ambiguous and *Leguminosae* was not. Until everybody started writing their floras with one family only, he contended that *Leguminosae* was a better term.

**Prop. E** was **rejected**.

**Funk** exclaimed “ Hot damn!” [Laughter.]

**McNeill** noted that the next two proposals were essentially automatically defeated, in the sense that they were totally dependent on the Section having passed Prop. E.

**Prop. F** (30: 84: 2: 0) and **G** (31: 83: 3: 0) were ruled **rejected** as they were corollaries to Art. 18 Prop. E, which was rejected.

[*A new proposal concerning Art. 18 was included as part of Wiersema’s set of proposals relating to illegitimate family names, and the discussion can be found under the Tenth Session on Friday afternoon.*]

### Article 19

**Prop. A** (70: 27: 5: 0).

**McNeill** introduced Prop. A as dealing with the issue that, as the *Code* was currently structured, it was possible for the correct name of a subdivision of a family to be based on different generic names depending on its rank, e.g. that of subfamily or tribe, according to priority. The proposal was a mechanism to try to minimize, if not totally abolish, the issue utilizing the list of conserved names. He noted that it was helpful for spermatophytes if the name of a subdivision of a family that included the type of a name listed in App. IIB was conserved against all unlisted names. In other words, that meant that, as in the Example [of Prop. B], in *Rosaceae* the name of the subfamily that included *Malus* would be *Maloideae* because *Malaceae* was the conserved name, even though *Pyroideae* was an earlier name than *Maloideae*. He summarized that it would mean that also the tribal name would be based on the same generic name. He concluded that it seemed to be quite a sensible proposal and added that proposals B and C were Examples.

**Prop. A** was **accepted**.

**Prop. B** (51: 28: 22: 0) and **C** (52: 29: 23: 0) were ruled referred to the **Editorial Committee**.

**Talent** made the suggestion that the Editorial Committee could consider adding *Spiraeoideae* to the Example in Prop. B, as the fact that *Maloideae* had been subsumed into *Spiraeoideae* was more of an issue than *Pyroideae*.

**McNeill** thought that would be very helpful and requested that she send a note about the addition by e-mail.

**Prop. D** (38: 66: 3: 1).

**McNeill** moved on to Prop. D, which he described as a close corollary to what had just been defeated with regard to abandoning names like *Compositae* and *Leguminosae*. The proposal would just preclude the use of *Papilionoideae* as a subfamily name, so technically it could be considered. He pointed out that as the Section had defeated the other proposal it would be a little anomalous to support this one.

**Demoulin** reminisced that it was this point that, at the Sydney Congress, prompted Meikle to say that it was one of the small peculiarities that made the *Code* interesting and enjoyable.

**Knapp** felt that was a very helpful comment.

**Prop. D** was **rejected**.

**Prop. E** (21: 62: 25: 0) was ruled **rejected** as it was a corollary to Art. 18 Prop. E and Art. 19 Prop. D, which had been rejected.

[*A new proposal concerning Art. 19 was included as part of Wiersema’s set of proposals relating to illegitimate family names, and the discussion can be found under the Tenth Session on Friday afternoon.*]

### Article 20

**Prop. A** (8: 87: 17: 1) was ruled **rejected**.

**Prop. B** (7: 82: 23: 1) was ruled **rejected** as it was an Example relevant to Art. 20 Prop. A, which was rejected.

### Article 22

**Prop. A** (18: 89: 3: 1) was ruled **rejected**.

**Prop. B** (25: 10: 74: 0) was ruled referred to the **Editorial Committee**.

### Article 23

**Prop. A** (32: 56: 17: 0).

**McNeill** moved to Art. 23 Prop. A, which was the matter of which epithets were declinable and which were not. He did not think that the proposer was present. He explained that the *Code*, very wisely he felt, had a number of examples of names such as those ending in *cola*, which people might think you should decline but were in fact nouns in apposition. The proposal was trying to expand this and make clear those that were adjectival and those that were substantive.

**Gereau** felt that the proposal suffered from being both overly prescriptive and partly wrong. He continued that in all botanical tradition *cola* was always treated as a noun in apposition. To his knowledge *fuga* had always been declined as an adjective as in *febrifugum*. He did not know for sure about *gena* but there were a number of linguistic errors included and he strongly felt that the excessive prescriptiveness of it was really not desirable.

**Challis** agreed and added, regarding the epithets ending in *gena*, that they had been used as nouns but had also been used often as adjectives.

**Harley** also agreed and did not want to descend to dog Latin, preferring to stick by what already existed. He advocated forgetting the proposal.

**Prop. A** was **rejected**.

**Prop. B** (14: 16: 79: 0) was ruled referred to the **Editorial Committee**.

**Turland** questioned whether Prop. B was entirely an Example and supposed that it was up to the Editorial Committee to decide whether the proposal was correct in its assertion that these were nouns.

**McNeill** pointed out that that was what the Editorial Committee did—look at Examples and check their suitability. First it was checked to make sure they did in fact reflect the rules of the *Code*. Secondly, whether they were helpful or whether there were already half a dozen Examples dealing with the same topic was assessed. If an Example did not follow the *Code*, then it would not be included, and there was some suggestion here that the proposal may be in that category.

**Prop. C** (6: 47: 57: 0).

**McNeill** introduced Prop. C, which arose from what the Rapporteurs thought was an over-literal interpretation of one of the inclusions in the *Vienna Code* of the requirement that the letters of the Latin alphabet be used for scientific names. The recommendation of the Rapporteurs was that it could be referred to the Editorial Committee for action, depending on the outcome of another proposal relating to Art. 32.1. He reported that the mail vote tended to support the idea that it go to the Editorial Committee: six in favour, 47 against and 57 recommending that it be referred to the Editorial Committee.

**Van Rijckevorsel** agreed that the two proposals were linked as they were both on Roman numerals but given the mail vote, he decided to withdraw both of them. He commented that reading Roman numerals as letters did not make any sense to him. He gave the example that if you have a clock with Roman numerals, you do not say “It’s I o’clock” or “It’s half past XI”. He felt that a numeral was a numeral, no matter what anybody else said.

**Prop. C** was **withdrawn**.

### Article 28

**Prop. A** (15: 12: 84: 0), **B** (13: 9: 87: 0), **C** (15: 8: 86: 0) and **D** (16: 8: 85: 0).

**McNeill** felt that Art. 28 Prop. A was a very useful editorial modification because the more recent edition of the *International Code of Nomenclature for Cultivated Plants* provided for not just cultivars, but other terms, the “group” for example. This was a proposal that the botanical *Code* take account of those changes. He reported that the series of proposals: A, B, C and D had had strong support to be referred to the Editorial Committee.

**Knapp** suggested, in the absence of comments, that the Section vote to send the series of four proposals to the Editorial Committee.

**Prop. A, B, C** and **D** were referred to the **Editorial Committee**.

**Knapp** reported that the Section could discuss the issues associated with Art. 29, which was electronic publication, for half an hour at the end of the day’s session starting at 17:00, and put off the vote on those proposals until the following morning after people had had time to think about it and discuss it together.

**McNeill** clarified that this would apply to all proposals from Art. 29 through to Art. 31, apart from those that had already been defeated by more than 75% of the mail vote.

[*The following general discussion about electronic publication took place at the end of the Fourth Session on Tuesday afternoon.*]

### Electronic publication discussion

**Knapp** introduced a half-hour or 40-minute discussion like that on the first day on *Acacia*, not on the particularities of the voting, but a more general discussion about electronic publication. She called on someone who was on the Special Committee on Electronic Publication to start the discussion.

**Karen Wilson** began by saying that this was the third time that she had stood before a Nomenclature Section talking about electronic publication. This time she thought that the technology had matured so much more and people were getting much more used to publishing in electronic journals in particular, and she thought the Special Committee had come up with some very good recommendations, which she hoped everyone had read. Her feeling was that a lot of the issues that people felt strongly about, including the members of the Committee, had been addressed, not only in the actual set of recommendations, but in the report of the Committee.

She described it as a broad Committee of 25 members drawn not just from botanists, but from people who specialized particularly in electronic communication, from the library and archiving world, and there had been wide consultation with other people in those sorts of areas. She reported that the result was a unanimous vote on the proposals to be made, one person had disagreed with a couple of the proposals, but he actually abstained rather than voting against.

She conveyed a very strong feeling in the Committee that now was timely and that if care was not taken, if a way was not found to accommodate electronic publication in the *Code*, then it could lead to trouble because people would do it anyway. She reported that at her institution in Sydney, the library was already starting to receive copies of papers from people who said “Here’s one of my 10 copies that I’m depositing in the library because I’ve published it electronically” she referred to Knapp, who had already published some new species electronically and deposited copies into libraries. She noted that librarians were going to hate taxonomists for this because they really loathe having mini-publications to have to deal with.

[Break for end of recording.]

**Karen Wilson** resumed, addressing the issue of where people should be able to publish. Some people in the group felt that ISBN was acceptable as well as ISSN, but most of the Committee felt quite strongly that it should be restricted to the more mainstream publications like journals, so there was more chance of the nomenclatural novelties being found.

She noted that as the Committee had looked at a whole lot of the issues, they had raised questions about durability, longevity, immutability—such as how to make sure people did not change an electronic publication. She outlined that for other issues like the format, they had looked at what the *Zoological Code* had done in terms of going for a portable medium and said “no way, that’s not really feasible these days, we should go online, and that’s where things will get archived as well”.

Some people had questioned the date of publication, but she thought it was easier to assign a date of publication for an electronic journal than for a hard copy.

Regarding the immutability and the format, she stated that the Committee was particularly exercised as to how to talk about format and how to avoid talking about particular pieces of software, but they had found that there was an ISO standard for archiving—a form of PDF called PDF/A. She highlighted that the benefit of PDF was that even though it was developed by Adobe, they had made it freely available because they wanted the world to use it and there were many programs, pieces of software, that allowed creation of a PDF or could read a PDF. An added bonus was that within a PDF the fonts were embedded and digital signatures could be included so that it was possible to check whether anyone had tried to fiddle with what was written and change it. She acknowledged that it was impossible to stop someone who was really determined from changing things, but it would be apparent in a PDF whether someone had done that. She mentioned that since 2005 PDF had been an ISO standard.

The technology in the field of archiving was improving all the time, she asserted, continuing that there were a lot of international archives and national archives, whether associated with groups such as the National Library of Australia or any other country, or private groups for that matter, or industry areas.

In answer to the people who were saying “What about peer review?” she argued that electronic journals, if publication was just restricted to them, would presumably still have the same standards that they had in hard copy.

The date of effective publication, as she had already mentioned, should be quite clearly recognizable from when an article was put up on the web. She admitted that the distinction between preliminary and final versions was an issue, but some journals were already making it very clear: preliminary versions of papers put on the web often very clearly say “This is preliminary”, with the later version saying “This is the final version”. She felt that if that was included as a Recommendation the journals should identify which was the final version and that would become the norm, if it was not, in fact, already.

Another issue was pagination, in terms of how to deal with knowing what page something was published on. She noted that in general PDFs, particularly from journals, included pagination, and the Committee did not see it as an issue because the *Code* did not actually specify that you had to have pagination. She suggested that that was probably thanks to Miller and some of the early dictionaries, which did not have any pagination.

She highlighted that the benefit with PDFs was that they could be easily searched, so how to find things was no longer an issue, as it might have been in the past.

She outlined that the Committee had come up with 11 proposals, which would not be gone through as the discussion was intended to be generally about the issues involved. The Committee felt that the main proposal would be to limit the effective publication to publication in PDF in an online journal with an ISSN. She explained that the Committee had subsidiary proposals that would aim to forbid alterations and to discount preliminary versions, plus recommendations on best practice in archiving, immutability and clear designation of which was the final version.

She pointed out that half of the Committee was actually in the audience, and some of them were more informed than her in some of the aspects of what they had come up with. She thought it was a very good report, which was drafted by Arthur Chapman and Mark Newman and Nicholas Turland, who she felt had done an excellent job in setting out all their quite extensive discussions.

**Lendemer** introduced himself as the editor of *Opuscula Philolichenum*, a journal that published new taxa in lichenized fungi and lichenicolous fungi and explained that the journal had been publishing primarily electronically, with a limited print distribution of 50 copies or fewer, for the last nine years, a longer period than most people, apparently. He thought that the proposals should be voted down, and enumerated three main reasons why, adding that he was a little passionate about this issue.

First of all, he thought it was really important to point out that, even though this Committee was composed of any number of people, as far as he was aware no editors of journals that were actually doing electronic publication were involved. He pointed out that his journal was cited in the report of the Committee but he had never been contacted. He had contacted the editors of other journals that were cited in the report and they were also not contacted. He thought that while they were cited as examples of what was happening, they actually had considerably more nuanced opinions and could have offered some interesting and unique viewpoints that might have potentially influenced the outcome of what was written in the report.

Second, he really felt that the argument for electronic publication came down to a feeling that the current requirement for a print copy or a printed distribution was really imposing a burden on people and the entire system and that it was preventing taxonomists from publishing new names. In reality, he did not feel like this imposed a burden and gave the example that limited print distribution had been being dealt with for more than two centuries and he did not see why it was a problem now.

Third, he thought electronic publication was already being done. He argued that the vast majority of journals were read online, or at least a lot of journals have gone online and were probably read more frequently online as PDFs or some other electronic format, more so than a paper copy.

**Janarthanam** was also involved with running a journal on behalf of the Indian Association for Angiosperm Taxonomy. He agreed with the previous speaker, who he felt had raised pertinent points. He felt that it was one thing to go for hard copy and put soft copy online, but shifting completely to soft copy was a different thing.

He did not know how difficult it was to get an ISSN but did not think it was that difficult, potentially allowing a lot of spurious publications. He was worried about good reviewing procedures and his feeling was that it would open up Pandora’s box. His second point was that if the Section chose to support electronic publication completely, IAPT should probably come out with some actual policy on its own. He did not feel that the community could compete with the private agencies who were publishing.

**Herendeen** was strongly in favour of the set of proposals and he thought it made great sense. He believed that many of the flaws that were evident in the previous attempts at this had been solved successfully in the set of proposals this time, and he thought it was really important that this move ahead. He believed that the requirements that were suggested were more restrictive than the requirements that were currently in place for paper publication. In fact, the requirements that were in the set of proposals did not make it any easier to get names into circulation than the current requirements for names published on paper, and he did not think that was a valid concern.

In terms of the community’s access to these electronic publications, he thought in general it would be easier. He pointed out that for institutions that could not afford very expensive journals—and there were many very expensive journals that published taxonomic names that many taxonomists did not have easy access to because the libraries could not afford them—in many cases access to these electronic publications would be easier and more affordable than comparable paper publications published by some of the extortionist journals.

There was one thing to which he was anticipating hearing an objection and that was the PDF requirement. PDF was here today, but who knew if PDF was going to be the preferred format in 50 years. He suggested one way to accommodate this would be if the Section could propose an amendment to the proposal that said something like “or successor format”, to cover whatever the community standard was that replaced PDF at some point in the future.

**Ladiges** wanted to make a comment from the point of view of publishers and journals as she played a role in CSIRO, the Australian Academy of Science’s stable of journals. At various meetings of the board this kind of issue had been discussed because of the great push to have more frequent issues of journals published electronically and then only produce hard copies a couple of times per year. She reported that from a very practical point of view of speed and saving trees and not having lots of different issues published as hard copy, the general principle that had been enunciated for this proposal would be really well-supported by the Australian stable of journals.

**Alvarado** thought that the Section needed to think ahead, say 350 years, because taxonomy had been established for a long time and he felt it was important to think about what would happen if all the current media did not work in the future. He thought it was fine to have electronic publications but, at the same time, an institution should be responsible for keeping a paper record of what was being published electronically. He thought it would be especially good in some places, for example, to have a bunker-like facility such as the Darwin Centre at the Natural History Museum in London, because that would prevent all that information from being lost. He suggested that even if there was a collapse in civilization, all that information would be there ready for future generations.

**Knapp** made a Chair’s comment that she thought we [i.e. the Section] would probably collapse first.

**Glen** had had the privilege of being part of the Committee, and confirmed that they did actually discuss what the previous speaker had to say. A fact that they took into consideration was that they knew of at least three national state archive organizations that were archiving electronic data for reading in the future, for example in 350 years. This was happening daily in Australia, the Netherlands, and Sweden that he knew of personally. It was all going into the PDF/A format, which the Committee had said it wished to adopt, and the archiving organizations—the national archives of the countries mentioned—had undertaken to maintain the documents in readable format into the indefinite future. He really did not see this as a problem.

**Thiele** noted that, of the objections that had been raised, he personally found that most of them did not sway him, except that he asked the Committee to respond to the one objection that was made that this would lead to a proliferation of essentially junk electronic journals.

**Kirk** had been involved in processes that included abstracting the literature, so had some experience of peer review, and his comment was always “Peer review is a myth”. He argued that the floodgates would not open if the Section chose to go down the path that the proposals suggested. He added an example that in the last three years he knew of four new journals that had started in India publishing nomenclatural novelties in fungi that were not peer reviewed outside India and unfortunately, in his humble opinion with no offence intended, some of the taxonomic judgments had been somewhat lacking, such that peer review was not working in that instance. He did not see how it was different between ink on paper and PDF/A and concluded that the floodgates would open if they wished to open.

**Dorr** had made the original proposal for electronic publication 18 years ago and also served as a member of the Committee. He had voted for everything although he did not agree with all of the proposals, but he wanted them to be discussed on the floor. Since then, two things had concerned him: one was the reliance on ISSN, which he felt was fine for establishing whether something was validly published or not, but for the enduser who was trying to determine whether or not a name was valid and it was available for that person to use, it could be extremely difficult to establish whether or not an article had an ISSN. He had noticed that in a number of papers that were published in PDF, in non-electronic journals, with ISSNs, the ISSN was not always printed on the reprint making it very difficult for the enduser. He noted that it was easy to solve the problem for the criteria for valid publication but for the person that had to decide whether or not something had been validly published, he felt this could be a problem without the resources that he commanded.

The second thing echoed something mentioned earlier: given that it had taken 18 years to get to this point, given that the Nomenclature Section only meets once every six years, if a format was chosen that failed or proved difficult, some sort of stopgap measure would be needed that allowed the problem to be corrected in the interim. He thought that if the wrong choice was made and Pandora’s box was opened up it could lead to a lot of difficulty in five or six years, but if there was some mechanism for trying to put the lid back on he thought it would be better.

**Norvell** was also editor-in-chief of *Mycotaxon*, which published four volumes annually, comprising around 20 to 100 pages and covering about 250 manuscripts. This year in January, she reported that they had converted, with the expectation that these proposals would go in the Congress, to an online journal. They were still submitting their bound volumes to selected libraries.

She described a number of things that they had encountered and really liked: one was that on each and every article the ISSN was used and they had also specified use of a DOI [digital object identifier] number, which meant that the publication or PDF file would always be freely obtainable by typing in the DOI, securing that the PDF file could be found independently of a disappearing website, which was one of the concerns.

She gave economics as the reason that the journal that was founded in 1973 by Richard Korf had moved from a strictly hard print copy. She added that she was a bibliophile who loved hard print and, as a bribe, Dick [Korf] was giving her her own bound volume, which still landed on her desk with a solid thump, which was what she wanted. Returning to her main point, she noted that libraries were discontinuing subscriptions and the journal had a rather small readership making it much cheaper to publish online, in the order of several thousand dollars difference. The journal could not afford the postal charges, because they keep rising and they were losing subscribers. Since the change she added that unfortunately the submissions were going up, not down as they had anticipated. In conclusion she was very much in favour of the proposals.

**Gams** noted that there were quite a few journals that published their papers online before the printed hard copy and, according to the proposals, the date of the online publication would now count, contrary to the previous situation, which had been from the moment where the hard copy was available.

He felt that pagination was certainly not superfluous, especially when citing a basionym for a new combination where it was crucial to have pagination. He wondered if this would now require having a DOI number, printing it on paper and manually counting to see that the species was published on page 17.

**Sennikov** wanted to analyse the situation from the pragmatic point of view, because in his opinion there was only one question that was really practical: either having electronic publication with paper deposits or having regulated electronic publication without paper deposits. He added that what would be decided was those deposits would be made and put in public libraries or not. He felt that this was the plain fact that was the consequence of the practice that was already established. In his opinion this would make the paper deposits the most obscure type of paper publications that had ever appeared, because they appeared in minimal number of copies and he questioned the accessibility of the paper deposits, should you believe that they gave proof of valid publication of the name and that the text was unchanged.

Recently he had asked the editor of the exclusively electronic periodical *Forum Geobotanicum* about effective publication. The journal was published in Germany, not India, and a number of nomenclatural novelties had been published. The answer he had received was, yes, you make paper deposits. He then sent a request to receive one of their official publisher’s paper deposits but received no answer.

He returned to the question of junk electronic journals, but felt that there were already plenty of junk paper journals. He felt that it may happen that grey publishing would be produced by electronic publishing but, at the same time, currently he felt it was an easy possibility to publish whatever, wherever, and whoever can do that. He noted that Constantine Rafinesque had had difficulty in publishing his books 108 years ago because he needed to invest a lot of money to produce thousands of his novelties. He gave the example of needing just €100 to publish a book nowadays by any internet publisher like Google, and it would be deposited in 10 copies somewhere, so it would be possible to publish a good deal of junk in this way and it would be all validly published.

He thought it was really a pragmatic matter and that nothing would change for the worse should the Section approve legality of electronic publication. He added that there would just be benefits and could not see that anything could get worse.

**Kellermann** noted that anybody could get an ISSN so he thought that putting an ISSN or ISBN into the *Code* did not guarantee quality. He gave the example that he could go to the National Library, fill out a form and then have an ISSN to publish *Jürgen’s Journal of New Names*.

**Reveal** had been out of university administrations for 12 years and wanted to know how many of those present had, within their university, policies for promotion and tenure provisions that allowed electronic publications. He noted that at Cornell it was not permitted, while at Maryland it was permitted. So he thought that young people needed to look very carefully at where they published or put a requirement in that all electronic publications have hard copies mandated.

**Paton** told his children that he was coming to a conference to talk about plant names, so he suggested that they already thought he was an old, irrelevant git. He admitted to having difficulty relating to them, and went on to say that it was going to be six years before the Section would get to talk about this subject again. His argument was that, given there was a high demand for information on the internet, if the taxonomic community did not provide a mechanism for others to publish information on the internet, it would look old and irrelevant.

**Penev** from *PhytoKeys*, also a publisher, wished to stress three points.

First, today’s print version was not the print version from 10 years ago. Anyone could print one or two perfect-looking copies of a print version. He felt that this was not a proof of good distribution, nor a proof that a print version was sustainable, but it was just a proof to satisfy the *Code*. He argued that existence of the print version was not a bad thing, but it should not be a criterion for effective publication anymore.

The second thing he wished to point out was that when electronic publications were referred to, it was really important to set very strong criteria for that. He agreed that anyone could get an ISSN, but anyone could also get an ISSN and make a print journal in copies so that was not a change. He felt that a DOI number—especially a CrossRef DOI number—on each article was really essential to have, and also a very strong archival policy for electronic journals, for example to recommend archival in at least one ISO- (International Standards Organization) certified repository, because there were also institutional repositories, say of smaller universities, which probably would not exist in some years.

He strongly supported allowance of electronic publications, and the question was really pragmatic, whether all electronic publication should be allowed, and should the date of electronic publication be the date of availability of the taxon, He felt that this was really important because it made the system more efficient.

**Cantrill** noted that one of the things that was being faced at the moment in this realm and in this country [Australia] was that the Government was very focused on accessibility for people with disabilities. There was legislation in place that would mean things like PDFs were not compliant, and so it was illegal to put those up without having all the other disability access to those sorts of articles. He reported that it was a real issue also happening in other countries around the world. He suggested that part of the solution meant dealing with some of those issues. He gave the example of the BHL [Biodiversity Heritage Library] Australian node site, where all the articles were available in three or four different formats to comply with disability accessibility provisions covered under legislation.

**Gandhi** had a very minor comment about lack of page numbers that were required in the citation of basionyms. As Karen Wilson had already mentioned, the basionyms that came from Miller’s 1754 work or 1768 work or even Rees’s *Cyclopaedia* had no page numbers but were still basionyms of valid names, as long as the full bibliography was given.

**Barkworth** responded to Reveal’s comment that the question that came up at her institute was what was the importance value in one of those indexes as a measure of importance of a journal, rather than how it was published. So she did not think that one [that the publication was electronic rather than printed] was of particular concern to the young people coming up.

**Knapp** asked for other comments and was excited that it was a young person! [Laughter.] She suggested from then on to choose speakers based on age.

**Flann** felt that electronic publication was basically an inevitable direction that the Section would have to deal with. She argued that journals were simply going electronic for economic reasons. She thought it was admirable that those in the room with journals deposited their print copies, but did not feel that the Section could assume that all of the other journals, especially the larger ones, which were not entirely nomenclaturally based, were going to do that over the next six years.

The other issue she brought up was that the *Code* was a living thing and there would probably be issues that would come up and they would need to be ironed out, but the Section did not have to wait 350 years to solve any issues that arose. She supported the proposals.

**Crane** wanted to make a comment about institutional subscriptions to e-journals. He reported that in the heyday of the University of Illinois, it purchased 39,000 hard copies of journals, and gradually they were whittled down and whittled down until they were now buying electronic journals in packages, which meant that a journal that you use or might need would not be available to you. It was obvious to him that everyone could not subscribe to all of these e-journals personally and would have to depend on institutions. He wondered how to get around this problem. He gave the example of the *Canadian Journal of Botany* and his institution cancelling the e-journal. Would the institute hold the back issues of the electronic journals or would they lose everything? This was not clear to him. He added that for some e-journals, only abstracts were available because of copyright. These were problems that he felt needed to be solved.

**Marhold** returned to the argument that journals could print copies and send them to libraries. He agreed that this may be true when it concerned taxonomic journals or journals that published taxonomic novelties in each paper. But he made the point that there were a lot of journals in which taxonomic novelties would appear only from time to time, and they would not be willing to publish whole volumes and send them to libraries. He concluded that the result was that the individual author should take just his own paper and send it to libraries. As many people had stressed, he agreed that the libraries were very unhappy with that. He added that the likelihood of being able to trace these papers, even in the reputable libraries, was low. He agreed with the fact that the requirement for a few copies of often individual papers sent to several libraries was useless because these libraries would have no capacity—even if they had the goodwill—to catalogue all the minor items that would come every day.

**May** strongly supported the proposal, and agreed with Flann that there may be some working out, and it would form a foundation for further progress.

In terms of access to descriptions he acknowledged that at the moment if you did not have an electronic subscription you could not get access, but argued that was the same if your institution did not have a print subscription in the past: some people had access, some people did not. He suggested the direction to move in the future, and gave the example of what mycology was starting to do: the deposition of the protologue in an online repository, which was easier if the versions were already electronic, and that was something to think about next time. He added that as more and more protologues became electronic, it became easier to automatically lodge them in an open-access repository, and suggested that would mean not having to worry about the subscription. He saw the proposals as a step along the way, and agreed with Flann that some of the details could be worked out.

[*The following discussion took place in the Fifth Session on Wednesday morning.*]

Before starting the discussion on the proposals made by the Special Committee on Electronic Publication, **Knapp** clarified that the opportunity for discussion of the principles of electronic publication had already been given to the Section and she was very clear that the following discussion should be confined to the actual proposals that had been put forward to change Art. 29. She warned that she would be quite fierce and cut people off if the discussion digressed to general principles.

### Article 29

**Prop. A** (92: 29: 0: 0).

**McNeill** introduced Art. 29 Prop. A from the Special Committee on Electronic Publication. This was the Article that dealt with what constituted effective publication and the proposal was to add the provision that “Publication was also effected by electronic distribution of material in Portable Document Form (PDF; see also…” including references to Recommendations still to be discussed “in an online serial publication, with an International Standard Serial Number (ISSN)”. At the end of the Article detailing how publication was not effected, the proposal added that a name was not effected by distribution electronically other than described above.

**Herendeen** offered a **friendly amendment** to add the phrase “or its successor format” after PDF was mentioned, so that in the future, when the standard moved from PDF to something else, the *Code* would accommodate that.

**Karen Wilson**, as the Chair of the Committee, was quite happy with that, but altered the wording to “successor standard format”, because she thought it was important to make it clear that this was an international ISO standard and that anything in future followed the same format.

**Dorr** suggested that since there were some 20-odd members of the Committee, it be agreed that if an amendment was going to be ruled friendly or not Karen Wilson was the person who decided, rather than soliciting all 20 involved.

**Soreng** proposed a **friendly amendment** to the **amendment** to add “accepted” successor, so it was not just any successor that came up and the Section would have to vote on it somehow. He suggested the wording “Or its accepted successor standard format by this body” or whatever wording seemed suitable to the Editorial Committee He clarified that by “this body” he meant IAPT.

**McNeill** thought that was actually the very reverse of what was proposed: that if there was a successor in between Congresses that was an international standard the article would still be effective, even if there was not an International Botanical Congress to change it. He suggested that this was actually quite unnecessary, because if in fact it was accepted by the Congress then it would be changed anyway meaning there was no need to put that in. He added that talking about accepted meaning “accepted by the publishing community generally”, was one thing, but if the intended meaning was “accepted by a Botanical Congress”, then there was no need to include it in the *Code* at the moment, because it would then be included when that new *Code* was produced.

**Soreng** saw perceived problems developing when the new standard came up and people started using it, but it was not widely used.

**Karen Wilson** suggested that when she said “standard” she was thinking of the international standard as set by ISO and an alternative would be to put “the standard ISO format”, but she was not even sure whether it would be appropriate to put “international standard format”. She continued that the ISO was a body that had been around forever and a day, but wondered what the meeting thought about it.

**McNeill** suggested that the word “international” be put in at that point. [This was considered a **friendly amendment**.] He added that it still had to be dealt with whether “accepted” was being proposed as an amendment or not.

**Herendeen** replied that he had been going to suggest “industry” standard, but thought “international standard” did the job just as well and his opinion was that the word “accepted” was not necessary. He thought that an international standard would do the job and acceptance by this body or IAPT could be less informed than where the industry was going.

**Knapp**, as mentioned on various previous occasions, reiterated that she did not want the Section to attempt to wordsmith proposals from the floor and felt it was quite important to establish that precedent from the outset, because it was already starting to occur to a certain extent.

**McNeill** asked the proposer of the word “accepted” if he still wished to insert it.

**Soreng** accepted the removal of “accepted”. [Laughter.]

**Knapp** summarized that the proposer of the word “accepted” accepted that “accepted” could be deleted so all was very acceptable now.

**Lendemer** proposed an amendment to Art. 29 Prop. A.

**Knapp** read out that the amendment was “provided that the minimal requirements for the distribution of printed matter outlined above are met”.

**Lendemer** noted that it also included adding “see Article”—and “see Rec. 30A.2”. [The amendment was **seconded**.]

**Barrie** wondered how the amendment was different from the current wording.

**McNeill** agreed.

**Greuter** felt that it was such a fundamental amendment that it should have four seconders, but as he also felt that they would be obtained he did not press that point.

He thought the amendment would make the thing not unworkable but very problematic, because it dealt with conditions of effective publication that were basic for valid publication. He argued that if the amendment was accepted it suggested that the condition for electronic publication to be valid was that, simultaneously or later on, printed matter was distributed, making effective publication dependant on a future event. He felt this would cause many problems, some foreseeable, some perhaps unforeseeable, for instance he wondered what would happen if, due to a mishap, the distribution did not take place, although it had been announced. He added that it also raised the point, not discussed here and not easily implemented, about the date of effective and valid publication. He strongly advised against accepting the amendment.

**Lendemer** did not see it that way at all. He thought that it made pretty clear this would essentially continue the process that already existed, basically acknowledging that electronic publication was allowed under the *Code*, provided that the minimal requirements for print distribution that were already set up and had been used for the past 100 years—he corrected himself to say since Vienna—would continue to apply. He did not see a conflict and added that any such conflict could be perceived as editorial, at least with any of the future proposals that come up.

**Stevens** spoke on behalf of the librarians he had talked to about this and reported that they were literally living in fear of having a few copies of journals or articles sent to them as they basically did not know what to do with them. He felt that the idea of a few printed copies being produced was a really bad compromise.

**Dorr** agreed with Greuter that too many contingencies meant that inevitably one of the contingencies would not be met, leading to a lot of names that were debatable as to whether or not they were valid, because somebody could only track nine copies having been deposited. He asked, who was going to be able to ensure that the publisher did distribute the 10 copies? What if they distributed nine? What if one of the libraries did not accept them? He argued that it became unworkable as presented in the amendment and it defeated the purpose of having electronic publication.

**Buck** contended what Stevens said. He admitted that there were certainly always going to be lazy and crabby librarians. He noted, however, that in the last six years since Vienna, articles had been distributed in this way, including Sandy [Knapp]’s articles. At the New York Botanical Garden the library had accepted that this was the way libraries were going. He suggested that if they were going to drag their feet, they were not going to be a good library and they would have to accept that dealing with these electronic and small-copy things was the future of libraries. He knew that his institution’s library just put them in an archival folder, gave them a catalogue number and put them on the shelf and there had been no problem with that whatsoever.

**Marhold** did not think this was current practice before the Vienna Congress. He noted that if a journal was published traditionally there was at least some assurance that it would be distributed to libraries, but he felt it would be silly to ask for any requirements on distributing paper copies. He suggested it would be wise to enumerate the libraries to which copies were sent as he argued that if this was not published anywhere, in 20 years there would be no chance of finding whether the copies really were distributed and whether it was effective and valid publication. He added that maybe some libraries would regularly put sent articles on the shelf, if they had enough staff and enough money, but he thought this issue was more relevant to local authors sending copies to local libraries that were understaffed rather than the big libraries.

**Kellermann** agreed with the former speaker as he knew of lots of libraries that discarded reprint collections, either because it was now on the Biodiversity Heritage Library or available online elsewhere. He argued that an article might be lodged now, but in 10 years there was no guarantee that the reprint would still be in the library unless he suggested that “keep forever” was printed in red letters on them.

**Magill** wanted to say that this was no different to what had been done forever as the *Code* already said that information had to be put into libraries. He wondered how in the old days when they published a few copies, it was possible to know that those were still there. He did not see any difference between what had been done in the past and the amendment.

**Redhead** called the question.

[There was a sufficient majority in favour of voting on the **amendment** and there was a 60% majority against so the **amendment** was **rejected**.]

**Knapp** returned the discussion to the proposal.

**Hawksworth** proposed an **amendment** to add “Or successor approved by the General Committee”.

**Knapp** reiterated the urge to avoid wordsmithing proposals. [The **amendment** was **seconded**.]

**May** felt that the decision about what an international standard format was should be left to the appropriate bodies, not the General Committee. He also thought that this raised the question of the time between the Congresses. He considered six years a long time, as things were moving very fast in lots of areas. He supposed the General Committee could meet in between.

**Glen** noted that this had been discussed in the Special Committee and the conclusion was reached that PDF archival was an ISO standard and any future standard would include the current standard. He personally believed that it was up to the ISO rather than the General Committee to determine any modification to the standard.

**McNeill** explained why he seconded the proposal. It was to remove uncertainty on the part of users as to whether in fact a particular standard would be that of valid publication. He clarified that the General Committee did not meet, except electronically, and added that it would make a decision once the standard had been approved in a matter of a week or two. He felt that this was simply a matter of providing a clear indication to the botanical community that an international standard had been accepted and had replaced what was currently in Art. 29.

**Funk** requested an explanation of the difference between the General Committee and the Bureau.

**McNeill** explained that the General Committee for Botanical Nomenclature, which would be elected on the final day of the meeting, was the body that handled botanical nomenclature between Congresses. It had nothing to do with the Bureau of Nomenclature of the Section. Each Bureau was appointed by the International Botanical Congress that it served. He elaborated that it was the organizers of the Melbourne Congress that appointed the people here with the exception of the Rapporteur-général, who was appointed by the Vienna Congress. In the same way the next Congress, wherever it be held, would have a Rapporteur-général appointed by this Congress.

The General Committee was an ongoing body that was active throughout the sixyear period from one Congress to another, if anything arose of importance to botanical nomenclature between Congresses, it was the body that could handle it speedily. He added that that was the reason for his supporting the suggestion of Professor Hawksworth. It would be able to respond to a situation like this and advise the botanical community that there was a new standard that was adopted by the International Standards Organization and endorsed by botanical nomenclature.

**Soreng** wondered what would happen to PDFs if the successor or international standard became accepted by the international community and the General Committee. Would they remain acceptable, or would a move to the new format be necessary?

**McNeill** noted that most standards were backwardly compatible.

**Glen** explained that part of the definition of PDF/A was that it was backward compatible and if there was a new standard, it would include being able to read the previous standard or previous standards, plural. He did not see this as a problem.

**Thiele** understood John’s point exactly, that given that this was such an essential issue for effective publication absolute clarity was needed as to whether a particular standard was effective or not. He also agreed with the point that the General Committee may not be the best body and expected that the General Committee would be a little more than a rubberstamp of the ISO. He wondered whether, taking up Karen’s point, the issue could be effectively devolved to the ISO by putting perhaps a footnote or referring to the ISO standard within the *Code* so that there was entire clarity. He argued that then whatever the ISO decided and as soon as the ISO decided on a successor standard, that was by necessity the standard in the *Code*.

**Hawksworth** pointed out that it was conceivable that there would be more than one format produced that met an ISO standard. He did not think it was reasonable to assume that only one would actually exist, because that was not what happened in some other areas of the ISO operation.

**Barrie** felt that the point of having the General Committee look at it was not that the General Committee was going to evaluate as to whether or not it was a standard—he was sure that the General Committee would look at the ISO standard and accept that—the point was that the General Committee would look at it and approve it and communicate it to the botanical community. He suggested that the crux of the issue would be an announcement that said that the General Committee noted that there was a new standard and it was the one to be followed, rather than critiquing it, unless the Committee was a lot more qualified than he thought it was.

**Paton** wondered if it be better to change “approved” to “communicated”.

**Hawksworth** thought that it needed to be “approved”, because it was not possible to know what ISO’s successors were going to be and he did not think it was possible to assume there would only be one, because the ISO would have a minimal requirement for a particular format, or there may be 10 different companies that put out things that meet that one, all of which we may not feel were appropriate for systematic work. He suggested it was limiting it too much by saying “the successor” and it should just be left to the ISO.

**Nic Lughadha** suggested the **friendly amendment** to change “its” to “a”, and “approved” to “communicated by” so it would read “Portable Document Format or a successor international standard format communicated by the General Committee”. She felt that would leave the General Committee open to approve and therefore communicate any number that it considered acceptable of the potential successor formats. [The **friendly amendment** was **accepted** by the proposer.]

**Herendeen** apologized for being thick, but did not understand why the “communicated by the General Committee” needed to be added.

**McNeill** thought he had tried to explain why: that it should be clear to the botanical community which formats were acceptable for publication when there was a successor, and then it was simply a method of communicating that, and that was now enshrined in this Article.

**Herendeen** supposed he did not see any harm in it.

**Knapp** summarized that the amendment was to have the General Committee involved. [The **amendment** was **accepted**.] She returned discussion to the original proposal as so amended.

**Redhead** pointed out that the way it was phrased “or a successor”, opened up the possibility that in the future PDFs may not be considered effective publication. He suggested it was not desirable to de-validate names that were already validated, so he was going to suggest “and any successor”.

**McNeill** thought it was a very good point, and it should be considered in looking at whether the wording needed to be improved.

**Knapp** agreed that was a very good point that the Editorial Committee should take into account.

**Soreng** was not sure where registration might play into this, but he felt that registration should be a requirement for electronic publication, because there was going to be a proliferation of journals.

**Knapp** asked if he was proposing an amendment to the proposal.

**Soreng** asked if it was the appropriate place to bring up registration. [Several mutters of “no”.]

**Knapp** noted that yesterday in the discussion might have been an appropriate place to bring it up. [Laughter.]

**Soreng** had wanted to bring it up yesterday, but had not been called on. He proposed adding “for electronic publication to be effective, the individual names proposed must be registered”. [The **amendment** was **seconded**.]

**Karen Wilson** noted that in the course of the Committee discussions, whether registration should be considered as part of electronic publication had certainly been looked at. She reported that the feeling was that it was a separate matter that should affect all names, not just electronic names so it was not part of the remit of the Committee. She added that it had been addressed in the report that went into *Taxon*. She personally thought that some tracking device would be needed, but highlighted the existence of IPNI for the vascular plants and noted that the mycologists had a proposal coming up to deal with registration in a form, which was basically sending the information to an indexing centre a bit like GenBank. She reiterated that there was no way that the Committee felt as a group that it should be a requirement for electronic publication, they did not think names should have to be registered first. She noted that it was a matter of making sure that everyone knew where to find the names afterwards. She added that she would be very unhappy to see it added, because she thought that it was an inappropriate place and premature to put something like that in when there was no process to actually deal with it fully. She listed a number of questions that would be needed to consider: What would you do with fossil names? What would you do with algal names? She pointed out that they did not have the same indexing centres that the vascular plants and the mycologists are lucky enough to have.

**Marhold** was very much in favour of registration and thought it held not only for electronic publishing, but also for various minor publications. He did not think that this was the place for it in the *Code* nor that this was the Article where it should be discussed. He added that there would be an Article about registration of fungal names and that would be the appropriate place to discuss the registration of all names.

**Janarthanam** thought the intentions of the amendment were good, but wondered at what stage they were supposed to register. Was it while sending for publication or after, accepting publication or after?

**Barrie** was hoping the “R” word wouldn’t come up so fast, but it was a very complicated issue and he did not think it was something that could be handled right here. He proposed a Special Committee on Registration to look into the issue and all the ramifications of it to report to the next Congress.

**Knapp** noted that would be done at the end of the Section.

**Orchard** agreed that it might not be the best place to put this amendment, but on the other hand, given that this was the key Article that was going to regulate whether or not we have electronic publication, he wished to know whether registration was part of the deal or not before voting on it. He added that if it was not going to be here, then some provision was needed to make sure that it was discussed as an initial part of this process at a later date.

**Karen Wilson** referred to Rec. 29A, which would be considered shortly, and which was a very strong recommendation that all effectively published electronic material be archived, that it be placed in multiple online digital repositories, and preferably in different places around the world. That was the implication because the Committee was very conscious that people needed to be able to find the electronic names. She added that with Google these days it would be a lot easier.

**Barkworth** noted that somebody had said earlier that registration would have to apply to everything, not just electronically published names. She reiterated that this Article was talking about electronic publication of names, and urged the Section not to muddle the registration issue with this one at this time

**Knapp** moved to a vote on the amendment. [The **amendment** was **rejected**.]

**Greuter** missed one thing in the discussion. If the Section voted on this, he saw quite a danger that names that were not so far effectively published would retroactively become effectively published or publications would become effective. He thought in fairness there should a startingpoint date inserted, which could be 1 August 2011, after the Congress had ratified the Section decisions. He suggested that it was no offence to anyone if it started then before the *Code* was published, but certainly not before that date. His proposed **amendment** was “On and after 1 August 2011, publication was also effected…”.

**Turland** pointed out that there actually was a starting date. Article 30 Prop. A proposed to add a new Article to Art. 30: “Publication by distribution of electronic material does not constitute effective publication before 1 January 2013”.

**McNeill** suggested that when that was discussed it could be considered whether the date should be changed to an earlier one.

**Greuter** preferred it if it would be voted upon together.

[*It was decided to discuss and vote on Art. 30 Prop. A before voting on Art. 29 Prop. A. The proposal was accepted with an amended starting date of 1 January 2012. The discussion is noted under Art. 30.*]

**Knapp** returned the discussion to Art. 29 Prop. A, to re-word Art. 29.1.

**Davidse** suggested that the intent of the proposal was to limit electronic publication to serial publications in contrast to books or one-off publications. However, he pointed out that there was a whole genre of publications that was in between the two extremes. He questioned whether the International Standard Serial Number was sufficient to differentiate the in-between situations and wondered if that had been addressed.

**Karen Wilson** confirmed that the Committee did think hard about it and the feeling was that they should put some restrictions on publication of electronic names to make them more easily findable. She explained that that was the reason for saying ISSN and added that there was a subsequent proposal to extend that to ISBN as well. She continued that getting into other literature that did not have an ISSN or an ISBN or the electronic equivalent probably meant they were publications that were not widely distributed and not widely accessible to the taxonomic community. They were trying to make sure that anything electronic was widely available to everyone. She reported that the Committee had put the restriction on, because they did not think it would be acceptable to taxonomists in general to have it open and just publish anywhere for electronic publication. She suggested that there may be a much better system in the future, but to start with they thought it was better to be cautious and to restrict it, because the journals in particular did have a stake in making sure that they did the right thing.

**Janarthanam** supported what Karen Wilson was expressing and proposed one more amendment: adding the word “peer-reviewed” because he saw a problem in a lot of ISSN journals that were not peer reviewed. [The **amendment** was **seconded**.]

**Buck** noted that they had intentionally discussed this in the Committee, but decided because it was not required for hard copy journals it should not be required for electronic journals.

**McNeill** agreed.

[The **amendment** was **rejected**.]

**Whitbread** felt that there was a very big difference between a portable document format and archival portable document format and proposed that “archival” be added before “portable document format” and “PDF/A” in the parentheses.

**Karen Wilson** responded that the Committee did consider that very seriously and added that Rec. 29A Prop. A specifically said that there was a new Recommendation that “Publication electronically in Portable Document Format should comply with the PDF/A archival standard (ISO…” and the number of the standard. They thought that at this stage it was perhaps best to leave it as a Recommendation, but make it very clear that they thought this was what should be used.

**Whitbread** proposed the change as a formal amendment to add the words “archival” and then to add “PDF/A”. His reasoning was because an ordinary PDF document allowed many things such as encryption, inclusion of sound files and reference to external documents and was therefore not integral to itself and could contain links to other things elsewhere on the internet making it potentially dangerous. [The **amendment** was **seconded**.]

**Kirk** offered what he hoped would be a **friendly amendment** to the amendment—to capitalize the letter “A” so that it was clear that it was *the* Archival Portable Document Format, not *an* archival…

**Barrie** had a question for the Special Committee on Electronic Publication as to what the disadvantages were of doing it this way.

**Turland** was on the Committee as well but had been keeping quiet because he wanted to be impartial. He explained that when the Committee considered this one of the reasons they included it as a strong Recommendation, rather than enshrining it in the rule, was because it would be very difficult, if not impossible, for users of the *Code* to discern whether a PDF was archival or not, and effective publication would, with this amendment, depend on an archival quality of the PDF and not just a PDF. He elaborated that recognizing a PDF was straightforward, but recognizing an archival PDF required a certain amount of computer geekiness, for want of a better term.

**Hollowell** reported that the two journals, *Annals of the Missouri Botanical Garden* and *Novon*, had tried to implement this a year ago, to change their reprints to PDF/A, and it was just a matter of communicating how they wanted to embed the metadata. She noted that it had worked just fine and simply required software with the latest update with a menu option. They did not want security restrictions or patented compression or JavaScript enabled and she offered a list if anybody wanted to see it. She concluded that it was certainly feasible and had been done with both BioOne and Allen Press successfully although those with an old version of Adobe PDF would have to upgrade. She supported the more general PDF for now, with the PDF/A to be considered by the Section.

**Nic Lughadha** felt that the issue was not whether it was possible to make them [PDF/A files]; the issue was whether it was possible to spot them. She could spot a PDF but would not know whether it was a PDF/A or not. She suggested the Section needed to be able to decide whether something was effective and therefore valid in a straightforward manner, without all becoming computer geeks.

**Miller** much preferred this as a Recommendation as he thought it was somewhat discriminatory against people who did not have the most current software and were not able to do this. He thought putting it in this place and making it a requirement would limit the number of people that were able to do it effectively.

**Bayly** agreed that there was a difficulty with a user spotting the differences in the types of PDF, but also knowing ahead of time what journals used as their standard format for publishing was something that it would also be necessary to be aware of and whether all journals would necessarily use the PDF/A format. He pointed out that this involved imposing another restriction by making it part of this particular Article. He supported this as being part of a Recommendation rather than this Article.

**Thiele** supported the amendment. He certainly took the view that it was difficult to determine whether a document was archival or not, but felt that the archivability of these documents was so important that right from the beginning of electronic publication it should be mandated. He felt that it would lead to strife in the future with non-archival publications if it was not mandated from the start.

**Peter Wilson** agreed with Bayly and wondered if the Section was in the position to mandate that Springer, for example, would adopt this as they would have to adopt it for their entire journal stable and he did not think the Section had the power to force Springer to use PDF/A for their entire stable of journals.

**Nelson** queried whether, if this passed, it meant a name published not in the “A” format [PDF/A] was not valid.

**McNeill** confirmed this was correct.

**Knapp** clarified that it would not be effectively published.

**Nelson** felt that that was a very severe restriction, which would not be transparent to most people. He questioned how it was possible to have an invalid name that could not be perceived to be invalid by the majority of botanists throughout the world and concluded that it was not a good situation.

**Whitbread** argued that if you were going to say Portable Document Format, you might as well say Word file or HTML file, because there was no guarantee that PDF file made today would be readable even one year from now. PDF is not an international standard format; only PDF/A is.

**Karen Wilson** was looking very hard here at the report, because the Committee certainly did look into this. She was not sure that she agreed with Greg [Whitbread], but she acknowledged that he knew the electronic area very well. Certainly the PDF/A was the current international standard for long-term archiving, but she could not see that in a year’s time, ordinary PDF was going to be unreadable. She thought they would always be backward-compatible because it was such an important format for everyone and she did not think it would be a practical problem, particularly if there was a move towards getting acceptance by the journals of the PDF/A as the standard to be used as a taxonomic community.

**Ladiges** called the question. [There was a sufficient majority in favour of voting on the **amendment**.]

**Knapp** clarified that what was being voted on was amendment of the addition of the words “Archival” and “/A”.

[The **amendment** was **rejected**.]

**Prud’homme van Reine** asked for clarification as to what would happen with Prop. B if Prop. A was accepted.

**McNeill** answered that it would be discussed after coffee. [Laughter.]

**Challis** clarified the point on whether PDF was an international standard. She noted that on page 1856 in the Report of the Special Committee on Electronic Publication, the bottom right-hand column, carrying on to page 1857 made it clear that PDF was an international standard, because the amended wording of Prop. 203 now said “its successor international standard format”. So PDF was an international standard format.

**Prop. A** was **accepted** as amended.

**Knapp** freed everyone to go and have tea and a biscuit to get the sugar levels up again. [Applause.]

**Prop. B** (77: 38: 3: 3).

**McNeill** introduced Art. 29 Prop. B, which was also from the Special Committee on Electronic Publication. He explained that it was quite short, and the preliminary words were to extend in Art. 29.1 what had already been accepted for serial publications to works that were identified by an International Standard Book Number.

**Head** supported the idea in principle, because it made things more inclusive, but from a practical perspective he was just a little bit concerned about how a book could be represented by a single PDF when in reality most books were electronically produced as a series of PDFs, typically one PDF per chapter. He wondered from a technical point of view how that problem could be resolved.

**McNeill** asked where there was a suggestion that there was one PDF involved.

**Head** felt that a name published in a work of literature would typically be represented by a single PDF if it were in a journal whereas in a book it might be represented by a series of PDFs, he just wondered if that issue had cropped up.

**Kirk** did not really see a problem. He felt that it was either a small PDF or a big PDF.

**Greuter** could see a potential difficulty that he hoped would not happen, in terms of reference if someone had the bad idea of producing an electronic publication in PDF without pagination. He would like to have the words “with pagination” or “paginated” somewhere associated with the PDF file.

**McNeill** clarified that would be solely in the case of books otherwise it was not really something that was open to discussion.

**Greuter** did not know whether any serials or periodicals without pagination existed but did not think it would do any harm to make it general. He proposed an amendment to put the word “paginated” before the word “publication”. [The **amendment** was **seconded**.]

**Kirk** requested clarification as to whether “paginated” meant in its entirety for every page or partially paginated, because some current journals, ink-on-paper journals, were partially paginated and putting something in the *Code* that was ambiguous was undesirable.

**Greuter** supposed that if there were unpaginated portions these would not be effectively published but paginated would, and he hoped that new names would appear in the paginated portion because he wanted to be able to cite them.

**Kirk** claimed that this would invalidate some fungal names…

**Knapp** highlighted that she had not recognized him and admonished him: “Paul Kirk, don’t you dare speak until I recognize you”. [Laughter.] She then relented and allowed him to speak.

**Kirk** continued by saying that if that was the intention then some names of fungi published in the last few years would be invalid, because the page on which the name appeared did not carry a pagination because it was a trendy way of publishing now, to not have a page number on the first page.

**Wiersema** gave a reason for having the pagination requirement: Art. 33.4, where if a name was based on one of the electronically published names it was necessary to give a full and direct reference with page or fig reference, and he wondered how to cite the page if there was no pagination.

**Kellermann** noted that there was no requirement for pagination for printed publications and gave the example of many basionyms in Rees’s *Cyclopaedia*, which did not have any page numbers and was still accepted. He also made the point that sometimes PDFs had a discrepancy in pagination where the electronic document was paginated from 1 to 20 but the actual page numbers of the journal might be from 250 to 260 [sic].

**May** agreed that if the condition was not imposed on paper it should not be imposed on the electronic version. He added that there were plenty of fungal names published recently in an effectively published publication called *Fungal Planet* that was not paginated and if it were necessary to refer to that, it was simple to put square brackets around the inferred page number and there was no doubt as to which was the first page and the second page and so on. He felt that there already was a mechanism of dealing with that and there was no need for conditions for electronic publication that were different to the rest.

**Lendemer** thought that the spirit of electronic publication was that of moving forward and progress and he felt that it would be moving forward and progressive to actually be able to cite the place of publication with a page number, so he was in favour of the amendment.

[The **amendment** was **rejected**.]

**Knapp** returned the discussion to Prop. B as it was originally worded, to replace “serial publication with an International Standard Serial Number (ISSN)” with “publication with an International Standard Serial Number (ISSN) or an International Standard Book Number (ISBN)”.

**Malécot** suggested that everyone open their *Code* and look on the first page. There was an ISSN in the *Code* and there was also an ISBN. His point was that it was possible to have both on the same volume, in this case there was an ISSN because it belonged to *Regnum Vegetabile* and there was an ISBN. He felt that it was a good recommendation and was in favour.

**Buck** added a point of clarification that in the past the people who issued the ISSNs or ISBNs would allow both, but now a monographic series was only allowed one—an ISBN—if each issue of the journal was a freestanding, one-article entity. He noted that it was no longer possible to have both; it was just that the *Code* was old—six years old.

**Knapp** added “ancient”.

**Prud’homme van Reine** noted that in his synopsis Prop. B also included other things—“If it were thought necessary” and so on—that were not on the board. He wondered what had happened to that part.

**Dorr** explained that that was a note clarifying the text because in *Taxon* Prop. 204 did not contain that text.

**Turland** added that the paragraph in question that was printed in the synopsis was lifted straight from the proposals by the Special Committee on Electronic Publication and was not actually part of the proposed wording of the new rule. He clarified that he was referring to “If it were thought necessary, footnotes could be added to ISSN and ISBN noting that the prefix ‘e’ or ‘e-‘ (e.g. eISSN, e-ISSN) is sometimes used for electronic publications but that such designations represent the same standards”. He reiterated that this was not proposed as part of the wording of the *Code* but was a note made by the Special Committee in their proposal.

**Greuter** suggested that if this proposal was accepted it implied the empowerment of the Editorial Committee to add those notes if, as it said, it was thought necessary or desirable.

**Cafferty** asked somebody from the Special Committee to explain why the proposal did not receive the unanimous support that Prop. A and C did.

**Karen Wilson** thought that the Committee was fairly strongly in support of it, but not unanimously, simply because they wanted to keep the scope for electronic publication narrow to start with. Personally she was one that was against it. She reported that they had felt that if the next Congress wanted to broaden it then that was up to the next Congress, when there would have been six years to see how it was working, while other people on the Committee felt that ISBN should be allowed immediately.

**Glen** wondered if there was a certain amount of unease about the idea that the articles in an ISSN journal were always peer-reviewed but books might not be.

**Soreng** thought that a date to which this went back needed to be included, otherwise it could validate everything that was not published.

**McNeill** noted that that had already been dealt with in Art. 30 Prop. A, which had already been passed and it was going to be 1 January 2012.

**Penev** felt that the question was simple. If new names were allowed to be published in printed books, this should also be allowed for electronic books. He argued that publishing in journals should only be in ISSN journals, either printed or electronic.

**Herendeen** was thinking of *Systematic Botany Monographs*, which was an occasional series and there were other occasional series like that. He had checked with Warren [Wagner], who said that it currently had both an ISSN and an ISBN but, given the comments of a few minutes ago, that may end and it may only be ISBN as it was an occasional series. He thought it was important that those occasional series that were regarded as books or monographs should also be covered, so he was in favour of the proposal.

**Knapp** moved to a vote on Prop. B to replace in the Article just accepted “serial publication with an International Standard Serial Number (ISSN)” with the words “publication with an International Standard Serial Number (ISSN) or an International Standard Book Number (ISBN)”.

**Prop. B** was **accepted**.

**Prop. C** (92: 28: 1: 0).

**McNeill** moved on to Prop. C, which was establishing that it must be the final version by adding to Art. 29.3: “The content of a particular electronic publication must not be altered after it was first issued. Any such alterations are not themselves effectively published. Corrections or revisions must be issued separately to be effectively published.”

**Reveal** admired the Committee for putting something into the *Code* that he felt if it was only electronic no one could prove. He asked, how could one prove that a document had not been altered unless there was a paper copy? He was not certain whether it should be a formal Recommendation and therefore considered later, but his notion was that the indexing centres should make a paper copy kept at their archives, so that if there was ever a question of whether or not a document was altered it could be demonstrated that it was or was not.

**Janarthanam** continued on from Reveal’s comments adding that maybe it was a good idea if the date of publication was printed on the first page of each article. He felt that then it would be easy to check based on the property of the PDF file whether it was created later or not.

**Prud’homme van Reine** felt that the question was, how do you know that it was changed? He had heard from two colleagues in Utrecht who spoke about a paper, and they had the paper on their laps, but then they found out that the PDFs that were sent out by the author were different and it was impossible to know which one was the first.

**Karen Wilson** reported that the Committee had gone into immutability, as they headed the section in their report, quite extensively because they were very concerned about the matter. She added that that was one reason for using PDF because it included metadata, just as a camera image included metadata with digital images, like date of creation of the file and date of modification.

She suggested that anyone could rightclick on that kind of file and check what had been done to it and it was possible to include digital signatures that were like a paperbased signature in PDFs, including the PDF/A that was being recommended. At the same time, she admitted that there was no way to stop someone who was really determined to alter something; all you could try and do was make sure that the metadata was there so that it was possible to check whether someone had done the wrong thing.

She noted that the Committee was also specifying that the earlier, preliminary versions of journals that were put on the web should be clearly marked as such so that they were not taken as effectively published and, as she had already mentioned, some journals were already doing that—noting whether it was a preliminary or final version, which she felt was great.

She added that it was possible that Jim Reveal might like to suggest as a Recommendation that the indexing centres print a paper copy and keep it—she did not know what the indexing centres would think of that, but that was one way that would provide something on paper for checking if people were really worried about it.

**Gandhi** reported that as part of the International Plant Name Index project he encountered quite interesting instances and gave an example from the beginning of the previous year when he came across a situation where an orchid hybrid species was published. He had immediately brought to the notice of the author that the published epithet was orthographically incorrect and the Latin diagnosis only stated that the new species was intermediate between two existing species. Immediately the author put up another PDF file on the web, correcting the orthography and adding a somewhat grammatically errored [sic], Latin diagnosis that was nevertheless sufficient for validation. He had then provided a correct version of the Latin diagnosis and there was a third version of the PDF file. In this case, he noted that because of the personal interaction he knew that there were three different PDF versions on three different dates, but people who did not know the history of what went behind would have not known at all, so he totally supported the proposal.

**Lendemer** asked for a point of clarification from the Special Committee as to whether this meant that there was absolutely no way to falsify all the metadata associated with a PDF.

**Knapp** reiterated that the Section had already passed a proposal that had said we would allow electronic publication in PDF. She highlighted that this proposal was to add to the *Code* a restriction that anything that was altered was not effectively published.

**Lendemer** was asking how you would determine if it was altered.

**McNeill** felt that the answer had been given that in a large number of cases, he had the impression the vast majority of cases, that it was indeed possible. He added that there may be some situations where it was not, but felt that would not invalidate the importance of the Article.

**Sennikov** was afraid this was a general concern because as far as he understood the situation it was easy to modify whatever, either on paper or electronically. He argued that it was nearly impossible to be totally sure that the content was not amended or to know when it was amended if it was amended. He noted that it was technically possible to amend PDF and that it could be done either by a publisher or some skilful authors and the day of creation of PDF might be also modified or the file could be created and used somehow.

He added that it was also possible to falsify paper publications and to amend things should someone wish to—a part of paper sets could be reprinted and nobody would know when it had happened and it could be distributed along with original copies or instead of original copies.

So he felt that it was a general concern that was valid for any kind of publication, not only for electronic publications but also for paper publications. He concluded that it was either necessary to trust those who publish, publishers and authors, or to try to control them as much as possible. He thought that the proposal looked rather like a Recommendation.

**Barrie** called the question. [There was a sufficient majority in favour of voting.]

**Prop. C** was **accepted**.

### Recommendation 29A

**Prop. A** (87: 26: 4: 0).

**McNeill** moved on to Rec. 29A Prop. A, which had already been alluded to quite a few times as the Recommendation that electronic publication in PDF should comply with the PDF/A standard.

**Karen Wilson** wished to amend the proposal slightly because Malécot and others had pointed out that all that was needed to specify the PDF/A standard was the first part of the set of numbers, “ISO 19005”, and it would be better to delete the rest of it because it was a reference to the first version of PDF/A. This was to keep it general because there was already a second version, PDF/A-2.

**Prop. A** was **accepted** as amended by the proposer.

**Prop. B** (90: 22: 6: 0).

**McNeill** explained that the next proposal, Prop. B, was really necessary as a result of what had just been accepted because it was extending the recommendations in Rec. 29A, which currently went into some detail, on electronic publication and made it compatible with what had just passed.

**Norvell** wondered if this was an appropriate spot to raise the issue or spectre of the DOI number, which was a way of archiving and ensuring that the published PDF file, for instance, was always there. She reported using CrossRef and the DOI number.

**Penev** felt that the DOI was a good thing, but it did not have any relation to a repository; it could only link to a repository, but CrossRef was not a repository by itself. He added that the Recommendation was very good but very vague, so he thought some recommendation for repositories to respond to some criteria to be used for deposition, like ISO certification, was necessary. The term “trusted” had a meaning in the world of repositories according to him, so it was important to define more clearly what kind of repositories were strongly recommended to be used by the *Code*. He proposed an amendment to add the word “trusted” in front of “online digital repositories”. [The **amendment** was **seconded**.]

**McNeill** felt that using a word that was so general as “trusted” meant it was necessary to define what measure you have for trust and thought that if there was indeed a measure of trustedness that the ISO recognized then that should be given to help define the word.

**Penev** responded by saying that it was a very dynamic field and there was a special organization that dealt with elaboration of criteria for repositories, but he suggested a strict definition should say “ISO-certified repository”, which was very clear, but very few were ISO-certified now but it was likely to be more in the future.

**McNeill** suggested “for example an ISO-certified repository”.

**Penev** continued that PubMed Central, which was the largest repository, may not be ISO-certified, he was not sure as the process was just starting and as it was a Recommendation he did not see why not. He clarified that “trusted” had a meaning in the jargon of this world—“Trusted repository” meant a good repository according to some criteria.

**Demoulin** wondered if the partisans of electronic publication were so satisfied with the permanence of electronic media that they did not feel it would be wise to recommend deposition of some printed version.

**Knapp** pointed out that that was a different matter and what was currently being discussed was the amendment to trusted online digital repositories.

**Sennikov** suggested “recognized” instead of “trusted”.

**McNeill** noted that it had been said that “trusted” had a technical meaning.

[The **amendment** was **accepted**.]

**Flann** wanted to check if the new 29A.1 would entirely replace the previous Rec. 29A.1 meaning that the previous (a), “The printed and electronic versions are identical in content and pagination”, would be nowhere to be found in the *Code* anymore; also (c), “The electronic version is publicly available…”. She wondered if these issues were covered somewhere else by the other proposals for electronic publication or if it no longer mattered, because she actually quite liked those two points.

**McNeill** thought that it no longer mattered because if there was no hard copy version, the electronic version would be effectively published, so whichever was published first was effectively published and of course they should be the same. He thought that it ceased to be essential, as it was before in the Recommendation.

**Greuter** was always a bit uneasy when he saw matter in the *Code* that addressed people who would not normally read it. He elaborated that authors of botanical names and botanical papers would read the *Code* but publishers usually would not. He proposed an amendment to say that authors should preferably publish in publications that were archived, satisfying the … etc. He suggested the wording would be editorial. [The **amendment** was **seconded**.]

**Alvarado** thought that there were a lot of publishers, especially museums and universities, who published monographs and certain journals that were already dedicated to taxonomic or systematic work and in those cases he thought it would be good to say both “publishers or authors”, because some publishers were specialized in nomenclatural things.

[The **amendment** was **accepted**.]

**Challis** responded to Flann’s earlier concern, that in the proposal to amend Art. 29.1 there was an Art. 29.2, which defined online as “accessible electronically via the World Wide Web”.

**Demoulin** elaborated his point by saying that he still had several drawers of punch cards that if he wanted to read he had to go to a museum. He gave the example that the same data had been transferred to magnetic tapes and now his computing centre had withdrawn the lectors [i.e. readers] of magnetic tapes, it went on with floppy disks… He argued that the computer world was run by people that did not care about the past, as taxonomists were obliged to do, so he maintained that it was probably wise to also add that “The deposition of printed copies in libraries was also advisable” and proposed such an amendment.

**Knapp** clarified that this would add a number (c), which would say “Deposition of printed copies in libraries was also advisable”. [The **amendment** was **seconded**.]

**Dixon** questioned whether the Section had just voted against that before tea.

**Knapp** explained that this was a Recommendation, not a requirement of the *Code*.

**McNeill** added that the Section had voted against it as a rule.

**Dixon** thanked them for the clarification.

**Herendeen** agreed that the Section had voted against it as part of the rule and now thought that the Section should vote against it as a Recommendation.

**Penev** was a strong advocate for electronic publishing but he strongly supported the Recommendation. He did not think it was wrong if publishers wanted to deposit their copies in libraries, it was not an obligation but it was a very clear recommendation. In his opinion there was nothing wrong with that.

**Prado** pointed out that the word “publishers” had been deleted, which meant it was strongly recommended for the authors to deposit the printed copy.

**McNeill** thought that Prado’s point was that it would be publishers who would have to do the depositing and the reference to publishers had been removed.

**Demoulin** had no problem with taking out publishers as it was a recommendation to the author to make sure some printed copies existed, as it was part of the old registration proposals. He suggested that an author could even send them in PDF if he was sure that somebody would print them at the library. He though it was just a matter of the author trying to make sure that there were some printed copies somewhere that may survive any new crash in the internet and any new big virus.

**Lendemer** did not really see a problem with having this in because it would just parallel the Recommendations currently in Rec. 30A for ephemeral printed matter, which already basically said the same thing: a minimal number of printed copies should be put in libraries. He thought the Section should vote for it.

**Veldkamp** suggested that (c) be made to conform with (b), saying “in more than one area of the world and preferably on different continents”.

[This was considered a **friendly amendment**.]

**Herendeen** felt that we were back to imposing on libraries to deal with…

**Demoulin** interpolated that it was not imposing, exclaiming “My God!”

**Herendeen** continued that libraries would be receiving individual articles from authors and he argued that it was a major imposition on libraries to deal with them, while some of them may be well-staffed to deal with it, many others were not.

[The **amendment** was **accepted**.]

**Gereau** called the question. [There was a sufficient majority in favour of voting.]

**Prop. B** was **accepted** as amended.

### Article 30

[*The following discussion took place during the Fifth Session on Wednesday morning.*]

**Prop. A** (91: 22: 4: 0).

**McNeill** explained that Art. 30 Prop. A was to add a new Article to Art. 30: “*30.n*. Publication by distribution of electronic material does not constitute effective publication before 1 January 2013.” He suggested that the Committee would accept an amendment for an earlier date, as it was not going to have any negative effects if it were earlier. He added that normally when there was a date in the implementation or a change to the *Code*, it was usual to make it a date after the publication of the *Code* that had recently been published about a year after the Congress, so 1 January of the following year, which in this case would be 1 January 2013. He clarified that if the Section decided on this date, changed the date and then defeated Art. 29 Prop. A, the matter would just disappear.

**Alvarado** wanted to know when the *Code* was printed and when it was effective generally, because he thought that the best possible date would be at the same time when the general *Code* becomes effective and not to have a different time.

**McNeill** clarified that the *Code* became effective on the date stated in the *Code*. In other words, the *Code* was retroactive, so anything that was passed today that did not have a limiting date went back to 1753. In other words, the *Code* was retroactive in its application except where expressly limited. He went on that in the cases of new requirements in the *Code*, these were normally expressly limited to 1 January following publication of the *Code*. He repeated that 1 January 2013 would be the normal date for implementation of an Article that would have a negative effect, would require something new to be accomplished in order to, for example, validly publish a name. He explained that one had to give notice to people beforehand, where in this case it was not putting up a new hurdle, it was actually removing a hurdle, then the requirement for a later date was not so essential.

**May** supported the proposal to put a date in and thought it did make a difference as at the moment many journals that published taxonomic novelties were deliberately not issuing the electronic version prior to the print version because they were aware of the ramifications. He felt that it would allow time for people to digest the changes and perhaps adjust their publication methods.

**Ladiges** agreed with the sentiment of May, but quite honestly felt that those who were waiting to have electronic publication, assuming that that was passed, would find 1 January 2013 a very odd delay and she supported the previous suggestion of a much earlier date. She made a motion and amendment to change the date of 1 January 2013 to 1 August 2011. [The **amendment** was **seconded**.]

**Demoulin** was ready to vote for the Art. 29 proposal, which for him was the first time he would vote for electronic publication after having voted against it repeatedly every time it came up. It took him at least 12 years to be convinced that it was acceptable and he did not see why one and a half years more would be such an important matter. He supported Art. 30 Prop. A as it stood, because he did not see any reason to make special ruling here and certainly would oppose the amendment to move it back to August. He added that if the change was to 1 January 2012 that would be okay, but 1 August meant admitting publication by people who had disregarded the *Code*, because if there were things that have been electronically published next August, it was almost certain it would have been done by people who disregarded the *Code*. He opposed the amendment.

**Paton** proposed a friendly amendment to the amendment to change the date to 1 January 2012. [The **friendly amendment** was **accepted** by the proposer.]

**Stevens** called the question.

**Knapp** explained that the Section needed to vote whether to vote or not. She asked for all those in favour of voting on Prop. A of Art. 30…

**Stevens** corrected her that the vote was on the amendments.

**Knapp** apologized, saying that there were too many amendments and she was getting confused. She added that eventually the Section would drive her mad, unless people already thought she was mad… She clarified that the vote was on the proposal to amend the date to 1 January 2012 from 1 January 2013. [There was a sufficient majority in favour of voting and the **amendment** was **accepted**.] She reopened the floor for discussion on the proposal to amend Art. 30 as amended with the date of 1 January 2012.

**Sennikov** questioned the whole thing, if the starting date was needed here at all, despite the Section having just successfully moved the starting date. He noted that electronic publication was quite a new thing and everything was happening so fast nowadays he thought there were two questions. First, what would happen if the starting date was accepted? He argued that accepting the starting date meant that up to that day, everyone who was going to publish entirely electronically had to produce paper deposits somewhere, and the issue of those so-called reprints, which were to be somehow archived, and the future of these reprints, as was stressed several times already, was highly in doubt. The next question would remain—and the transitional period between when there were no electronic publications at all and when electronic publications were started to be allowed was very, very short. He wondered if the paper reprints needed to exist at all and suggested it would be a simpler and more pragmatic solution to get rid of those reprints, simply by removing the starting date of this provision, such names would be effectively published if there was no starting date in this particular case.

**Lendemer** argued that a starting date was essential, because there were so many publications and hundreds of names that would be effectively published if there was no starting date. He gave the example of *Mycologia* alone, which published new species in every issue and already posted its papers online specifically stating that they were intended to be published on the date, two months at least before they were printed in printed matter. Without a new date, all of those things would be valid. He gave other examples: *North American Fungi* and the *Lichenologist*, which came out months in advance online before they were printed. He was for keeping it the way it was.

**Dressler** strongly supported Greuter’s view and wanted to know why the Article was needed if it could be entered into Art. 29.

**McNeill** felt that was an editorial matter and noted that it was proposed in this way because of the way the *Code* was currently structured. He added that it may well be that when the Editorial Committee looked at it, it may be better to arrange it differently.

**Van Rijckevorsel** made a minor technical point that voting on this Article should be made contingent upon the other proposal passing, because if the other proposal was defeated and this was accepted, it would lead to a very odd situation.

**Turland** and **McNeill** both felt that would be purely editorial.

**Turland** added that if this passed and then Art. 29 Prop. A failed, then the wording just about to be voted on would become irrelevant and it would not be included in the *Code*.

**Knapp** thought that the Section had to trust the Editorial Committee to have brains. [Laughter.]

**Prado** noted that this would be putting in practice a new rule before the publication of the *Code* and wondered how it would be communicated to everybody that the *Code* accepted a new rule before its publication.

**McNeill** pointed out that communication would not be difficult because the report of the Congress would almost certainly appear in the October 2011 issue of *Taxon*, three months beforehand.

**Prado** wondered if *Taxon* had priority over the *Code*.

**McNeill** clarified that communication could be in any medium. The fact that this Congress agreed on this could be communicated on the Web; it could be communicated in any way.

**Prado** meant the application of the rule before the publication of the *Code*; he thought the *Code* was valid [only] after publication.

**McNeill** felt there may be a misunderstanding about when decisions made by the Congress became effective: a decision made by the Congress was effective immediately it was made. As Greuter rightly pointed out, 1 August was the first date it could possibly be, because this would not be approved until the final plenary session; at that point everything became effective.

**Knapp** clarified that the Section was voting on Prop. A to Art. 30 to add a new Article saying “Publication by distribution of electronic material does not constitute effective publication before 1 January 2012”.

**Prop. A** was **accepted** as amended.

**Bill Barker** wanted to add another Recommendation in relation to point (a) of Rec. 29A Prop. B because he was concerned about printed and electronic versions being produced on the same day and the consistency of those publications. He wished to add an additional Recommendation that effectively paralleled the previous (a) in relation to printed and electronic versions being produced on the same day. For him there would be no worry about choosing between versions as long as they were identical in content and pagination. He suggested producing another Recommendation or an amendment to this one, which may be not possible.

**Turland** drew attention to Art. 31 Prop. A and wondered if that covered what Bill Barker was suggesting: “In the absence of proof establishing some other date, the one appearing in the printed or electronic matter…”.

**McNeill** thought it was different but resolved the situation.

**Bill Barker** thought it could probably be sorted out with an amendment to Art. 31 Prop. B.

**Prop. B** (92: 23: 3: 0).

**McNeill** moved on to Art. 30 Prop. B, which was that “An electronic publication is not effectively published if there is evidence it is merely a preliminary version that was, or is to be, replaced by a version that the publisher considers final, in which case only that final version is effectively published”.

**Knapp** pointed out that the discussion was now back to rules and not Recommendations.

**Reveal** had a slight question in trivia: with the provision going into effect on 1 January 2012, he suggested it was possible the situation might occur where a journal had a pre-publication issue of a taxonomic paper without a clear indication because the publisher did not know what had been decided by the Section. He wondered if by “intent” it should be assumed that even though nothing was said, that paper was available electronically but was not a valid place of publication.

**Knapp** summarized in her own words what she thought Reveal had just said giving him the option to tell her if that was wrong. She thought he was suggesting that if there was an electronic publication that came out prior to 1 January 2012 that it would not be effectively published in the electronic version.

**Reveal** disagreed, and elaborated that he meant that if a paper was electronically available prior to 1 January 2012 without a clear statement or indication by the publisher that it was intended to be a final version in a printed journal, because the publisher just did not know what had been decided by the Section. His question was whether on 1 January that pre-publication issue would become a valid place of publication.

**McNeill** suggested one would apply the wording of the Article, which he felt was pretty clear: “if there was evidence that it was merely a preliminary version”. He gave the example that *Taxon* pre-published its articles but these were preliminary in the sense that it was possible for there to be wording changes when the final volume was put together. He argued that there was a clear practice even if it did not say “preliminary” on it that there was evidence that that was not—that these were facttracked to provide information but were not the final version.

**Reveal** concurred with the assessment of *Taxon* and numerous other journals but was just a bit concerned that some publications that were in electronic format before the first of the year may be considered valid before they were actually published. He reformulated his question as to how the word “intent” should be interpreted.

**McNeill** replied that nothing was validly published before 1 January [2012].

**Nic Lughadha** proposed what she hoped might be considered a friendly amendment to put something in to make clear that the evidence needed to be internal evidence, otherwise it could be separately in an e-mail. She suggested somebody could tell her that it was only a preliminary version and that was evidence but it was not generally available and not available at the point when the decision needed to be made. She suggested the intent would be to say that when looking at the paper it was possible to see the evidence rather than having to look at other sources.

**McNeill** suggested something like “evidence within the versions”.

**Nic Lughadha** agreed and added “within the publication”.

**Karen Wilson** thought it was reasonable as long as it was discussed so the Committee could hear if there were any problems.

**McNeill** did not think that the word “internal” would work as it had to be evidence from the versions.

**Turland** was worried about the idea.

**McNeill** appreciated the proposal but had some concerns about it, because he felt that in a printed work there was a beginning and end and the use of “internal evidence” was quite standard in the *Code* for judging those, but if a publisher for example had a policy that was publicly announced that only at a particular date the final version would be put together in volume form, this may not appear on any of the actual electronic versions and he wondered if that could create problems.

**Kirk** thought that the Section was trying to incorporate in the *Code* every possible minute detail of electronic publication and cover it before it happened. He asked whether the *Code* did not currently provide mechanisms for dealing with this eventuality. He was making a general comment, not just about internal versus external.

**McNeill** thought that Kirk was arguing against including internal evidence.

**Kirk** agreed.

**Knapp** concluded that it was no longer a friendly amendment. She started discussion on the amendment to include “internal” or “evidence within the publication itself” as part of the effective publication process. [The **amendment** was **seconded**.]

**May** pointed out that it included considering evidence within the publication, so it may be better to change the word “versions” to “the publication”.

**Lendemer** wondered if the reference was to internal evidence within an electronic publication, would this only apply to PDFs or would it apply to the website of the journal. He thought there was a relevant thing that he could use to illustrate the problems associated with what was being dealt with. He noted that it was unfortunate to always have to use mycological examples, and gave an example from the website of *Mycologia*. “Articles available in press at *Mycologia* are posted online as PDFs as they become available, usually four to eight weeks prior to the release of the final versions in print or online format. “In press” versions of papers have undergone peer review and copyediting. Although minor changes may be made to these papers before the final versions were printed or posted online, they were considered formally published, were searchable in PubMed, and may be cited using the unique DOI as the example cited below” and they cite an example. He wanted to know how that would be dealt with.

**Penev** supported inclusion of the word “internal”, because usually preprints either in HTML or PDF were marked or should be marked as preprints.

**Turland**, just in case the Section was not reading ahead, pointed out that Rec. 30A Prop. A from the Committee recommended that “Preliminary and final versions of the same electronic publication should be clearly indicated as such when they were first issued”.

**Nic Lughadha** had read ahead on this occasion and felt that that Recommendation did not quite do it for her. Regarding the question about whether a general statement on the website explaining a policy on preliminary versions would be adequate, she suggested it would be possible to say that there was evidence with or within the publication. She thought it was a potential issue that it should be published evidence that the publication was preliminary.

**McNeill** pointed out that, because this was an Article, it was quite important.

**Nic Lughadha** realized that.

**McNeill** requested clarification: was Nic Lughadha saying that there must be published evidence that a publication was merely a preliminary? He noted that such evidence could be published anywhere.

**Nic Lughadha** was floating that idea, but she preferred evidence and to address the particular question about the evidence on the website, she suggested evidence “with or within” or an editorial equivalent, so the “with” would cover the fact that the website on which the PDF was released explained the policy.

**McNeill** thought that “associated with or within” was fine.

**Malécot** referred to the discussion about internal evidence within the publication, and wondered about fast track articles, where there may not be page numbers, such as a *Taxon* fast track article with no page numbers. He wanted to know if this could be considered as internal evidence or was there a way to add this into that article.

**Herendeen** thought that would be clear evidence that it was a preliminary publication rather than a final version.

**Reveal** urged the Editorial Committee to add that as an Example for this Recommendation, that page numbers lacking in a pre-publication were evidence that it was not intended to be the final publication. He felt that an Example like that would solve an enormous number of problems.

**Knapp** made a note that the Editorial Committee should take that comment into consideration.

**Gandhi** strongly supported the amendment. As part of an indexing centre, he frequently got questions about validity of a particular name and as long as it was possible to see what kind of version it was, whether preliminary or final, it would be possible to help the IPNI users, so he felt it would be a tremendously useful amendment.

**Knapp** moved to a vote on the amendment to include something along the lines of “associated with or within the publication” to Prop. B to add a new Article to Art. 30. [The **amendment** was **accepted**.]

**Price** requested clarification, as she believed that it had been voted in Art. 29 that the content of an electronic article could not change, so in the first published version the content could not change, and one would assume that meant the mechanical content could not change, but one could also consider that the content was the actual document itself. She wondered, if a preliminary version was published and the content was changed in the second version, was it then invalidated?

**Karen Wilson** thought that McNeill and Turland could answer that. [Laughter.]

**McNeill** thought that if there was change, it was the final version that was accepted.

**Turland** added that the preliminary version would not be effectively published anyway, because it was preliminary.

**Price** read from Art. 29 Prop. C: “The content of a particular electronic publication must not be altered after it is first issued” and had interpreted that that was the preliminary version. She felt that one could interpret the content as being “you cannot change it electronically after it had been issued” but one could also interpret the content as being the actual written words, so you could not then change it, improve it, or make any additional editorial changes—content had two meanings.

**McNeill** took the point that it would have to be made clear that it was the content of an intended-to-be-final electronic publication.

**Turland** added that the content of an effectively published electronic publication, once it was effectively published, could not then be changed; before it was effectively published, it could.

**McNeill** though it was a very good point and would need to be dealt with editorially, and thanked Price for pointing it out.

**Prop. B** was **accepted** as amended.

**Prop. C** (12: 105: 2: 0) was ruled **rejected**.

**Zijlstra’s proposal**

**Knapp** introduced another proposal from the floor to add an Article to the *Code*.

**McNeill** added that it was a new proposal for a clause in Art. 30 in relation to electronic publication and read it out:. “Publication by distribution of electronic material does not constitute effective publication if it concerns a non-scientific online publication. A scientific (online) publication has a distinguished editorial board with extensive academic qualifications and a reviewing system for consideration of manuscripts that are submitted”. He noted that there was an Example given of *Dolomythos*. [The **proposal** was **seconded** and supported by four others because the proposer Gea Zijlstra was not present. The audience groaned.]

**Alvarado** thought that particular sentence that said a distinguished editorial board was a bit difficult to assess. He wondered how it would be possible to know who was distinguished and who was not and felt that was really a problem. [Applause.]

**Zijlstra’s proposal** was **rejected**.

### Recommendation 30A

**Prop. A** (84: 30: 5: 0).

**McNeill** addressed Rec. 30A Prop. A, still in the same series. He reported that the Rapporteurs made the comment that although it was certainly desirable, the implementation would not normally be within the control of authors of nomenclatural novelties. He noted that one of the points that was raised in some other earlier discussions was that the preliminary and final versions of the same electronic publication should be clearly indicated as such when they were first issued.

**Prop. A** was **accepted**.

**Prop. B** (89: 20: 11: 0).

**McNeill** noted that Rec. 30A Prop. B contained necessary changes to the existing Recommendation to take account of electronic publication.

**Applequist** believed that earlier a Recommendation had been approved, that publications that were published electronically should be deposited in at least 10 libraries throughout the world. She suggested these should be combined.

**McNeill** accepted that as an editorial suggestion.

**Prop. B** was **accepted**.

**Prop. C** (11: 106: 2: 0) was ruled **rejected**.

### Article 31

**Prop. A** (95: 21: 3: 0).

**McNeill** noted that the discussion had come to Art. 31, the last of the electronic publication proposals, and he introduced Prop. A, which was a modification necessary on the acceptance of Art. 29 Prop. A. He added that it was pretty well almost editorial.

**Penev** asked, if the dates of the electronic and printed publications were different, which was the date of the effective publication?

**McNeill** answered that it was the first.

**Annette Wilson** thought that if anybody was in any way uncertain about it, it could be amended by adding “printed or electronic, whichever was first” or words to that effect.

**McNeill** thought that was a suggestion for the Editorial Committee, because this Article was not actually about electronic publication per se, it was about publication in general, and there was no reason under this Article for there ever to be any printed or any electronic [version], as the case may be; it was just making sure that they were both covered by the same rule.

**Annette Wilson** felt that was fair enough.

**Malécot** thought it might be worth adding information to say this was not the date when the electronic media was downloaded, because that date may be printed or located on the file; an editorial comment to say it was not the date of download but was really the date of the article itself.

**Prop. A** was **accepted**.

**Prop. B** (92: 23: 5: 0).

**McNeill** introduced Prop. B, again from the Special Committee on Electronic Publication, as a new proposal that seemed to be desirable, having accepted electronic publication. He explained that the proposal was really pointing out that if there was no evidence for different dates of publication of parallel electronic and printed versions they should be treated as published on the same date.

**Bill Barker** thought this was where it was suggested that the desirability, or perhaps insistence, that pagination and content be the same be placed. He thought it was a different subject, though, and needed some advice on that. He suggested he would propose an amendment and then a decision could be made as to whether this was the correct place for it or not.

**Knapp** agreed that that was exactly the way to do it.

**Bill Barker** proposed an amendment that an extra sentence should be added that “The electronic and printed versions should have identical content and pagination”. [The **amendment** was **seconded**.]

**McNeill** pointed out that, as worded, it was a Recommendation and perhaps it should be looked at under Rec. 31A in a moment, unless it was just poorly worded and had been meant as a rule.

**Bill Barker** thought it should be an insistence, a rule, and changed “should”, to “must”.

**McNeill** reiterated that the amendment would be “In order to be effectively published the electronic and printed versions must be identical in content and pagination.”

**McNeill** suggested that the proposal should be taken up as a new proposal later.

**Turland** agreed that it did seem to be a slightly different thing, defining parallel electronic publications.

**McNeill** added that he thought it was quite a different thing, actually.

**Knapp** clarified that the proposal was not amended.

**Greuter** wondered whether this would be the place to make life easier for botanists. He suggested that it was not a rare event that no precedence in time was specified between electronic and printed versions and even that they were purposely issued on the same date. He continued that if it was always necessary to cite two places of publication because novelties appeared simultaneously in the electronic and printed version, even if paginated identically, it would involve two different citations. He thought it would be wise, or at least convenient, to minimize this by declaring that in such cases, for nomenclatural purposes, one of the two—his preference went to the printed one, as a traditionalist—would take precedence. He proposed to amend the proposal by adding “but when simultaneously published, for nomenclatural purposes, the printed version is treated as taking precedence”. [The **amendment** was **seconded**.]

**McNeill** reassured the Section that the Editorial Committee would make it clear.

**Gereau** wondered why, if there was an electronic and printed version of the same article in the same journal with the same pagination, identical in every respect including the same date, they should be considered separate publications. He argued that they were the same publication and there seemed no point in differentiating between them and saying that the printed version had some status separate from that of the electronic version as it was the same material. He saw no point to the amendment and felt it should be disregarded.

**McNeill** imagined that the wording to describe it being electronic and the wording to describe it as printed would be slightly different. He assumed there would at least be the word electronic or something or other appearing or an HTTP or some sort or reference as he could not imagine citing the electronic version identically to the other, but then added that maybe that would be the case.

**Gandhi** supported the amendment. He explained that for bibliographical purposes, whenever there were two different publications available for citation, he preferred to cite one and added that this had been the procedure whether it was the International [Plant Name] Index or even *Flora of North America*. If the amendment was accepted then he agreed that the printed version would be the one to be cited.

**Dorr** did not support Greuter’s proposed amendment. He gave the classical literature example of Humboldt, Bonpland and Kunth for South America, who published simultaneously a major work in folio and quarto; the species and the text were the same, the volumes were the same, but the paging was different. He continued that there was no way in traditional taxonomy—before electronic taxonomy—to distinguish which one of those two had priority but there had been no problem with people citing the paging for the quarto edition and the folio edition simultaneously and it did not encumber the literature all that much. He also mentioned that people had developed concordances so it was possible to figure out what the pagination was for the other depending upon which one you had accessible. He did not think that there would be a huge proliferation of people trying to cite both paper and electronic things at the same time.

**Applequist** thought that the real problem would come not from people over-citing the PDFs but if in some case the PDF was actually different from the print version. She suggested that maybe in these days of computerization that would never happen, but wondered what would happen if the protologue turned out to be a little different in one than in the other.

**Sennikov** was afraid that the recommendation may introduce a demand that someone had to look for a printed version of a certain periodical, which may be distributed nearly entirely electronically, so the printed versions may be properly printed, archived and so on but still be nearly inaccessible to the general public. He was afraid that this would be a dangerous demand.

**Penev** saw a possible confusion, related to that example: printed and electronic versions with the same content but different dates. Usually the electronic version had earlier dates, so which one should have precedence according to this amendment?

**McNeill** explained that this had no bearing on the precedence of a date; if it came out first—this would only apply to works that were published simultaneously—the earlier publication always had precedence.

**Lendemer** did not really see the problem here, and wondered why, if there were parallel electronic and printed versions that were identical and appeared on the same date, they would be cited differently. It seemed totally superfluous to him. He suggested that if they were cited differently then they would not be parallel in some manner and then this would not apply.

**Harley** suggested that to clarify the meaning, it would be better to put “published for nomenclatural purposes” after “precedence”.

**McNeill** thought that was editorial and that the substance should be considered.

**Harley** felt that it actually did mean something slightly different, the way it was.

**Redhead** was a little bit concerned that if one was given precedence over the other and then it was cited as a basionym, and the wrong one was cited, would that make the name [based on the basionym] invalid?

**Barkworth** suggested that if—and she really did not like it—precedence was given to one over the other, surely she felt that precedence should be given to the electronic version, which was more widely available. She added that if it was necessary to go and dig out the few printed versions, it was going to be a pain in… many places. [Laughter.]

**Knapp** noted that “ We Westerners always speak our minds, don’t we Mary?”

**Gandhi** responded to what Dorr had said about Humboldt by noting that even though the two parallel editions existed, it had been traditional to cite the quarto edition over the folio edition, except in Volume 4 for *Asteraceae*, where the folio edition was effectively published in 1818, although the title page showed 1820. His other minor comment was that when both electronic and printed versions were available, even if one were to cite the electronic version, it did not matter as no new combination would be invalidated because anything could be corrected as a bibliographic error. He felt that there would not be any question of invalidating any new combination just because one cited the secondary version.

**Janarthanam** agreed with an earlier speaker that it was redundant because, for example, if an article was published in *Taxon* it was the same journal, the pagination was the same, it did not matter which was cited. In his mind it was not required and he opposed it.

**Cafferty** was not sure if he had misunderstood but he thought precedence was a red herring. He thought Greuter was really trying to just make life easier so it was not necessary to cite two places of publication. He suggested to add, after “nomenclatural purposes”, “citation of one or another was acceptable”. [This was considered a **friendly amendment**.]

**Kellermann** did not think that citation was really a problem but just in the rare case that the PDF was for some reason different to the printed edition, he felt that that was what Bill Barker had wanted to deal with. He thought that Greuter’s first amendment would cover this issue as well—because one would have precedence, so if one of them was different then it would always be possible to know which one was the right one.

**Turland** noted that it would not be parallel then.

**Kellermann** clarified that he meant if they were published at the same date, same pagination, but for some printer reason the PDF had a line left out or the PDF looked different to the printed version.

**Dhabe** noted that most electronic journals did not go for a printed version, one could print as many copies and it was readily available free of cost. He argued that precedence should not be given to the printed version, but to the electronic one.

**May** thought that because they had been said to be parallel meant that they were the same. He wondered what was going on if the possibility was introduced of citing them differently. He argued that if something was in *Mycotaxon* Issue 111, page 113, it was undesirable to have to put after that “printed version”, “electronic version”, URL this and that: it was published in *Mycotaxon* 111: 113 and that was it. He felt that if two different ways of citing the versions were indicated here, that would open up the way to unnecessary complications.

**Saarela** agreed with the previous speaker, wanting to know what the difference was between “issued in parallel” and “simultaneously published”, in this context. To him, they meant the same thing.

**McNeill** agreed that it was an unnecessary addition.

**Lendemer** wondered, if the fact that the discussion was about nomenclature was ignored for a second, how this differed from citing electronic and printed versions of journals. He argued that they were not cited different ways if they were identical and appeared on the same date, so he just did not see how it differed.

**Barrie** called the question. [Applause.]

**Knapp** moved to a vote on whether to vote on the amendment. [There was a sufficient majority in favour of voting.]

[The **amendment** was **rejected**.]

**Knapp** returned the discussion to Prop. B in its original state.

**Van Rijckevorsel** questioned if the entire proposal was necessary. It looked to him that something was effectively published only once and whatever version was published first was the one that was effectively published and if they were published together they were still only the same thing. He did not think it made sense to have a provision that gave such details, so if it was left out then just the date of publication was enough, as in the Prop. A.

**Marhold** thought it was useful because if the provision was not there somebody could say “Well, there was no evidence, so we can put in doubt whether it was published on the same date or not”. With this provision he argued that if there was no evidence it had to be taken as published on the same day and any doubt would be erased.

**Soreng** commented that it seemed like when it came out as electronic first and then as hard copy there was no way of knowing whether the two were different until somebody scrutinized the whole thing in comparison and if there was a slight difference, it may be hard to find.

**McNeill** clarified that did not apply to this particular clause, because it referred to things issued in parallel.

**Prop. B** was **accepted**.

**Bill Barker’s proposal**

**Bill Barker** was not sure whether his proposal should be a rule or a Recommendation. He put it up as a rule first but was prepared to have a friendly amendment to change it to a Recommendation, if that was the way the discussion went. He proposed a new Article: “For effective publication electronic and printed versions published on the same date must have identical content and pagination”. [The **proposal** was **supported** by five seconders.]

**McNeill** asked whether that meant that if it was published on the same date and the versions were not identical, they were not effectively published, whereas if it was the next day, the second one would be effectively published.

**Bill Barker** clarified that the precedence issue was to do with the date, if they were not the same day then there was no issue.

**Knapp** asked if this would be a new Article in Art. 31.

**Turland** thought that was open to discussion and wished to clarify something before the discussion started. He wanted to know if the intention of the proposer was that if electronic and printed versions were published on the same date and they did not have identical content and pagination, then neither was effectively published.

**Bill Barker** was going to say that there would have to be a necessary rider to this, which he did not have prepared, but it fitted with what Greuter was saying, that there probably needed to be a choice of one over the other. He suggested proposing something so it could be discussed.

**McNeill** agreed that something would have to have to be added, because at the moment neither of the versions would be effectively published.

[*Discussion between the Rapporteurs and proposer to arrive at the right concept, agreeing that the wording would be solved editorially.*]

They suggested “Electronic and printed versions published on the same date must have identical content. In the event that they differ in either content or pagination, the electronic version has precedence.” [This new version of the new Article was **supported** by four seconders.]

**Kirk** wondered what would happen if the content was the same but the pagination was different.

**Bill Barker** suggested a choice would still need to be made because it was undesirable to have confusion over pagination, so he thought it would be desirable to still have the precedence.

**Saarela** asked, if the electronic version did not have pagination, would it not be considered a copy that was not final, and thus it could not be effectively published?

**Paton** thought it seemed very messy. He felt it created doubt, as people would have to try comparing paper and electronic versions. He thought it was fine as a Recommendation but would oppose it being an Article.

**Harley** thought it was quite wrong as an Article and seemed much better as a Recommendation. He noted that it meant that if you left out one full stop in one version then that would create the problem.

**Briggs** thought it would result in sending people on a search to cross-check a printed version with the electronic one in a way that was not helpful.

**Lendemer** did not understand why if a printed version and an electronic version were being produced on the same date, they would be keyed in differently such that they would be different. He wondered if there really was a problem if both printed and electronic versions were issued on the same date and they were not identical in content and pagination; would there really be a problem with both of them not being effectively published? He queried whether that was the same thing as saying if you screw up publication now you have to try again later.

**Nic Lughadha** responded by saying that she did not think it was the same thing, because you would be saying I have to look and check if they were both the same and compare them in order to be sure. She felt this was adding work to the botanist or indexer.

**Miller** asked if you ignored differences in pagination, if two things come out and there were significant differences in content, why they were not both effectively published and it would be up to the choice of future authors who wanted to cite and refer to them which one they chose. He did not see that this accomplished anything and wondered if the issue related to little differences about the way type was set or pages were numbered.

**Greuter** was under the impression that there had been some confusion because two completely different versions had been displayed. The first one was indeed completely unacceptable to him, whereas he felt that the second one had quite some merit. He suggested that it should be simplified to read: “In the event that two simultaneously published electronic and printed versions differ, the electronic version takes precedence”. As he understood it, it reflected the intent of the proposer. He explained if you have two different versions and they were simultaneous, you want to know which one counts, so that we do not have to cite two different pages, or in the case of severe discrepancies to resort to the first amender’s rule or something like that—say if new names were spelled differently in the electronic and in the printed version published simultaneously.

**McNeill** confirmed that the first sentence disappeared.

[The proposer **accepted** that as a **friendly amendment**.]

**Buck** recognized that it was an editorial thing but was really unhappy with the word “simultaneously” rather than “on the same date”. He pointed out that in the past it was not necessary to worry about what hour something came out but with electronic versions, it was possible to know to the second when something came out, but not possible to know the hour somebody dropped it in the mail at the post office. He thought that by using “the same date” it would avoid somebody trying to do hourly effective publication, one over the other. [Laughter.]

[The proposer also **accepted** that as a **friendly amendment**.]

**Potgieter** made the comment that often printed things were in black and white and electronic things were in colour. She wondered if that made it different content and if so, then she thought that the electronic one really should take precedence.

**Whitbread** noted that if there was an electronic and hard copy published simultaneously they would have different IS[B/S]N numbers, so would effectively be different publications. He thought it was necessary to differentiate between the hard copy publication distributed electronically and electronic.

**Knapp** asked whether he was speaking for or against the Article.

**Whitbread** clarified he was for the removal of the first part.

**Sennikov** queried the practical applicability of the rule depending on what the difference was. If it was a single word, he argued that it looked cumbersome. He added that the rule again urged looking for minor differences in order to downshift the value of one of the versions when maybe the difference was not at all significant.

**Stevens** called the question. [There was a sufficient majority in favour of voting.]

**Knapp** clarified that the vote was on a new Article as part of Art. 31—a rule and not a Recommendation: “In the event that the electronic and printed versions were published on the same date and differ in either content or pagination, the electronic version had precedence”.

**Unknown speaker** queried whether a 60% majority was needed for it to be adopted.

**McNeill** agreed that was definitely the case.

**Turland** called for a card vote.

**Knapp** had counted 70 to 45, which was not a 60% majority, so it went to a card vote. She added that a vote “yes” was to include this as an Article of the *Code* and a vote “no” was to not include this as an Article of the *Code*. [Time passed.] She reported the results of the card vote: the “yes” votes were 257 and the “no” votes were 250. Because this was an addition of an Article to the *Code* and required amendment to the *Code*, a 60% majority was needed so the proposal failed.

**Bill Barker’s proposal** was **rejected** on a **card vote** (257:250; 50.7%).

[*Here the record reverts to the normal sequence of events.*]

### Recommendation 31A

**Prop. A** (11: 39: *66: 0).

**McNeill** reported that the mail vote on Rec. 31A was quite heavily towards the Editorial Committee because of the comments of the Rapporteurs that it might be improved by deleting the text following the first occurrence of the word “publication”. He added that the Recommendation simply advised prompt distribution of published material rather than going into the detail of what would happen if it was not possible to publish promptly.

**Greuter** questioned whether this was a recommendation to the users of the *Code* or to the editors or publishers. He felt it was absolutely pointless to put into the *Code* Recommendations that did not concern the taxonomic community.

**Prop. A** was **rejected**.

### Article 32

**Prop. A** (29: 64: 14: 1).

Prop. A was, **McNeill** felt, quite an important or interesting proposal. He read what the Rapporteurs had said, that it “would make a fundamental change to the *Code* by removing the concept of valid publication above the rank of family”. He added that it created all sorts of questions because then you were trying to regulate names above the rank of family, which the *Code* did not really consider to be names. The Rapporteurs had made comments as to how that might be dealt with. He explained that it also meant that the rules on the formation of names above the rank of family would be governing names that were not validly published. He suggested that that could be overcome by simply changing the requirement for valid publication of names above the rank of family, so that they were not subject to the regular provisions of Art. 32, but were simply restricted by the provisions governing the form that they took. The argument for this from the proposer [Brummitt], who was regrettably not present, was that the principle of priority did not apply above the rank of family. This would mean that one could choose whichever name one liked, so why then require that such names met all the other requirements of the *Code*? The Rapporteurs did not make a specific suggestion as to how the proposal might be modified in a way that it would retain valid publication of names above the rank of family, while still excluding them from the requirements of Latin diagnosis and so forth as contained in Art. 32 and subsequent Articles. He reported that the mail vote was quite substantially negative: 29 for, 64 against.

**Demoulin** often agreed with his friend Brummitt, but in this case he did not see the logic in saying that because there was no priority it was possible to choose any name you want, for example without having them published with the descriptions. He wondered how to choose if there were no elements on which to base your choice.

**McNeill** explained that apart from descriptive names, most names above the rank of the family were automatically typified.

**Greuter** asked whether the Rapporteur’s suggestion was an amendment to the proposal.

**McNeill** clarified that they had not proposed an amendment but were drawing the attention of the Section to the fact that if people wished to pursue the proposal, there would be a need for an amendment that made sense. He highlighted that should be done at this point in the proceedings. [No-one did so.]

**Prop. A** was **rejected**.

**Prop. B** (36: 39: 35: 0).

**McNeill** noted that Prop. B was an attempt to clarify what was meant by the requirement that scientific names be in the letters of the Latin alphabet. It was a change to what was agreed in Vienna in an attempt to clarify what the letters of the Latin alphabet actually are.

**Karen Wilson** had missed the discussion about the earlier part, but suggested that the definition of the alphabet would be better in the Glossary. [Laughter.]

**McNeill** felt that it would not fit in the Glossary as it was originally defined as the intention was to try to keep the Glossary as integral with the rest of the *Code* as possible. As an aside he outlined that there had been a time when the *Code* had a number of Appendices that discussed things like typification, but what tended to happen was the guide to the determination of types diverged from what was actually in the *Code* about types. He explained that nothing had been included in the Glossary that was not virtually the words of the *Code*, except specifically where the word was not defined in the *Code* but where the dictionary meanings were quite diverse or quite different, when the dictionary meaning that was relevant to the *Code* was inserted. These entries were also indicated in the Glossary. He therefore felt that it would be a little different to put something as nominal as the alphabet in the Glossary. However, he suggested it could go in as a Note or probably a footnote.

**Demoulin** had never had a real problem with this issue, but, as he was often consulted on orthographic matters and involved in discussion about it, he was sometimes surprised to discover that people may have very strange ideas about orthography. He felt it was better to have things stated clearly and fully spelled out in the *Code* and thought the proposal did no harm.

**Magill** wondered what effect the proposal would have on the use of diaeresis and hyphens and names.

**McNeill** explained that they were covered elsewhere and would not change.

**Greuter** felt that the proposal in itself would be harmless if the Article was harmless because it would add precision. But he argued that the Article itself was so blatantly unacceptable that he would never apply it, and opposed making it even more precise in its awkwardness. The reason he gave was that this would confirm that neither a hyphen nor a period was a letter of the Latin alphabet. He pointed out that many new combinations were validly published with the generic name abbreviated—there was a period in it—but under this Article, if taken as literally as proposed and made even more precise, those names would not be validly published. Similarly, he noted that many of Linnaeus’s names were published with epithets abbreviated, with a period at the end, and according to the Article those Linnaean names that were universally expanded would not be validly published. He encouraged the Section to vote the proposal down because otherwise he felt the Section would really document taking the Article seriously.

**McNeill** added that the comment that Greuter had just made was reflected in proposals that were still to be discussed, mostly from Paul van Rijckevorsel, referring to just those points about periods. He thought that these were matters that could be addressed quite simply by indicating that abbreviations and so forth were not excluded. He noted that the proposal initially was accepted in Vienna because the concern was that there was nothing in the *Code* to preclude the use of a Cyrillic alphabet, for example.

**Prop. B** was **rejected**.

**Prop. C** (27: 69: 15: 0) was **withdrawn**.

**Prop. D** (48: 42: 14: 0).

**McNeill** introduced Prop. D, which was dealing with situations that were quite acceptable, indicating that just because the letters of the Latin alphabet were being used for names did not exclude situations of this sort [as described in the proposal]. He reported that it had very marginal support in the mail vote: 48 to 42.

**Greuter** agreed that the proposal certainly mended some of the shortcomings of the Vienna wording but he wondered if this proposal or anything else in the *Code* permitted expanding of abbreviation as was customarily done.

**Van Rijckevorsel** answered that as far as he knew, there was only one provision, namely Art. 33.1. He felt that was rather skimpy so had made a few additional proposals, which were defeated.

**Greuter** asked if the proposer would accept a friendly amendment to add the phrase “abbreviations were to be expanded” at the end of the proposed text. [It was not accepted as a **friendly amendment** so was proposed as a formal **amendment** and **seconded**.]

**Gereau** felt that the amendment seemed to have absolutely nothing to do with the rest of the proposal, which was talking about typographic signs in the arrangement of taxa. He assumed by “abbreviations” it meant things like Linnaean signs that could be expanded to *capillus-veneris* etc. He reiterated that there was no relationship between the amendment and the rest of the proposal and the amendment should not be included.

**Govaerts** wondered if it would also mean that epithets like *st-johnii*, where the “Saint” was abbreviated, would also have to be expanded.

**Knapp** thought that the proposer of the amendment was saying that that would be true.

**Govaerts** added that that was not normal practice and estimated that there were perhaps more than 100 names like that.

**Challis** agreed with Gereau that the amendment did not seem to fit the original proposal, which was to do with rank-denoting terms.

**Barrie** noted that the Editorial Committee would be within its power to split it into two notes if it seemed they did not make sense together. He expanded that it was possible to have both together for the vote, but it was not necessarily how it would appear in the *Code*, if it made more sense he suggested it could be included as Note 1 and Note 2.

[The **amendment** was **rejected**.]

**Prop. D** was **accepted**.

**Prop. E** (79: 16: 11: 0).

[*The following comment, pertaining to Art. 32 Prop. E was made during the Sixth Session on Wednesday afternoon with discussion on Art. 33 Prop. C.*]

**Turland** noted that the proposal was almost editorial. “Almost” because it was something that was implicit in the way the *Code* was applied, so he did not think the proposal really changed the meaning at all, but when something that was implicit was being made explicit he felt it would be nice to have the endorsement of the Section, just to make it obvious.

**Prop. E** was **accepted**.

[*Here the record reverts to the normal sequence of events.*]

**Prop. F** (26: 29: 48: 0).

**McNeill** moved on to Art. 32 Prop. F, which was a reference. The comments of the Rapporteurs were that this was a helpful reference, although it would be unnecessary if Art. 41 Prop. A was accepted, which of course had not yet been dealt with. He reported that the vote was 26 for, 29 against and 48 to refer it to the Editorial Committee.

**Reveal** [one of the proposers] suggested that the matter could be referred directly to the Editorial Committee.

**Prop. F** was referred to the **Editorial Committee**.

**Prop. G** (59: 27: 22: 0).

**McNeill** noted that Prop. G was linked to Art. 7 Prop. C, which had been accepted the day before, and the proposal would make clear what most had understood to be the case, that a previous effective description or diagnosis was only relevant when no descriptive material was provided in the protologue. He reported that it had received fairly good support: 59 for and 27 against.

**Sennikov** thought that the change may be potentially harmful in such cases when there was no extant original material connected with a major description provided by the author who published the name. He suggested that it may happen that the material associated with descriptions referred to in the protologue may be extant and may be highly useful and even convenient to be designated as types and it was possible that the emendation would eliminate this useful possibility.

**McNeill** commented that it would be quite an extraordinary interpretation of the vision of validation by a previously effectively published description. He thought it had been assumed that where there was a description in the validating publication, that was the validating description and that typification by an earlier description was only a choice when there had not been descriptive materials [in the validating publication]. He agreed that it was not spelled out, but his experience was that it had not been interpreted differently.

**Greuter** thought the question that a type could be a description had been voted out of the *Code* in perhaps even St Petersburg, then Leningrad. The proposal linked to that concept, which was long gone from the *Code*.

**Prop. G** was **accepted**.

**Prop. H** (40: 55: 11: 0) and **Prop. I** (23: 71: 10: 0) were alternatives.

[*The following discussion, pertaining to Art. 32 Prop. H and I, took place during the Ninth Session on Friday morning with Art. 46.*]

**McNeill** introduced the two proposals: Art. 32 Prop. H and Prop. I, which were essentially alternatives. He read out what the Rapporteurs had written: “Prop. H would establish what many have assumed the *Code* implied and upon which Art. 46 Ex. 10 is predicated, namely that when, in the protologue, part of the descriptive material is ascribed to the publishing author and part to the author to whom the name is ascribed, then both the name and the validating description are to be attributed to the author to whom the name is ascribed, with consequent implications for typification. Article 46 Prop. I is essentially the same proposal, but refers only to the attribution of the name, whereas… Art. 32 Prop. I is an alternative that leaves the validating description open to choice even when both the name and some descriptive material is ascribed to someone other than the publishing author”. This had, as they had noted, “the advantage of greater flexibility in lectotypification at the expense of a less consistent approach”.

He noted that there were associated proposals in Art. 46, which, if adopted, would ensure that there would be no change to the current practice on authorship. He summarized that this was essentially the type of situation in which a name was ascribed to an author other than the publishing author and there was also a description ascribed to that author, but the publishing author added some comments or descriptive material of his or her own.

**Knapp** requested clarification as to whether the Rapporteur-général was suggesting that the two proposals be treated as alternatives, giving the Section the option to decide which was preferable and then move to accept.

**McNeill** felt it might be useful to do it that way or simply discuss Prop. H first, whichever was preferred.

**Funk** wondered why both proposals had received so many “no” votes in the mail vote.

**McNeill** explained what the votes were: 40 “yes” for H, 55 against; 23 “yes” for I, 71 against, and he agreed that they were both predominantly negative votes.

**Knapp** opened the floor for discussion and comment on Prop. H.

**Greuter** suggested that both proposals be discussed jointly, since they were alternatives.

**Knapp** agreed that her suggestion was that they be treated as alternatives. She reminded the Section that if there were two alternatives, the vote would be a simple majority as to which one was chosen and then the vote to amend the *Code* was a 60% majority.

**Greuter** voiced a clear preference for anything that allowed for flexibility in type choice. He felt this was very important and it would be destabilizing not to allow that, because the change would be retroactive and it might overthrow past lectotypifications done in very good faith.

His second point was that this needed to be kept separate from the question of author citation. This concerned the possibility to typify from a given context. Author citations were a technicality to allow establishment of where the name came from. Author citation need not imply that types came from the cited author; it implied that they came from a cited publication.

In his opinion, these two things were unrelated and it was important to retain flexibility. He added that if mechanical decisions were made that the author to be cited is the author of the validating description or validating descriptive matter, this may lead to very awkward situations. For instance, a name taken up from a herbarium label validated by a later author with a clear description, a formal description, and the type is cited from the original label, on which there is a mention of “flowers yellow”. He asked if it is impossible in such a case that the name is ascribed to the collecting author, who coined it on the label.

**McNeill** interrupted that he thought the discussion was moving to Art. 46.

**Greuter** apologized. [Laughter.]

**Knapp** lamented the slippage in Articles.

**Gandhi** had come across situations like this, especially in the treatment on *Flora of North America* by John Torrey and Asa Gray. He had discussed several examples with McNeill, Nicolson, and Greuter wherein Nuttall provided the name and description and in several cases John Torrey and Asa Gray had added their comments. Then it became a question whether to typify the name only with Nuttall’s collection or whatever was cited within that protologue. At that time the decision was taken that whatever was cited within the protologue should be considered towards typification. He preferred proposal H.

**Knapp** moved to a vote between Prop. H and Prop. I to choose between the two with a simple majority, and the Section would move on to discuss whichever one had been chosen as a proposal to amend the *Code*. [**Prop. I** was chosen over **H**.]

**Knapp** opened the floor for comment or discussion of Prop. I.

**Sennikov** proposed the amendment to remove “not all by the same author or authors”, because authorship was no longer relevant. [This was not accepted as a **friendly amendment**.]

**Barrie** preferred the original proposal and did not favour the amendment.

[The **amendment** was **rejected**.]

**Gandhi** was somewhat concerned that if the proposal was accepted, if any section of the descriptive material was adequate for validation, then who would be the author, the publishing author or the ascribed author?

**McNeill** noted that the proposers had addressed that under Art. 46.

**Gereau** felt that ambiguity in all nomenclatural matters should be avoided and the proposal introduced tremendous uncertainty as to the validation of the species name and should really be voted down.

**McNeill** requested an explanation from Gereau as to why it would make a name not validly published, as he felt there was no suggestion that there was no description there.

**Gereau** was not indicating that it would not be validly published, but the ambiguity in the nature of its validation was which descriptive statement, and being able to choose among a number of them, was highly undesirable, as he felt this should be more restricted.

**Perry** suggested that, in that case, he should have voted for Prop. H, not I.

**McNeill** thought he probably did.

**Gereau** agreed absolutely.

**Prop. I** was **rejected**.

[*Here the record reverts to the normal sequence of events.*]

**Prop. J** (8: 27: 72: 0) was ruled referred to the **Editorial Committee**.

**Prop. K** (6: 80: 22: 1) was **withdrawn**.

**Prud’homme van Reine** had been asked to bring two small proposals from the floor, additions to Art. 32.

**McNeill** thought that proposals from the floor, which immediately and naturally followed something already discussed and addressing similar issues to those that had been immediately dealt with, should be taken in the sequence. He added that other more general ones would be better taken together under other business on the last day. He pointed out that it was much better when new material was prepared and people had it in writing or it could be displayed on the board beforehand.

**Woelkerling’s proposal**

**Prud’homme van Reine** introduced the first proposal dealing with the definition in Art. 32.2: “A diagnosis of a taxon was a statement…” He had been asked to propose that at the end of the sentence “of the same rank” be added. The proposal came from Bill Woelkerling, who worked with coralline algae, where it often happened that the differences to other taxa were given to quite other subgenera or genera and not to species when he was speaking about species.

**Sennikov** was not sure what the consequence of this would be if taken literally. He mentioned that recently a few new taxa were described from Turkey and the authors distinguished their new species from subspecies of the other species. This was not the only case that he had seen of this kind where in principle the taxa were compared at different ranks, but at the same time it was still a different species. He felt that the situation was awkward and confusing, but currently it was acceptable and would probably not be acceptable with the emendation.

**McNeill** summarized to make sure he had understood correctly: there was a description, the diagnosis distinguished it from another species but referred specifically to a subspecies of that other species.

**Sennikov** confirmed that it was referenced to the subspecies of the other species. So a new species was different from the subspecies of the other species.

**McNeill** thought that implied from the other species as a whole.

**Buck** pointed out that the proposal had not been seconded.

**Knapp** apologized for the oversight. [The **proposal** was **seconded** and **supported** by three others.]

**Turland** added to Sennikov’s comment that it was very common to have a diagnosis saying, for example, a new variety or subspecies was similar to the type, presumably meaning either that of an autonymic taxon or that of the species. He thought that this could theoretically cause problems.

**Govaerts** had the same comment as he had yesterday: again, as the proposal was retroactive, any taxon that had been described in the past, which compared to taxa at all the ranks, the name would be invalid. He estimated that it could affect tens of thousands of species.

**McNeill** thought that was an exaggeration, but agreed that the fact remained as correct. He thought that the situations that people had described did exist and could be destructive although he noted that they were still the exception, not the rule.

**Cameron** felt that the proposal was unnecessarily prescriptive, as a taxon may often need to be distinguished from taxa that may in the course of revisionary work be recognized at a rank relevant to the proposed taxon. He understood Bill Woelkerling’s concern, but it seemed to him that there may be varieties that in future may be seen as subspecies. In these situations he felt it was quite appropriate to make reference to how the new taxon was going to be distinguished from things that eventually may prove to be of an appropriate rank for making the comparison. Despite appreciating the reasons why Bill Woelkerling had made the proposal he was actually not in favour of it.

**Woelkerling’s proposal** was **rejected**.

**Prud’homme van Reine’s proposal**

**Prud’homme van Reine** introduced another proposal from the floor about the binding decisions that were given about whether a description or a diagnosis satisfied the requirements of Art. 32.1(d). He argued that if the binding decisions were not listed then they had no use and proposed having them listed in an Appendix. He proposed adding this to Art. 32. [The **proposal** was **seconded** and **supported** by three others.]

**Barrie** thought the proposal made a lot of sense as it was necessary to have the decisions somewhere where people could see them and he felt this was the most logical place. He noted that the General Committee was working on these. He had assumed that they would not go into an Appendix, but agreed that it really was necessary to have something in the *Code* that allowed placing them in an Appendix.

**Marhold** also found it useful and pointed out that as the Section had voted that the Appendices might be electronic only, this would not involve any extension of the *Code* or do harm and it would certainly be useful.

**Gandhi** also wanted to support this proposal because many people contacted him wondering where to submit their problem, to which committee, which address? He agreed that having them listed in an Appendix would be very useful.

**Wiersema** wondered if it was it convenient to also deal with the situation under Art. 53.5, confusingly similar names, which were binding decisions that were not listed anywhere.

**Redhead** wondered how many of these there were—aside from the recent one that he had submitted, which was on *Ascomycota*—and whether the previously ruled ones would be put into an Appendix, if the proposal was accepted.

**McNeill** explained that this was a new provision that appeared in the *Code* in Vienna, so by definition there were very few. The General Committee had been quite exercised with regard to some of the proposals, particularly some proposals from the Nomenclature Committee for Vascular Plants, where there were questions as to what extent that Committee had actually applied the criterion of description or diagnosis, so there were a small number. He presumed that there would be many more, as had happened before—for six years there was only one rejected name and then all of a sudden it became a substantial Appendix. He agreed that it was good to start at the beginning.

**Knapp** summarized that the proposal was to amend Art. 32.4 to provide that these binding decisions would be put into an Appendix. She assured the Section that the Editorial Committee would make the wording sensible.

**Prud’homme van Reine’s proposal** was **accepted**.

[*The following discussion, pertaining to a new proposal by Cameron & Prud’homme van Reine concerning Art. 32, took place during the Tenth Session on Friday afternoon.*]

**Cameron & Prud’homme van Reine’s proposal**

[Amend Art. 32.2 as follows:

“A diagnosis of a taxon is a statement of that which in the opinion of its author distinguishes the taxon from allied taxa of the same rank or which, in the opinion of the author, can be reasonably interpreted to be of equivalent rank.”

i.e. replace “other” with “allied” and add “of the same rank or which, in the opinion of the author, can be reasonably interpreted to be of equivalent rank”.]

[The **proposal** was **seconded** and **supported** by three others.]

**Cameron** noted that the proposal might look suspiciously similar to a proposal that was put from the floor by Prud’homme van Reine on Tuesday [Woelkerling’s proposal], with which he had had a problem of interpretation. He had misinterpreted the key issue and considered it overly prescriptive, and he thought that the body of opinion agreed. The suggestion then was to simply add the words “of the same taxon”. In the meantime he reported that he had spoken to Woelkerling, who had had a lot of experience with the diagnoses, particularly with the coralline algae. Cameron explained that, when he had voted against the initial proposal, he had in the back of his mind that the overly prescriptive nature of actually enshrining in the *Code* itself the necessity that the taxon be distinguished from another taxon of the same rank was not taking account of the fact that many different interpretations of rank had varied over the years, and that in the course of revisionary work people were often comparing something with an entity that did not yet have the formal rank that would be equivalent to the new taxon.

Having discussed the original considerations from Woelkerling, Cameron had realized that there was a second, quite additional element that it was sound taxonomic practice, and actually implicit within the *Code* and in practice regarding diagnoses, that a diagnosis was only useful as a diagnosis if the taxon was compared with closely allied taxa as well as taxa of the same rank or taxa that could reasonably be interpreted to be of equivalent rank.

The proposal had been carefully reworded and Woelkerling thoroughly supported the new version.

**Gereau** thought that the new version was no improvement, and was still overly prescriptive, unenforceable and undesirable. He felt that if he found a new species that could best be compared to a superficially similar subspecies of another species, with which it could easily be confused but by more taxonomically important characters constituted a new species, then in his opinion it was not of equivalent rank and it should be compared, and he should have the right to do so.

**Veldkamp** had a problem with the word “allied” because it suggested that phylogenetic research was necessary to be able to write a diagnosis, and he felt that could not be true.

**Briggs** disagreed with the proposal but if she were to consider it, she would want to say “from one or more allied taxa”. She thought that it sounded as if describing a species within a genus you might have to separate it from various species, where often the comparison would be made with the most similar one.

**Barrie** pointed out that this was going to have consequences for Art. 32.4 and trying to decide whether or not a species had an adequate description or diagnosis. He felt that it would make it a lot more difficult to tell whether a diagnosis was adequate and lead to a lot more problems deciding whether or not names were validly published based upon them. He was not in favour of the proposal and preferred the original wording that was in the *Code*.

**Cameron & Prud’homme van Reine’s proposal** was **rejected**.

[*Here the record reverts to the normal sequence of events.*]

### Recommendation 32B*bis* (new)

**Prop. A** (30: 74: 5: 0) and **Prop. B** (30: 71: 8: 0).

**McNeill** noted that the proposal had received a substantial “no” vote: 30 for, 74 against, and the Rapporteurs were also not enthused. It intended to restore a provision in the *St Louis Code*, and he called on the proposer to explain why the Rapporteurs were wrong in their criticism.

**Van Rijckevorsel** had thought that this was a fairly basic proposal, a basic Recommendation, even a classic Recommendation. He elaborated that it had been in the *Code* for a long time, even if it was not particularly suitably worded. He was always hearing from big database people who were having to deal with not validly published names and the problems they caused, so he felt that it was a useful addition, and wished to hear what the objection was.

**McNeill** thought that the comment was on the previous wording.

**Redhead** commented on both proposals because, when he just read Prop. A, he thought “Well this is rather trivial and we know this and why put it in the Recommendation?”. But, upon reading the Example, he said “This is very germane and really illustrates some of the horrible tangles we can get into when we delay publishing names”. So he strongly supported both Prop. A and Prop. B.

**McNeill** agreed that the two proposals belonged together and Prop. B would definitely fall if Prop. A was not accepted.

**Janarthanam** thought the proposal should be supported because he had seen people coming to conferences with a new name that got published in abstract books, which made it not a valid publication, but a nomen nudum.

**Rico** also supported the proposal. She agreed with the colleague from India because she had names that were provisionally listed in agronomical journals, then eventually people had been using them frequently. During assessment and cleaning up of some conservation assessments for the IUCN and the Red List, these names came along and they had not ever been published. She highlighted that because they were circulated and the mistake was transferred through years, there were even more complicated problems—like *Acacia* blah, blah, blah. She strongly supported the proposal.

**Knapp** indicated that the person in the red shirt should receive the microphone.

**Kirk** noted that it was not a shirt.

**Knapp** apologized and added that her wardrobe sense was also deteriorating.

**Kirk** supported the intent of the Recommendation, and felt that within the confines of the room it may be considered pertinent but in the rest of the world he did not think this would change anything. Nothing. He thought that people would continue to publish names in posters that get into abstract publications that may have a diagnosis and blah, blah, blah. To publish was to make public. People make things public on the web, from databases, herbarium collections, blah, blah, blah. Always not effectively published names. He maintained that it would not make any difference. So whilst he supported it, he felt it was superfluous.

**Demoulin** did not believe this had a place in the Nomenclature *Code* as it was not nomenclature but scientific, taxonomic behaviour. He understood that there were circumstances when it was recommendable that people hurry in publishing something, but there were also many more circumstances where people should wait a little bit before publishing. In his case, as a self-confessed perfectionist, he had delayed the publication of a number of new species (sometimes 40 years) until retirement in two months. He believed that it was useless to publish things when not quite certain how to define them in comparison to other species in a critical group. He maintained that new species should be published only when you had really mastered the taxonomy of the group. He opposed the proposal.

**Glen** took the previous speaker’s point and added that unfortunately there were rather too many folk of similar mind, who delayed publication and then got run over by a bus or something. He colourfully described how these names often “escaped into the wild” becoming feral names that became invasive in field guides and the like and it was impossible to know what was intended. He made a plea for the Recommendation.

**Gandhi** though it was a useful Recommendation, although he felt it may or may not change anything in real life. He added that, as many people knew, in major herbaria there were many specimens annotated by graduate students and postdocs as new taxa, and for various reasons they left the profession, botanical life, and such annotated specimens remained for years, causing confusion to later researchers.

**Herendeen** agreed that the positive reasoning for the proposal was evident to everyone present, it was best practice and did not belong in the *Code*.

**Redhead** brought up the point that, within the *Code*, the use of the word “name” was for validly published, and so this latter use of the word “name” would not apply when it was not valid.

**McNeill** responded that this was okay because it was talking about publishing it.

**Harley** considered it an unnecessary proposal to put in at this point, but if it was to be put in, he suggested adding not only circulating a name, but also annotating herbarium material before publication.

**Demoulin** felt that there were two very different issues: there was a Recommendation not to use unpublished names, and this was the longstanding Recommendation and he entirely approved of that, but there was a very new thing that he disapproved of—recommending to hurry in publishing.

**Knapp** summarized that the Section would be voting on Prop. A to add a new Rec. 32B*bis*, which was about publishing, discovering, recognizing new taxa, recommending to publish things as quickly as reasonably possible and its associated Example. She explained that if approved then the Example would go straight to the Editorial Committee, if rejected then the Example would be rejected as well, so the two proposals would be voted on together.

**Prop. A** and **B** were **rejected**.

**Veldkamp** had a question relating to Rec. 32B: he had been asked by several people for whom he was making Latin diagnoses, whether the word “ally” was allowed. He asked because in these days of molecular research an ally was not what it used to be 10 years ago. He wondered if this should be something like “several species”.

**Knapp** noted that if he wanted to change that, he would need to make a new proposal from the floor before Friday to change the *Code*.

[*The following discussion, pertaining to a new proposal by Veldkamp concerning Rec. 32B, took place during the Tenth Session on Friday afternoon.*]

**Veldkamp’s proposal**

**McNeill** read out Rec. 32B and the suggested amendment “The description or diagnosis of any new taxon should mention the points in which the taxon differs from its allies”, the proposal was to change “allies” to “similar taxa or allies”—to insert “similar taxa”.

[The **proposal** was **seconded** and **supported** by four others.]

**Demoulin** wondered if it would be a friendly amendment to replace this by “taxa with which it was likely to be confused in the opinion of the author”, so as not to make any judgment of what was similar or what was an ally. [This was not considered a **friendly amendment** and it was not proposed as a formal amendment.]

**Veldkamp’s proposal** was **rejected**.

[*Here the record reverts to the normal sequence of events.*]

[*The general discussion on electronic publication took place at the end of the day but has been moved to prior to Art. 29 in accordance with a logical order.*]

**Knapp** ended the day by noting that she was sure the Section could keep talking about electronic publication for quite a long time and did not want anyone to feel that she had cut the discussion off unnecessarily, but she pointed out that the room did need to be vacated or everyone would be locked in, and then it would be possible to talk about electronic publishing all night. She thanked all present for having a nice, open, calm discussion about something that would be dealt with first thing the next morning.

## Fifth session

Wednesday, 20th July, 2011, 9:00–12:30

**Knapp** welcomed everyone back and started the day with a couple of announcements, the first of which was that the Royal Botanical Gardens, Kew runs a nomenclatural training course, which she felt was incredibly useful and very, very good, and she pointed out that there were some leaflets at the registration desk in case anyone wanted to participate. She added that it was also possible to talk to Katherine Challis about it, which lead onto the second announcement: that it was Katherine’s birthday. [Applause.] She thought that it was probably a slightly mixed blessing to have your birthday during a Nomenclature Section but wished Katherine a happy birthday.

**Karen Wilson** added that it was also Gregor Mendel’s birthday and noted that Google had peas forming the letters of Google.

**Knapp** thought it was very nice that Gregor Mendel shared his birthday with Katherine.

[*All other business conducted during the Fifth Session was relevant to provisions of the Code dealt with earlier. The proceedings of the corresponding debates can be found under Art. 29, Rec. 29A and Art. 30 in the normal order under the Fourth Session on Tuesday afternoon.*]

## Sixth session

Wednesday, 20th July, 2011, 13:45–17:30

[*Some business conducted during the Fifth Session was relevant to provisions of the Code dealt with earlier or later. The proceedings of the corresponding debates can be found under Art. 30 (Zijlstra’s proposal), Rec. 30A, Art. 31 (including Bill Barker’s proposal) and Art. 32 in the normal order in the Fourth Session on Tuesday afternoon and also under Art. 38 in the Seventh Session on Thursday morning and discussion on sanctiotypes in the Tenth Session on Friday afternoon.*]

### Article 33

**Knapp** decided that instead of having to discuss and read all the new proposals from the Mycological Consortium (as she had chosen to call them) at the same time, discussion of Art. 33 would take place first in order to allow people time to read the proposals.

**Prop. A** (17: 87: 6: 0).

**McNeill** noted that Art. 33 Prop. A, was defeated by more than a 75% vote in the mail vote and intended to move onto the second item, Prop. B.

**Reveal** thanked Madam President for allowing him to speak.

**Knapp** notified him that the term was “Queen of the World”. [Laughter.]

**Reveal** corrected himself to say Queen of the World, with apologies. [Laughter.]

**Knapp** apologized [but in a very royal way].

**Reveal** noted that the reason for Prop. A was to make clear that when an epithet was not associated with a generic name, as outlined in one of the examples that…

**Knapp** pointed out that four supporters were needed to speak to it. [The **motion** was **seconded** and **supported** by three others.]

**Reveal** continued that in the *Code*, there was an example of Linnaeus in which he failed to associate an epithet with a generic name. Reveal and his colleagues had found a number of instances where that was also true. He asked the question: if the two articles were published simultaneously, what page was the name validated on? If the two articles appeared at different times, as was often the case when there was a name in a journal and an index at the end of the journal that may have been published months or even years later, the question was: who was the author?

He felt that everyone was well aware that, in journals, the authors of names tended not to write the index. Therefore he argued that the name would not be valid where it was not associated with a generic name but would be validated in the index, meaning that one would have a change of authorship—often some obscure person, although he conceded that probably many editors did not regard themselves as particularly obscure. The question here was that in those cases, where the index and the work were published at the same time, there really was no difficulty, particularly when it was a book. When it was in the same journal and it was published at the same time, there was a question: who validated the combination in the index? Was it the editor or not? This proposal addressed that question by mandating that if a name was published in an index, that it is not necessary to go to the labour of finding out who was the editor who might have indexed the work but, rather, simply ascribe it to the name of the authors who attempted to propose it but failed under our modern rules.

**McNeill** noted that the Rapporteurs’ comments were, essentially, that they thought the proposal was unnecessary because it would be provided for quite clearly under both Art. 45.1 and Art. 46. He elaborated that Art. 45.1 made it clear that it was the last stage in the valid publication of a name that was the date of valid publication and if the requirements were met over successive dates then it was the latest one, so if the index came later it would be in the index. What was more, he added that Art. 46 addressed the question of the authorship of the name: if it was in the index without authorship and a page number, it clearly related to the person who wrote the entry for that taxon on the page number. He argued that there was no suggestion of raking through finding who might conceivably have been the editor or the person producing the index. If, on the other hand, an index was ascribed to somebody else, he explained that again Art. 46 prescribed what the authorship should be, so the perception of the Rapporteurs was that it was really unnecessary.

**Gandhi** explained that the reason he proposed this, along with Reveal, was that he was reviewing a manuscript for the *Flora of North America* and had come across this situation. He reported that the manuscript author had vigorously opposed accepting the validity of the combination listed in the index. According to him, the article author was not the author of the index. Gandhi had thought it would be quite helpful if such an Example was given.

**McNeill** thought that whoever took that view was misreading the *Code*.

**Gandhi** reiterated that the argument presented to him was that the author of the article maintained that he was not responsible for the index, and did not accept it. It seemed that an Example would be very useful to offer support in explaining it to such an author.

**Challis** had understood that the proposals were concerned about the authorship of the name and suggested that it might be simpler to include an Example somewhere in Art. 46, if that were thought desirable.

**Knapp** deemed that the proposal should be referred directly to the Editorial Committee unless there were any objections.

**Prop. A** was referred to the **Editorial Committee**. [Note: it was recorded in Taxon 60: 1513. 2011 as rejected by the mail vote.]

**Prop. B** (59: 23: 10: 7).

[*The following debate, pertaining to Art. 33 Prop. B, took place during the Seventh Session on Thursday morning.*]

**Knapp** was just checking to see if there were any other *Microsporidia* emerging from their parasitic holes.

**McNeill** said yes there was, not on *Microsporidia*, but a proposal that was a corollary to the acceptance of Art. 37*bis* had not been dealt with: Art. 33 Prop. B.

**Greuter** had a question, as he was having difficulty understanding what the proposal was covering. He read a “new combination or new name” and felt that if by “new name” a new name as normally understood was meant, then “new combination” was included and the provision should be under Art. 32. However, if by “new name” a replacement name was meant, he did not really understand why names of new taxa should be excluded.

**Hawksworth** thought it was a necessary corollary of what had already been accepted, and it had attempted to cover replacement names.

**McNeill** checked that the new combination or replacement name was what was intended.

**Hawksworth** confirmed that was the intention.

**Prop. B** was **accepted**.

[*Here the record reverts to the normal sequence of events.*]

**Prop. C** (95: 6: 8: 0).

**McNeill** introduced a set of proposals by Turland, starting with Art. 33 Prop. C, which was linked to Art. 32 Prop. E.

[*The latter was accepted, and the discussion can be found under Art. 32 in the Fourth Session on Tuesday afternoon.*]

**McNeill** explained that Art. 32 Prop. E was closely linked to the rest of these proposals, which included Art. 33 Prop. C, and they aimed to clarify the requirements for valid publication of names of taxa that were already named. He added that at the moment, Art. 32 dealt substantially with names of new taxa but the *Code* had not clearly distinguished between the rules for valid publication of the name of a new taxon and for valid publication of the name of an existing taxon, one already named.

**Turland** explained that the rationale behind the set of proposals was to try and simplify the wording mostly in Art. 33, some of which had already been dealt with right at the beginning of the Nomenclature Section, when the term definitions were added to Art. 6—replacement name and new status and new combination and name of a new taxon, defining what those meant—thus enabling those terms to be used in a more concise way later on in the *Code*, in particular in Art. 33.

He continued that there was a fundamental dichotomy in the *Code* whereby a name may be validly published, either as the name of a new taxon or what might informally be called a renaming. He elaborated that he meant a new combination, a name at a new rank, a new status, or a replacement name or nomen novum. He referred to the Section’s earlier decision to use “replacement name” as the preferred term and added that it was now implicit from Art. 32.1 that these renamings could not entirely fall under Art. 32.1. The only way that the renaming could be validly published was if it fell under Art. 33, which he noted was, of course, the case.

That fact that it was implicit in the *Code* but not explicitly spelled out was his rationale for the proposal, adding the relevant words at the end of clause (d) of Art. 32.1. In the actual proposal, he had given the following example. If there was a new combination, all that was needed was to cite the basionym, the full and direct reference to the place of valid publication of the basionym. Supposing that basionym itself was validly published by reference to an earlier description or diagnosis—so the basionym did not include a description or diagnosis in its protologue but it included a reference to an earlier one—he said that if this was published today as a new combination, it could be done validly even on such a basionym.

So that combination was not validly published through Art. 32.1 because there was no direct reference back to the validating description or diagnosis because the basionym did not include one. There was an indirect reference via the basionym so the new combination must be validly published some other way and that came through Art. 33.4. What the proposal was doing was making explicit what was implicit in the *Code*.

**Wiersema’s proposal**

**Wiersema** had discussed some of the considerations that he had about this group of proposals with Turland and with Greuter. He noted that it would involve some rearrangement of Art. 41, Art. 33 and to a lesser extent Art. 32. Regarding Art. 32 he understood that there was no intent to change any of the application of the current rules of the *Code* or the interpretation. It was simply a matter of clarifying, but in the process it would, as Turland had noted, split out the treatment of new names on the one hand, which would be dealt with in Art. 32, and new combinations, replacement names, status novus, in Art. 33.

He felt that there was also another issue about some of the content of Art. 32, in that it did not all fit together nicely, so he suggested that the Editorial Committee would benefit from having some latitude in order to restructure parts of Art. 32. He noted that there were some proposals to move the content of Art. 41 into some of Art. 32 and 33. He supported all of the suggestions but wished to propose as an addition, if the changes were accepted, that the Editorial Committee have the latitude to do some restructuring and tighten up anything that may not have been accounted for in some of the proposals, with the assurance that there would be no change in the application of the rules so that the interpretation would stay the same.

**McNeill** noted that was a stated proposal. He summarized that Wiersema was drawing attention to the fact that, beyond the normal authority that the Editorial Committee had to adjust things that were discussed here, because there were a number of proposals that had the same intent but were somewhat different in actual wording, particularly concerning how to handle Art. 42, that in this case there would definitely be much more need by the Editorial Committee to make adjustments of positions of particular clauses that were not necessarily discussed here. He added that the Editorial Committee had always had the power to do that, but it was good that the Section should know that this was an option.

**Greuter** strongly supported what Wiersema and the Rapporteur had just said. From his past experience, such a major and most beneficial reshuffling of the form and arrangement in the section of types—he thought it was after either Berlin or Tokyo—which was an absolute mess before and no one really knew where to look—had come out in quite a logical structure afterwards. The result was that there was the first portion dealing with types in general; then, a second one, dealing with types of names of species and taxa of lower ranks; then, one of selection of those types; and the fourth one about generic types—types of supraspecific names, to be correct.

He felt that something similar was obviously needed in Chapter II of the *Code* from Art. 32 to 45, because it was one of the most messy sections, with which generations of botanists, taxonomists and nomenclaturalists had been tampering without any major attempt to put order into it. He asked the rhetorical questions: Why wasn’t that made? Why didn’t they do it? He noted that he was collaborating in former Editorial Committees long enough, and suggested that people may say that he could have done it, but the thing was so messy that it was impossible to deal with editorially. He felt that the main stumbling block was Art. 41, in which all categories of other Articles were entangled, explicitly or mostly implicitly.

He did not know how familiar the Section participants were with Art. 41 and explained that it was the one forming rank groups within which the descriptions could apply. He suggested that the one with which people may be familiar was for a species—descriptions of species or infraspecific taxa, but not of higher taxa, may be used for validation. This had been assumed to apply also for combinations. That was to say it was not allowed to [re]combine the name of a series as a species name. These were the same groups but this was never explicit. He felt that with one of the Turland proposals, the situation would be clarified and become much clearer. If that was passed, he saw a good chance for the Editorial Committee to come up with a coherently structured new chapter. He acknowledged that it would require quite some reshuffling and quite some care not to inadvertently introduce any change, but he was sure that the Editorial Committee would be able to do that. He would heavily support that the mandate to do it be given explicitly so that they did not find themselves too shy and bound by the present text and order and numbering.

**Knapp** queried the use of the word ‘shy’.

**Greuter** confirmed that he felt that the Editorial Committee was usually very shy. [Laughter.]

**McNeill** interpreted that as a seconder to Wiersema’s proposal, which could be treated as though it were an amendment but it was actually a separate proposal, as to how to deal with the set of proposals being covered, which might better be considered first.

**Knapp** proposed that the Section consider it as a proposal. [The **proposal** was **seconded** and **supported** by three others.]

**Turland** recommended that it be made clear that the discussion would return to Art. 32 Prop. E once this separate proposal was settled.

**McNeill** thought it should be discussed, but that it might be better to vote on it once finished with the whole sequence. He noted that was up to the current Section to decide.

**Knapp** clarified that the suggestion was to discuss whether or not, we, as a Section, would like to give the Editorial Committee the latitude—whilst being quite careful—to really substantially modify the structure of Chapter II of the *Code* to make it more coherent.

**Turland** added that they were not being given the power to change the intent of any of the Articles unless expressly agreed by this Section in the proposals that were coming up.

**Van Rijckevorsel** pointed out that Art. 32 basically did two things at the moment. Firstly, it set out the conditions for valid publication and, secondly, it went into detail on the matter of a description and diagnosis. To him, it would make sense to take out everything on the description [and] diagnosis and, perhaps, move it into Art. 36, which was also dealing with descriptions and diagnoses. He felt that was a basic point, which would clear up much of Art. 32.

**Knapp** moved to a vote as there were not objections to doing so. She reiterated that the proposal was to give the Editorial Committee the latitude to reorder Chapter II of the *Code*—without changing any of the intent of any of the Articles unless expressly amended by the Section.

**Wiersema’s proposal** was **accepted**.

**Knapp** thanked the Section and added that she was sure the Editorial Committee would greatly appreciate that latitude being given.

[*A short discussion of Art. 32 Prop. E, to amend clause (d) of Art. 32.1, occurred here and has been moved to the normal order in the Fourth Session on Tuesday afternoon.*]

**McNeill** returned the discussion to where the action started in Art. 33 Prop. C, which was adding another clause to Art. 33.1 or after Art. 33.1.

**Turland** explained that the idea of the proposal was to take the tenets of Art. 41 as they applied to Art. 33, and place them in Art. 33. When a name was validly published by reference to a previously published description or diagnosis, there were limits on the rank of the taxon to which that previously published description or diagnosis applied. He added that this applied when you had the name of a new taxon, which fell under Art. 32. He pointed out that there was a parallel proposal by Rijckevorsel, which would come up later under Art. 41 and was essentially taking the wording of this rank restriction from Art. 41 and placing it in Art. 32, and he fully supported that proposal.

The current proposal was a parallel one, so that the same principle applied to renamings: to new combinations, replacement names and names at new rank, which was normal practice in publishing and really nothing new. It was part of the restructuring. He gave the following example: if a new combination in the rank of species was published, the basionym would have to be the name of a species or an infraspecific taxon. It was not possible to publish a new combination at specific rank based on, for example, a generic name. Nobody did that and it was because of Art. 41. Previously it was necessary to jump to Art. 41 to see that this rule was apparent. The idea here was to actually incorporate it where it was more obvious, in Art. 32 and Art. 33.

**Prop. C** was **accepted**.

**Prop. D** (60: 6: 40: 0).

**McNeill** introduced the proposal as dealing with taking advantage of the definition of status novus—new status—and the definitions at the beginning.

**Turland** felt he should explain how the wording “new generic name with a basionym” came to be in the *Code*. It was mostly added editorially during the editing and production of the *Vienna Code* and gave the impression that a status novus could not exist above the rank of genus, although prior to the *Vienna Code*, there was nothing in the *Code* that ruled that. It was really tightening up on what was done editorially in the *Vienna Code* and replacing “new generic name with a basionym” with “status novus”, which had been defined in Art. 6 on Monday.

He noted that there was also a side issue that when the Section voted on the status definitions in Art. 6—that “status novus” should be “new status”. The Rapporteur had pointed out to him that, really it should be “name with a new status” because a new status was not actually a name, it was a rank, which was a different thing to a name. He suggested that perhaps it would be possible to editorially use a more precise term—“name with a new status” or, perhaps, “name with a new rank”.

**McNeill** agreed that it could be treated as editorial as the principle had already been enunciated and approved.

**Knapp** reiterated that the Section was voting on Prop. D to replace the phrase “new generic name with a basionym” with something like “name with a new status” or “name at a new rank” editorially.

**Prop. D** was **accepted**.

**Prop. E** (64: 0: 39: 0) was referred to the **Editorial Committee**.

**Prop. F** (6: 5: 93: 0) was ruled referred to the **Editorial Committee**.

**Prop. G** (86: 5: 17: 0).

**Turland** read out what was written in the Rapporteurs’ comments. “Prop. G is an attempted clarification of Art. 33.3, partly through use of the definition of status novus, in Art. 6 Prop. A, and partly to better reflect the situations to which the Article applies”. He added that it was an editorial rewording of Art. 33.3 that did not really change the meaning but, hopefully, made it clearer.

**Prop. G** was **accepted**.

**Prop. H** (69: 6: 31: 0).

**McNeill** noted that the proposal was also an editorial clarification made possible by the decisions on Art. 6.

**Sennikov** proposed an amendment to the wording to insert “volume, issue,” before “page or fig reference” and insert “(if applicable and required for unequivocal identification of the protologue)” after “reference”. The reason he gave for the amendment was that there could be many volumes in the same year, especially in the case of books, and many periodicals about 100 years ago were published with dozens of issues per volume, each with separate pagination, so it was literally too difficult to find the protologue if there was no volume reference or issue reference given. He added that “If applicable and required” was for the cases when these details were not needed or not applicable and he felt that even “page and fig reference” should also fall under “if applicable” because not all publications were paginated. [This was **not** accepted as a **friendly amendment**.]

**McNeill** drew the attention of the Section to the fact that this would apply retroactively from 1 January 1953 and invited others to comment on it, such as Challis and Gandhi, if they wished.

**Knapp** recognized Paul Kirk.

**McNeill** added “or Paul Kirk”.

**Knapp** justified that he had his hand up first.

**Kirk** thought that McNeill knew what was coming… [He did.] Kirk supported the amendment extremely strongly [taken as the amendment being **seconded**] for the simple case that in many serial publications published in individual numbered parts there was a page 1, so it was not possible to give the concise reference to a basionym by omitting the issue number. The volume and page was not accurate because there was issue 1, issue 2, issue 3 in the same year.

**McNeill** was not questioning the necessity and importance of doing so but was asking the question as to whether the requirement from 1 January 1953 would result in a number of names that had been assumed to be validly published no longer being validly published.

**Kirk** confirmed that it would.

**McNeill** asked for confirmation as to whether Kirk was supporting the proposal or against it and concluded that he wanted those names not to be validly published.

**Dorr** felt that the amendment would be severely destabilizing and was very much opposed to it, as he thought it would undo an enormous amount of work that was done with people dealing with combinations published after 1 January 1953.

**Wiersema** agreed 100% with what Dorr had just stated. The reason for this was in Art. 33.5, where it said “For names published on or after 1 January… errors in the citation of the basionym or replaced synonym, including incorrect author citations, but not omissions, do not preclude valid publication”. He argued that this would be taken as an omission if it was left out, and so it would render names that lack this not validly published.

**Reveal** noted that for those who actually published books, there were no volume numbers and issue numbers; just page numbers, and he thought that it seemed to exclude the publication of anything in a flora.

**McNeill** clarified that was not the case because it specified *if necessary* for the unequivocal identification of the protologue. This was addressing the situation to which Kirk was referring and which was extremely common in older works, and the need for doing this was very important. It seemed to him that the proposer might want to think about the issue, withdraw this proposal and propose it afresh at an appropriate time in the discussion, to date from, say, 1 January 2013.

**Sennikov** added that he had considered Art. 33.5 because these issues—volume and issue numbers—were indeed omissions, so the cases already fell under Art. 33.5 and the wording that he wanted to add to Art. 33.4 was just to make it explicit. Otherwise he suggested that taking the wording of Art. 33.5 literally and deciding that, indeed, in the case when the volume number was missing and the citation did not allow finding the due page amongst, say, a dozen volumes published in that year, that they were omissions. He argued that since the omissions had happened, the reference was incomplete and so the name was already invalidly published and nothing would be lost in adding his amendment, but some precision would be gained.

**Demoulin** gave an example when this would be destabilizing: there were a number of journals that added one volume with continuous pagination a year, such as *Bulletin de la Société Mycologique de France*. It was quite possible for somebody to refer by the year and not by the volume or issue. In a case like that, somebody who made a new combination could cite a basionym in *Bull. Soc. Mycol. France* 1956, page so and so. Like some others, he considered it a very dangerous amendment.

**Herendeen** called the question.

[There was a sufficient majority in favour of voting on the **amendment** and there was a majority against so the **amendment** was **rejected**.]

**Knapp** returned the discussion to Prop. H to reword Art. 33.4.

**Prop. H** was **accepted**.

**Prop. I** (14: 48: 43: 0) was **withdrawn**.

**Prop. J** (8: 104: 2: 0), **K** (8: 97: 3: 0) and **L** (9: 95: 5: 0) were all ruled **rejected**.

**Prop. M** (64: 7: 37: 0) was referred to the **Editorial Committee**.

**Prop. N** (14: 13: 81: 0) was ruled referred to the **Editorial Committee**.

**Prop. O** (95: 9: 4: 0).

**McNeill** introduced Prop. O, to delete an Article that was introduced at Vienna, which was rather disruptive in many people’s view. It was a situation that was quite contrary to the normal principle of the *Code*, in which valid publication is determined on the basis of what actually appears in the protologue rather than what the author says they are trying to do. [The following are not McNeill’s words, but presumably what he intended to say.] It had created the situation where a name that was claimed to be a new combination, but was not validly published as such, yet it met all the requirements for valid publication as the name of a new taxon, could not be validly published. He suggested that it would seem much wiser just to delete the Article.

**Prop. O** was **accepted**.

**Prop. P** (16: 25: 64: 0) was ruled referred to the **Editorial Committee**.

### Recommendation 33A

**Prop. A** (83: 2: 23: 0).

**McNeill** noted that the proposal was substantially editorial in the light of what had already been approved.

**Turland** read the Rapporteurs’ comments: “Prop. A corrects what appears to have been an oversight in the *Vienna Code*, when the phrase ‘new generic name with a basionym’ was introduced widely in the *Code*, and which earlier proposals in this set … would replace by the now defined term status novus”, adding that it had been decided to call status novus “name with a new rank”.

**Prop. A** was **accepted**.

**Prop. B** (12: 82: 14: 1) was ruled **rejected**.

### Article 34

**Prop. A** (4: 95: 9: 0) was ruled **rejected**.

### Article 35

**Prop. A** (49: 50: 3: 2).

**McNeill** introduced the proposal as relating to something that was agreed upon in Berlin or in Yokohama, establishing a date at which the ending, the termination of the name of a subdivision of a genus would determine the rank being given (he corrected himself to a name at a rank above genus). The proposal was to change the date at which this was implemented.

**Reveal** had discovered after the fact that there were a large number of cases in Engler and Prantl’s works—including his journals, of which there were several—in which names at the rank of subtribe, even though they were given the termination [*inae*], were not assigned to a rank. When they were, which was not common, they were assigned the rank of section. Nonetheless, because the determination was consistently used according to the independently published recommendations for publishing in Engler and Prantl’s works, we cannot maintain that the use of *inae* was covered by his instructions in the publication per se, because the work in which this information was given was published independently of the works. In attempting to go through this 1890–1908 literature, which was interesting, Reveal had tried to find examples where this was not a case of an Englerian student or associate or colleague or project, and he could not.

He suggested that some of those in the audience may have had some examples, and that was great, but it seemed to him that all of these subtribe names had always been used as subtribes, instead of considering them to be validly published as rankless names. His suggestion was to continue to use them as subtribes; just move the date and be done with it.

**Prop. A** was **accepted**.

**Prop. B** (57: 5: 44: 0) was **accepted**.

### Article 36

**Prop. A** (20: 98: 0: 0) and **B** (15: 87: 8: 4) were ruled **rejected**.

**Prop. C** (54: 38: 9: 5).

**Demoulin** introduced a set of proposals he had devised at the time of the Mycological Congress a year ago. Some of the proposals represented his personal view after the discussions at the Congress, and the following ones, in Art. 36, were a special kind because they represented a large majority will of the people who discussed nomenclature at that Congress., They had asked either Gams or himself to present the proposal in the name of the people taking part in those discussions at the International Mycological Congress.

He suggested that the Section may be aware that he would have preferred to keep just Latin for everything including fungi, but he admitted that there were circumstances where it was better to have a compromise. Given the hostility of a few persons towards the use of Latin in the mycological community, he suggested that this could be the way to live in peace in the future, so he had made the proposals as instructed to do. He thought they were the things that the mycologists wanted. He referred to the comments by the Committee for Fossil Plants because the idea was that, since the fossils had it, it would be possible to take the same situation as they have and use the comments of the Fossil Committee, which said that they have had a good experience with allowing both Latin and English.

**McNeill** suggested that the Section should concentrate on Prop. E because that was the one that had the substance in it and C and D were really corollaries. Moreover, he added that the Nomenclature Committee for Fungi had voted 11 to 3 in favour of the proposal and that the International Mycological Congress in Edinburgh last summer, where it arose, had supported it. He reiterated that the Committee for Fossil Plants had expressed their positive experience of a similar situation in a report.

**Demoulin** added that it came up in a discussion about the language to be used, but noted that the Mycological Congress was very much opposed to Prop. A to use “any language”. The opposition was very, very strong despite the fact that some people were opposed very much to Latin. They were also opposed very much to “any language”.

[Prop. E was voted on first and then C and D.]

**Prop. C** was **accepted**.

**Prop. D** (52: 39: 9: 5) was **accepted**.

**Prop. E** (65: 34: 4: 5).

**Buck** referred to Demoulin saying that they were trying to follow the model of fossil plants and argued that fossil plants required illustration, just like the algae, so he thought they wanted to eliminate a requirement but not add the requirement that was there for fossil plants and algae.

**McNeill** explained that that was in a quite separate Article so there was no suggestion that this proposal involved that.

**Gams** strongly supported the proposal, although it was a very delicate matter. He wanted to make a **friendly amendment**. If the diagnosis was to be written or published in English, then it should be preceded by the word “diagnosis”, otherwise it may be confused with any lengthy English description.

**McNeill** pointed out that there was no requirement for a diagnosis in the *Code*. There was only a requirement for a description or diagnosis. He added that that did not preclude him making the suggestion, but he thought he should draw attention to that. [Nothing further was done with the amendment.]

**Alvarado** saw a point in still using Latin for formal descriptions. He thought one of the most important things was to make sure that the author wanted to describe a new taxon because sometimes it had happened, especially with animals, that people did not want to publish a new name but they wrote down the holotype was in this place and then a brief description, not in Latin, because they do not have that requirement. Then it became official but, in fact, they did not want to do that. He thought that the Latin requirement was, in part, useful because in that way the author was stating “I want to propose a new taxon”.

**Herendeen** spoke for the Committee for Fossil Plants, and wanted to provide a bit of background and support for the proposal. Previously, fossil plants allowed any language to be used for publishing new names. It was decided 12 years ago that that was too difficult to deal with, and so the requirement was tightened to either English or Latin, and it had worked very well for the fossil community over the last 12 years. As to the last comment, he was not aware of any publication where someone got confused as to whether a new name was actually being published recently while in English or prior to that. He did not see that as a real problem in the field. He reported that the Committee for Fossil Plants was strongly in support of what the mycologists were trying to do.

**Hawksworth** had discussed this quite extensively with Chinese, Russian and Latin American delegates at Edinburgh. They were very strongly of this opinion as well, so he wanted to stress that it was very much an international meeting that expressed this opinion.

**May** clarified that both in the Committee for Fungi and at the Nomenclature Section at Edinburgh, there was a greater than 60% support for this proposal, just to be very clear, both in the Committee for Fungi and at the IMC.

**Peter Wilson** wanted to propose an amendment, a radical one really, because he thought this should be extended to all organisms under the *Code*. He felt that the time had come, “with me as an ageing botanist who had facility in Latin…”

**Knapp** exhibited surprise and exclaimed “Surely not!”

**Peter Wilson** “…and on whose shoulders the responsibility for other people’s diagnoses and descriptions falls…” [Laughter.] “…and someone whose proposed date of retirement is approximately the next Congress, I think the time has come for us to actually consider this seriously for all organisms.”

**McNeill** thought that was the substance of a subsequent proposal that was intended, and suggested he may wish to liaise with Smith from South Africa, and that it may be better to include it—not as an amendment, but in that separate proposal for other groups immediately after this had been dealt with.

**Peter Wilson** accepted the suggestion and added that he was strongly in favour of it for the mycologists.

**Redhead** reiterated that there was strong support within the Committee for Fungi and at the International Mycological Congress nomenclatural sessions for this and he certainly supported it. A colleague at his institute had asked that he put forward an amendment to it to be entertained that it should be changed to Latin, English, German, French or, he thought, Italian; the original languages that were with the *Code*. He had promised that he would put that forward as an amendment. [The **amendment** was **seconded**.]

**Knapp** thought it was a laudable thing that he was being true to his word.

**Redhead** thought that the original languages were German, French, English and Spanish.

**McNeill** explained that there was no Spanish originally.

**Norvell** did not support the amendment as she felt it was creeping dangerously close to any language, and she thought that Latin or English was sufficient.

**Nelson** questioned how you could include French and exclude Italian, Spanish and Portuguese.

**McNeill** added Chinese. [Laughter.]

**Peter Wilson** opposed the amendment. He believed that English now occupied a place in scientific communication that Latin occupied in Linnaeus’s day. He referred to the Rapporteurs’ comments that the people who wanted their work to be taken notice of would publish in English and he thought that was the way it should stay.

**Cafferty** called the question.

[There was a sufficient majority in favour of voting on the **amendment** and there was a majority against so the **amendment** was **rejected**.]

**Barrie** called the question. [Laughter. There was a sufficient majority in favour of voting.]

**Prop. E** was **accepted**.

**Smith’s proposal**

**McNeill** noted that this was the point where he had been given notice that people would like to produce an alternative proposal to the defeated Prop. A and B. He thought it was better, where something was essentially a new proposal, that the ones that were already published should have the opportunity to be presented and considered first, which was why it had been done that way. Now was the time for either Smith or Peter Wilson to make their proposal. He suggested that it be presented as a proposal for the intent of what it was desired that the *Code* would provide for and the nitty-gritty of 1 January 2013 and so forth could be left to be an editorial matter.

**Smith** felt that the first proposal as phrased would have introduced the “Babylon Effect”, as it was commonly known. He found it understandable, listening to the comments, that it was heavily defeated in the mail vote. In support of what Peter Wilson had said a few minutes ago, and what the Rapporteur had just stated, he put forward that this principle that had just been voted on be extended to include all organisms covered by the *Code*.

**Knapp** understood that the principle would be that in order to be validly published, a name of a new taxon treated by the *Code* published on or after 1 January 2013 must be accompanied by a Latin or English description or a diagnosis. [The **proposal** was **seconded** and **supported** by three others with many raised hands and laughter.]

**Alvarado** noted that in the year 1953, when it was agreed that there were Latin diagnoses for plants and all such organisms, Latin was still a living language because the Catholic Mass was still in Latin. He argued that nowadays, Latin had disappeared pretty much from everywhere so the only place where Latin was used as a language and not just as a dead language that was studied by scholars, was botany. He felt that if the Section said “Let’s remove Latin from botany”, Latin would effectively die as a useful language, so it would be a great loss for humanity. It was the language of the Roman Empire! [Laughter.]

**Knapp** recognized Katherine Challis and noted that she was allowed to comment many times because it was her birthday.

**Challis** pointed out that the requirement for Latin was not being removed—it would be Latin or English, so any traditionalists could still write their descriptions in Latin.

**Sebsebe Demissew** thanked Smith for suggesting the proposal as he and his colleagues had really been struggling to find somebody to write a Latin diagnosis for them over the years, so it was a relief to at least be able to communicate in English.

**Levin** did not think that any more discussion was going to change anybody’s mind in the room so, therefore, he called the question. [There was a sufficient majority in favour of voting.]

**Smith’s proposal** was **accepted**. [Applause.]

**Sennikov’s proposal**

**Sennikov** hoped that the audience would excuse him for being so impatient, but he wished to submit a new proposal from the floor to make a small emendation to what had just happened to replace the date 1 January 2013 with 1 January 2012. He argued that for the same reason as had already been discussed, that the account of the Congress decisions would already be available in *Taxon* during 2011. In principle he hoped that the community was as impatient as he was. [Laughter. The **proposal** was **seconded** and **supported** by three others.]

**Sennikov’s proposal** was **accepted**. [Applause.]

### Recommendation 36A

**Prop. A** (14: 95: 3: 1) and **B** (18: 84: 6: 0) were ruled **rejected**.

**Prop. C** (50: 42: 13: 1) was referred to the **Editorial Committee**.

### Article 37

**Prop. A** (15: 91: 2: 0), **B** (10: 104: 1: 0) and **C** (5: 105: 3: 0) were ruled **rejected**.

**Prop. D** (6: 7: 93: 0), **E** (64: 0: 42: 0) and **F** (79: 1: 31: 0) were ruled referred to the **Editorial Committee**.

### Recommendation 37A

**Prop. A** (83: 30: 3: 0).

**McNeill** introduced Rec. 37A and noted that there were four proposals, relating to citation of herbarium specimens. Article 37A Prop. A was dealing with recommending the citation of herbarium serial numbers of type specimens. It had considerable support in the mail vote: 83 to 30. He highlighted that it was a Recommendation only, so was not mandatory. If herbaria did not have barcoded or otherwise indicated specimens it was not an inhibition, but it was a Recommendation to make identification of which specimen was the type of a name more readily accomplished.

**Dorr** was troubled by this because he felt it was very easy to transpose numbers. If a string of seven, eight, nine numbers did not agree with what was on the specimen, then it had the potential to cast doubt onto whether or not the action had been taken. He thought it was a foolish proposal.

**Rico** thought nowadays, everyone who had barcoding in the herbarium, had a barcode reader, so it was possible the numbers would be the responsibility of the editor more than the people writing the thing. She thought that was just an excuse.

**Magill** noted that most barcodes today were gummed to labels that come off, and the policy that his institute was using was if a barcode was lost a new one was put on; there was no attempt made to find the old number. The numbers published for these names would, therefore, be changed over time. He added that they did have accession numbers that could be used, but that was different.

**McNeill** noted that actually, the Recommendation was that the herbarium serial number be used. It was not actually saying that there need be a barcode.

**Acevedo** reminded the Section that not all herbaria had those numbers assigned to their collections, so that would preclude people from smaller herbaria.

**McNeill** pointed out that it was purely a Recommendation with no mandatory element. Where there was no number, it obviously could not be entered. No problem.

**Acevedo** accepted that.

**Dressler** did not see any reason why it should be restricted only to types.

**Gandhi** believed it was a useful suggestion to cite an accession number following the type citation. He shared one of his recent interesting experiences. A name was published with the citation of the collection number, date of collection and the herbarium housing that collection, a Latin diagnosis was provided and, for all practical purposes, the name appeared to be validly published, because it met the requirements of every Article for validation. However, later on, it was discovered that the cited number had three different specimens housed in the same herbarium.

He reported that they had recorded the name as validly published, but later on it became apparent that there were three specimens without the holotype being annotated or indicated. The person who studied it in detail later on had declared that the previously published name was invalidly published, so had proposed a new species name.

Gandhi had consulted his IPNI colleagues, McNeill and Greuter and they said the relevant name was validly published because they met the requirement of whatever was needed—like the citation of the type, the herbarium housing the type and the date of collection; things like that. His point was, if the accession number was available, to please use it because it was useful for stability.

**Greuter** felt that the intent of the Recommendation was good, but he had some difficulty with some of its wording. He suggested that the amendment could be “The number identifying the herbarium sheet, if present,” instead of “The herbarium serial number”. The reason he gave was that there were many complex situations in many herbaria—including those of the Third World—for instance the presence of more or less herbarium serial numbers that identify the sheets in herbaria that have been incorporated into larger herbaria, so that it was no longer possible to speak of a herbarium serial number and it was impossible for the outside user to know what the serial number was exactly. He argued that these were numbers identifying the sheets and if they were not pencilled they could serve very well to identify the sheet. He explained that pencilled numbers were often added on loans, a practice he thought should be discouraged because such loan numbers were then often quoted in type citations as the sheet number. [The **amendment** was **seconded**.]

**May** noted that for fungi and cryptogams there were no sheets, but packets. He disagreed with the amendment and preferred the term “accession number” to cover the circumstances in most.

**McNeill** suggested that there might be a friendly amendment from “the herbarium sheet” to “the herbarium specimen”. [This was **accepted** as a **friendly amendment**.]

**Kirk** stated that fungi were not plants and their reference collections were not herbaria. [Groans.]

**Knapp** berated the audience “No moaning. No moaning allowed.”

**Hawksworth** pointed out that Kirk was quite correct and he supported him, and suggested use of the term “reference collection”, he added that the Section should also remember that the *Code* now covered permanently preserved cultures in microbial collections where this was quite critical, so the word “herbarium” should certainly be removed.

**McNeill** agreed it was totally unnecessary and he thought it could just be “the specimen”.

**Knapp** noted that was an editorial thing.

**Saarela** thought that “the number identifying” was too ambiguous as it would include a barcode. He agreed with the earlier commenter who said that barcodes should not be used for this because they were not permanent.

**Paton** thought part of the problem was there is not necessarily one number: a collector number, a barcode number, an accession number. He thought it was vague, but was also against clarifying it as he did not really like the Recommendation.

**Thiele** thought the issue could be solved by saying “a number permanently identifying the specimen”. [This was **accepted** as a **friendly amendment**.]

**Stuessy** felt that if it just said “a number”, to him that meant the number of the collector and he did not think that was the intention, rather an institutional number, a collection number. He thought that needed to be qualified.

**Janarthanam** thought it could be something like “unique number” after “herbarium”, identifying the specimen. Serial numbers and accession numbers were unique for each sheet.

**Knapp** exercised the Chair’s prerogative and decided to stop the wordsmithing of the Recommendation and decide about the principle.

**Unknown speaker** called the question.

[There was a sufficient majority in favour of voting on the **amendment** and there was a majority for, so the **amendment** was **accepted**.]

**Walsh** was sure the intention was implicit but he wondered if it needed to be added, in the case that a herbarium might mistakenly have two identical permanent numbers or the number was incorrectly cited, that priority should always be given to the information around the specimen rather than the institutional number.

**Knapp** asked for clarification as to whether he was proposing an amendment to the proposal.

**Walsh** guessed he was, and suggested adding the words “In the case of a duplicated or incorrectly cited…”

**McNeill** thought it sounded that what he was suggesting belonged in an Article rather than a Recommendation.

**Walsh** accepted McNeill’s opinion and retracted his comment. [The **amendment** was **withdrawn**.]

**Prop. A** was **accepted** as amended.

**Prop. B** (18: 97: 1: 0) was ruled **rejected**.

**Prop. C** (90: 20: 5: 0).

**McNeill** introduced Rec. 37A Prop. C and D, which were independent distinct proposals. Both encouraged what the Rapporteurs considered important information in the protologue: that the type and its place of preservation be presented in the Latin alphabet rather than in any language—such as Chinese. These were proposed by two Japanese authors and they were suggesting that designation of the type and the place where it was preserved should appear in Roman characters—Latin characters—rather than Chinese or Japanese.

**Stevens** noted that it could be a little bit complicated because on some Chinese sheets the collector’s name was in Chinese and there was no transliteration.

**Prop. C** was **accepted**.

**Prop. D** (93: 17: 5: 0) was **accepted**.

### Recommendation 37B

**Prop. A** (8: 101: 1: 1) was ruled **rejected**.

**McNeill** moved to the series of proposals on sanctioned names that were outlined earlier on.

**Lendemer** requested clarification as to whether the issues would just be discussed or if the Section would be voting on it today, because he wanted the evening to read it in detail.

**McNeill** clarified that this would be the debate on it and the decisions on the grounds that it had been distributed.

**Lendemer** moved that the discussion be postponed until the following day to allow people the evening to read such a complex proposal.

**May** thought that due to the complexity and importance of the Art. 59 proposals it might be appropriate to introduce it now to allow time for people to digest it.

**Redhead** decided that the proposers were not prepared at this point to talk about Art. 59, but wanted to point out that either tomorrow or the next day there would be a group presenting from the floor a proposal, which would be discussed with the other published proposals in Art. 59, and they had supplied on the registration desks copies of this to allow the members of the Section to look at it overnight in advance of it being discussed. He highlighted that there were many copies of a new proposal on Art. 59, which would be discussed in conjunction with the published proposals that were on the agenda.

**McNeill** added that this meant that when Art. 59 did come up for discussion no-one would be in a position to say “But wait a minute, we haven’t read it”, as ample opportunity would had been given.

**Redhead** added that there were other documents also available—the Amsterdam Declaration and a rebuttal to the Amsterdam Declaration—all pertaining to Art. 59.

[*A short discussion of Art. 38 Prop. A occurred here and has been moved to the normal order in the Seventh Session on Thursday morning.*]

**Knapp** thought that the proceedings had come to an impasse, where there were a lot of difficult things to be dealt with that were not worth even trying to deal with in the 20 minutes maximum left for the day. So she let everybody off early, but with the proviso that, if it was not all finished in time, everyone would have to come back on Saturday for Saturday detention in the Nomenclature Section! [Laughter.]

## Seventh session

Thursday, 21st July, 2011, 9:00-12:45

**Knapp** welcomed the Section back with two items of news. She proudly reported that the Section had made it into the journal *Nature* and noted that in her considerable experience with *Nature* it was incredibly rare that systematic botany got a mention, so she suggested that we should be incredibly pleased with ourselves for having done that. The second thing was that she had made a little tot-up of how many proposals had been done and how many were left to do and the good news was that the Section had dealt with 130 proposals so far and the slightly bad news was that 70 proposals remained, not including the sanctiotypification proposals and Art. 59, which were going to be slightly revised, plus 28 proposals to amend the glossary, which were very helpful. She concluded that there were essentially 100 things left to do, slightly more than half-way done and more than half-way done with our time. She exhorted the Section to concentrate their minds, be brief, not repeat themselves and if people were saying the same thing to restrain themselves.

### Article 37*bis* (new)

**Prop. A** (60: 25: 7: 10).

**McNeill** introduced Art. 37*bis* (new) Prop. A, a proposal from the mycological community to require that organisms treated as fungi under the *Code* should have the citation of an identifier issued by a recognized repository in the protologue as an additional requirement for valid publication.

**Hawksworth** pointed out that the details of the proposal had been worked out very extensively by consultations involving a large number of mycologists, and that after they had been published in *Taxon* they were discussed at the International Mycological Congress in Edinburgh last summer where they were unanimously supported. He announced that something like 80% of new fungal names were already being registered voluntarily in the system according to this proposal.

**Norvell** suggested two friendly amendments: in Art. 37*bis*. 1 to substitute “the” for “an” after “the citation of” and add the phrase “for the name” after “repository” so that the text read “…the citation of the identifier issued by a recognized repository for the name (Art. 37*bis*. 3) in the…”, and in Art. 37*bis*. 2 add a phrase after “(b–e)” that would read “when accessioned and published information for an identifier differ, the published information shall be considered definitive”. [This was considered a **friendly amendment** by the proposer.]

**Knapp** checked that what had been added was correct.

**Norvell** clarified that it would be a lower case “w” and a parenthesis after “e”.

**Knapp** joked that it was close enough for government work but it was never close enough for the Nomenclature Section. [Laughter.]

**Demoulin** disagreed with his friend Hawksworth on this and especially on the fact that it had been unanimously supported at the last Mycological Congress, because he was there and he had never accepted this as an Article. He could accept it as a Recommendation, but as an Article he thought it was premature. He felt that after 12 years the vampire was rising from his grave again! He had personally always been in favour of some form of registration as a means of making several registering centres aware that a publication existed. But he argued that this went into a lot more detail on what should be done and whether it was effective or valid publication. He feared that the result was the same: “you can’t use the name”.

He suggested that the Section think about what had happened every time a new requirement for a valid publication had been added in the *Code*. With the Latin diagnosis, the type designation, there had been a period of time before everybody applied the new rule, which lead to a number of names that sometimes were very well presented but lacked a single requirement for valid publication, which led to posterior publications, sometimes by the publishing author, who had been made aware that he should add something. Sometimes, he added, this was also done by predators, who took the opportunity to add their name and make a new publication by validating other things.

He thought this was an unfortunate situation and having special rules for fungi should be avoided as much as possible. He acknowledged that he had himself presented things that were special rules for fungi, but only ones he considered unavoidable because of historical reasons, like the sanctiotypification story, or absolutely urgent political necessities, like the change in title or the English diagnosis. But he did not really see the need for fungi to have a special rule on a matter like this.

If some day there was a general agreement on registration, or even this step further of deposition, he felt that the Section should be aware that at the moment it could be a Recommendation because a repository existed. He felt that making it a requirement meant a monopoly was being given to the single existing repository at the moment and there could very well be people who objected to working with the website. He referred to people like himself, who just hated having to navigate an airline company, university, congress website and thought that the concept of websites were mutants with a different mind than his. His last comment was that people also would not like to give their name to give bigger power to the controversial director of the CBS.

**Buck** felt that this was obviously registration rearing its head again and he thought the problem was that, for most who had traditionally been against registration, the effective date of publication was tied to the date of registration rather than anything else. His biggest problem was that this identifying number was often a MycoBank number, even though it was not listed by name. Yet from his understanding, if you were at an institution that did not have ready access to the internet, it was not possible to register a MycoBank name by filling out a form on paper and mailing it in. So that really limited the taxonomy to those in more developed countries, and he thought it kind of reeked of imperialism. He was against the proposal.

**Kirk**, before he supported the proposal, wanted to correct two inaccuracies. He stated that there were now two options for registering names and in the next week there would be a third. He would be in Beijing helping the Chinese mycologists launch a Chinese-language portal for registration. Second, in the second portal for registration, which was centred on *Index Fungorum*, the global nomenclator for names of fungi, there was nothing to suggest that one should go online to register the name. A simple e-mail to him would guarantee an identifier for the proposed name.

He supported the proposal—86% of names produced by mycologists were being registered voluntarily. He argued that this clearly suggested that they wanted it to be part of the *Code*.

**McNeill** interjected that the Nomenclature Committee for Fungi voted 11 to 3 in favour of the proposal.

**Ulloa** agreed with Demoulin and Buck that registration was debated and defeated strongly in St Louis with another audience that was more internationally representative than in Melbourne. She was totally against the proposal and noted that there had already been a lot of concessions made for the fungi people. She referred to the decision about entering English yesterday that was after a fungi proposal and expressed the opinion that the Section had had very little time to discuss the issue and the vote was called without letting people in the audience discuss that proposal.

**May** clarified the percentage—86% of mycologists attending the IMC were in support of the proposal. He also proposed a friendly amendment to delete the asterisk and the phrase “The only current operational repository appointed was MycoBank”, because the word “appointed” was incorrect, it had not yet been appointed, and secondly there were other operational repositories. [This was considered a **friendly amendment** by the proposer.]

**Gams** begged to disagree with Demoulin and strongly defended the proposal. He highlighted that the mechanism was working in practice and most of the journals were requiring this deposition and the text was certainly not written as a Recommendation but could only be handled as a requirement in an Article. The only thing that worried him was a letter of concern in *Mycotaxon* by Morris and co-workers, who had made very constructive suggestions of adding mechanisms that assured that the MycoBank entries were in as perfect as possible agreement with the published matter. He considered what Norvell had proposed was already a concession to the suggestions and endorsed all the suggestions made in that paper.

**Marhold** was not a mycologist and did not think that it was an exception for mycologists, he felt it was a good example that registration could work and that “we should skip any ideology and emotions and stop hunting for witches and start to discuss [the] matter”. He acknowledged that there were now a lot of options to publish and he thought it was high time to find some mechanism that would register names and make the community aware of the names. He added that the fact that something was defeated 12 years ago did not mean that it was defeated forever. He certainly doubted whether international representation here was less representative than it was in St Louis.

**Stuessy** agreed with Marhold that it was probably inevitable that registration would eventually take place, so suggested taking a practical view and looking at this as a pilot project. He advocated letting the mycologists run it, seeing how it went and finding out what the problems were. He felt that would then offer a better solution to address the broader question for all the other groups.

**Penev** noted that there already were pilot projects with *ZooKeys*, where for the last two years all the taxa were registered in ZooBank by the publisher, and also in *PhytoKeys*, where it was mandatory, so he did not think it was a big problem at all and highly supported this proposal.

**Lendemer** described himself as a lichenologist who published, edited and actively described new taxa and new combinations etc. He quite regularly used *Index Fungorum* and MycoBank, which were the two repositories that were currently active.

In reply to Kirk’s point that the third option would be available in a week, the bottom line was that what was being discussed were the two things that were currently active, not a third thing that may or may not actually become active in a week. He added “we can’t talk about the future; we can only talk about the present”.

He continued that MycoBank was the only one of the two currently working repositories that actually gave a number immediately upon deposition. [Initially he thought that *Index Fungorum* did not, but in his comment slip he wished the record to be corrected that *Index Fungorum* did automatically give a number.] His point was that most journals were requiring MycoBank and he considered MycoBank to be the more applicable of the two, but the unfortunate thing was that he felt MycoBank was very clunky for use for groups that did not use cultured fungi as types. He found that the interface that was involved for putting in type information and other aspects of the protologue, which were required to generate a number at the end to submit with the manuscript, required fudging different aspects of the type information and it did not really offer a good way to input all of the information normally used in citing a type specimen for some groups of fungi.

He also thought it was important not to confuse compliance with a mandate. He referred to the number everyone had been citing—86% of mycologists were doing this, or 86% of mycologists supported this and a certain percentage of mycological names being published were currently doing this—but he did not think this was helpful. As the editor of a journal that used MycoBank numbers for every new combination, every new taxon proposed, he did not think it should be a mandate. He submitted all these things to MycoBank and got the numbers himself, but he did not think that authors should be forced to do that as a condition for publication.

**Gandhi** agreed that we should not be dwelling in the past. He pointed out that we had overcome the electronic publication requirement and the Latin requirement. His personal belief was if the majority of the mycologists preferred a kind of registration their opinion should be respected.

**Applequist** felt strongly that whatever your opinion of registration, the basic rules of the *Code* should be the same for all included groups, except where necessitated by taxonomic history or life-history traits. Otherwise what we have was not a botanical and mycological code; it was a botanical code and a mycological code, schlepped together into the same book.

**Greuter** proposed a friendly amendment as apparently no-one had noticed that the Article did not say what it was. It was placed, as suggested, between two Articles dealing with names of new taxa, but as he understood it, it was to work for all new names and this must be said, otherwise it was not clear. So the amendment could be just at the beginning to insert, after “For”, “new names of”. [This was considered a **friendly amendment** by the proposer.]

**Gereau** noted that mycological colleagues gave the assurance that the registration system was working quite well on a voluntary basis, and he suggested letting it remain on a voluntary basis.

**Wiersema** wanted it to be on record that the mycologists at his institution, which happened to be the second largest mycological collection in the world, were supporting this registration of names.

**Redhead** pointed out that this was an opportunity for mycology. He noted the advantages of having *Index of Fungi*, and then *Index Fungorum* and then MycoBank and the exchange of information between the two of them, and the long history of accumulating and databasing names so that something like 90-some per cent of the fungal names were available electronically in some form. He asked the Section to consider the human cost of having people track this, of receiving journals, and now publishing electronically, the costs of trying to track all the names, and he suggested there was a chance of losing the opportunity to have a complete list of names and he wanted to keep the momentum going and keep continuity of the fungal names. He highlighted that if you were looking for a potential homonym for a plant or algal name the list could be consulted. He was very strongly in support of this proposal as a mandate.

He also noted that the International Mycological Congress had a very international attendance, being held in Edinburgh, close to Europe, there was a good representation of mycologists there and the majority were in support of it.

**Funk** requested clarification regarding a rumour going around that there was an egregious breach of confidentiality with the fungal registration and that a competing lab was notified of something and they were able to publish it ahead of time. She wanted to know if that was true and, if so, had it been fixed so that it could not happen again?

**Norvell** confirmed that had happened, but it was only one event and it happened in December and the individuals involved solved it immediately. She added that *Mycotaxon* had changed its policy so that the MycoBank number should not be applied for until after the paper had undergone final review but before final submission to the Editor-in-Chief.

**Fortunato** did not work with fungi, but she supported the proposal and thought that the people who worked in her country and other developing countries also agreed with it.

**Janarthanam** thought it was good proposal, but raised doubts about the number of repositories there were in India, as the present National Biodiversity Act did not allow people to send any living material outside the country.

**Knapp** clarified that the proposal was about registering names, not having repositories of material.

**Janarthanam** had misunderstood and apologized.

**Rico** referred to the value of 86% and wanted to know how the people who did not attend were considered, whether they were asked by ballot vote, or some other process.

**Norvell** explained that there was a three-day Nomenclature Section and also questionnaires that were disseminated among all participants who attended the Congress in Edinburgh. These were disseminated and around 160 ballots were returned. She added that everybody who went to the Congress had the ability to vote and/or attend the Section via both a show of hands and the actual written ballots.

**Rico** wanted to know about the people who did not attend the Congress, because they were unable because of budget or something like that, were they considered?

**McNeill** thought that the short answer was “no”.

**Lendemer** called the question. [There was a sufficient majority in favour of voting.]

**Prop. A** was **accepted** as amended.

[*The following discussion, pertaining to a new proposal by Marhold concerning Art. 37bis, took place during the Tenth Session on Friday afternoon.*]

**Marhold’s proposal**

[The proposal was submitted electronically and read: “Add words “and as vascular plants” to the Art. 37*bis*. 1 in the following way: 37*bis*. 1. For organisms treated as fungi under this *Code* (Pre. 7) and as vascular plants, from 1 January 2013 the citation of an identifier issued by a recognized repository (Art. 37*bis*. 3) in the protologue is an additional requirement for valid publication.”]

**McNeill** introduced a proposal by Karol Marhold on Art. 37*bis*. He summarized that it had been agreed by approving the proposal to establish in Art. 37*bis* that we would have registration of fungal names, and there was now a proposal coming from Marhold. [The **proposal** was **seconded** and **supported** by three others.]

**Paton** strongly approved of registration, but thought it was premature to go for it before there was some way of declaring how registration would be run sustainably and a plan to go forward.

**McNeill** agreed.

**Funk** proposed that a Special Committee be set up to examine registration of plants and how it might be implemented in the future.

**Knapp** suggested that the Section first needed to vote on the proposal and then she could make that proposal if it failed.

**Funk** agreed that was fine but noted it might be faster. [Laughter.] In which case she would vote “no” on the proposal.

**Challis** thought a Special Committee on registration had already been established.

**Knapp** clarified that it had not yet been voted on.

**Gereau** stated that there was no mechanism proposed, no method of assuring the perpetuity of the process, and there was no funding mechanism proposed. He felt it was completely unacceptable at this time.

**Greuter** wanted to be able to vote “yes” on the proposal but could not as it was formulated. He moved an amendment, possibly friendly, to change “2013” to “2018”, which would be after the next Congress. [Laughter. That was **accepted** as a **friendly amendment**.]

**Applequist** noted that there was still no guarantee that such a repository would exist in 2018, but she felt that a proposal of this magnitude should have been proposed in advance and subjected to a preliminary mail vote, not dumped on the Section at the end of the last day. [Applause.]

**Nic Lughadha** reiterated the advantage of having registration approved for fungi and the opportunity to see how that went and adopt the same rules, or slightly different arrangements, for registration for vascular plants in six years’ time, whereas Greuter’s amendment would jump the gun and assume that it was going to work in just the same way for vascular plants as for fungi. She opposed the whole proposal.

**Cafferty** called the question. [There was a sufficient majority in favour of voting.]

**Marhold’s proposal** was **rejected**.

[*Here the record reverts to the normal sequence of events.*]

### Recommendation 37*bis* A (new)

**Prop. A** (56: 24: 8: 11).

**McNeill** turned to Rec. 37*bis* A (new) and checked to see if there were any friendly amendments or changes before discussion started.

**Norvell** noted that part of the Morris & al. manuscript had suggested an insertion of a following Recommendation and wondered if it should be considered at this time. They wrote: “The author of a manuscript should request identifiers for names proposed in that manuscript after peer review had been concluded.”

**McNeill** thought that it should be a separate proposal.

**Hawksworth** started to point out that it was actually already there…

**Knapp** exclaimed “Excuse me, excuse me, wait. David, don’t start talking until I recognize you or I’ll send you out!” [She then relented and recognized him.]

**Hawksworth** replied “Sorry, ma’am. Do I get detention after?”

**Knapp** responded in the affirmative.

**Hawksworth** clarified that just before (b), the suggested insertion was already covered where it said “as soon as possible after the papers were accepted for publication”.

**Knapp** confirmed that the Section could discuss that when that new Recommendation came up as proposed, later, and focused the discussion on Prop. A, for the new Rec. 37*bis* A.1, which started “Authors of names of organisms blah, blah, blah, blah, blah.” She added that it did not literally read “blah, blah, blah”.

**Greuter** apologized for continuing to interfere but he was always wary of wording ambiguities and in this case it was the word “minimal” under (a), which could be read in two opposite senses. It could mean at least minimal, or it could mean only minimal. He suggested that this should be clarified or perhaps rather than “minimal” one could add some other words, like “essential”. He asked the proposers to specify whether they meant at least minimal, or whether they meant only minimal.

**Kirk** explained that the “minimal” meant those elements that were required for valid publication: the name, the author, the diagnosis and information about the type for a new taxon, the basionym and its reference for [a new] combination etc. “Minimum” was in the context of *Code* mandated requirements, rather than illustrations, discussions, lists of references, gene sequences blah, blah, blah, which were not required by the *Code*.

**McNeill** suggested that for purposes of debate “minimal” be replaced by “the required” and the Editorial Committee could specify by reference, what the requirement actually was.

**Kellermann** noted that in 37*bis*. 2 the minimum elements for information that must be accessioned were named because it said “are those required for valid publication”.

**Alvarado** thought it would be fine to specify required elements in writing, because it could be interpreted in different ways by different people.

**McNeill** assured him that the Editorial Committee would make sure it was unambiguous.

**Lendemer** pointed out that depending on what system was used to get your number there were different elements that were required, not necessarily what names were being used, but for information for type specimens different elements were required. And he wanted to call the question.

**Knapp** did not feel that was allowed unless he stood up the second time. But she agreed that there seemed to be no more comments. She added that the same person could not call a question more than twice in a day—it was a new rule! [Laughter. There was a sufficient majority in favour of voting.]

**Prop. A** was **accepted** as amended.

[*At this point discussion of Art. 7 Prop. L took place, the proceedings of which can be found under Art. 7 in the normal order under the Second Session on Monday afternoon.*]

**Norvell’s proposal**

**Knapp** introduced a proposal from the floor for Rec. 37*bis* A (new): “The authors of a manuscript should request identifiers for names proposed in that manuscript after peer review has been concluded and the manuscript has been accepted for publication.” [The **proposal** was **seconded** and **supported** by three others.]

**Redhead** outlined that the Recommendation was a result of some positive critiques of the proposals that were published, or about to be published in *Mycotaxon*, and several people had looked at it and agreed that the polishing was appropriate and good.

**Dorr** felt that it was starting to get a little bit ridiculous. He wasn’t sure if anybody remembered the Reid Moran article about a new county record in which the text was “I got it there then”, and then the acknowledgements went on for pages. He felt that we were starting to set up requirements for people to make nomenclatural action, yet all the steps that they need to do were being prescribed, short of “The author should use a computer of such and such power and keyboard of such and such dimension”. He thought it was becoming very silly and trivial.

**Redhead** explained that this was being proposed because there were potential changes between when a number was obtained and when [a name] actually came out, and it was thought that there was a potential for making mistakes, and so this would take care of those situations.

**Norvell** wanted to make clear she was doing this at the request of the author. If it was regarded as trivial that was fine, but she wanted to make certain the author’s position was expressed here on the floor.

**Penev** did not think it was authors but the publishers who should do that after the manuscript was accepted.

**Paton** had fed into the paper that proposed this and thought it may already be covered under the first Recommendation, because it did say after the paper had been accepted for publication in the first Recommendation. So he thought it may not be necessary.

**Norvell’s proposal** was **rejected**.

**Prop. B** (55: 24: 9: 11).

**McNeill** introduced Rec. 37*bis* A Prop. B, which required that information or other nomenclatural acts be added to the record of the name. He noted that whereas Prop. A received 11 to 3 support from the Committee for Fungi, this one received almost the same, not quite, with one abstainer: 10 to 2 in favour of Prop. B.

**Reveal** noted a minor editorial detail that “record number” should be changed to “identifier” in the last line.

**Prop. B** was **accepted**.

### Article 38

[*The following debate, pertaining to Art. 38 Prop. A, took place during the Sixth Session on Wednesday afternoon.*]

**Prop. A** (55: 5: 16: 19).

**McNeill** introduced Art. 38 Prop. A, where Doweld had noted that there was a slight gap due to a change in wording on the Article dealing with the need for an illustration as a type of a name of a fossil plant. There was a period of time in which names that were thought to be validly published were, in fact, not and proposed this amendment to the *Code* to plug that gap. McNeill reported that it had considerable support—55 to 5 in the mail vote—and it was looked at by the Committee for Fossil Plants, who agreed by a vote of 7 to 3 that it was desirable, although they were less enthusiastic about his Examples, but those would go to the Editorial Committee anyway and the fossil plant people would offer advice. Proposal A was the proposal; Prop. B was the Examples.

**Herendeen** from the Committee for Fossil Plants pointed out that this was a technical problem and the solution would fix it. He did not think the Section needed the history on it. He noted that the couple of Committee members who voted against it were questioning just how many cases there might be lurking out there that would fall into the gap. He noted that they did know of one and it was a conservation proposal, so this would fix a problem and, while it was not known how big the problem was, he felt it was better to fix it.

**Prop. A** was **accepted**.

**Prop. B** (21: 21: 36: 19) was ruled referred to the **Editorial Committee**.

**Prop. C** (28: 33: 16: 20) was ruled **rejected** as a corollary of Art. 8 Prop. B, which was rejected.

[*Here the record reverts to the normal sequence of events.*]

### Article 41

**Prop. A** (48: 19: 34: 3) and **B** (45: 17: 38: 4) were referred to the **Editorial Committee**.

**Prop. C** (11: 68: 23: 0).

**McNeill** noted that Prop. C received quite a strong vote against: 11 to 68, 67% against. He added that it dealt with names above the rank of family and it seemed to the Rapporteurs that it was based on some misconception but was seeking to increase the restriction as to the requirements for the publication of such names.

**Reveal** agreed that the Rapporteur was quite correct, what was in the proposal was exactly what everyone already did, and the last line, which seems to be of a bother to some folks, above “diagnosis and description must be in Latin” was exactly what was required today. Because names above the rank of family were not in Art. 41, where there were exceptions to the rest of Chapter II, all provisions in Chapter II applied—as decided by the Editorial Committee back in 1989 or ’90 when Ruud Hoogland and he were starting this work. Therefore a Latin requirement was necessary, so the only thing the proposal did was just add in what was already done.

He suggested that the other item that may be of some interest was that there was nothing in the *Code* about what was required to validate a name above the rank of order. In theory, you could use a description of a variety as a validating reference to an order or above, and as a result in 1981 Cronquist began to characterize orders using descriptions of monospecific genera in monogeneric families and create monofamilial orders. This tradition that he started of using this provision of validating an ordinal name on the only species in that order had been followed. Instead of everyone having to just know what the tradition was, we were proposing actually including it, so nothing changed from what was already being done at present.

**Turland** responded to the proposer’s comment about the last sentence of the proposal. He argued that it would in fact change the current situation, because currently it was possible to have a suprageneric or, in this case, suprafamilial name that was not the name of a new taxon but effectively a renaming, a name at a new rank. He gave the example of an ordinal name based on a familial name, and because such names can be renamings, they were exempt from the Latin requirement because they were not names of new taxa. He explained that this proposal, with the last sentence, would have the effect of invalidating an unknown number of suprafamilial names. The function of Art. 41 was to restrict the ranks of the taxa to which the previously published validating description or diagnosis applied, so it did not really have the effect of exempting, or otherwise, the Latin requirement.

**Redhead** wondered if the proposal was supported, was it open-ended and would this then require Latin descriptions in the future?

**McNeill** responded that there was no change suggested in the Latin description, noting that Art. 41 did not deal with that. He stated that Reveal was perfectly correct, that a new taxon at any rank required a Latin description at the moment. The Rapporteurs’ point was that it was already covered in the *Code*. The case was that many names at higher ranks were not names of new taxa; they were renamings of existing taxa at a different rank.

**Redhead** questioned whether there was currently a limitation now: from 1935 forward Latin was required and from 2013…

**McNeill** explained that a renaming must be that of a validly published name. So if the previous name was published before 1935 without a Latin description then it was validly published. In other words, you could publish a name today at the rank of superorder based on an order that was validly published, even if that order validly published had an English description because it was in 1934.

**Reveal** asked Turland for confirmation that, in his opinion, this name would indeed be valid because it was a new name and a new rank, which overturned the opinion given back in the early ’80s—late ’80s or early ’90s.

**Turland** responded that *Malvaceae* Adanson was not validly published in 1763 because that was before the starting point for suprageneric names, so it could not be a renaming of *Malvaceae* Adanson.

**McNeill** noted that did not mean it could not be validly published…

**Turland** added that it could be validly published as a renaming based on something else though. Presumably *Malvaceae* was validly published prior to…

**Reveal** suggested we pretend for convenience’s sake that it was *Malvaceae* Jussieu 1789.

**Turland** agreed that in that case, *Malvidae* of Wu was indeed validly published as a name at new rank, a new status, based on Jussieu’s family name.

**Prop. C** was **rejected**.

**Prop. D** (3: 24: 42: 0) was referred to the **Editorial Committee**.

**Prop. E** (27: 21: 56: 0).

**McNeill** noted that Prop. E had a Note as well as an Example, and the Rapporteurs commented that the Note did not belong here, but rather in Art. 16, and that it would be a helpful addition to the *Code*. He suggested that if the Section were to vote in favour of it, it would be assumed that it would be placed in the most appropriate spot editorially. The mail vote was very heavily in favour of a reference to the Editorial Committee, doubtless with that in mind: 27 for, 21 against and 56 to refer it to the Editorial Committee.

**Buck** questioned whether, according to the Rapporteur’s comments, this new Note would not affect the current *Code* at all.

**McNeill** thought that if it was genuinely a Note then it stemmed from the existing Articles of the *Code*, something that was implicit in the *Code* but not explicit.

**Buck** suggested it should probably go straight to the Editorial Committee.

**Knapp** confirmed that a “yes” vote would be so interpreted.

**Prop. E** was referred to the **Editorial Committee**.

**Prop. F** (29: 37: 38: 0), **G** (26: 37: 39: 0), **H** (7: 75: 22: 0) and **I** (33: 29: 41: 0) were referred to the **Editorial Committee**.

### Article 42

**Prop. A** (29: 50: 36: 0).

**McNeill** introduced Art. 42 Prop. A, which was a rewording of one component of the Article. The intent of this was to make it clearer, define more precisely, what constituted an illustration with analysis, or rather the analysis of an illustration. There was a grammatical defect in the proposal, but that was purely editorial. He reported that the proposal was not very strongly supported: 29 in favour, 50 against, with 36 recommending it go to the Editorial Committee.

**Nic Lughadha** requested a reminder of the new definition of illustration that had been agreed on earlier in the week, because it might be relevant in this context. [Art. 8 Prop. A was displayed.]

**Buck** objected primarily to the fact that it said you had to have more than one figure, and if your single figure was diagnostic there was no reason to have more than one. That was why he had voted against it to begin with.

**Barrie** wondered why this was being restricted to vascular plants.

**McNeill** replied that it was because there were provisions for unicellular organisms—algae—that were separate. Article 44.2 was the one that covered non-vascular plants.

**Nic Lughadha** was still not getting enough sleep, but she thought this meant that an analysis could now be a photograph and she found that new and unusual. She had always understood it to be a figure with detail, and now it looked like the door was being opened to pointing at a photograph instead of a description in certain rare instances.

**Prado** clarified that the proposers’ intention was to clarify for vascular plants what was meant by “analysis” in the *Code*. He elaborated that sometimes it was very difficult to decide if a drawing representing a vascular plant was [adequate for a name to be] validly published or not, and one Example was presented to help to understand the modification. The proposal had three parts: A, B and C; B was a clarification about caption. He was not sure if he had proposed the correct wording of the Article, but he thought it needed improving in the *Code* because it was hard to use this rule.

**Stevens** really could not see what the proposal added.

**Soreng** agreed that there was a problem with using photographs as types. There were a lot of old photographs of specimens at Berlin and if they could be used as types, he would be scared.

**McNeill** reassured him that we were not really discussing types but what an analysis was in the phrase “illustration with analysis”.

**Gereau** felt that anyone who had worked with a great deal of the literature from the late 18th century through much of the 19th century would probably have encountered great difficulties in deciding whether a given illustration really was an illustration with analysis, and he felt that clarification was highly desirable. He thought the “at least one additional figure” clause here was very clarifying and desirable. He acknowledged that the proposal as written had some problems but it should certainly not be rejected and he would like to see it referred to the Editorial Committee.

**Gandhi** noted that in his line of work, to determine the validity of a published name, all he looked for whenever there was a fig involved was whether there was an additional figure cited in addition to the main figure. He did not go into detail about whether the cited additional figure was one or a group of figures that provided the analysis. He believed that it was beyond the requirement of an indexer.

**Knapp** asked whether he was supporting or opposing the proposal.

**Gandhi** [Laughing] responded that in the beginning, as Stevens had said, it was not clear to him how it changed the *Code*, but he supported it.

**Davidse** was not so against it in principle, but did not particularly like the phrase “the plant habit plus a detail of a segment, neither diagnostic” used in the Example. He interpreted that those were now determined as not diagnostic. He thought it was necessary to be neutral and consider that the original person thought that they might be diagnostic.

**McNeill** noted that the clause would be deleted by the Editorial Committee because there was nothing in the *Code* to specify that a description had to be diagnostic, so why should an illustration?

**Davidse** agreed that that was exactly the point that he was trying to make.

**Prop. A** was **rejected**.

**Prop. B** (42: 46: 27: 0) was **rejected** as a corollary of Art. 42 Prop. A being rejected.

**Prop. C** (35: 48: 30: 0) was ruled referred to the **Editorial Committee**.

**Prop. D** (18: 68: 22: 0).

**McNeill** introduced Prop. D, by Brummitt, which was a similar restructuring, in this case trying to do something that on the face of it looked very desirable: that was to avoid so much repetition as currently exists in the *Code* with regard to illustrations with analysis. However the Rapporteurs had pointed out that it had some difficulties. It had been pretty well defeated in the mail vote: 18 in favour, 68 against.

**Prop. D** was **rejected**.

**Prop. E** (12: 92: 6: 0) and **F** (7: 97: 4: 1) were ruled **rejected**.

**Prop. G** (34: 22: 47: 0) was referred to the **Editorial Committee**.

### Article 43

**Prop. A** (7: 4: 96: 0) was ruled referred to the **Editorial Committee**.

### Article 44

**Prop. A** (9: 79: 16: 1) was ruled **rejected**.

### Article 45

**Prop. A** (45: 11: 47: 1) was ruled referred to the **Editorial Committee**.

**Prop. B** (54: 7: 27: 11).

**McNeill** noted that Art. 45 Prop. B, by Demoulin, was linked to proposals on *Microsporidia*, and he was rewording and adding a section, restricting the present Article to the first sentence and transferring the rest into a new Art. 45.5. McNeill added that the Nomenclature Committees for Algae and Fungi were both asked to comment on the proposal, or more correctly the Committee for Fungi was, and inadvertently the Rapporteur wrote Prop. A instead of Prop. B for the Committee for Algae, who therefore did not actually vote on it but did express their support for the proposal. The Committee for Fungi did vote on it and voted 10 to 1, with some abstentions, in favour of the proposal. That was a 71% vote in favour.

**Demoulin** added a small word of explanation that this came not only from reflection after the Mycological Congress on how to handle the *Microsporidia*, but also from another meeting, which was devoted to blue-green algae and where there was discussion on the nomenclature of such organisms treated under different *Codes*, and discussion with Professor Oren from the Bacteriological Nomenclature Committee, who was very satisfied by the way the botanists were handling the situation but noted that there still were some loopholes of the kind that Redhead had pointed out in the case of *Microsporidia*.

So Demoulin had tried to devise an absolutely general provision for those cases of the algae and fungi that could be sometimes treated as protozoa. This was an old classical situation, the dinoflagellates, but there were more and more organisms that people were hesitant to place in one group and so this was the result. It was a completely general way of having portability of names from one group to another and Professor Oren had told him that it was a good way of handling it and it had been well supported by mycologists and algologists.

**Reveal** was a little concerned by the phrase “nonbotanical *Code*”. He felt this suggested that he could write his own personal *Code*, say he was following it—it did not deal with botany, but he was describing fungi and algae. He suggested the inclusion of some modifier like “Internationally accepted”, or “established”.

**McNeill** thought that was a very good point, which the Editorial Committee should address.

**Redhead** felt, in general, that there was support for this at the International Mycological Congress. He was in the Committee for Fungi and also supported this as a broad approach to many of the problems being run into with organisms that may not have been traditionally viewed as fungi.

**Prop. B** was **accepted** with a supermajority.

[*At this point discussion of Preamble Prop. A took place, the proceedings of which can be found under Preamble in the normal order under the Second Session on Monday afternoon. A short discussion pertaining to Art. 33 Prop. B occurred here and has been moved to the normal order in the Sixth Session on Wednesday afternoon.*]

### Article 46

**Prop. A** (53: 41: 11: 1).

**McNeill** noted that the proposal was by Brummitt, and while it followed from the Brummitt proposal that had been rejected, it could be considered separately. He summarized that it was proposed that names above the rank of family would not have the suggestion that they required authorship. The vote was 53 in favour, 41 against, so it was not roundly defeated.

**Greuter** pointed out that the proposal was not an Article as it was worded because it did not say “are not to be used”. He moved that it be treated as a Note, the added new sentence of which was just a statement of fact.

**McNeill** thought it had to be an Article and should read “are not to be”, because the provision at the moment of Art. 46 did not mention ranks but taxa, so there was no provision in the *Code* to say that those names for which priority did not apply did not need to have an author or may not have an authorship.

**Greuter** felt that that was an amendment to his amendment.

**Knapp** clarified that McNeill was leaving Brummitt’s proposal unamended. She asked if Greuter was formally proposing to amend this to a Note.

**Greuter** proposed McNeill’s amendment in his name. [The **amendment** was **seconded**.]

**Knapp** instructed the recorder to add the words “to be”.

**McNeill** corrected that to “Are not to be”.

**Knapp** became philosophical, quoting Shakespeare, “To be or not to be, as it were”.

**Reveal** had no objection to this although he thought it would cause a little confusion when trying to figure out what to do. He suggested that if it was going to be a Note, instead of “not”, to make it optional to a user whether or not they would use author names. So “Author citations…”, instead of “are not to be used”, “Author citations may not be…”, in order to allow the choice.

**McNeill** suggested it would be a Recommendation to that effect, that they should not be used.

**Reveal** agreed that probably a Recommendation was appropriate, if Greuter had no objections, that [author citations] need not be used above the rank of family.

**McNeill** asked if Reveal wished to propose something.

**Reveal** thought that Greuter had allowed the proposal to become a Recommendation rather than a Note.

**McNeill** disagreed and clarified that it was currently an Article.

**Reveal** then decided to re-amend the amended amendment to read, as a Recommendation, “Author citations are not necessary to be used after names of taxa above the rank of family” to be fixed by the Editorial Committee.

**McNeill** added “should not”.

**Knapp** repeated that the proposal was to amend this to be a Recommendation instead of a rule.

**Reveal** agreed, noting that the new wording should allow one the choice of doing it or not doing it. [The **amendment** was **seconded**.]

**Knapp** noted that discussion was on the amendment, from being a rule to being a Recommendation.

**Redhead** thought we were originally discussing the original amendment…

**Knapp** said it was withdrawn.

**Redhead** said it was not.

**Knapp** clarified that it was turned into a Note.

**Redhead** thought we only should be discussing the original amendment but now an amendment had been made that countered the…

**Knapp** explained that the original amendment was to add the words “to be” and returned the discussion to the words “to be”. She apologized and noted that it was getting a bit confusing because the proposer was not present.

**McNeill** added that it would almost certainly have been a friendly amendment but unfortunately with the proposer absent it could not be.

[The **amendment** was **accepted**, so the words were changed to “to be”.]

**Knapp** moved on to Reveal’s amendment, which was to change this from a rule to a Recommendation.

**McNeill** clarified that the Recommendation had to be “should not” because there was no obligation to provide authorship. It was something that may be done if it was desirable, particularly in nomenclatural works.

[The **amendment** was **accepted**.]

**Knapp** moved to discussing Prop. A as a Recommendation and not a rule.

**Demoulin** agreed that it could be discussed independently of the rejected proposal but still believed it really only made sense if that proposal had been accepted. He felt that if the requirement of a description for valid publication of those names was maintained, he did not see why there should not be reference to who did the description, like for any other taxa. He did not see why the use of author names in that category should be discouraged.

**Gereau** agreed.

**Knapp** praised Gereau for being good and not saying what had been said before and requested that be recorded.

**Wiersema** was not clear what was being voted on. He was not sure if the entire Prop. A was being included or just the highlighted part as the Recommendation.

**Knapp** clarified that it was just the highlighted part.

**Wiersema** thought that had already voted been on.

**Knapp** explained that the Section had voted on turning that from a rule to a Recommendation.

**Wiersema** thought that had been approved.

**Knapp** explained that the change from a rule to Recommendation had been approved and now the vote was on whether the Recommendation was going to be included in the *Code*. She agreed that it had got a bit confusing and asked for a vote on including the highlighted text as a Recommendation in the *Code*.

**Turland** thought there was definitely a majority against.

**Knapp** agreed, but not a 60% majority.

**McNeill** said it did not have to be—there was a majority against.

**Turland** corrected him to a majority in favour.

**Knapp** decided to discuss it again to make sure it was absolutely clear.

**McNeill** stated that on the hand vote it was a clear majority, but not clearly a 60% majority.

**Knapp** reiterated that it was a clear majority in favour but not a 60% majority.

**Barrie** called for a card vote.

**Reveal** had a point of order in that his original suggestion for the Recommendation was not the wording being voted on. His suggested wording for the Recommendation would allow an author to either use or not use authorships.

**McNeill** noted that that was totally unnecessary, because that was exactly what the *Code* already said.

**Reveal** agreed.

**McNeill** asked if he should have just voted against the original proposal.

**Reveal** had done so, and would vote against this.

**Knapp** [later] reported the results of the card vote on Art. 46 Prop. A as a Recommendation that author names should not be cited above the rank of family. For was 244 votes, which was 51.8%, and against was 227 votes, which was 48.2%, which meant that it did not achieve a 60% majority so therefore the proposal failed.

**Prop. A** was **rejected** on a card vote (244: 227; 51.8% for).

[*The following debate, pertaining to Art. 46 Prop. B, took place during the Ninth Session on Friday morning.*]

**Prop. B** (42: 33: 28: 0).

**McNeill** introduced Prop. B, to amend Art. 46.2, which was addressing the situation in which an author did not actually ascribe the description to anyone and the presumption had to be that it was to be ascribed to themselves. He added that there was a very precise definition of “ascribed”, which did not cover a number of situations with respect to descriptions as opposed to names.

**Nic Lughadha** pointed out that the text on the screen did not match the text in the synopsis and made less sense than the text in the published synopsis. She pointed out that there was a “both” on the screen that did not belong.

**Knapp** agreed and thanked her, adding that “and” should also be struck out. She invited other comments or discussion on the proposal, which now matched the one in the synopsis, in case those in the audience did not have their synopsis and were counting on the projected version of the Bureau, which she considered to always be a fatal error. [Laughter.]

**Gandhi** still did not understand how it differed from what was already in the *Code*. He felt an Example to support it would be appreciated.

**Perry** explained that it was simply the fact that the Example in the *Code* said that the description was ascribed to an author, when in fact there was no mention of the author’s name with the description. So it was simply to make evident what the *Code* already implied but did not explicitly state.

**Applequist** wondered if this was a substantial difference, and wished to know how many names might change authorship as a result of the acceptance of the proposal.

**Perry** suspected none.

**McNeill** presumed this was based on her analysis of existing Examples in the *Code* and others. He added that there was a large number of Examples associated with this, which would be referred to the Editorial Committee whether or not this was approved.

**Prop. B** was **accepted**.

**Prop. C** (43: 36: 24: 0), **D** (39: 34: 28: 0) and **E** (26: 25: 55: 0) were ruled referred to the **Editorial Committee**.

**Prop. F** (30: 45: 29: 0) was discussed as an alternative to **I** [below] and **rejected**.

**Prop. G** (24: 41: 37: 0) and **H** (7: 14: 83: 0) were ruled referred to the **Editorial Committee**.

**Prop. I** (59: 22: 23: 0).

**McNeill** moved to Art. 46 Prop. I, which was adding a new sentence in Art. 46.2 and had associated Examples. He noted that the proposal had received positive support in the mail vote.

**Gandhi** referred to the Example, which illustrated how he had come across several situations wherein the publishing author and the ascribed author provided their own descriptive material towards the validation of the name. In such cases it became a problem as to whom the correct authorship should go and he felt that even though it was a technicality, it was still needed. He believed it should be the ascribed author who also provided some validating remarks.

**Gereau** thought that it would change the ascription of authorship in a great many cases—for example, this one, which would become “Buchanan-Hamilton ex D. Don”—and he found it misleading and undesirable.

**Perry** had a point of order: what happened to Prop. F?

**McNeill** apologized, he had said that Prop. F was an Example only but was, in fact, addressing the same issue but had a different approach to it. He did not think they should be looked at as alternatives but acknowledged that Prop. F should perhaps have been taken first.

**Knapp** thought that, since discussion of Prop. I had started, that should be completed and voted on and then discussion could return to Prop. F.

**Perry** felt that, despite what the Rapporteur said, they did address the same issue.

**McNeill** clarified that he said they do address the same issue but he did not think they were strict alternatives.

**Perry** suggested the Section look at them together.

**Knapp** felt that as they were not strict alternatives, discussion should continue to Prop. I bearing in mind that Prop. F was a different approach—but not strictly alternative—to the same issue.

**Wiersema** noted that this had been characterized as though it might create instability but he thought it helped eliminate uncertainty because these situations could be being treated both ways right now. He felt that this gave a clear way of dealing with them.

**McNeill** decided that he was inclined to think the proposals should be treated as alternatives, actually, so the discussion was extended to both proposals, and a vote would be taken as to which one was to be preferred and then the final vote to accept between Prop. F and Prop. I.

**Knapp** took the advice from the Rapporteurs and outlined that the discussion would be started over and instructed everyone to rewind the clock and start again discussing Prop. F and Prop. I as alternatives. She added that this meant doing the same as earlier: taking two proposals, choosing between them and then voting to amend the *Code* with the proposal that was chosen as the preferred proposal.

**Malécot** thought that Prop. F was dependent on Art. 32 Prop. I, which had been rejected a few minutes earlier.

**McNeill** acknowledged that it was slightly sloppy writing on the part of the Rapporteurs. He explained that, had that other proposal passed, this would have been critical for clarification. He thought it could still be considered independently.

**Demoulin** noted that the mail vote had rejected Prop. F and supported Prop. I, and he found that logical and would vote the same way, because he found Prop. I, even if it may destabilize a few situations, clearer and the most logical thing to do.

**Perry** asked Gandhi what he regarded as being the validating description in his proposal.

**McNeill** thought that the question arose from the fact that the Section had defeated a proposal that would define the validating description and had opted for leaving flexibility in determining typification so that either description could be conceived as a validating one. He interpreted the question as: “in your proposal’s wording, what do you mean by ‘validating description’, seeing as we have, in fact, not defined one”?

**Gandhi** clarified that in his line of work he came across hundreds of situations wherein the ascribed author just provided some very meagre descriptive material, which was not enough for a validation, so as long as there was adequate diagnosis or descriptive material by the ascribed author for the validation of the name, then that was what he called validating.

**McNeill** checked that this meant any description that would validate.

**Gandhi** agreed.

**Applequist** felt that if something of this sort was wanted, Prop. F was superior because it also covered a hypothetical case in which the publishing author ascribed part of the description to the same author as the name and part of the description to a third party, a case that Prop. I did not cover.

**Perry** had a problem with Prop. I, where in the first line it said a validating description was of the author who provided the name and a description and in the bottom line just “description or diagnosis” was referred to, not saying whether it was validating or not.

**Greuter** highlighted that he had just a moment ago given an example in the other context, which the Rapporteur had wrongly thought was inappropriate. He asked the proposer or the Rapporteur if either of those versions, and if so which, would cover his concern. It was not infrequent that a name was validated from a herbarium label and ascribed to the author of that herbarium label in a perfectly good formal publication by another author or later author who gave a description, diagnosis and then cited the type with the full text of the herbarium label on which was “flowers yellow”. In that case, “flowers yellow” was from the author to whom the description was ascribed. His feeling was that that both these versions would then make the author of the label the author of the name. He added that if he was correct, he would vote against both.

**McNeill** thought he was correct but that there were situations that the *Code* could not possibly cover and that was a rather extreme one.

**Perry** thought Greuter was right but the idea was so that it would be possible to be able to assign the author of the name before lectotypification or the choice of validating description; otherwise, nobody could ascribe the authorship until somebody had lectotypified the name.

**Gandhi** had mentioned at the very beginning examples with John Torrey and Asa Gray’s *Flora of North America*, wherein they had incorporated several names associated with validating descriptions from Nuttall but they had also added their own remarks. In such cases he reported that when this was examined nearly four years ago involving McNeill, Greuter and Nicolson, among others, the decision was taken, as far as typification was concerned, to look at the entire protologues. He concluded that the question of authorship and the typification would be kept separated.

**Knapp** moved to a simple majority vote on a choice between Prop. F and Prop. I. By simple majority Prop. I was chosen as the proposal to discuss as an amendment to the *Code*.

**Gandhi** called for a card vote on Prop. I.

**Knapp** [later] reported the results of the card vote on Prop. I: for was 209 votes, against was 230 votes. As this did not reach the supermajority, the proposal failed.

**Prop. I** was **rejected** on a card vote (209: 230; 47.6%).

**Prop. J** (42: 28: 34: 0) was ruled referred to the **Editorial Committee**.

**Prop. K** (52: 27: 28: 0).

**Reveal** noted that the first line had nomen novum, which should be replaced with “replacement name”. He explained that the case in the proposal did not occur very often, but when it did, it made it rather challenging. He suggested for those of the audience who were bibliophiles in getting authorships right that the proposal would help in making a fairly simple decision, but it did require some effort on the part of the subsequent individual working in the indexing centres to actually look at such things as introductions and acknowledgements and so forth. It was just a convenient way of handling a relatively rare situation.

**Prop. K** was **accepted**.

[*Here the record reverts to the normal sequence of events.*]

**Prop. L** (19: 79: 6: 0) was ruled **rejected**.

**Prop. M** (9: 66: 27: 0).

**McNeill** introduced Art. 46 Prop. M, which noted the difficulty of determining authorship of the name of a new taxon that was validated solely by an illustration with analysis and there was no evident authorship to it. He felt that there were difficulties in implementation of it and the Rapporteurs suggested that a more satisfactory solution would be to rule that a name so validated must always be attributed to the author of the publication that was defined in Art. 46.5.

**Van Rijckevorsel** felt that the main issue was that at the moment nothing was provided for. The illustration with analysis was there by exception and all the provisions on authorship did not take it into account. As a practical matter, the most clear case was when you had on the illustration a name that was ascribed to an author. Then it was obvious to everybody who was the author. He added that, as the Rapporteurs noted, that was not necessarily the case. In his view, that was a second case, when sometimes the illustration was included in a publication, in which case the author of the publication could be the author of the name. He also referred to the case in which there was an illustration with a name on it and no author, in which case the name may not be ascribed to an author and there was no clear author of the publication and then you were really in trouble. He had drafted an addition in case the name was not ascribed on the illustration and that should cover most cases. The cases that it did not cover were going to be very, very difficult and he had no idea how to deal with them.

**McNeill** pointed out that it was not possible to cover all situations in the *Code*, but he thought it seemed a useful addition to what was in the original proposal.

**Sennikov** thought that the proposal looked a little bit illogical because, in this case, the illustration with analysis served in place of a description, and it could be ruled in the same way as a description and it was the same matter. The ascription of the name that was published was not necessarily connected with the authorship of the description, and the authorship of the illustration, if taken literally, may not be connected with the ascription. The author to whom the new name was ascribed may have not seen that illustration and may have done nothing but suggest the name for the taxon. So if common logic was applied in the same manner, this rule would be contralogical [sic].

**Gereau**, while preparing for the mail vote for this case, had gone through the BHL [Biodiversity Heritage Library] for the last six cases of species validated solely by an illustration with analysis to see what the results would be, and in five out of those six cases there was no author on the fig and there was an author ascription in the associated text. Not descriptive text but where the name was mentioned in the text. In the sixth of those cases the authorship was slightly different, and that listed in the text was clearly the author of the work. So he thought the proposal was not helpful and possibly confusing.

**McNeill** noted that it was quite strongly defeated, but not by 75%, in the mail vote.

**Prop. M** was **rejected**.

**Prop. N** (86: 7: 13: 0) was **accepted**.

**Prop. O** (69: 5: 30: 0).

**McNeill** introduced Prop. O, which he noted, rather like Prop. N, was to make explicit what was currently implicit.

**Sennikov** had a question for the proposer: did this case cover the situations of citations, when the descriptive matter was quoted from the previous publication?

**McNeill** answered that what he was describing was not covered under the proposal, but there were proposals that addressed the matter and they were the ones that had just been passed over.

**Sennikov** wondered why we should believe that the person who referred to the previous description could own the authorship of that previously published description, even if it was quoted. He could not see the need for the Note, because it was just a reference to a previous publication. He likened it to his making a reference to Linnaeus but not owning the authorship of the Linnaean text to which he had referred.

**McNeill** thought the point was that it paralleled the proposal immediately before, which had been accepted. Article 46 was quite precise as to what ascription was but it was perhaps too precise in places, because people did not normally put their name at the end of a description, except when they were citing somebody else’s description. It was assumed from the fact that they were the author of the work. Proposal O was to deal with this situation with regard to the normal situation where a name was validly published on the basis of a concurrent description or diagnosis. Proposal O was dealing with the same situation where the description or diagnosis was in an earlier work.

**Turland** agreed and added that if the description or diagnosis was from an earlier work but it was quoted, reproduced verbatim in the current work, then this would not apply to that situation.

**McNeill** stated that it would fall under Prop. N.

**Greuter** would be happy to support the proposal if he understood it, but his English was too poor to understand it fully as it was worded. He suggested that it be referred to the Editorial Committee to try to put it into an English understandable for the nonEnglish speaking. [That was acceptable to the proposers.]

**Glen**, as an Englishspeaking person, was also lost, and he asked the proposer to work through an example.

**Turland** noted that there were actually some current Examples in the *Code* that showed the issue and he thought Art. 46 Ex. 13 would be an example. So when publishing *Elaeocarpaceae*, Candolle wrote “*Elaeocarpeae*. Juss.”, so he ascribed the name to Jussieu, but the validating description came from that earlier publication of Jussieu, so therefore he also ascribed the validating description to Jussieu.

**Challis** believed that the intention of this proposal was to deal with post-1958 names where there was a defective type citation and someone subsequently published the name of the new taxon correcting the type and they referred to or quoted the earlier author’s description.

**Turland** answered that that was not necessarily so, it would apply there but that was not the intention.

**Challis** continued that it would apply, and there were several examples where this would be very helpful to clear up the authorship. She was speaking for the proposal.

**Prop. O** was **accepted**.

[*The following discussion, pertaining to a new proposal by Reveal concerning Art. 46, took place during the Tenth Session on Friday afternoon.*]

**Reveal’s proposal**

**Reveal** referred to a proposal from the floor submitted on Wednesday.

**McNeill** stated that he had no other proposals in front of him except one, which he was just about to come to. He pointedly noted that the Section had been instructed twice to make sure he had all new proposals. He was still willing to take it, but he had no note of any more proposals.

**Reveal** persevered that McNeill had it in his own possession.

**McNeill** responded “Sorry?”

**Reveal** repeated that he had given it to McNeill; he had it in his own possession.

**McNeill** conceded that had occurred earlier on by e-mail, yes, but he did not have it in front of him now. [Laughter.]

**Knapp** pointed out that this was the Nomenclature Section and when the Rapporteur said he wanted it on a piece of paper in front of him, we were all nomenclaturalists, and that was just what it meant on the tin. She moved to the proposal from Reveal and Gandhi to add a Note to Art. 46 with an Example. [The **proposal** was **seconded** and **supported** by three others.]

**Reveal** briefly noted that it had been brought to their attention by Greuter and discussed by McNeill, Wiersema and others over a couple of weeks in early summer, late spring, and this was a result of that discussion.

**Gereau** requested a point of information from the proposers as he did not understand the impact of this. This was authorship of *Cephalotaxus latifolia* W. C. Cheng & L. K. Fu ex L. K. Fu & R. R. Mill, as originally cited. It was not clear whether this was as it was originally cited or as it appeared in *Novon*.

**Gandhi** referred to the usage of “ex” authorship citation. Normally the manuscript [i.e. original] author failed to provide something like the description or the relevant material needed for validation. There were a few cases wherein the originally ascribed author did provide a Latin description but for some technical reason might have not published the name validly. Then in the next step the name became validated. The validating author may be a common factor to both, before the “ex” and after the “ex”. Normally in such cases the authorship was accepted as originally ascribed, but in this particular case, where L. K. Fu was a common factor both before and after [the “ex”], and they did not ascribe the authorship to the original author, they wanted the authorship for themselves. So that was explicitly made clear by citing their own name. He wondered what to do in such cases. Should the “ex” authorship be accepted or omitted? He felt that the cited Example would be useful.

**Greuter** thought that the question was that, as the *Code* said, ascription, as defined, meant that the name was ascribed or attributed by the author to someone else. If someone else published a name such as “X ex Y” and Y was himself or “X ex myself”, he ascribed the name to himself and not to the person preceding “ex”, so it was not ascription to Mr X or Y. He moved an amendment, possibly friendly, to let the Note stay as it was, Note 4, referred to here, but turn it into an Article. Article 46 Note 4 was in the *Code* and had been followed and was useful, but it was not actually covered by the Articles, so it was not a Note. That was the reason for turning it into an Article, so that it could continue to be used without problems, and he thought it would solve the problem that had been addressed by the present proposal, so it might be accepted as a friendly amendment by the proposers. [It was **accepted** as a **friendly amendment**.]

**Knapp** summarized that the proposal, as amended, was to change Art. 46 Note 4 to an Article and keep the Example.

**Nic Lughadha** was a native English speaker but wanted to ask about the significance of the “withstanding” in this case, as it was not clear to her.

**Knapp** thought that that text was no longer relevant.

**Dressler** noted that there was a “may” in the Note and wondered if that was an editorial matter to change the wording.

**McNeill** confirmed that if it was changed to an Article it would have to have the appropriate structure of the verb.

**Redhead** was a little confused as he thought that the latter half of Art. 46.4 already allowed what he thought was trying to be added into the *Code*, so it looked redundant. He acknowledged that he may be misinterpreting it but thought that “However, in both cases authorship as ascribed, followed by ‘ex’, may be inserted before the name(s) of the publishing authors” was exactly what was being talked about.

**Gandhi** explained that since one of the authors before and after “ex” was a common factor, some people may want to omit everything after “ex” and just retain only what came before the “ex”, in order to avoid that mistake. L. K. Fu, before the “ex” as well as after the “ex”, was a common factor.

**Stevens** called the question.

**Knapp** felt that the debate was getting into a discussion about the finer points of nomenclature, and actually this should have been in proposals and people could have discussed it for a longer time than here on the floor. [There was a sufficient majority in favour of voting.]

She counted 37 “no” and 59 “yes”, which was 61%, so the proposal was accepted and Note 4 would be turned into an Article.

**Reveal’s proposal** was **accepted**.

[*Here the record reverts to the normal sequence of events.*]

## Eighth session

Thursday, 20th July, 2011, 13:30–17:30

### Recommendation 46D

[*The following debate, pertaining to Rec. 46D, took place during the Ninth Session on Friday morning.*]

**Prop. A** (71: 24: 14: 0).

**McNeill** introduced Rec. 46D Prop. A by two authors actually called Nobis. The Recommendation: “Authors should cite themselves by name after each new name they publish rather than refer to themselves by expressions such as ‘nobis’ or ‘mihi’. These expressions should be used only if they are identical with the name of the author in which case they should be written with an initial capital, i.e., ‘Nobis’ or ‘Mihi’, and, where necessary…”. The covering note suggested that the *Code* was discriminating against authors by the name of Nobis and there were indeed two Polish authors by this name who were authors of this proposal.

**Knapp** concluded that this “just goes to show nomenclature can be fun”.

**McNeill** thought that editorially it might be wiser to confine it to the first portion rather than the second one.

**Karen Wilson** commented that in this age Latin was becoming so much less common that using “nobis” or “mihi” in these circumstances should be discouraged, just for clarity. She added that too many people would not know what was meant.

**McNeill** interpreted that as supporting the first part of the Recommendation.

**Karen Wilson** confirmed that.

**Greuter** suggested that Prop. B be referred to the Editorial Committee to try to cover its intent by finding a simpler wording.

**Demoulin** wondered whether it was necessary to have this proposal at all for very rare cases. He was against it but thought it was better for the Section to decide whether the Recommendation was desirable or not, and if so it could be arranged by the Editorial Committee. He felt that if it was referred to the Editorial Committee and the Editorial Committee decided after all we can do without it, there would be people who would later say that the Editorial Committee had been doing too much.

**Marhold** would vote against it as he felt it was common understanding that if somebody had the name Nobis, nobody would treat him as “nobis” in that sense.

**Barkworth** understood that one of the criticisms when a paper was rejected was that the author was using “nobis” after the name and he should not do so because the *Code* said he should not, so it was the reviewers that were a problem.

**Knapp** added that it was people who were ignorant.

**McNeill** emphasized that he may have misled the Section earlier as the only part that was new was the second paragraph. The first sentence was in the *Code* already.

**Knapp** noted that her counting skills were being tested to the limit and reported that there were 52 in favour and 49 against, which did not reach the supermajority to amend the *Code* so the proposal failed.

**Prop. A** was **rejected**.

### Article 48

[*The following debate, pertaining to Art. 48, took place during the Ninth Session on Friday morning.*]

**Prop. A** (83: 0: 26: 1) was **accepted**.

**Prop. B** (66: 20: 18: 0).

**McNeill** introduced Art. 48 Prop. B, which was seeking to clarify the circumstances under which the adoption of an existing name resulted in the publication of a later homonym utilizing some of the criteria from Art. 52. He noted that the phrase “original type” appeared in the Article at the moment, which was a rather unusual circumstance for older names, other than generic names, and not a term that was well defined in the *Code*. The proposal had good support in the mail vote: 66 in favour, 20 against, 18 to the Editorial Committee.

**Greuter** had a difficulty with clause (c): “a previously conserved type under Art. 14.9”. “Previously conserved” meant that it would have dates for conservation, but he argued that it was not possible to know exactly when a name was conserved—was it by the final action of the Congress, a report etc.? Either way he felt that (c) was irrelevant and could go. He suggested a friendly amendment to delete “previously”, then he would be prepared to leave (c) in but otherwise he would propose an amendment to delete “(c) the previously conserved type under Art. 14.9”. [This was not considered a **friendly amendment**.]

[The **amendment** was **rejected**.]

**Prop. B** was **accepted**.

**Prop. C** (2: 14: 89: 0) was ruled referred to the **Editorial Committee**.

### Article 49

[*The following debate, pertaining to Art. 49, took place during the Ninth Session on Friday morning.*]

**Prop. A** (7: 72: 27: 1).

**McNeill** introduced Art. 49 Prop. A, which was concerned less with author citation and more with illustrating that the basionym or replaced synonym must legally be a definitely included element in the new combination etc. He added that the Rapporteurs felt that it was not in the appropriate place and that it applied more generally. There was a strong negative vote in the mail vote: 7 in favour, 72 against, not quite 75% though.

**Gandhi** had come across several situations like this, wherein the publishing author with this new name cited an earlier name with the identical epithet with the expression of doubt, so it became a problem whether to treat the newly published name as a new combination or as [the name of] a new species.

Whenever such difficult situations arose he always had discussions with his nomenclature colleagues and drew support from Art. 52, wherein whenever an earlier synonym was cited with an expression of doubt, it did not cause superfluity to the newly published name. He wanted to know why, if that was the criterion, was this not applied to the situation given in the proposal? That is, the cited earlier name with an expression of doubt should not be treated as the basionym. In other words, the newly published name should be treated as the name of a new taxon. In general his colleagues agreed with him because the epithet was identical, it should not be mistaken for the basionym.

Despite a lot of negative votes in the mail vote he was positive that a decision had to be made to treat the newly published name as a new combination or treat that name as the name of a new taxon. He was quite agreeable one way or another.

**McNeill** referred to the Rapporteurs comments that if the *Code* required definite inclusion then an expression of doubt ruled that out. Generally speaking, if the supposed basionym had an expression of doubt, it was not definite inclusion and therefore ruled out under other Articles. Their concern was it did not belong in Art. 49 and might fit better in 33.

**Gandhi** was agreeable to that; he felt that as long as there was some Example in the *Code* addressing the situation it would be very useful.

**Alvarado** had a question for the proposer: did he mean, for example, that if someone described a new species but was in doubt of the genus, then the species was valid but the genus was not?

**Gandhi** responded that that situation was already addressed in the *Code*. Such examples were validly published. It was because the expression was of taxonomic doubt, but not nomenclatural doubt, that the names were valid.

**Prop. A** was **rejected**.

[*The following general discussion about the Acacia issue took place during the First Session on Monday morning.*]

### *Acacia* general discussion

**McNeill** explained that the proposal by Brummitt, Art. 51 Prop. A, received substantially more than 75% “no” votes [83%], indicating that it would not be discussed. He suggested that the published proposal had a right to be talked about first, although the half-hour discussion was intended to be wideranging and informal. He asked if anyone wished to bring the proposal forward for discussion, requiring five people to propose it. He went on to explain the content of the Brummitt proposal. It suggested using the name *Acacia* independently of the taxonomy, so the name *Acacia* could still be used regardless of whether it was considered to belong to the group that is called *Acacia* now, or the group that would be *Vachellia* or *Senegalia*. The generic name would not indicate the relationship.

**Knapp** displayed the proposal on the overhead screen. She also mentioned a compromise proposal, which had been published in *Taxon* but had not been part of the formal synopsis of published proposals [Turland proposal.]

**Barrie** thought it would be easier to try to get the first proposal reintroduced before moving onto the next, suggesting the Section could vote on whether it should be discussed or not.

**McNeill** clarified that nobody had proposed the Brummitt proposal for discussion.

**Barrie** proposed discussion of the proposal by Brummitt, Art. 51 Prop. A, and asked if there were four people to support it. [There were.]

**Herendeen** had understood that the half an hour that was set aside was an informational half hour to get people up to speed and it seemed to him that information should be presented so that people understood these proposals, regardless of how many mail votes they received. He asked if someone could present a quick, one- or two-minute synopsis summarizing the *Acacia* options.

**Knapp** clarified that if the Section wanted to discuss a proposal that had been defeated in the mail vote, procedure required that it be proposed and seconded by four people, which had been done, so it was possible to proceed to discussing the proposal.

**Van Rijckevorsel** found the Brummitt proposal very interesting, but he was not sure if he was in favour of it. He thought the wording was quite inappropriate, so wanted to submit an alternative wording if it was going to be discussed. He disliked the Turland proposal, which would mean that nobody could use the name *Acacia*, and proposed a third option: conserving the name *Vachellia* with a conserved spelling. If there was deep feeling that a different name would be more acceptable, then the Section could conserve the spelling for *Vachellia*, and the working option he suggested was *Afroacacia*. He suggested this would be the most efficient way with the minimum disruption of names and the minimum disruption of the *Code*.

**McNeill** summarized Van Rijckevorsel’s proposal. The *Vienna Code* had been accepted—*Acacia* was the conserved name with *Acacia penninervis* as type, the suggestion was a conservation proposal, which would have to go through the necessary steps but could be expedited, by which the name *Vachellia* would be treated as conserved, with a conserved spelling, which would be *Afroacacia*. That would give the name priority from the date of publication of *Vachellia*, which may or may not be desirable. It would have to receive the broad support of the Section, and then the proper procedures for a conservation would have to be adopted.

**Van Rijckevorsel** accepted that as an almost accurate summary. He elaborated that he was proposing to include the rule in the *Code*, that it was to be treated as conserved, which would mean that the Section could accept it immediately, and there would be no need for a conservation proposal, but he also clarified that he was not seriously proposing this, just suggesting it would be the minimum disruptive proposal.

**Gereau** claimed that both the proposal on the board and Van Rijckevorsel’s amendment, and all other proposals calling for exceptional nomenclatural actions with no precedent and unpredictable consequences, were really completely unnecessary. He pointed out that the uses of the name had been established by procedures that already exist. He encouraged voting “no” on all compromise proposals and living with the results of the votes in Vienna and Melbourne.

**Lewis** noted that Van Rijckevorsel’s suggestion of *Afroacacia* was looked at quite seriously as a possible compromise position, but reminded the audience that *Vachellia* was a pan-tropical, not an African-specific genus. The reason *Afroacacia* was not supported was that it would be egocentric to pick on a name that was specific to one continent, whereas the plant actually spanned several. He addressed Gereau’s proposal to just move on, because a vote had been taken and *Acacia penninervis* had been supported, and suggested that, while that might be a practical and pragmatic way forward, he feared the end result would be six more years of disagreement about *Acacia*. He considered it absolutely fundamental for the good of the nomenclatural and botanical community to arrive at a solution that was acceptable to all, and not acceptable to just some of the people in the audience.

**Boyne** was at one of these Sections for the first time and was not entirely familiar with the rules. He prefaced his question with the caveat that it might seem naïve, but he wondered, was it possible to have two versions of *Acacia*? *Acacia* sensu stricto for the most narrowly defined monophyletic *Acacia* and *Acacia* sensu lato to apply to all genera once classified as *Acacia*, whether monophyletic or not.

**Schrire** was worried about the Brummitt proposal because he claimed that recognizing *Acacia* as one taxon was becoming less and less tenable as evidence had shown that *Acacia* was polyphyletic and comprised widely separate evolutionary lineages. He found it untenable to recognize all of these by the name *Acacia* or *Acacia* sensu lato, as suggested. He pointed out that implicit in the Brummitt proposal was that all of the combinations in *Senegalia*, *Vachellia*, and *Racosperma* would be considered illegitimate and therefore unavailable for future use. He found this a dangerous, bad principle and retrograde step.

**Maslin** thought it was a bad idea to accept either of the proposals [Brummitt’s or Turland’s] because two acceptable names were already available: *Acacia* and *Vachellia*. He noted that combinations had already been made for around half the species in *Vachellia*, and the name had already been taken up to some extent in scientific and other literature and web resources. He offered to enumerate these if desired. [It was not desired.] He suggested the Section let the due processes of the nomenclatural system take their course.

**Levin** wanted to know if enough people supported discussion of the Turland *Acacia* compromise, as for the Brummitt proposal. He also requested that Turland explain his proposal.

**Turland** asked for a copy of the proposal to be put up on the screen. [This was done.] He introduced the concept and why he had proposed it, prefacing his comments by saying that he was quite neutral on the issue and personally had no preference for where the type of *Acacia* should be. He neither supported nor opposed the proposal, it was a suggestion for the Nomenclature Section to consider. The rationale behind it was that if a vote occurred at the Nomenclature Section in Melbourne, which resulted in one side of the *Acacia* dispute winning and one side losing, then it could result in negative feelings, which could persist for the next six years. So the idea of the compromise was so both sides could leave Melbourne with something acceptable, rather than one side going away defeated and the other side the winners. Basically, the mechanism suggested was to conserve three names: *Acacia*; and then the name *Austroacacia*, used in the sense of *Acacia* under the *Vienna Code*; and *Protoacacia*, used in the sense of *Vachellia* under the *Vienna Code*. These names would be deemed to have been simultaneously published, so in effect, *Austroacacia* and *Protoacacia* would be conserved orthographies of *Acacia*. The three conserved names would need to go through the usual process of conservation of names, so they would need to be agreed by the Committees. If the genus *Acacia* were recognized or circumscribed in the broadest sense, which many people agreed was taxonomically untenable, *Acacia* would be the correct name. However, if the segregate genera were recognized, then *Acacia* would not be used for any of them, and *Austroacacia* would be used in the sense of *Racosperma* or *Acacia* and *Protoacacia* in the sense of *Vachellia*. The existing combinations in *Acacia* would provide the authors and places of publication, so it would not necessitate 1400 new combinations, but it would obviously necessitate name changes.

**Knapp** clarified that if this was to be discussed it needed to be proposed and have four seconders. [The **motion** was **seconded** and supported by three others.]

**Demoulin** gave the perspective of a teacher of general botany to pharmacists, geographers and biologists. With limited time to devote to any important plant genus for something like *Acacia* he would teach pharmacists and geographers that it was a big genus, very important in ecology of tropical and subtropical areas with a dry season, and it produced Arabic gum, note that there were a lot of species in Australia, it included two invasive species in the Mediterranean region, and that was all. He voted for the Brummitt compromise, because it would allow him to retain that kind of statement, but he was also ready to vote for the present compromise, because it would retain the word *Acacia* in some combination that would be easily understood. He added that pharmacists used books that had been using *Acacia* for two centuries, and geographical description of vegetation, even tourist guide books. If the Brummitt proposal was considered rejected, he was in favour of the Turland compromise.

**Alvarado** felt that science should not be hindered by traditions. He thought that the Section should follow the usual procedure that was followed with other plants, that the clade in which the type of *Acacia* was found should be called *Acacia* and other groups should be called according to their oldest described species—in their own genus.

**Sebsebe Demissew** noted if the IBC meeting had been somewhere in Africa, the vote could have been different, but he respected the decision of the people here, even though he expected that there were many members of the Australian community voting for the motion. He reminded the Section that *Acacia* was not just a matter of molecular studies and clades, it had ecological, historical, cultural etc. implications. He stated that you cannot dissociate Africa from *Acacia*, but having now agreed to follow the decision of the Committee, he found the Turland proposal to be a better option. Not the best option, but a better option that would keep *Acacia* for Africa as *Protoacacia*. He made the comparison that people in Africa would consider it better to have a little bit of yourself in a photograph than none at all. He considered having *Austroacacia* and *Protoacacia* would be a better option for the future, not just in terms of legume systematics, but thinking of the global community, tourism etc.

**Karen Wilson** made the parallel with a similar problem in Australia in the past when *Eucalyptus* was split up. *Corymbia* created a furore amongst foresters and native plant enthusiasts. What people eventually had come to accept was that you could still use the name eucalypt as a common name, and she suggested the same could apply to *Acacia*. She felt that Australians would use acacia as a common name, and people in Africa would continue to do the same. She pointed out that the general public could continue to use acacia as a common name and the botanical community would know which was the appropriate name to use in scientific terminology.

**Rico** wanted to point out that while many people were focusing on how important *Acacia* was in Africa it was also important in the neotropics. She referred to Maslin, who said that some people had already taken up *Senegalia* and perhaps other names in papers, but asserted that in real life ecologists and others were very confused. She suggested that some of the changes to *Senegalia* and *Vachellia* had nomenclatural inaccuracies and the same people who made *Vachellia* and *Senegalia* combinations had started to submit proposals to conserve some *Vachellia* or *Senegalia* under *Acacia*, due to their importance. Her main point was that the *Acacia* problem extended further than Australia and Africa, into the Americas, Asia and to India, giving examples of *Acacia nilotica* in India and *Acacia farnesiana*, *Acacia tortuosa* and *Acacia aroma* in the Americas.

**West** brought the discussion back to the Turland compromise proposal. Like Lewis she was very keen to see some sort of compromise come out of the debate, and requested clarification from the proposer or Rapporteur-général as she suggested that the proposal may set a precedent for the Section to be able to change names of any group of plants.

**Turland** noted that because the issue involved conservation of names, it was possible to argue that if a Section did not like the name of a particular plant and there were compelling reasons for nomenclatural stability, then it was already possible to change the name under the current rules. He suggested that what was meant was the desire to change the name of a plant for some reason other than nomenclatural stability, and he agreed in theory that the answer was, yes, it could set a precedent. He added that he hoped that *Acacia* was a unique case and that he personally thought it unlikely that something as controversial as this would again reach the stage of having been conserved and ratified by a Congress, and then questioned afterwards.

**Mabberley** was not in favour of the Brummitt proposal and had some sympathy with the Turland proposal, but thinking about the true users of names, he believed that some *Acacia* was better than no *Acacia*. He suggested an amendment to the notion of *Protoacacia* if the Turland proposal was to be seriously considered. The connotations of evolutionary succession were undesirable and he suggested *Millero*-, after Miller. He could see where *Austro*-, came from, although he noted that the distribution of this group did go as far as Hawai’i and various other places.

**Gandhi** had been contacted by the Botanical Survey of India, who asked him to convey their interest about retaining the name *Acacia* for India. He also agreed with Karen Wilson that the term acacia could be used as a common name for any group, regardless of what scientific name was chosen, and gave the example that in the southern U.S. the term mimosa was used for *Albizia*, which was far more incorrect than using acacia as a common name.

**Smith** thought that the Section recognized and accepted the outcome of the first card vote on Monday. Unfortunately, as had already been mentioned, he felt that this would do nothing to get rid of the controversy, and the next six years, 12 years, 18 years, however long, could be spent, at least by a component of the botanical society, trying to change things around. He suggested this was an opportunity for compromise. He was not in support of any of the compromise proposals as the full implication of adopting any of them was impossible to foresee.

**Redhead** pointed out that if the Turland proposal were to be voted on, amendments would have to be made to Art. 11.1, which talked about correct names.

**Orchard** wanted to know whether the third proposal from Van Rijckevorsel was on the table for discussion or not.

**Unknown speaker** asked for a point of information as to when the thirty minutes might finish.

**Knapp** confirmed the discussion would be closed in about five minutes.

**Van Rijckevorsel** formally proposed introducing a new rule, which would treat *Vachellia* as conserved, with a conserved spelling to be determined, with *Afroacacia* as a working proposal. Other options would be *Anacacia* or *Abacacia* (something with an ‘A’). [The **motion** was **seconded** and **supported** by three others but seemingly not revisited in the later discussion.]

**Demoulin** pointed out that in Europe, acacia was used as a common name for *Robinia*.

**Lewis** queried whether there was another *Acacia* proposal by Linder and Crisp.

**McNeill** confirmed that there was a proposal to set up a Special Committee to explore the matter including suggestions as to what the Special Committee would consider including the existing proposals and now the others that had come forward. They qualified it that the Committee should include people who were not previously involved, and unbiased, which struck many as difficult to achieve, unless they were not vascular plant taxonomists.

**Lewis** responded that, while his personal view was that it would be not a good idea to drag this further, it struck him that that would be a default position if the Section did not reach a consensus supporting either the Brummitt or the Turland proposals. He queried whether the other option was that it would stay as it is.

**McNeill** envisaged that, having had this discussion, the people most interested in the topic would get together over the following day or two, to see if something could emerge that would be likely to receive significant support. He felt that the value of having had this general discussion was that it got a lot of views on the table, which would make it easier for taking things forward informally, in the hope that something more concrete might be presented at the appropriate time later in the week.

**Knapp** closed the discussion at just over a half hour.

[*Here the record reverts to the normal sequence of events.*]

### Article 51

**Prop. A** (11: 89: 6: 1).

**McNeill** introduced Art. 51 Prop. A as significant primarily because it was the proposal from Brummitt regarding the use of the name *Acacia*. He reported that the proposal had received quite a substantial negative vote in the mail vote: 11 in favour, 89 against, six to the Editorial Committee, and one to a Special Committee, with an 83% negative vote. [The **motion** to discuss the proposal defeated in the mail vote was **seconded** and **supported** by four others.]

**Davidse** was strongly against the proposal as well as the other so-called compromise proposals. To him, it introduced an entirely new concept, sort of through a back door, which he thought of as hyper-conservation. He thought, as had been said during the earlier discussion period, it was a very bad precedent to vote for.

**Nicholas** was not supporting the proposal, but wanted to mention that he had seen publications already out there that used the notation, in particular *Lessertia*, with *Lessertia* and then in brackets after that, *Sutherlandia*, so it was being used by other people out there.

**Gandhi** was also not supporting the proposal. His department was concerned that the citation of *Acacia* followed by *Senegalia* within parentheses or *Racosperma* in parentheses might cause confusion as to whether such parenthetically cited names represented subdivisions, such as subgenera.

**Hawksworth** also thought it was a very bad precedent putting something like this in the *Code* because he could imagine all sorts of things in the future going into that detail. He maintained that the *Code* should be about the principles and not deal with special cases.

**Van Rijckevorsel** thought it was a very brave and courageous and bold proposal, which unfortunately was, well…

**Knapp** asked “A dangling sentence, Paul?”

**Anderson** recalled that when this was discussed at Vienna, there was…

**McNeill** interrupted that this was not discussed at Vienna.

**Anderson** continued, what was discussed was the *Acacia*…

**Knapp** ruled the comment out of order and stated that the Section were not discussing what happened in Vienna, at all, in this session. She added that it was a Chair’s rule.

**Anderson** was just going to say that there was political pressure put on…

**Knapp** cut him off and repeated that there would be absolutely no discussion of what happened in Vienna, adding that we were here in Melbourne now, and were moving forward.

**Nic Lughadha** called the question.

[There was a sufficient majority in favour of voting.]

**Prop. A** was **rejected**.

**Turland’s proposal**

**McNeill** introduced another proposal relating to the topic of *Acacia* that was published in *Taxon*.

**Schrire** proposed that the Turland proposal be discussed. [The **proposal** was **seconded** and **supported** by three others.]

**Potgieter** suggested some of the wording be changed, hopefully as a friendly amendment. She proposed that the name *Protoacacia* rather be *Acanthacacia*, as it did not have a geographical bias and was actually quite descriptive of the thorny nature. [This was **accepted** as a **friendly amendment**.]

**Redhead** thought that it was what he had commented on earlier, that it looks like publication of alternative names, so Art. 11.1 would have to be modified to make exceptions, because Art. 11.1 said that there can be only one correct name.

**Turland** did look at that when it was mentioned on Monday. Each family or taxon of lower rank with a particular circumscription, position and rank could bear only one correct name. He wondered if there was the suggestion that there was more than one correct name for a particular taxon here.

**Knapp** suggested that, although he was not the person proposing the motion from the floor, he was the person who wrote the proposal, and it would be good if he would explain it very succinctly so that people who were not involved in having read it before understood what had been proposed.

**Turland** explained that the mechanism was to deem that when *Acacia* Miller (1754) was published, there were actually three names simultaneously published: *Acacia* Miller (1754); *Protoacacia*…

**Knapp** added that this was now amended to *Acanthacacia*.

**Turland** …now amended to *Acanthacacia*; and also *Austroacacia* Miller. So, this meant that also any combination published under *Acacia*, with this cut-off date, 1 January 2011, was treated as having been simultaneously published under all three generic names. So not only the generic names were simultaneously published, but any combination published under *Acacia* before that date was also simultaneously published with the corresponding combination under *Protoacacia* or *Austroacacia*.

Then there was also a ruling on priority, where *Austroacacia* and *Protoacacia* both had priority over *Acacia*, except when a genus was circumscribed to include the types of all three names. In other words, when *Acacia* was circumscribed in the broadest sense, in that case, *Acacia* had priority over the other two. The function of this was that the name *Acacia* would not be able to be used at all, unless a genus was circumscribed to include the taxon *Acacia* in its broadest sense, which was arguably taxonomically untenable.

Then the other rules were to take care of various exceptions, for example with alternative names, and the other thing that would be necessary would be to have the names conserved, which could not be done in the Section; that would need to go through the Committee for Vascular Plants and be conserved with conserved spellings and types.

**Redhead** guessed that, the way it was outlined, any particular classification was either accepting them all together in one, in which case it was called *Acacia*, and there was one correct name under that classification, or, if it was split, then there was only one correct name under the split, which would either be *Austroacacia* or *Protoacacia* or whatever it was changed to. Then he had noticed that the type was simultaneously publication [sic] via conservation of *Austroacacia penninervis* versus *Acacia penninervis* with the same authority, which he thought seemed … imaginative. [Laughter.]

He was just worried it was going to run into conflict with Art. 11.1. It seemed to him, from what had been explained, that perhaps the bullet had been dodged on that one, because the question came up: what was the correct name? He supposed under any particular classification, under the scenario outlined, you would be led to one correct name. So he guessed that alleviated his concerns and had just wanted to walk himself through it.

**Malécot** was wondering why there was a date there with 1 January 2011 and what would happen if someone wanted to describe a new *Acacia* in 2012 from, say, Brazil, *Acacia brasiliana*.

**McNeill** explained that it was just an *Acacia*.

**Malécot** clarified that someone would have to make a new combination into *Acanthacacia*.

**Turland** agreed that that was correct.

**Lewis** firstly supported the proposal and secondly he thanked Turland for going to so much effort and trouble in trying to seek some sort of compromise on the *Acacia* issue. He acknowledged that there were some technical difficulties, but considered it important for the future credibility of the nomenclatural process that a wellconsidered attempt was made to give the wide tropical botany community some sort of solution on this issue. There were many who felt under-represented at nomenclatural sessions and he felt that the proposal absolutely spoke for them. Currently it was the only compromise proposal left on the table. If the Turland proposal were to be voted down, then in his personal view the *Acacia* saga had not been resolved.

**Applequist** was concerned about the statement that to be conserved the names had to be run through the Nomenclature Committee for Vascular Plants in a separate step. It seems to her that the Section should be able, by democratic vote, to change the *Code* by putting names into the Appendices, just like any other change. But if this had to be run through the Committee, she felt it important to ask what happened if the Committee did not approve the conservation of those names and the rest of the material was already inserted in the *Code*. She saw a serious risk there.

**McNeill** also thought it was a serious risk. He added that Art. 14 was very clear as to how names should be conserved and he thought it would be very unusual to amend Art. 14 temporarily in some way and certainly would not want a situation to arise in which the due process was not put forward for virtually every name. The purpose of the conservation proposal was of course stability of names and that would be the criterion that would be being looked at.

**Wiersema** agreed that it would have to go through the relevant Committees in order for the conservation to be active, which made it all provisional. He asked how to put in a rule that depended on these other elements in order to enter the *Code*. Did that have to be accounted for somehow in the wording of the rule?

**McNeill** thought that the rule could enter as it stood, because all it would mean was, until and if the conservation was completed, the names could not be used in that sense. He acknowledged that that would be difficult because they would all be typified under the same name, *Acacia penninervis*.

**Wiersema** added that the other names did not exist and then wondered if they would exist simply because they were in the *Code*.

**McNeill** explained that if the proposal was passed, then they would exist, but until they were conserved with different types they would be inoperable. He added that it was not illogical to include it, it would be just provisional and if they were not conserved in that manner then presumably it would be done at a later Congress. He noted that there had been provisional provisions in the *Code* before, to be implemented in a further Congress, and then it turned out they were not.

**Wiersema** believed that those were stated as such when it was the case.

**Nicholas** philosophized that in science, if there were extraordinary conclusions, extraordinary proof was required and he thought in cases of extraordinary nomenclatural problems, extraordinary solutions were actually required. He fully supported the proposal.

**Nelson** understood that the proposal would perhaps create some nomenclatural problems, perhaps not; however, he wondered what nomenclatural problem the proposal was designed to solve, because it was not at all clear to him that there was one.

**Sebsebe Demissew** thought that everyone knew what had happened in the last five or six years, and was not going to go through the details.

**Knapp** thanked him for that.

**Sebsebe Demissew** continued that there had to be a solution as it was undesirable to bicker around for another five years and he thought that Turland’s proposal was the best option.

**Luckow** responded to Nelson that there was a nomenclatural problem that he and possibly others were not aware of, in that as things were there were two parallel nomenclatural systems being applied. She explained that in Africa and many parts of Latin America neither *Senegalia* nor *Vachellia* had been taken up, which meant that *Acacia* was not being used in a consistent fashion by everyone either in the legume community or even in the floristic community. She characterized this as a worst nightmare for what could happen and pleaded for a solution that would lead to a single application of the names.

**Thiele** felt that it might be useful to have an Australian perspective. He acknowledged that he could not speak for all of Australia because Australia had varying views on the proposal and others may wish to speak for it. He was against it and wanted to make the room aware that a number of Australian institutions, in preparing for this Congress, discussed the proposal at great length. They greatly appreciated the work that Turland had put into preparing a compromise and fully supported the desire for an outcome that helped in some of the political issues that had been raised and they could see the desire for compromise.

However, before coming to this meeting several institutions decided that, no matter the outcome, they would not support this compromise because they saw it as solving a problem that was solvable in other ways and solving it using a mechanism that to them compromised the *Code*. They were concerned about the precedent that it may set. He hoped that it was an entirely unique case, but did not think it was possible to say that. In the future, other cases may well arise that were equally controversial, which may cause a desire among some parties for a similar type of solution. In conclusion, they did not support this proposal.

**Reveal** had a question out of ignorance. Neither of the names that were orth. cons. had been published and these were newly proposed names. When would they become effective and would they have to go through the Committee for Vascular Plants to be conserved? Because normally a name was proposed and then, for one reason or another, it was acted upon.

**McNeill** replied that if he had read the proposal correctly, if it were to be passed, the three names would be deemed to have been published in 1754, therefore they did exist. The action of the Committee would be to conserve them, because as they stood they would be sitting there, all published by Miller, but none of them with priority over the other. So, what was more, they would not be distinguishable one from the other. So it was inoperable until such time as the names were conserved, but the answer was they would be deemed to be published by Miller.

**Reveal**, in his newly imagined electronic journal, *Acacia of the World*, wondered if he were to publish, on 2 January 2012, hundreds of new combinations into *Austroacacia* and *Acanthacacia*, what would that do?

[Pause.]

**Knapp** wondered if either of the Rapporteurs wanted to answer that question or if they would prefer to let it hang there. [Laughter.]

**McNeill** replied that they could cause enormous nomenclatural disruptions.

**Breitwieser** asked Thiele to explain what he meant by saying there were other ways of solving the problem.

**McNeill** noted that, if the proposal should fail, then of course it was not a vacuum, *Acacia* had been conserved six years ago with *Acacia penninervis* as type. That was the default if the proposal was rejected.

**Kellermann** was for the compromise, but it seemed to him that the Committee step was a major issue and he wondered how many people of the Committees were present.

**Knapp** pointed out that the Committees were newly elected by the Section at the end of the session, so there currently was no Committee.

**Ladiges** had a problem with the view that the names were deemed to have been named back in 1754 when they were not. She did not claim to be a nomenclature expert, but did not understand that component. It sounded like storytelling to her.

**McNeill** agreed that indeed it was. He elaborated that it was a mechanism to give priority to ensure that a whole lot of new combinations did not have to be published.

**Mabberley** wanted to reiterate the point that he had made on Monday that some *Acacia* was better than no *Acacia*. Users of names for all of these trees and other plants, those users were going to have to change everything and he did not think that was in the spirit of the *Code*.

**Cameron** noted that it seemed to have escaped other people’s propositions that there was one simple resolution that we could go back to, which had the benefit as Mabberley had just pointed out of not losing the name *Acacia* altogether, but in the spirit of the exceptional circumstances did actually give voice to some of the concerns that had been raised by people from other continents. This was simply to, in the spirit of the controversy regarding the validity of the decision made in Vienna, which…

**Knapp** interrupted to reiterate that there was to be no discussion of Vienna.

**Cameron** argued that he was not discussing the issue, whilst of the available options he would favour the Turland proposition, he wanted to point out that were that to fail, another option that should then be considered as an alternative was that we could go back to accepting *Racosperma* for the group that was currently *Austroacacia*. That would retain a proportion of species in that genus.

**Knapp** refocused the debate on the Turland proposal, not alternative proposals to the Turland proposal. While she understood people’s desire to broaden the discussion, she highlighted that the Section had already had a very wide-ranging discussion for about 45 minutes on the first day, at the very end of the day when we weren’t discussing any proposals in particular, so as Chair she ruled that we were discussing the Turland proposal and not alternatives to that at this time—actually the Schrire proposal.

**Nic Lughadha** followed up on Ladiges’s “storytelling” by asking, if we can deem that things were published in 1754 by Miller, could we not equally deem that they were conserved by the Committee for Vascular Plants last week? [Laughter.] Otherwise, she felt that the proposal was seriously flawed if there would be a gap, where we had to wait to see [if the names were conserved].

**Applequist**, as the person who had raised that alarm, felt she should say now that two of the Committee members felt that if the Section democratically voted for this proposal, the Committee would be bound—whatever their own feelings—to conserve the names the Section instructed them to conserve and she hoped that others on the Committee would feel likewise.

**Greuter** was speaking in fact neither for nor against any feeling of *Acacia* lovers. He was speaking for the *Code*, and the *Code* won’t be happy with this. [Laughter.] Nor, he predicted, would the Editorial Committee. He saw a number of technical problems if the proposal was accepted as presented, but could not even see the solution for the future Editorial Committee. For instance, there was a rule in the *Code* somewhere, he thought Art. 6, that a name in order to be validly published must be effectively published and in order to be acceptable at any way must be validly published.

He thought a motion had been approved earlier, an Article saying conserved names were validly published, even if they were not in the place where they have been deemed to be validly published in the conserved entry.

**McNeill** noted that this applied only to family names in App. IIB.

**Greuter** repeated that it was only for the family names, so it would not even apply to *Acacia* and *Protoacacia* and *Austroacacia*. He argued that they could not be conserved if that was not in. Further on it was deemed that all combinations under these nonexistent and nonconservable generic names were validly published when they were formed, which was not the case. They were not. They were not conserved. So even if you had a rule that these names were conserved, the generic ones, it would not allow the formation of names under the conserved imaginary generic names to be usable. They would not exist under the *Code*.

There would be so many basic principles in the *Code* overruled and affected by this unusual proposal that he could not see how it would be possible to make enough cross-references to implement them at all. He warned against accepting it. However his feeling may be one way or the other, he thought it was unworkable.

**Potgieter** had a point of order, there should be no “o” in “*Acantho*-”.

**Gandhi** was in a very unusual position to express two opinions, because he was given thoughts from two different institutions. From Harvard, they were reluctant to accept this compromise proposal because the two names that were not published by Miller were being ascribed and attributed to him as though he published them. He was concerned that a few in the audience may not realize that Miller did not propose them. However, from India, they had asked him to support this proposal. So he would be voting in both ways. [Laughter.]

**Flann** pointed out that if this was not accepted, then there was a solution, as had been mentioned, in that the Congress had voted then to accept the process that had occurred up to now. In her mind, it was more of a political issue than a nomenclatural issue. She thought that everybody wanted to resolve the ongoing dissent and dissatisfaction, but a compromise would only work if both sides accepted it. Personally, she would vote against the proposal unless it was clear that *Austroacacia* was broadly accepted as well as *Acanthacacia*.

**Geltman** felt fortunate that there were no acacias in his flora and really had no idea why the question was so sharp. However, he thought that it was a great danger for botanical nomenclature and felt that we must do as much as possible to ensure that it would be the first and the last exception, because he felt that the precedent could open gates for exceptions in other genera.

**Orchard** noted that it seemed to him that the meeting had perhaps, by consent, overlooked the basis of the whole controversy, and that was to conserve *Acacia* in order to stabilize over 1000 names. If the proposal was accepted, then *Acacia* effectively had no names. He asked, what did we vote for on Monday if not to vote for the stability of nomenclature?

**Smith** had to support the compromise. He was so requested and instructed by a great many people that were unable to attend. The reality was that there should have been an effort to have a compromise that would be acceptable to many parties. If it was not possible to reach that point, he thought the reality was that the controversy would just carry on until the next conference or the one after that. He added that this was not a threat. He thought a great many voiceless people who simply did not have the means to attend would be seeking ways that were legal within the provisions of the *Code* to undo this.

**Barrie** wanted to support what several other people had already said. One was Nelson, who pointed out there currently was no nomenclatural problem. He elaborated that it was not a nomenclatural problem because there was *Acacia*—all of the taxa had names that could be used, so no nomenclatural problem was being solved. The second was Greuter, because he felt it would be a nightmare to get this to work logistically over time.

He added that he thought 20 years from now people would wonder why this was in the *Code*. He suggested that students would go to Miller and say “Well, these names aren’t in the *Gardeners Dictionary*. What’s going on?” If the names started being used, he thought there would be a lot of people who would not be coming to the *Code* looking for answers but looking in the literature like they look for everything else.

As much as he appreciated the sentiment and intentions behind everyone who had worked on the issue over the last eight or ten years, he could not support the proposal and thought the issue should be kept as it was and just recognize that *Acacia* was conserved with an Australian type.

**Nicholas** reported that his colleagues who were ecologists and physiologists had said to him that they would continue to use *Acacia* no matter what was decided at this conference, and this worried him. The reality was that people in Africa were going to continue to use *Acacia*. He felt that a solution was really needed and, while not wanting to insult anyone, noted that at teatime, someone actually called it “nomenclatural imperialism”, so that was how deep the feelings were running.

**Rico** wanted to point out the degree of confusion that had been created so far in that there were already publications out that had two entries for the same species, one as *Acacia* and also for *Vachellia*.

**Ford-Werntz** referred to Nicholas’s comment that his nontaxonomic colleagues were going to use *Acacia* regardless. She wondered if he was speaking for or against the proposal in that case, and if he was saying that they would not adopt the *Acanthacacia*, *Austroacacia* names either.

**Nicholas** had not managed to talk to them, but was sure that they would be happy with *Acanthacacia* because it was descriptive and it retained *Acacia* in the name. They were not happy with *Vachellia*.

**Fortunato** was part of the legume group, people who worked and also published on *Acacia*, and the new proposal that the Section had now, as divided into *Austroacacia* and *Acanthacacia* by continents, was not a solution as it was much too complicated. She noted it was just a vision of people who work in *Acacia*. You can say in other positions *Acacia* was a genus just as divided, and the new proposal was more complicated.

**Knapp** asked if Fortunato was speaking for or against the proposal.

**Fortunato** replied that she felt that it was possible to accept it, but after that, new things, new changes would be made.

**Barrie** made the point that under the current system and current conservation of *Acacia* people were well within their right to use *Acacia* in their names, they did not have to use *Vachellia* or *Senegalia* if they were using *Acacia* in a broader sense and that had not changed at all.

**McNeill** commented that under the *Code* there were other mechanisms by which the name *Acanthacacia* could be the correct name for what would otherwise be *Vachellia* that did not require this particular proposal.

**Knapp** lamented that she had just told somebody else they could not talk about that! [Laughter.]

**McNeill** apologized and justified that it was in response to a comment.

**Knapp** added that since McNeill was sitting up the front she could not point at him in that same sort of way…

**Karen Wilson** was very sympathetic to the issues that some of our colleagues were facing, also sympathetic to the users here and issues that they would face. If there were other ways of reaching the same result, then she thought it was actually relevant to hear about them now because that would certainly influence how she voted on the issue.

**McNeill** clarified that there was no mechanism for reaching the same result, it would be a mechanism for achieving a result in which *Acanthacacia* would replace *Vachellia*.

**Karen Wilson** was interested to know from some of the Africans or Americans whether that would influence their thinking too.

**Knapp** suggested that discussion should stick to the proposal now so as to not muddy the waters.

**Rico** thought it would be better to have *Acacia* in a word than no *Acacia* at all in any words; at least some people in each continent would know that the thing belonged in the past to something that was an *Acacia*. To some extent, the prefix of *Acanth*- or *Austro*- would give you the idea where it was sitting in the cladograms.

**Levin** suggested a card vote at the outset, because it was a pretty critical situation, unless that was out of order to call it at the start.

**Knapp** wished to stick with the procedure as it was correctly laid out, first a show of hands and after the show of hands, if people wished to have a card vote, they could so call that.

**Nic Lughadha** thought that this was such a political issue that people would probably vote more freely in a card vote and suggested that just on this one occasion it might be best not to have a show of hands.

**Knapp** made a proposal from the Chair, noting that Nic Lughadha and Levin had both proposed a card vote. If she could have three people to second that, the Section could do a card vote from the outset.

**Annette Wilson** made what she termed a point of order: that she saw no reason to hide this vote if the Section had not hidden any others.

**Knapp** noted that she was very sorry but that was technically not a point of order and she was not going to take it as such. She said that she had made a proposal from the Chair, it had had a number of seconders, and she suggested now that the Section was going to…

**McNeill** said the Section was going to take a vote as to whether people wanted a card vote immediately.

**Knapp** agreed to take a vote as to whether people wanted an immediate card vote or whether they wanted to vote a show of hands first and then whatever happened after that.

[Turland said something off-microphone; McNeill laughed.]

**Knapp** exclaimed that Turland could just be consigned to outer darkness. [Laughter.] She clarified that Turland was suggesting that we discuss the proposal for a card vote, which she deemed had just occurred, so she asked the Section if they were ready to vote on whether or not to have a card vote. [There was general agreement and the Section voted for holding a card vote on the proposal immediately.] For complete clarity she outlined that voting “yes” for the proposal that was put forward by Schrire from the floor meant that we would have three names, one *Acacia*, one *Acanthacacia* and one *Austroacacia*, all simultaneously proposed by Miller in 1754. The names *Acanthacacia* and *Austroacacia* would need to go to the Committee for Vascular Plants to be conserved and then there would be a number of things following on from that. Voting “no” meant you did not approve of the proposal. [Time passed.] Knapp reported the results of the card vote: the “yes” votes were 169 and the “no” votes were 396, which meant that the proposal failed by a 70% majority vote.

**Turland’s proposal** was **rejected**.

**Knapp** wished to put on record, that as Chair, she very much appreciated the civilized way in which the debate was conducted. She thanked everyone who contributed and participated for being calm, collected, brief and very to the point and felt that a round of applause was due. [Applause.]

### Article 52

[*The following debate, pertaining to Art. 52, took place during the Ninth Session on Friday morning.*]

**Prop. A** (17: 10: 81: 0) and **B** (16: 2: 90: 0) were ruled referred to the **Editorial Committee**.

**Prop. C** (4: 97: 8: 0) was ruled **rejected**.

**Prop. D** (12: 71: 26: 0).

**McNeill** noted that Prop. D, which was the addition of a new Note with an Example, had been heavily defeated in the mail vote—65% “no”, but not by 75%.

**Gandhi** was curious to know from the audience whether the cited Example was not clear or that Example was wrong and would not be accepted.

**McNeill** explained that the Rapporteurs’ comment was that the suggestion was implicit but it was perhaps not necessary, because a Note was there for something that was implicit in the *Code* but was not altogether explicit. The Rapporteurs comment that this “ought to be quite obvious from Art. 11.2. If it were felt that a frequent mistake was to regard as illegitimate under Art. 52 a new name that included the type of a name at a different rank with a different final epithet, then a suitably reworded version of the Note (with ‘does not make’ changed to ‘does not in itself make’) might be considered.”

**Gandhi** added that the only reason he thought that this Example was an interesting one was when an author was using a different epithet from a different rank, one of his colleagues had argued with him that the proposed new name was superfluous and illegitimate. He strongly disagreed with the person but the person did not agree with him, so he thought this kind of Example would be useful.

**McNeill** stated that the Example would certainly be looked at by the Editorial Committee and he had no reason to suppose it was incorrect.

**Gandhi** wished to withdraw the proposal if the Editorial Committee was willing to consider it.

**Prop. D** was **withdrawn**

### Article 53

[*The following debate, pertaining to Art. 53, took place during the Ninth Session on Friday morning.*]

**Prop. A** (34: 63: 8: 0) was **withdrawn**.

**Prop. B** (16: 50: 40: 1) was ruled referred to the **Editorial Committee**.

**Prop. C** (8: 95: 8: 0) and **D** (8: 92: 9: 0) were ruled **rejected**.

**Prop. E** (32: 50: 27: 0).

**McNeill** introduced Art. 53 Prop. E, which was to add text to Art. 53 voted Ex. 9, regarding the confusability or similarity of *thibeticus* and *tibeticus*, *thibetensis* and *tibetensis* and, for that matter, *tibetanus*. He added that there was a negative mail vote: 32 in favour, 50 against.

**Barrie** was sympathetic to putting *thibeticus* and *tibeticus* as being confusable but was not happy with, and argued against, considering the endings *ensis* and *anus* and *icus* as all confusable. He thought that was a bad idea. He was against the proposal.

**McNeill** thought that the proposal was solely suggesting that each pair was confusable; not for a moment that *tibeticus* was confusable with *tibetensis*. He added that the Rapporteurs’ feeling was that there were rather a large number of Examples in the *Code* already dealing with Tibet and wondered if another one was necessary.

**Barrie** said that in that case he did not have a problem with it.

**Gereau** thought that some of the epithets listed in the reworded Example were likely to be confused, but certainly not all of the variants were necessary and he felt it should go to the Editorial Committee for their considered opinion.

**Knapp** suggested sending the addition of the text about Tibet to the Editorial Committee…

**Turland** pointed out that that was not possible as it was a voted Example.

**McNeill** thought that the Section would have to at least endorse it with the caveat that the Editorial Committee need not include them all or something to that effect, because with the voted Examples it was necessary to agree that these were confusable. He did not think there was any question that if one pair was the others were also, because the terminations were the same.

**Glen** had what he framed as a dumb question: “do we really need to have a complete list of every possible pair of confusable epithets in the *Code*, because this seems to be occurring in Ex. 9?”

**McNeill** noted that there was a proposal for an Appendix that he thought had been adopted.

**Kellermann** thought the “t” and “th” example was quite useful. There were several cases where that was a problem but seeing that an Appendix had been voted for, all of the similar examples probably should move into the Appendix and that was up to the Editorial Committee.

**McNeill** thought that he was hearing that the vote would be in favour of confusability with the caveat that the Editorial Committee should decide how many should appear in the *Code* and how many should be in the Appendix.

**Wiersema** wondered about the Appendix being set up to deal with the decisions under Art. 53.5. He wanted to know if that meant that these kinds of cases would have to go through the procedure of being submitted to the General Committee and get their decisions before they could enter that Appendix.

**McNeill** deemed that the decisions were of higher status than that because they were voted Examples in the *Code*. However, if an Appendix was introduced for the references and materials from 53.5, it would be sensible to include an extract from the *Code* of those situations that were also confusable, otherwise the Appendix would be incomplete.

**Wiersema** was not sure that the Art. 53.5 situation had actually been dealt with.

**McNeill** apologized, as it was still to come.

**Knapp** explained that a vote “yes” would be interpreted as these epithets were confusable but with the proviso that should an Appendix for these instances be approved that these would be referred to that Appendix and for the attention of the Editorial Committee.

**McNeill** noted that not all three would necessarily appear in the *Code*.

**Prop. E** was **accepted**.

**Prop. F** (16: 67: 25: 0) was **rejected**.

**Prop. G** (81: 16: 9: 1).

**McNeill** moved on to the matter of an Appendix that he had erroneously thought had already been decided on. This was Art. 53 Prop. G, by Paul Silva; a proposal to establish an Appendix to list binding decisions regarding confusability of names and to add at the end of Art. 53.5 “These binding decisions are listed in Appendix VIII”, or whatever number it would be.

**Prop. G** was **accepted**.

**Prop. H** (62: 1: 43: 0) was **accepted**.

**Silva’s proposal**

**McNeill** noted that Prud’homme van Reine had a new proposal from Paul Silva that related to Art. 53 and asked him to introduce it.

**Prud’homme van Reine** had received a letter from Paul Silva, who asked him to introduce a term, “parahomonym”, in Art. 53.5. There was a recommendation at the end whether or not to treat the names concerned as homonyms. The proposal was to add “para-” between brackets after “homonyms”. He added that the Committee for Algae was in favour of the proposal.

**McNeill** summarized that it would add parahomonyms to the *Code* in a manner in which it was defined.

[Aside discussion between Rapporteurs]

**Turland** pointed out that this would be the only place where the word “parahomonyms” appeared in the *Code* and he wondered if it would be adequately defined simply by putting “para” in parenthesis before the word “homonyms”.

**McNeill** had actually translated the proposal a little bit, to a slightly broader one to require the Editorial Committee to include “parahomonyms” in the *Code* in a manner in which it was defined. [The **proposal** was **seconded** and **supported** by three others.]

**Paton** described himself as a bit thick and slow and asked if somebody could explain what a parahomonym was.

**McNeill** explained that it was indeed just what was defined in Art. 53.5—names that were sufficiently alike to be confused but that were not homonyms. He had elaborated the proposal a little as there was no point in putting it in unless it was clearly defined.

**Demoulin** thought “para” should not be in parenthesis because they could not be homonyms.

**McNeill** clarified that the discussion was not on the precise suggestion of Prud’homme van Reine, but instead whether the word “parahomonyms” should be included in the *Code*, with a suitable definition in a suitable place in Art. 53 or even elsewhere.

**Redhead** was not certain it was needed because this was a recommendation of whether or not to treat the names concerned as parahomonyms. The Article was there to decide whether to treat them as homonyms, so why would they be treated as parahomonyms?

**McNeill** clarified that the issue was whether or not it was worth instructing the Editorial Committee to provide a definition for a parahomonym, which was “two names that were rather similar”. He explained that “parahomonym” was a wellunderstood word for a name that was almost a homonym, but there was a rule in Art. 53 that some such names were treated as homonyms and others were not, but they still remained parahomonyms.

**Nic Lughadha** was concerned to understand exactly what definition the Editorial Committee was proposing because “two names that were rather similar” did not do it for her. “Confusingly similar names” was the phrase with which she was familiar and if the intent was to say that parahomonyms were confusingly similar names then she would be prepared to consider it, but she felt that the Section needed to know what definition was being proposed.

**McNeill** accepted that and noted that, as it was worded, he thought it would have been confusing. The original proposal was in this context, in which it would have had to have been a confusingly similar name.

**Gereau** saw absolutely no utility for the proposal. He stated that we have homonyms. We have a rule that says we can rule things to be considered homonyms because they are confusingly similar. We call them confusingly similar names and after being so ruled they are homonyms by our decision, so what is the point of another term?

**Barrie** added a point of clarification, that after a name was ruled as confusingly similar the pair did not become homonyms—they become treated as though they were homonyms.

**Prud’homme van Reine** mentioned that there already was a definition and he was planning to propose to put that in the Glossary; he referred to a definition that Hawksworth had already given—“two or more words orthographically or phonetically so similar to each other that they were likely to be confused”.

**McNeill** noted that it could only be added to the Glossary if it appeared in the *Code*, and he was pretty sure it did not.

**Marhold** thought that the situation and terminology in the *Code* was clear enough and he did not think another term was needed. Epithets were either treated as homonyms or not and that was it. He did not think there was a need for another term that would just require other changes in other places in the *Code* and would confuse people.

**Stevens** called the question. [There was a sufficient majority in favour of voting.]

**Knapp** moved to a vote on the inclusion of “parahomonyms” in Art. 53 in the context of confusingly similar names.

**Silva’s proposal** was **rejected**.

### Article 54

**Prop. A** (33: 53: 4: 9) was **withdrawn**.

### Article 55

**Prop. A** (74: 0: 32: 0) was ruled referred to the **Editorial Committee**.

### Article 58

**Prop. A** (101: 4: 12: 0) was **accepted**.

**Prop. B** (11: 4: *94: 0) was referred to the **Editorial Committee**.

## Chapter VI

[*The following debate, pertaining to Chapter VI, took place later in the day during the Eighth Session on Thursday afternoon.*]

**Prop. A** (72: 7: 10: 4).

**McNeill** introduced Prop. A under Chapter VI, which was that the chapter title should be changed. Instead of “NAMES OF FUNGI WITH A PLEOMORPHIC LIFE CYCLE” it would become “THE NAMES OF ANAMORPHIC FUNGI OR FUNGI WITH A PLEOMORPHIC LIFE CYCLE”. He reported that the proposal had received strong support, both by the Nomenclature Committee for Fungi and in the mail vote.

**Prop. A** was **accepted**.

[*Here the record reverts to the normal sequence of events.*]

### Article 59

**Prop. A** (2: 77: 2: 3) and **B** (11: 70: 1: 4) were ruled **rejected**.

**Prop. C** (64: 11: 4: 5), **D** (35: 38: 4: 7), **E** (34: 38: 6: 7), **F** (61: 14: 4: 6), **G** (59: 14: 4: 5), **H** (58: 13: 5: 6), **I** (32: 40: 6: 7), **J** (50: 16: 10: 7) and **K** (61: 10: 7: 6) were **withdrawn**.

**Prop. L** (21: 55: 2: 6) was **rejected**.

**Prop. M** (13: 65: 2: 6), **N** (10: 66: 2: 6), **O** (10: 65: 4: 5) and **P** (11: 64: 4: 5) were ruled **rejected**.

**Redhead’s set of proposals**

**McNeill** moved on to Art. 59, which was going to be quite different from what was in the original documents, but the material had been distributed. Redhead was going to introduce the alternative proposals developed by the group of mycologists present. He added, for those who were not as quite as familiar with the *Code* over the years as others, that Art. 59 was the Article that dealt with the provision of alternative names for groups of fungi with a pleomorphic life history, where there was both an anamorph asexual stage and a teleomorph sexual stage. Historically the name of the fungus had been that of the so-called holomorph, the anamorph and the teleomorph, and the matter was what type of material was eligible as type of a holomorph. The provision, which many people with modern molecular understanding of relationships between organisms felt was anachronistic, allowed the ability to have two separate names for the same fungus depending on whether it was present in an asexual or a sexual stage. He described that as an ignorant vascular plant taxonomist’s summary of Art. 59.

**Redhead** had served as the secretary of a Special Committee that was put together after the Vienna Congress. At that time there were some changes to Art. 59 that were put forward by Hawksworth and were modified. A couple of the proposals put forward were contentious and they were sent to the Special Committee for consideration. They had worked on it for five and a half years, never quite reaching consensus on what to do. Ultimately he had published a report *on* the Special Committee rather than *of* the Special Committee, and then he took some of the ideas that were put forward within the Committee and published them as a series of proposals, which were in the synopsis.

Gams and colleagues had published a series of counterproposals and those were also in the synopsis. Together they had all been milling out what to do about it. Since then there had been a symposium in Amsterdam, and one of the things that had been discussed within the Committee was put forward, which was what would happen if Art. 59 was removed? There was a series of repercussions. So then he was asked to put together a proposal to present here, indicating what would happen if Art. 59 were removed. He hoped that amongst the mycological community here, including Gams and colleagues, that they had agreed to roll all the proposals in under Art. 59 and basically replace them with three different options.

The first option had been distributed in hard copy and was put forward as a new set of proposals that covered Art. 59 and some additional Articles. The second package would be basically the set of proposals that he had published in *Taxon* and were in the synopsis, with a slight emendation to it, in that he had forgotten to take out Recommendations. The third option was that same set of proposals but, if the first two options fell, then the third option was to recover some of the information that was in those proposals and eliminate one of the more offensive paragraphs to some people and remove a date. They would be presented as three different packages. He tabled the three sets of proposals to be debated for Art. 59.

**Knapp** asked for clarification as to whether he was presenting these to each be voted on as amendments to the *Code* with a 60% majority as three separate sets of votes; or as two options to be voted with a simple majority between the two options and then a modification of the other option.

**Redhead** clarified that if the first option was rejected, discussion would move to the second one and if that was rejected, the Section would go on to the third one.

**Knapp** just wanted to make sure that everybody understood that this was not a simple majority type vote between two options but the presentation of scenario one, a vote; scenario two, a vote; scenario three, a vote.

**McNeill** clarified that scenario two would not come to attention unless the first one was defeated.

**Dorr** was concerned about the procedure, as there was a set of proposals that were published and people were able to consider before the meeting. He wanted to know if that was being withdrawn and this being substituted, as he thought that was necessary.

**McNeill** agreed because if the material that was in the published area would never appear if the first one was passed then the published material was being withdrawn for the moment, although it may be returned to.

**Dorr** thought it had to be withdrawn and the proposal considered, and then if Redhead wanted to have the Section consider what was published before the meeting, that had to come up separately.

**Knapp** added that it would have to be re-proposed and have four seconders as it were.

**Dorr** just did not want to be put in the position of having to choose between three things.

**Knapp** agreed and, as had very clearly been pointed out, proposal one had been proposed by Redhead from the floor. [The option 1 **set of proposals** was **seconded** and **supported** by three others.]

**McNeill** added that this meant that all the other matters on Art. 59 had now been withdrawn, although they may come back later on.

**Redhead** agreed that was correct. He referred to the handout that had been available for at least two days and had been definitely announced last night.

Basically, he outlined that Art. 59 covered the scenarios where it was possible to have alternative names for sexual and asexual stages called teleomorphs and anamorphs. This had created a system where the same species or same taxon could have two or more names. The suggestion was eliminating Art. 59 totally so that the exception was eliminated from the *Code*, but then there was the concern that if you just removed Art. 59—because the *Code* was retroactive and because mycologists had been publishing alternative names simultaneously—that some of the names might become illegitimate or invalid, depending on the circumstances and typification. So Art. 59.2 was left in so that for that period of time when Art. 59 was in effect these names would not become illegitimate or invalid.

Basically the section above that in the printed handout eliminated the ability to publish alternative names. The second paragraph protected names, by catching them in a safety net, and then they had to be sorted out by the mycological community as to which names were to be used and which ones not to be used. The other proposals presented were ideas to try and manage the fallout of removing the protection of Art. 59 and they could actually be voted on independent of Art. 59 but they were there to buffer the effect.

So he started by putting forward the proposal:

“*59.1*. On and after 1 January 2013, all names of fungi, including fungi with mitotic asexual morphs (anamorphs) as well as a meiotic sexual morph (teleomorph), must conform to all the provisions of this *Code* that are not restricted in application to other groups of organisms or from which names of fungi are not specifically excluded.

*Note 1*. Previous editions of this *Code* provided for separate names for so called “form-taxa”, asexual forms (anamorphs) of certain pleomorphic fungi, and restricted the names applicable to the whole fungus to those typified by a teleomorph. All legitimate fungal names are now treated equally for the purposes of establishing priority, regardless of the life history stage of the type.

*59.2*. Names published prior to 1 January 2013 for the same taxon of non lichenized *Ascomycota* and *Basidiomycota* with the intent or implied intent of applying to, or being typified by separate morphs (e.g., anamorph, synanamorph or teleomorph) are not considered to be alternative names under Art. 34.2; nor are they to be treated as nomenclaturally superfluous under Art. 52.1. If they are otherwise legitimate, they compete in providing the correct name for the taxon under Art. 11.3 and 11.4.”

He noted that there was a growing body in mycology, because of phylogenetic work, where there were intermingled genera and intermingled species names sometimes for the same species with different names and different genera. There was also concern amongst other communities about the standard way of doing things and how that would confuse things, so there was some debate here.

**McNeill** checked that at the moment Redhead was proposing the acceptance of the first part but not the additions to Art. 14 and that they should be debated separately

**Redhead** agreed that those could be debated separately, if the first part was accepted.

**Gams** referred to two other pieces of major background information that had also been available in hard copy. One was the Amsterdam Declaration of mainly molecular workers and applied mycologists. The other was the critical reaction to that document. Very briefly he pointed out that the mycological community was very strongly divided and he felt that even a majority was against the move to one species, one name. He urged the Section to abandon this proposal, which was exactly the expression of what in the Amsterdam Declaration was intended, and to reject this.

**Demoulin** agreed with Gams. He suggested that if you watched the history of discussions on the issue, the divide of about 50/50 between the partisans of dual nomenclature and of one fungus, one name was pretty stable. There was a very intensive debate at the International Mycological Congress in Oslo 2002, where there was a vote taken that came to about that percentage. He felt it should be noted that the Special Committee that Redhead had handled was also divided about 50/50. There was more support for one fungus, one name inside the general fungal committees that he chaired, but he felt that the difference between the Special Committee and the Committee for Fungi lay in the fact that the Special Committee was composed of people directly concerned by anamorphic fungi, while the Committee for Fungi probably had a majority of agaricologists and then some lichenologists and very few people working with anamorphs.

He referred to the Amsterdam Declaration, where the majority of people were in favour of one fungus, one name. But he suggested that it was really a meeting of the convinced, like a conference on intelligent design where they invited one evolutionary biologist, politely listened to him and then said “Yes, we were tolerant, we’ve listened to somebody representing this standpoint but we stick to our standpoint”. He felt that all the discussion about the Amsterdam Declaration was highly political and not very scientific. He argued that it was trying to give the impression that the large majority of mycologists and mycological associations were supporting the Declaration.

He gave one example of the so-called European Mycological Association. He had been involved with European Mycological Congresses since 1966 and the Association was mostly the grouping of people who organized the Congresses. He had never seen any activity of the Association outside of organizing Congresses, which at the beginning were essentially field trips. He stated that he came from Belgium, a small country with a limited number of mycologists, and he thought he knew them all. He had looked at the list of people having signed the Amsterdam Declaration and he had discovered two Belgian mycologists he had never heard of before who had signed it. He added that if you looked at the signatories of the other declaration, the one of Gams and his colleagues, there were also two Belgians signing that declaration, himself and André Fraiture, who was supposedly the Belgian representatives at the European Mycological Association that we were told was unanimously in favour of the Declaration!

**Knapp** thanked Demoulin and urged everyone to be quite brief. She then recognized “the man with the 2002 International Mycological Congress t-shirt”.

**Kirk** supported the proposal. He offered a little bit of context as editor of the *Index of Fungi*, a twiceyearly current awareness of new nomenclature, which was the equivalent of *Index Kewensis*. In the last 18 months he had noticed a change, such that in the July issue 5% of the names—higher for those names relevant—would have a comment: “Validly published but contrary to Art. 59”. He claimed that there was already anarchy and if nothing was done now, in six years’ time it would be open warfare.

**Alvarado** thought that if the proposal was going to take effect there should be a provision to keep the teleomorphic and anamorphic names that had not been matched together. So that a single name would occur only for those taxa that people were completely sure were matched. He did not think it would be good to just say the classification stops on this day and then the names were not available anymore.

**McNeill** explained that there was no reason under the *Code* why it would not be used.

**Redhead** agreed that that was correct, and felt it was a misunderstanding.

**Wiersema** knew that the mycologists at his institution were sympathetic to the movement toward one fungus, one name, but all the opinions that had been expressed and circulated and to which he had been privy had been opposed to the Amsterdam Declaration proposals that were being presented, so he would have to vote against them for the institutional votes.

**Lendemer** intended to vote the exact same way. He thought there was a perfectly good reason why there were two sets of names and he thought they were perfectly usable. He added that they were really great when you had to file specimens in a herbarium and put a name on a specimen, because they did not always have both states; any specimen could be one or the other state. He thought that if one name was adopted people would start saying this genus, this species, anamorph, teleomorph, which he felt was practically moving back to polynomials and did not seem very much like progress to him. There was something else he was going to say, but he had lost it. [He laughed.]

**Knapp** warned that he had better say it now because she might not call on him again. [Laughter.] She then relented and said that she was just teasing him.

**Greuter** was not going to speak either for or against the proposal but he was willing to say that from a nomenclatural point of view it looked appetizing, clean, workable and easily applicable. He had two questions for the proposers. One was whether anyone had assessed the nomenclatural consequences of adopting the proposal. One of the main worries he saw was, for instance, the fact that in the past teleomorph genera may have been published and provided a name, while including anamorph genera as a synonym, so that if this was passed now they would become illegitimate. If that was an unjustified worry he would be pleased to know.

The second thing he wished to know, before voting on the first option, was if the second version or scenario was worse than this one maybe he might vote in favour of this rather than being thrown back to the second one.

**McNeill** answered that the second scenario, as he understood it, would be the material on a subset of the proposals in Art. 59 that had been presented in the synopsis that received substantial support from the Nomenclature Committee for Fungi.

**Redhead** responded to the concerns about the effect, and noted that it was debated through the Committee. They had gone through several examples to see what the effect would be. The conclusion was that in some cases there would be substantial effect, but nobody had the time to go through all the scenarios. That was why they had put in the second set of proposals [as part of option 1] and hoped that special committees would be put together to look at selected groups and then draw up a list and say “This is the list of names that we want to accept for this group”. He envisaged that they would then be approved via some mechanism, which had been outlined, so those were the safety nets for the potential effects.

**Knapp** queried whether it was part of option number one.

**Redhead** suggested that the second set of proposals could be voted on separately but they were all part of this package. He added that if the Section voted to accept the first part then it would be necessary to consider the next two.

**Knapp** decided to be nice and allow Lendemer to speak twice because he had remembered what he wanted to say.

**Lendemer** thanked her. [And laughed.] Having heard someone say that people were submitting names with the statement that it was contrary to Art. 59 his attitude was if they were not following the rules, too bad. He did not feel that the Section should create rules and make changes just to recognize people doing something wrong.

**Unknown speaker** added that they would just break them too.

**Lendemer** agreed. He did not see the point in recognizing people doing something wrong. He reminded the Section that if they did not vote for any of these three options Art. 59 would remain in place and nothing would happen.

**Knapp** asked for other comments from people who had not made comments before but also asked them to remember everything they were going to say when they were given the microphone.

**Hawksworth** wished to explain a bit more of the history of the issue. When it was discussed at the International Mycological Congress last year and there was a vote on it, the majority of mycologists, including mycologists across the board, not just ones specialized in fungi that had the problem, were for working towards one name for one fungus. He reported that they wanted that to be done in a way that would result in the minimum disruption of names and that it was not clear how that could best be achieved.

The meeting that was convened in Amsterdam was deliberately organized in order to try and come up with a roadmap of where to go. It was an open meeting, obviously people with particular interests were those that went, but it was deliberately one where people involved in applied areas of mycology were represented, because these were some of the areas where people had particular problems with this issue. Particularly those dealing with plant quarantine and health and safety and so on, where people in biology generally and in regulation just did not understand why there was a problem with different names.

His concern was to try and get a reasonable way ahead because the current situation, as Kirk had mentioned, was that it was rampant, that people were ignoring this. In a single publication that came out of collected papers this year, there were five different ways this was being used in different papers. It was total chaos. He suggested that the proposal was the only way to actually have a clean approach forward, and he was afraid that if it did not pass the chaos would continue. He concluded that the current *Code* was not being followed and that options two and three did not go far enough toward what people actively wanted, and so he felt this was a route the Section had to take.

**May** appreciated the difficulty of non-mycologists in trying to decide how to vote on this issue. In effect, he suggested that if one was interested in allowing the mycologists to have a say on this it was worth considering what is the majority that is usually used, that it would take a 60% majority to pass it. He thought, being very fair and looking at the wider mycological community, each time the issue had been put there would have been a 60% majority. He referred to Demoulin’s comments about particular organizations including the International Commission on the Taxonomy of Fungi, various national fungal bodies and so on. He acknowledged that when those bodies supported the Amsterdam Declaration, in some cases it was not unanimously, but he thought it was a very fair comment to say that all of those organizations had consulted their members. The bodies he was on had all consulted widely and in all of those bodies there would have been more than 60% support for ratifying the Amsterdam Declaration.

He concluded that there had been attempts to come up with middle-ground compromise solutions that worked towards it, but they were so messy that it was just not going to work. He felt that it was necessary to take a quantum leap and he felt that the community was ready for it. Redhead had got the provisions in there for the consultation of groups like the International Commission on the Taxonomy of Fungi to draw up the list, so there were mechanisms to deal with the conservation proposals that would be necessary. He argued that the mycological community wanted to move towards this but the move had to be quantal and if done incrementally he felt it would be more chaotic than either not doing it or going there in one hit.

**Luckow** felt kind of weird speaking, since she was a non-fungal person, but the mycologist from her institution did speak to her about the issue before the meetings and she had serious reservations about going forward with this proposal.

**Prud’homme van Reine** noted that phycologists also had to deal with different generations, gametophytes and sporophytes. The first that had been published had priority and in each genus all the species had to follow that. There was no rule if the sporophyte or the gametophyte had priority. The mycologists did it in another way. He thought that they had to clean up the mess but the decision had to be made here.

**Knapp** asked if he was speaking for or against the proposal.

**Prud’homme van Reine** responded “Ummm…” [Laughter.] He thought against.

**Knapp** commended him for his honesty.

**Gandhi** was also a non-mycologist and wanted to convey that his department supported the proposal.

**Johnston** thought that there were two issues being dealt with here. One of them was whether it was sensible to have two separate names for a single organism. He did not think it was. The second was if there should be only one name for a single organism, then how to choose that name. It seemed to him that, under this proposal, basically a process of priority used widely throughout the *Code* was proposed. He felt it was a sensible, very simple and clean solution to the mess and he was speaking in support of the proposal.

**Gams** noted that so far there was the clear rule of precedence of teleomorph names and the whole system of *Ascomycetes* was based on teleomorph genera. According to this proposal and the next one any genera, whether anamorphic or teleomorphic, would compete according to priority and would cause very drastic changes. He argued that it would all be committee work to decide which of these two competing genera were going to be switched. He felt that it was quite often not even desirable to make this choice and gave the example that in *Trichoderma* versus *Hypocrea*, *Hypocrea* was well known as *Hypocreaceae*, *Hypocreales* and so on and the teleomorphs, anamorphs were now usually connected. The anamorphs were better differentiated. He had no objection to using the anamorph names for most of the species, but unfortunately several of them did not have an anamorph at all or it was not *Trichoderma*-like.

He believed that the Amsterdam Declaration and its adherents were inspired mainly by a few well-studied genera of technical or economical importance. He felt that in facing the whole biodiversity of *Ascomycetes* only a minority of these fungi had been critically studied with molecular techniques and that had to be taken into account. He maintained that it was not justifiable to synonymize names that had been connected as anamorph and teleomorph of the same taxon, because the molecular evidence was not yet available.

Some defendants of the one name, one fungus move compared the situation with the development of insects. He did not think that was fair. Every insect larva would develop into an imago and that defined the species. But with fungi that was absolutely erratic whether there was exclusively an anamorph or by chance the teleomorph available.

**Kirk** explained that the scenario that he had mentioned in his previous comments were from forest pathologists, not systematists. They were taking the naming of the organisms important to them into their own hands, because the current system did not work. He responded to Gams, saying yes, there would be a lot of work, but he claimed that “It’s our mess, we’ll clean it up.” [Pause.]

**Knapp** exclaimed “Right, no holding the microphone and not talking!” [Laughter.]

**Kirk** continued, saying that the priority names would be dealt with because users of names outside systematics want them dealt with. According to him, insignificant organisms that had been seen by one or two people on the planet Earth were not important and just muddied the waters. He felt that Gams’s comments were not relevant to the users of names.

**Demoulin** thought that the issue where it was so divided was whether names were needed for what was standing in front of you, what you see or not. And he thought there were a number of cases where people would like to have a name to designate what they saw. That was a curse with the algae. It was perfectly true what Prud’homme van Reine had said, that the phycologists never tried to fix this issue and they accepted priority, but you may find in the algological literature comments that it would be nice to be able to name those very different things; and—even if it was not legal—you would see it still in many papers and books.

For example, the red seaweed called *Asparagopsis armata*, because this was the name that was chosen in the ’30s when the Feldmanns discovered that two very different morphological things, *Asparagopsis armata* and *Falkenbergia rufolanosa*, were a gametophyte and a sporophyte of the same thing. They chose the name of the gametophyte, but you very often just see the sporophyte that reproduces vegetatively because the climate conditions were not favourable for formation of the gametophyte. And in vegetation description in the Mediterranean, for example, you will see it under the name of the stage that you see—that is, *Falkenbergia rufolanosa*. He took this as proof that it was useful to have a dual nomenclature, since people who were not allowed to do it did it nonetheless.

**Cafferty** asked Gams or Demoulin to respond to the comment by May that the proposals had consistently received 60% support from the mycological community, he wished to know if they agreed with that.

**Knapp** asked for one-word answers: Yes or no.

**Gams** began by saying 60%…

**Knapp** reiterated that it was a yes or no question.

**Gams** thought that 60% would be true but…

**Knapp** interpreted that the answer was also “yes”.

**Demoulin** did not have the numbers, but trusted Gams.

**Knapp** summarized that the answer was “yes”.

**Gams** repeated that it reflected exactly the division of the mycological community.

**May** responded to the idea of naming different stages in the life cycle. He reminded everyone that one of the principles of the *Code* that was right up at the beginning was that each taxonomic group with a particular circumscription, position and rank could bear only one correct name and then it went on to the say “except in specified cases” and he argued that this was one of them. In terms of databases, and he pointed out that there were a lot of integrated global databases of biota, the existence of two different names for one fungus was very chaotic outside of the particular user community.

He noted that in all other cases, whether it was plankton that had a completely different-looking free-swimming form and then they settle down to form something, or butterflies, in no other biota could you have *Code*-compliant or *Code*-mandated names for the different stages. He concluded that it was an anomaly for the fungi and it was necessary to get rid of it.

**Price** called the question. [There was a sufficient majority in favour of voting.]

**Knapp** moved to a vote on option 1 of Art. 59: Art. 59.1 and 59.2, which belonged together.

**McNeill** added that this was excluding the part of how to look after the problems, as that had not yet been discussed.

**Knapp** confirmed that the vote was on the section before the corollaries.

[The **proposal** was **accepted**.]

**Redhead** explained that there were three other parts to be discussed: a part for Art. 14, a part for Art. 56 and a part for Art. 57.

**Knapp** confirmed that Redhead was suggesting each of them be voted on separately.

**Redhead** agreed and began by introducing the first part to be considered: **Art. 14 (new)** “For fungi, permanent lists of names” as Appendices to be numbered in the future, obviously in this case these [are names] “registered and assigned either historically or currently to selected specialized taxonomic groups” [quoted from the proposal]. He suggested that this meant that the rusts or the smuts or some other group could be carved out where there was a group of specialists in the world—or the *Basidiomycetes* or the *Ascomycetes*. The idea was to send them to subcommittees appointed by the Committee for Fungi in consultation with the International Commission on the Taxonomy of Fungi, and the accepted names on these lists were developed and then they could be conserved as a block. This would be a way for the mycologists to handle the lists internally with specialists involved in those particular groups.

**Applequist** thought that this seemed to be an attempt either to create something like the names in common [sic] use that was resoundingly defeated some years ago, or an attempt to substitute the taxonomic judgement of some elite group regarding which anamorphs and teleomorphs went together for the taxonomic judgement of the community over time. She maintained that such proposals did not have a place in the botanical *Code* because the community had repeatedly said it did not want them there.

**Buck** had a question for the Rapporteurs. Was there any other reference in the *Code* to a group outside of IAPT, like the International Commission on the Taxonomy of Fungi? He thought not.

**McNeill** was also thinking it would be appropriate to ask, because personally until he saw the Amsterdam Declaration he had never heard of the organization. He invited the proposer to explain what the International Commission on the Taxonomy of the Fungi was.

**Buck** added that he was not sure it was appropriate for our group to tell another group of which we were not a part what to do, so he thought it was inappropriate to have a non-IAPT group embedded in the *Code*.

**Knapp** thought that other group would be consulted by the Committee for Fungi.

**Buck** felt that the point was that they would be embedded in the *Code* and they were not part of this organization.

**May** moved an amendment to suggest a wording along the lines of “appropriate international bodies, such as the International Commission on the Taxonomy of the Fungi”. [This was **accepted** as a **friendly amendment**.]

**Greuter** had several points. The first and most important was that a relatively sweeping change of Art. 59 had just been accepted, and the Section had been made aware that it would entail many disruptive changes of names. He believed that the only way to deal with that flood was to establish such lists as were advocated. He thought they would probably be something comparable to what was proposed as the Names in Current Use list—not “common”. He noted that these were defeated very strongly at one Congress and just defeated at another so he argued that it was not quite correct to say that the community was unanimously against them.

He suggested leaving the past out of the question and thought that such lists were needed for the mycologists. He believed that they would be useful and essential to avoid risking similar revolutions as had been experienced in the recent past.

He raised another issue regarding how the lists would be governed and embedded and he was glad of the amendment that had been passed. However, he was not glad about the fact that the Committee for Fungi was figured as something autonomous and autocratic, as in the nomenclature hierarchy the General Committee was above all other Permanent Committees, and it was that body with which in the first place the Committee for Fungi and this subcommittee should liaise. He formally moved that “in consultation the General Committee” be added ahead of the other one. [This was **accepted** as a **friendly amendment**.]

**Demoulin** noted that there was a question that had not been answered—what was the International Commission on the Taxonomy of Fungi? In his opinion, there was a big problem in the governance of fungal nomenclature and taxonomy outside of the well-established rule of the IAPT and someday that would have to be fixed.

**Hawksworth** explained that the International Commission on the Taxonomy of Fungi was an inter-union body between the International Union of Microbiological Societies and the International Union of Biological Sciences, established in 1986, that looked at issues to do with promoting taxonomy. It had been brought to the attention of these Congresses before because it was a subcommission of that group that produced the list of names in *Trichocomaceae*, which was the subject of a special resolution in the Tokyo Congress in 1993. And they actually produced a list there for protective purposes, partly because of the difficulties with this Article. He added that it was not true to say that this body was not known to the series of botanical Congresses.

**Barrie** agreed that there was an issue with the way the thing was being structured, because in all other cases where there was special consideration of names, or lists of names, they were referred in the first instance to the General Committee and the General Committee then referred them to the Permanent Committee and whatever subgroups they might have. It was published in *Taxon* and then it went to the Committees; the secretaries picked them up. That was the way it was structured, and it was the way this should be structured. He suggested that the lists be referred to the General Committee first and then referred down to the other bodies and noted that it would apply for both the Art. 14 and Art. 56 proposals.

**Knapp** asked for clarification as to what exactly he was proposing.

**Barrie** thought that it should be reworded in some way so that it said that these lists that were being set up were in the first instance referred to the General Committee. He suggested that was the way the nomenclature group here kept control over what was going on, because the General Committee was the main body that had authority between Congresses.

**Knapp** asked if he had a suggestion for how it should be so worded.

**Barrie** did not.

**Knapp** asked him to help out a bit.

**Barrie** apologized [laughing] and described himself as an “idea guy”. [Laughter.]

**McNeill** suggested “Accepted names on this list, once reviewed by the Nomenclature Committee for Fungi and the General Committee become…” He felt that it was the final process that needed to go through the regular channel of approval. [This was **accepted** as a **friendly amendment**.]

**Knapp** noted that everyone was still smiling, which she thought was quite a good sign.

**Wiersema** did not see any provision for listing the types of the names and felt that the names were meaningless without the type being there. He wondered if this needed to be included somewhere.

**Knapp** heard someone up there say “Taken as read”, so she thought names and their types might be added. She encouraged the Section not to wordsmith the *Code*.

**Redhead** noted that currently they looked at lists of conserved or rejected names and there were names there that had no types, so in some cases a name was rejected and it did not matter whether it had a type.

**Lendemer** offered a friendly amendment, for a group that had never been impacted by Art. 59—because apparently they had played by the rules—which was lichen-forming fungi. He thought that they had always been excepted from Art. 59 and that they should be excepted from the impact of the decision. He suggested that at the beginning it should say “For nonlichenforming fungi” to parallel what used to be in Art. 59, which had now been changed, and what had just passed. [This was **not accepted** as a **friendly amendment**. It was **seconded**.]

**Dorr** asked that the amendment, if it was accepted, be parallel to the language of [the newly approved] Art. 59.2; he was not sure that the Editorial Committee would do that.

**McNeill** assured him that they would use the phrase that was used in the *Code* generally for lichens.

**Dorr** deferred to McNeill and the Editorial Committee.

**McNeill** was sure they would make it balance.

**Knapp** pointed out that somebody was bound to call them up on it if they got it wrong.

**Demoulin** thought that the amendment was absolutely logical and in line with Art. 59.2, which had been accepted. He thought there may have been some misunderstanding by Redhead and asked him to explain why he did not consider that a friendly amendment.

**Hawksworth**, speaking as one of the two lichenologists in the room, preferred that it was left out, for the simple reason it was a biological situation and in many cases the biology of the fungi was not known; it was not known whether they were lichenized or not. He felt it was better to be left out because there may be cases where lichenized and non-lichenized groups were in the same genus where a list was needed.

**Norvell** called the question.

**Knapp** moved to a vote on adding the words “nonlichenforming” or their proper equivalent according to *Code* language to this Article.

[There was a sufficient majority in favour of voting on the **amendment** and there was a majority against so the **amendment** was **rejected**.]

**Funk** thought it had been decided that it was inappropriate to list any body that was outside the IAPT nomenclature structure, so why mention the International Commission on the Taxonomy of Fungi? She suggested an amendment to remove everything after “appropriate international bodies” and get rid of “such as the International Commission on the Taxonomy of the Fungi”, because that could change and then it was stuck in the *Code*. [This was **accepted** as a **friendly amendment**.]

**Van Rijckevorsel** was still very unhappy about the last sentence, especially “become the correct name”, because he felt that meant no more taxonomy was possible. He also wondered, the correct name of what? He was a little concerned about how it was to be conserved against competing names, while all provisions now in the *Code* were more specific, for example, with the same type.

**Greuter** distilled the consequence of these comments, which he wholeheartedly supported, into an amendment to remove “Become the correct name and” and replace the first line “that were registered and assigned either historically or currently to selected specialized taxonomic groups” with “established”. He suggested that would leave the mycologists the flexibility of structuring their committees or subcommittees as they wanted and they might regret having restrictions in afterward. [These were also **accepted** as **friendly amendments**.]

**Prud’homme van Reine** noted that the International Society of Protistologists had asked their group if a delegate of that group could be involved in the ruling of botanical nomenclature, and now they had one member on the Nomenclature Committee for Algae. He suggested that it could be done the same way for the fungi.

**Barrie** announced that he had run out of ideas and was ready to rewrite the proposal. He thought it should say “For fungi, permanent listed names may be referred to the General Committee”, so that it was similar to what was in Art. 14 and 56. “Permanent listed names may be submitted to the General Committee, which will refer them for examination to the Committees in the appropriate”—“for examination by the Committee for Fungi”. Then the Committee for Fungi can do it as…

**Knapp** summarized that the suggestion would make a parallel to the wording in other places.

**Barrie** agreed and added that he would also delete Appendices X and Y at the top, as that was not an appropriate place. He continued that then the names could be treated as conserved and listed in Appendices X and Y in the last line.

**Knapp** thought it was reasonable to assume that it would be put into more sensible English.

**Barrie** was hoping that the Editorial Committee would save him. He summarized that the general idea was to say that the lists were going to be referred to the General Committee and the General Committee would refer them in turn to the Committee for Fungi and any appropriate subgroups that the Committee for Fungi had established. Once they were reviewed, the Committee for Fungi would send the recommendations back to the General Committee who would review them again and once they were approved they would end up on the list. He added that this was the way things were structured now for conservation proposals, which were sent to the General Committee who referred them to the relevant Nomenclature Committee, who sent recommendations back, and the General Committee reviewed them and then they put them on the list [of conserved names]. He was trying to set this up so the same thing would happen with these lists. He did not think that the General Committee was going to be looking at the lists to begin with. He thought that what it also said was that the lists could come from anywhere.

**Redhead** clarified that the idea was to have subcommittees that would refer to it. They may be asked by the General Committee via the Committee for Fungi but then it was the subcommittees that submitted it.

**McNeill** thought the effect of Barrie’s suggestion would be that before they started being examined they would be published. He noted that may or may not be desirable, but being referred to the General Committee actually meant being published.

**Redhead** agreed that the registration lists would certainly be published and available.

**McNeill** meant that they would obviously have to be published before they could go in the final stage, but whether they needed to be in the first stage he deferred to the Section.

**Barrie** pointed out that it was new territory, so was not sure how it worked.

**McNeill** felt that it was not quite the same as the conservation proposal in that sense.

**Barrie** was just trying to make it analogous to it.

**Redhead** saw it, with cleaned-up wording, as a friendly amendment but deferred to Hawksworth’s opinion.

**Hawksworth** did not like the idea of the lists being published before they had actually been scrutinized more. He thought it would create a lot of paperwork not at the right stage. He added that if it was the feeling of the meeting that it was necessary to go that route, then we should go that route, but it seemed a rather unnecessary layer of administration to him.

**McNeill** concluded that it was not a friendly amendment but he could live with it if it was passed.

**Hawksworth** agreed.

**Knapp** moved to discussion on the amendment to add the fact that the lists of names would be submitted to the General Committee, which would refer them to the Committee for Fungi for examination. Also to move the “listed in Appendices” down towards the bottom. [The **amendment** was **seconded**.]

**Van Rijckevorsel** had two general points. Firstly, he noted that there were several provisions in Art. 32 and 53 in which something could be submitted to the General Committee and it would not be published until the relevant Committee had made a recommendation. His second point was that at the moment something only went into an Appendix after it had been passed by a Congress.

**McNeill** noted that the report of the General Committee was pointing out that the submission of references under Art. 32.4 and 53.5 should always be published, so that exception would disappear, all being well.

**Norvell** wondered if the General Committee wanted to do a lot of work. She suggested it would be better to refer them first to the Committee for Fungi to go through the list, then to the General Committee. This would cut down on the work for the General Committee, which may not be acquainted with these organisms.

**Buck** agreed with Barrie’s amendment, because he thought all the other proposals for conservation were done in the open air so why should the fungi be done in secret?

**Kirk**, in the interests of transparency, noted that all this work would be done in a publicly available online database. The publication, if there ever was one, would be published from the database. The alternative for those who were frightened of a list—non-seafaring legs—would be that a database would publish 500 proposals for conservation individually. He claimed it was not rocket science in terms of database content and publishing.

**Barrie** had a couple of ideas. One was that it was not necessary to publish the list in *Taxon*, a reference could be published to the list that Kirk was just talking about. He suggested that all that would be have to be done was announce the list was available right now for review at this date and then refer to the list. The other thing was that although there was this conceit that the General Committee received all the reports and then dispersed them to the various Committees, in reality what happened was that the Committee secretaries picked them up as soon as they were published in *Taxon* and the Committees started working on them. So it was not like the secretary of the General Committee was sending out letters saying these were the things that showed up and this was the time to start working on them. It was understood in the system that that was what happened. So that could be easily taken care of, especially if this list was going to be made available online. He suggested putting an announcement in *Taxon* saying the list closed on such and such a date, and those names would be reviewed now. And then it could be done periodically whenever necessary.

**Knapp** wished to know if he was speaking for his amendment and answered her own question “of course, because it’s your amendment”.

**Greuter** suggested that there had been insufficient clarification from the audience on what was exactly intended, but that it would not be possible to come up here with an exact wording that was not messy. So he suggested referring it to the Editorial Committee to be cleaned up, taking into account the comments received from the floor.

**Knapp** thought that the discussion was on the amendment to put it in the right sort of order first.

**Greuter** agreed that was his proposal and he also noted that for some reason the types had disappeared. He wondered if he had missed something, or if it was just a slip. He thought it was a friendly amendment, names and types.

**Knapp** noted there were rejected names that may not have types.

**Greuter** disagreed and stated that they would be lists of names treated as conserved, not lists of rejected names. He asked if that was not a friendly amendment. He thought it was.

**Knapp** clarified that it was not a friendly amendment and returned the discussion to the amendment on the order of General Committee, Committee for Fungi, Special Committees.

**Marhold** agreed with Greuter. He felt that there was a usual procedure for conservation of names that involved the General Committee and that there was no need to try to invent something special. Regarding the conditions of publication of this material, it was not written in the *Code* that it must be in *Taxon*; it could be electronically on the webpage of IAPT, whatever. He suggested leaving this all to the Editorial Committee to formulate it in a usual way as was normal procedure within the *Code* and not to lose time here trying to play with words.

**McNeill** heartily agreed.

**May** called the question.

[There was a sufficient majority in favour of voting on the **amendment** and there was a majority for, so the **amendment** was **accepted**.]

**Knapp** returned the discussion to the proposal for a new Art. 14, which she noted had been seriously amended. [She laughed.]

**Sennikov** felt that the mention of types needed to be added to be in practice with the rest of conserved names and proposed “Accepted names and their types in this list are to be treated as conserved”. He argued that this was necessary because types were usually mentioned in lists of conserved names. [This was not considered a **friendly amendment**. The **amendment** was **seconded**.]

**Lendemer** asked if he could call the question.

**Knapp** said that was not possible without having at least a couple of discussion points and people had actually had a chance to have their say.

**Buck** asked Hawksworth why he did not consider it as a friendly amendment, since all other organisms had to require types for conserved names.

[*The recording begins with audience laughter after an unrecorded portion, where David Hawksworth answered that the addition of types to the conserved names list was not considered a friendly amendment “because of the amount of work involved”.*]

**Knapp** [Laughing] “O-kay…”

**Wiersema** felt that if the types were not there, it would lead to endless problems trying to sort out all of the issues of the competing names on the list later on, and nothing would have been solved.

**Applequist** knew that the Chair did not approve of wordsmithing, but thought that there may be a certain distinction, technically, between conserving a name that had a type and conserving a name with a conserved type. The current placement of the phrase implied that the names and their types were to be treated as conserved. She suggested it may be better to say “conserved and listed with their types”. [This was accepted as a **friendly amendment** to the **amendment**.]

[The **amendment** was **accepted**.]

**Walsh** admitted it may be his misunderstanding or an unintentional ambiguity, and wondered if the lists for fungi were really permanent. In the penultimate sentence, were these names accepted at this stage or were they simply on the lists and then, subsequently, conserved and accepted? He suggested that maybe the words “Permanent” and “Accepted” could be removed.

**McNeill** asked the philosophical question “What was permanent?” Once on the list, he assumed the intention was that it would have the same permanence as the lists of conserved names, in the sense that names were not removed from them without some process.

[Inaudible, several people speaking at once without a microphone.]

**Kirk** thought that the problem was that it was permanent from the start. There was a problem with the word “permanent” in that position, Appendices could not be permanent and then subject to review.

**McNeill** considered it editorial.

**Marhold** thought that “accepted” should not be there.

**McNeill** thought it had to be there.

[Inaudible, two speakers stating the matter was editorial]

**McNeill** did not know exactly the nature of the lists being envisaged, but assumed that there would be synonyms included as well. He presumed that there would not often be a choice between an anamorph and a teleomorph name. He noted that both might be mentioned but it was the accepted names that were being conserved, not any other name that was mentioned in order to indicate the taxonomic association.

[The **proposal** was **accepted** as amended to read:

“*Art. 14 (new)*. For organisms treated as fungi under this *Code*, lists of names may be submitted to the General Committee, which will refer them to the Nomenclature Committee for Fungi for examination by subcommittees established by that Committee in consultation with the General Committee and appropriate international bodies. Accepted names on these lists, which become permanent as Appendices XX–YY once reviewed by the Nomenclature Committee for Fungi and the General Committee, are to be listed with their types together with those competing synonyms (including sanctioned names) against which they are treated as conserved. For lists of rejected names see Art. 56.n.”]

**McNeill** moved down to the next proposal, Art. 56 (new).

**Redhead** explained that, as a caveat to the earlier proposal that had just been dealt with, once names were got rid of, even if it was possible to figure out what they were, it was not desirable to have them resurrected, unless there was a very strong case, in which case that should be presented, because we do not want to keep going backwards in time. He felt that there was such a mess to clean up that once it had been decided that it was impossible to know what those [names] were, even if something was discovered later on, there would have to be a very, very strong case to get it back.

**Barrie** assumed that what Redhead was saying was that the only way get names back was by conserving them, and he suggested deleting everything after “only gain priority” and add “by conservation under Art. 14” or something like that.

**McNeill** explained that names that were rejected in the lists were to be permanently rejected as though under Art. 56. He added that what Barrie was suggesting was that they could only gain priority by being conserved.

**Barrie** agreed that that was correct.

**Knapp** clarified that the suggestion was just different wording—“can only gain priority by conservation under Art. 14”. [This was **accepted** as a **friendly amendment**.]

**McNeill** encouraged keeping the discussion to substantive issues, not precise wording, because it would have to be modified.

**Gereau** thought his comment was moderately substantive. When he had submitted an article to *Taxon* in 1994 in which he said that something should be considered a nomen confusum, Dan Nicolson had informed him that that term had not appeared in the *Code* for more than a decade. He did not believe that either “nomen confusum” or “nomen ambiguum” had any meaning under the *Code* today.

**McNeill** agreed that there was no provision of the *Code* that covered them and he thought it had been deleted in Leningrad. He thought that it sounded as though there was a situation that was envisaged for these lists, which had just been approved, that they would include tentative synonyms. He thought that the proposer should clarify what he meant by “nomina ambigua” and “nomina confusa” in that context.

**Redhead** noted that the descriptions of earlier fungi were just so vague that it was impossible to figure out what they were and they had been misapplied or dropped. He stated that when those terms had disappeared from the *Code*, it was envisaged that one could always lectotypify or neotypify or something but these things were so vague that you could just randomly pick almost any white fluffy something on a stick and say “Well, that’s it”.

**McNeill** wondered why these names would appear on the list of accepted names and where they would be linked to. It sounded to him as though what was really being suggested was that you should just operate the normal application of Art. 56. He interpreted that Redhead was saying that they were going to produce lists that would be rejected under Art. 56 in the regular way, and not a by-product of the other lists established under Art. 14.

**Redhead** agreed that they were trying to create lists of names they wanted to reject, so the lists established under Art. 14 may not have been the appropriate place.

**Greuter** had a problem with the proposal, in that it referred to rejected names listed in the Appendices established under new Art. 14, but no such rejected names were mentioned in the new Art. 14. He felt that this should be integrated if approved in the prior paragraph, and he thought it was worth approving, so that accepted names and rejected names could be mentioned. As that was too complicated to formulate here, he suggested that it be approved in principle and left to the Editorial Committee and not to mention it under Art. 56, but to mention it under Art. 14. He also saw no reason whatever to justify the rationale and recommended just treating it as if formally rejected, full stop. [These were both **accepted** as **friendly amendments**.]

**Knapp** moved to a vote on Art. 56 (new), with the proviso that it be referred to the Editorial Committee to put in the right place and in the right words.

[The **proposal** was **accepted** and referred to the **Editorial Committee** for wording and placement:

“*56.n*. For organisms treated as fungi under this *Code*, lists of rejected names may also be included in the Appendices established under Art. 14.n. Such names are to be treated as though rejected outright under Art. 56.1 and may become eligible for use only by conservation under Art. 14.”]

**McNeill**, before moving on to the final proposal, on Art. 57, asked the proposer if he really wanted this to be a paragraph of Art. 57, in which case it needed to be rephrased, or whether he really had this as a Recommendation, Rec. 57A (new); the point being it was written as “should”, at the moment, so it was not written as an Article.

**Hawksworth** stated that it was written to parallel the equivalent provision for names with misapplied types, based on the same wording.

**McNeill** referred to Art. 57.1: “A name that had been widely and persistently used for a taxon or taxa not including its type is not to be used in a sense that conflicts with current usage unless…” etc. etc. He noted that there was actually no Recommendation associated with rejection of names, but there was no reason why there should not be if it applied for those. He thought that it seemed a very reasonable wording, except that it was worded as a Recommendation.

**Greuter** did not think that it was possible, because we cannot recommend going against or disregarding the *Code*, and that would be the case here. He felt that it definitely had to be an Article, not with “should” but something like “Retention of the teleomorph-typified name is authorized, until such time as…”; or “The case should be submitted to the General Committee for consideration and, pending a decision by the appropriate Committee, the use of the teleomorph name is authorized”.

**Knapp** noted that it was definitely a friendly amendment but as it was quite a lot of typing she suggested that exactly the same thing might be done editorially, but “should” could be changed to “must” and that would have the same effect. [That was considered acceptable.]

**McNeill** added that it was recognized that it had to be changed editorially to parallel the normal practice in this matter.

**Knapp** clarified that this turned the proposal into a rule and not a Recommendation.

**Dorr** referred to Funk’s comment earlier that, until fairly recently, the *Code* had been fully self-contained. He thought the decision to introduce reference to ISSNs and ISBNs, which were outside our control, was acceptable. He added that in the Section the idea of ISO and other things outside our control had also been introduced. He did not think that references to other bodies such as the International Commission on the Taxonomy of Fungi should be put in. He felt that was a very dangerous thing and a violation of a principle that had been held for well over 100 years.

**McNeill** had overlooked that when he said the wording was acceptable and thought that it could be modified in exactly the same manner as the proposal accepted at the beginning had been modified.

**May** moved an amendment, which he hoped was friendly: to remove the text from “by the Committee for Fungi” onwards. [This was **accepted** as a **friendly amendment**.]

**Applequist** was confused, as it seemed to her that the existing Art. 59 had given precedence to teleomorph-typified names. She thought that the Section had just been asked to delete that and replace it with an Article that said teleomorph- and anamorph-typified names would compete against each other for priority. Now this paragraph said that whenever that proviso took effect it had to be run past a committee. She thought that this would presumably happen thousands of times.

**Hawksworth** noted that it only related to widely used [names] and it was not the intention that this would be done as a matter of course.

**Barrie** did not want to sound too chauvinistic, but pointed out that it should be approved by the General Committee, not the Committee for Fungi.

**McNeill** agreed.

**Greuter** thought it was really the wording. He had suggested earlier including the phrase “must not be taken up, but retention of the teleomorph-typified name is authorized”. He felt that continued use of the anamorph name, if it had priority, should not be precluded until there was a verdict that it was to be banned and that those that wanted to continue to use the teleomorph name should also not be penalized. He thought that was the idea.

**Hawksworth** agreed it was the correct idea but was not quite sure the wording was perfect. [Groans emanated from the audience.]

**Knapp** wondered if Hawksworth thought that the wording would be editorial.

**Hawksworth** promised to do his best to make sure it was.

[The **proposal** was **accepted** as amended to read:

“*57.2*. In pleomorphic fungi, in cases where, prior to 1 January 2013, both teleomorph-typified and anamorph-typified names were widely used for a taxon, an anamorph-typified name that has priority must not be taken up until retention of the teleomorph-typified name has been considered by the General Committee and rejected.”]

### Recommendation 59A

**Prop. A** (10: 69: 3: 3), **B** (9: 70: 2: 3) and **C** (13: 67: 2: 3) were ruled **rejected**.

[*A short discussion of Chapter VI Prop. A occurred here and has been moved to the normal order earlier in this Session.*]

### [Article 9 Proposal V]

[*This discussion of Art. 9 Prop. V, which also dealt with Art. 59, occurred here. Eventually the proposal was withdrawn, but to follow the logic of the discussion it is retained here as opposed to following the normal order and moving it to the Second Session on Monday afternoon.*]

**Hawksworth** thought that the proposal really should be kept, because there would be cases where it may be desirable to have a teleotype and perhaps an epitype as well.

**McNeill** introduced Art. 9 Prop. V, which was a definition of the term “teleotype”. He added that it was replacing the use of the concept of epitype for a situation in which a type was designated that showed no evidence of the sexual stage, but was treated as the type for a holomorph. This was a new term that was not the same as the traditional epitype; it was an extension of the meaning of epitype and it was deemed to be better to have a special term.

**Redhead** noted that part of the problem with designating an epitype that consisted of a teleomorph for a fungus that was originally described as an anamorph was that it precluded the designation of an epitype that actually matched it in morphology. There was always that element of doubt that it was being mismatched. He explained that was why they had refined it so that you could teleotypify a name, but you could still epitypify the anamorph with another anamorph for which, maybe, you had the genetic sequence. This allowed flexibility and was why they had coined the term. He pointed out that it was to pertain to Art. 59, which had now been modified, and reference to it had gone there, but it may still prove to be useful in typifying fungal names.

**May** commented that if Art. 59, as before, had been deleted, the point of the proposal seemed to have disappeared because this was in order to put a teleomorphic title onto an anamorphic name to then give that teleomorphtypified name priority. He asked Redhead, given that there was no priority difference between anamorphs and teleomorphs, under what circumstance would he consider utilizing this provision?

**Hawksworth** responded with the example that essentially, you may have an anamorph name that was already epitypified and then discover the sexual stage and want to link that, definitely, to a specimen that showed a teleomorph. There was a case he knew that was just about to be published for another common *Aspergillus* species that was in that category.

**McNeill** wanted to make sure that it was understood that this was subject to editorial modification, in the light of the deletion of Art. 59.

**Demoulin** was in favour of the proposal in the framework of the present Art. 59, or revised version, but with the quasi-deletion that had been voted for, he also failed to see the need for such a lengthy addition. He thought that it could be used for historical purpose, to discuss what had been done in the past, but questioned whether it was worthwhile putting that in the *Code*. He added that in the case to which Hawksworth referred, since there was no special priority anymore, he thought it could be dealt with by conservation eventually. He would not vote for the proposal.

**Lendemer** wondered how this would impact the passage of Art. 57.2, which had just been passed, because that was where teleomorph and anamorph were in the typified names and you were not supposed to take up the priorable anamorph-typified name. He was confused that this proposal would mean that if you teleotypified it you accorded it teleomorphic status and he thought that would have some kind of impact with Art. 57.2.

**Redhead** was inclined to think that the proposal was not necessary anymore, and was only weakly supporting his own proposal.

**McNeill** did not think it could possibly be included in its present wording and it would have to have an explanatory historical note, but as that had never been in the *Code* before, maybe even that was unnecessary. He was very dubious about its need and so was the proposer.

**Funk** wondered why we were talking about it. [Laughter.]

**Knapp** answered that was because the floor was still open and people were still raising their hands.

**Redhead** withdrew the proposal. [Applause.]

**Reveal** wanted to remind everyone who dealt with epitypes of the actual wording in Art. 9.7. An epitype may be selected only when it was demonstrably shown that the specimen was ambiguous and exact identification of the name could not be done. He was seeing more and more epitypes being proposed in literature without any justification or any indication that there was ambiguity at all with the specimen; rather, people were complaining about the state of the specimen. That’s tough. Epitypes do not correct this.

**McNeill** could not agree more and also received many papers with that.

**Knapp** suggested that everyone was quite tired, several pieces of very important business had been dealt with, and that we end for the day so it was possible to rest before the dinner. She reminded the Section of how much business remained to be dealt with in what was, essentially, a half a day tomorrow. She explained that was because there was an IAPT business meeting scheduled for the afternoon. She also reminded everyone to think about what they wished to say about the proposals that were still to be discussed and to make their remarks as brief as possible. She thanked everyone very much.

## Ninth session

Friday, 22nd July, 2011, 9:00-12:30

**Knapp** reminded the Section that by her calculations, 33 of the published proposals still remained to be dealt with as well as an untold number that may have been skipped over.

**Marhold** proposed that all the very valuable proposals concerning the Glossary that were mostly of an editorial nature be referred to the Editorial Committee. [This was deemed not the appropriate moment to discuss the issue and it was deferred until later.]

**McNeill** advised that the secretaries of the Permanent Nomenclature Committees of Algae, Fungi, *Bryophyta*, Fossil Plants and Vascular Plants should be ready to make a brief presentation towards the end of the meeting, a report on their activities over the period of the last six years.

[*Some business conducted during the Ninth Session was relevant to provisions of the Code dealt with earlier. The proceedings of the corresponding debates can be found under Art. 9 Prop. H in the Second Session on Monday afternoon; Art. 32 Prop. H and I in the Fourth Session on Tuesday afternoon; Art. 46 Prop. B-K in the Seventh Session on Thursday morning; and Rec. 46D, Art. 48, Art. 49, Art. 52, Art. 53, Art. 54, Art. 55, Art. 58 and Chapter VI in the Eighth Session on Thursday afternoon.*]

### Article 60

**Prop. A** (37: 60: 13: 0).

**McNeill** noted that the Section had arrived at Art. 60, an Article that had often generated more heat than light. [Laughter.]

**Knapp** commented that that was muttered under his breath.

**McNeill** maintained that it was quite audible. He went on to introduce Art. 60 Prop. A by Gandhi and Reveal, which had a significant negative vote: 37 in favour, 60 against.

**Gereau** felt that the principle expressed was largely correct but wellknown and that the etymology of the Example was unsure at best, as he had discussed in detail with the Vicerapporteur and the overall effect of the proposal was undesirable.

**Demoulin** concurred with Gereau, and added that the etymology was not doubtful but absolutely incorrect. He continued that the description implied that “hollow-seeded” was meant, which implied that the Greek *koilos* was meant and not at all the Latin *caelum*, which is “heaven”. He asserted that it was nothing to do with that and felt it was better to drop the proposal completely.

**Veldkamp** added the additional note that Blume in the *Bijdragen*, where this name was published, complained about all the spelling errors in it, and his excuse was that he was ill and did not see the proofs. [Laughter.]

**Prop. A** was **rejected**.

**Prop. B** (25: 73: 10: 0).

**McNeill** moved on to Prop. B, which was dealing with making clearer that the use of the diaeresis was permissible by inserting mention of it in a number of places. He reported that again, the mail vote was rather negative: 68% “no”.

**Greuter** felt that the problem with the diaeresis was that it was mistreated in the *Code*. He offered a proposal, which might be considered by the Chair as an amendment, although it was rather different in context. In the paragraph in which the diaeresis was mentioned, Art. 60.6, he suggested it should say that a diaeresis “is an optional phonetic device that is not considered to alter the spelling”. He felt that if it were put like that, it would not have to be dealt with in any other part of the *Code* where the diaeresis was mentioned.

**Knapp** suggested that was actually substantially different to the proposal in question.

**McNeill** thought a separate proposal would have to be made though it might be considered while dealing with Art. 60.

**Knapp** suggested the Section was ready to vote on Prop. B in order to make clear that the diaeresis was permissible and amend three Articles in Art. 60.

**Demoulin** thought that the diaeresis should be dealt with in the way Greuter proposed but also thought it was probably necessary to have it in real orthography. He proposed to amend the proposal by adding, after “permissible”, “and often useful”.

**Knapp** confirmed that this was in Art. 60.4: “The diaeresis was permissible too and often useful.”

**McNeill** queried whether this was in all three Articles or just the one, deciding just the one, as “permissible” only occurred once.

**Knapp** agreed it concerned only Art. 60.4. [The **amendment** was **seconded**.]

**May** spoke against the amendment because he would like to discourage the use of [the diaeresis], because in databases it was a real pain. [Laughter.] He continued that, given a lot of information about plant names was now held in databases, it was a real nuisance and he did not think it should be encouraged.

**Alvarado** thought that the use of the diaeresis was maybe useful in the past but nowadays was a bit redundant. He pointed out that it was permissible but it did not say that it needed to be done; you could either write them down or not if you were using a computer database. He felt that it was not saying that it was advisable, just “permissible and often useful”, which was not the same.

**McNeill** asked if he was speaking against the amendment.

**Knapp** asked if he was speaking for the amendment.

**Alvarado** responded “Hmm” and found it difficult to say… [Laughter.]

**Knapp** suggested that he was sitting on the fence. She moved to a vote on the amendment to add the words “and often useful” to Prop. B in Art. 60.4…” after the words “permissible too”. [The **amendment** was **rejected**.]

**Prop. B** was **rejected**.

**Greuter’s proposal**

**Knapp** used the Chair’s prerogative to take Greuter’s proposal at this point, since the Section was thinking about diaeresis and would probably forget it in 10 minutes. She added that the proposal would need four seconders and invited Greuter to repeat the proposal.

**Greuter** explained that in Art. 60.6 it should say “The diaeresis” and the words defining it: “is an optional phonetic device that is not considered to alter the spelling”. He added that this, incidentally, meant that for database purposes it need not be used and continued that it was useful in a published text, didactic in many cases—for instance, in school floras, just as there were floras that gave the tonal accent on generic names to tell…

**Knapp** interrupted to point out that he was starting discussion, then noted lots of seconders, so allowed him to talk about it. [Laughter.]

**Greuter** thanked her and apologized as he had thought that it had been seconded.

**Knapp** said it had not.

**Greuter** acknowledged that he was rushing ahead, and apologized [again].

**Knapp** accepted his apology. [And laughed.]

**Greuter** took up the discussion again, saying that in many school books—for instance, excursion floras used in schools—tonal accents were put on generic names to tell people that the names were pronounced *Clématis* and not *Clemátis* and *Ádonis* and not *Adónis* and a lot of things that common users would not easily know. In this context, he argued that the diaeresis was just a special case of this; nothing more, nothing less.

**Knapp** asked whether the proposal also suggested deleting the last bit of the Article.

**Greuter** replied in the negative; it was just the portion concerning the diaeresis.

**McNeill** asked for confirmation if this was from “The diaeresis” to the semicolon.

**Greuter** confirmed that, and added that it would make one of the following proposals redundant.

**Knapp** reiterated that the proposal was to just add those words to Art. 60.6.

**Turland** asked if Greuter was suggesting deleting the words between “indicating” and “permissible”.

**Greuter** was not.

**Knapp** was not quite clear on the proposal.

**McNeill** suggested that it meant replacement of everything there, except possibly the Example.

**Funk** [?] suggested this was editorial.

**Knapp** agreed that it could possibly be dealt with editorially.

**McNeill** agreed as long as the Section was clear what was being proposed, that it was replacing the present definition with a new one.

**Greuter** clarified that he did not read it out in full because he had it handwritten. It would read: “The diaeresis, indicating that the vowel is to be pronounced separately from the preceding vowel” and then, instead of “is permissible”, would come what he suggested.

**Knapp** confirmed that the words “is permissible” would be deleted.

**McNeill** agreed, but he thought Greuter was wanting to get all that didacticism out of the *Code*.

**Knapp** clarified that the proposal was to replace the words “is permissible” with the words “is an optional phonetic device that was not considered to alter the spelling”.

**Barrie** felt that it was necessary to have something that said permission in there, or else…

**Knapp** chastised him for talking without the microphone. [Laughter.]

**Barrie** was not arguing specifically to keep “is permissible”, and he thought it was editorial, but he felt that something was needed that made it follow, so that it was not merely a definition of a diaeresis, because the ligature thing said they were not to be used, so he felt that something was needed there that said a diaeresis could be used but it was all editorial. He highlighted that the main thing was clarifying that a diaeresis was not considered to alter spelling.

**Demoulin** wondered whether it would it be possible to have simply “and is thus permissible”. [This was considered a **friendly amendment**.]

**Greuter’s proposal** was **accepted**.

**Prop. C** (54: 33: 22: 0) and **D** (32: 12: 64: 0) were ruled referred to the **Editorial Committee**.

**Prop. E** (69: 17: 16: 0).

**McNeill** introduced Prop. E, which was to append a sentence to Art. 60.9, in relation to providing a rather different and hopefully more precise and more usable definition of when a hyphen was permissible.

**Turland** acknowledged Greuter for the idea, which was to provide a mechanical way of deciding in most cases whether an epithet should be hyphenated. The idea was to make it easier on taxonomists who may not be particularly into linguistics, and was intended as a helpful mechanism.

**Gereau** felt that the effect of the proposal was desirable but it needed rewording because it lead to some undesirable consequences. He gave the example that the epithet *saudiarabica* was still permitted under this revised wording but *saudiarabica* without the hyphen was also required, meaning it would have the effect of requiring two conflicting things. He added that the removal of the hyphen in *Eugenia costa-ricensis* in the current Ex. 20 was contrary to the current rule, because the words “Costa Rica” usually stand independently, so it needed some really serious editorial work not to have effects that were contrary to other rules.

**Greuter** was sorry that he had inspired the proposal. [Laughter.] Contrary to what the Rapporteurs appeared to imply, he thought it was not a case of adjective-adjective epithets only. There was presently an example in the *Code*, *Veronica anagallisaquatica*, which would be dehyphened if the proposal were accepted. There were a number of wellknown Linnaean-named epithets with hyphens, like *Cystopteris filixfragilis*, that were declinable adjectives that would be de-hyphened; not *filixmas*. He continued that this would lead to the funny situation that *Cystopteris filixfragilis* would have to be dehyphened and *Dryopteris filixmas* not. He did not think it was a mature proposal and was against it.

**Prop. E** was **rejected**.

**Prop. F** (78: 13: 17: 0) was ruled referred to the **Editorial Committee**.

**Prop. G** (57: 20: 33: 0).

**McNeill** moved on to Prop. G, which was to add a new rule following Art. 60.10. He added that it had quite good support in the mail vote.

**Van Rijckevorsel** noted that the proposal was on abbreviations in epithets and alluded to earlier in the week when Greuter had spoken on Linnaean names and abbreviations. He wished to raise a proposal from the floor at this point.

**Knapp** clarified that the current discussion was regarding Prop. G.

**Van Rijckevorsel** requested that the new proposal from the floor be dealt with immediately following this.

**Knapp** agreed.

**Van Rijckevorsel** thought that the proposal was intended to be as restrictive as possible and he knew only of cases of surnames that had a full stop, an abbreviation in them, and he thought they should be converted into epithets and be dealt with.

**Knapp** asked if there were any comments and discussion about that.

**Greuter** apologized and admitted that his continual desire to comment may be a bad habit. [Laughter.]

**Knapp** said that it was all right, “we’ll cure you in the end”. [Laughter.]

**Greuter** felt that what was really important, and should be in the *Code*, was that the many abbreviations that had a period in the name when published, including many Linnaean ones, not be considered not validly published because there was a period in the name or epithet, but that they be expanded as had always been done. He suggested to amend the proposal to say “Abbreviated names and epithets are to be expanded in conformity with botanical tradition”. [This was not accepted as a **friendly amendment** and the **amendment** was **seconded**.]

**Wiersema** suggested that if abbreviations were to be expanded in conformity with botanical tradition, that meant that in the Example under Prop. H the spelling would become *saintjohnianum* or *sancti-johnianum* due to Rec. 60C.5(d).

**Barrie** had a question for people who may be working with these things: were they putting these *St.-johnianum* type names in indices and databases or were they being spelled out? He was trying to ascertain which approach was going to cause the least amount of work to deal with.

**Knapp** asked if there were any indexers who would like to comment.

**Challis** felt that it was best to have the epithet as the author published it, unless there was an orthographical reason to correct it. She was concerned about whether this would create a lot of additional work, but did not know because she had not prepared anything.

**Gandhi** noted that he and Reveal had encountered a similar problem before the starting up of IPNI, when the Gray Cards existed individually at Harvard. In those cases, especially where the author was not surviving, they had taken the liberty of expanding “Saint” as *sancta* or *sancti* where it was appropriate.

**Knapp** summarized that the answer was that it was done both ways.

**Greuter** thought that there may be a few people in the room familiar with the *Species Plantarum* of Linnaeus. He felt that if the advice to adopt the epithets as published, perhaps dropping the period, was followed, that would lead to names such as “*Cystopteris F-fragilis*” and “*Hypericum androsaemifol*”. He thought that about onethird of all lengthy Linnaean epithets had been abbreviated in that way in the *Species Plantarum*, as was the usual procedure then. He suggested “in conformity with botanical tradition” because he argued that everyone knew how to expand these.

**Van Rijckevorsel** thought it would be useful to look quickly at the proposal he had made, because it dealt with just that point.

**Knapp** disagreed and thought the Section was going to need to vote on the amendment before moving to the new proposal.

**Van Rijckevorsel** insisted that just for information purposes, he would like to show the new proposal for background and information because it dealt with just that point. Not to be discussed or voted on but…

**McNeill** wondered if there was a problem in supporting the current proposal or if he was speaking against it and saying there was another one coming up.

**Van Rijckevorsel** reiterated that there was another proposal coming up that dealt with the point that Greuter was making.

**McNeill** clarified that he was asking if Van Rijckevorsel was speaking to the amendment or not.

**Van Rijckevorsel** was opposing the amendment on the grounds that he had a specific way to deal with the issue. He wanted to see it up on the screen so that people knew that the problem could be dealt with.

**Knapp** agreed to put it up.

**Barrie** just wanted to say, given Gandhi’s comments, that he was supporting Greuter on this, he thought it should be done.

**Kellermann** queried whether it was clear that the abbreviation would be expanded in Latin or would it be expanded in French if the person after whom it was named was French. He wondered if there was a problem there, that different people might expand the abbreviation differently if modern names were concerned as opposed the Linnaean names.

**Knapp** thought that the current discussion was on the amendment as it had been put. She did not want to develop proposals from the floor in a way that was going to take lots and lots of time, because there was a very limited amount of time in which to get through quite a lot of the rest of the business of the Section. She noted that the Section were seeing the new proposal on the screen, which may be germane to the acceptance or not of the amendment by Greuter. She moved to a vote for the amendment to Prop. G, which would add a sentence saying “abbreviated names and epithets were to be expanded in conformity with botanical tradition” [The **amendment** was **accepted**.]

**Knapp** moved to voting on the amended Prop. G.

**Prop. G** was **accepted** as amended.

**Veldkamp** had what he couched as a stupid question: how to expand “*st*” for St John? He thought that having accepted the previous proposal, it should be extended here in the Example.

**Knapp** felt that would be editorial.

**McNeill** thought that the answer was that botanical tradition did not expand it.

**Knapp** felt it was very ambiguous.

**Veldkamp** queried if it should be “*saint*”.

**McNeill** said no, because botanical tradition did not expand it, so it would be “*st*”. He clarified that what had been accepted was that abbreviations would be expanded in accordance with botanical tradition and if the botanical tradition was not to expand, they were not expanded. He assumed that was the intent of the amendment, adding that otherwise it would have been nonsensical.

**Wiersema** pointed out that, as he had indicated before, Rec. 60C.5(d), last sentence, recommended that these were expanded.

**McNeill** thought that it should be discussed as a separate issue as it was in fact a genuine Recommendation, not a back-door rule.

**Wiersema** agreed, but assumed it was based on botanical tradition. [Laughter.]

**McNeill** hoped so.

**Greuter** elucidated that what had been accepted said that in the case of personal and geographical names that contained a full stop, the full stop was treated as an error and to be removed. It followed that abbreviated names and epithets were to be expanded. He argued that if “St. John” was an abbreviation, it fell under the second clause and was to be expanded, adding that the Example now in the *Code* agreed. However, he argued that in other cases where it was just a period in a geographical or personal name it was to be omitted. He thought it was clear enough and suggested that when concrete examples were encountered they could be discussed individually but as a general rule it would work.

**Prop. H** (31: 16: 62: 0) was ruled referred to the **Editorial Committee**.

**McNeill** noted that Prop. H was a discrete Example involving *Nesoluma “St.-Johnianum*”, which would normally be referred directly to the Editorial Committee.

**Knapp** suggested that, rather than devising Examples by committee, we might, as a Section, consider empowering the Editorial Committee to make this make sense with Examples.

**Magill** thought that the Editorial Committee seemed to be biased in the other direction of what the Section wanted.

**McNeill** felt that the Editorial Committee, or some members of it, certainly did not mind at all on the matter and would be entirely willing to follow what seemed to be general botanical usage. He suspected that, like many of these situations, the recommendation in Rec. 60C was not always followed and that, in fact, practice was extremely diverse and it was probably necessary to reflect that in whatever was done. He added that those working in indexing and so forth would know that better than him. He interpreted that Wiersema was agreeing that things were diverse.

**Wiersema** thought that putting this Example in one way or the other would clarify the issue.

**Prop. I** (36: 33: 35: 0) was ruled referred to the **Editorial Committee**.

**Greuter** felt that Prop. I should be discussed in the context of Rec. 60C.

**Knapp** disagreed because the Section had voted that things having to do only with Examples would be referred to the Editorial Committee.

**Dorr** agreed with Greuter that the Example made no sense whatsoever unless Rec. 60C was removed and suggested postponing it until Rec. 60C was decided on.

**McNeill** reiterated the decision that all Examples would be referred to the Editorial Committee, which would act in accordance with the decision of the Section on the other proposal.

**Prop. J** (26: 57: 23: 0).

**McNeill** introduced Prop. J, which dealt with Rec. 60C. He noted that it had received a negative but not extraordinarily negative vote in the mail vote at 26 for, 57 against, 23 to the Editorial Committee. He read what the Rapporteurs said: “Prop. J makes a valid point that epithets which are unchanged personal names, rather than Latinized genitive or adjectival forms of a personal name, are against the tradition of botanical nomenclature (although apparently permitted under Art. 23.2) and … should be restricted to the nomenclature of cultivated plants… The proposer suggests such epithets are arguably disallowed under Art. 60.11, although if they are not given Latin terminations Rec. 60C.1 does not apply. In view of these issues, and so as not to encourage emulation of the epithets in Ex. 27 (‘*barbro*’ and ‘*jenny*’), deletion of the Example is proposed.” He added that the proposal also called for an introductory sentence to restore the wording in the *St Louis Code*. He summarized it as an attempt to prevent the appearance of epithets of that sort.

**Van Rijckevorsel** agreed that there were indeed two separate issues, which were closely linked. Firstly, Art. 60 Ex. 27 was an example of something that should not be followed. In that sense it is already undesirable. Secondly, there was the question of the introductory sentence of Rec. 60C.1. He would like it to be restrictive, modelled on Rec. 60B.1, which was quite unambiguous. He did not know how many names would be affected, and noted that that would have to be determined, but thought it was a limited number of names, guessing a few dozen. He felt that this would give great clarity.

**McNeill** queried as to whether Van Rijckevorsel was discussing the other proposal.

**Van Rijckevorsel** clarified that he was proposing that the introductory sentence of Rec. 60C.1 be modelled on Rec. 60B.1.

**Knapp** noted that the proposal was actually that it returned to the phrasing of the *St Louis Code*, which she did not happen to have with her.

**McNeill** thought it was “When personal names are given Latin terminations in order to form specific [and infraspecific] epithets, formation of those epithets is as follows”.

**Knapp** noted that that was in the *Vienna Code*.

**Van Rijckevorsel** thought that going back to the phrasing of the *St Louis Code* would not solve the problem.

**McNeill** suggested that, if it did not work, the Section could vote against it and another proposal could be made at a later point.

**Van Rijckevorsel** suggested that he could amend the one under discussion.

**McNeill** commented that the suggestion that Ex. 27 was there as an example to be followed was not the point, but rather it was there as an example of something that was indeed validly published under the *Code*.

**Knapp** thought that rather than continually amending things from the floor it was necessary to stick with what had actually been proposed.

**Challis** did not think that the example of an epithet “*jenny*” was a problem. She thought the epithet should be accepted as the author wanted to publish it unless it was orthographically incorrect.

**Knapp** asked if she was speaking against the proposal.

**Challis** was speaking against it and thought things should be left as they were.

**Gereau** also spoke strongly against the proposal. He argued that names used as nouns in apposition as epithets were uncommon but were scattered throughout botanical history and gave a few examples. When Léonard named *Ancistrocladus likoko*, he did it explicitly for a game guard named Likoko. When C. B. Clarke named *Cyperus ajax*, he had in mind the mythological person, Ajax. When Carl Luer named *Pleurothallis jupiter*, he didn’t call it “*jupiteri*”. He maintained that these instances were scattered; there was a tradition of them and there was no reason to regulate them.

**Prop. J** was **rejected**.

**Prop. K** (15: 49: 41: 2), **L** (62: 12: 31: 0), **M** (66: 6: 35: 0) and **N** (42: 8: 55: 0) were ruled referred to the **Editorial Committee**.

**Prud’homme van Reine** commented regarding Prop. L, that there was no reason to expect that Schmidt would honour only one of the two Gepps that were the specialists for *Codium* at that moment. He felt it was a good Example that appeared in the *Berlin Code* and had been followed by all phycologists working with tropical marine macroalgae, for *Codium geppiorum* was a very common species.

**Knapp** thanked him and pointed out that it had been referred to the Editorial Committee, not deleted. She was sure that the Editorial Committee would receive that advice from algologists and would act accordingly.

**Van Rijckevorsel**, regarding Prop. K, wished to convert the Example to a rule.

[*This was not accepted as an amendment but as a new proposal and there were no seconders so it was not discussed.*]

[*The following short comment, pertaining to a new proposal from Nagamasu and discussion of a new proposal from Davidse & Ulloa on Art. 60, took place during the Tenth Session on Friday afternoon.*]

**Nagamasu’s proposals**

**McNeill** introduced two proposals on Art. 60, the first was by Nagamasu and it related to Art. 60.1. The present wording was “The original spelling of a name or epithet was to be retained, except for the correction of typographical or orthographical errors and the standardizations…” etc. The proposal was to remove “or orthographical”. He added that it was not what it said [on the screen], but that was what it meant.

**Knapp** noted that there were not four seconders to the proposal; therefore the **proposal** was **not discussed**.

**McNeill** went on to the second of Nagamasu’s proposals, which was to delete Art. 60 voted Ex. 5.

**Knapp** noted that again there were not four seconders; therefore the **proposal** was **not discussed**.

**McNeill** added that there was a rider to the proposal that was addressed primarily to the Editorial Committee, which was to add some Examples of orthographic errors; and he thought that also need not be discussed.

**Davidse & Ulloa’s proposal**

**McNeill** introduced a new proposal from the floor by Davidse & Ulloa relating to Art. 60.6 regarding the use of the diaeresis, suggesting to change “is permissible” to “is to be discouraged”. [Laughter. The **motion** was **seconded** and **supported** by three others.]

**Demoulin** noted that there had been a proposal to encourage, now a proposal to discourage, but he thought a very good wording had been adopted that should close the discussion of diaeresis for good now. He would certainly vote “no” to the proposal.

**Davidse** outlined the proposers view that, as Greuter had eloquently explained, the diaeresis served a function for pronunciation and had nothing to do with nomenclature as such, and, as had also been discussed previously, it did still occasionally cause problems for people in databases. He was not suggesting outlawing it necessarily, because it had a long tradition, but at least discouraging it would be helpful.

**Greuter** felt that, as worded, the amendment had no place in an Article; it was clearly a Recommendation. “Discouraged” was a Recommendation wording, so if it was felt appropriate, which he did not think it should be, it would have to be proposed as a Recommendation separate from the Article. However, he did not see why it was necessary. Database people, having been told that this was a pronunciation device and not part of the orthography, could just leave it off, hopefully, in their own interest, because it was not part of the name, and those who for good practical reasons wanted to put it in should not be discouraged from doing so.

**Stevens** called the question. [There was a sufficient majority in favour of voting.]

**Davidse & Ulloa’s proposal** was **rejected**.

### Recommendation 60C

**Prop. A** (51: 29: 29: 0).

**McNeill** moved on to Rec. 60C Prop. A, which was to amend Rec. 60C.1 relating to the derivation of infraspecific epithets from acronyms. He noted that it had quite good support in the mail vote: 51 in favour, 29 against, 29 referring to the Editorial Committee.

**Greuter** referred to somewhere in the *Code*, he thought in the earlier Articles, where it said that an epithet could have any form whatever. Firstly, he argued that this proposal was trying to meddle with things—in a destabilizing way—that had never been questioned and he strongly opposed it. He felt that the proposal was to add acronyms where they had no place, epithets formed from personal names as acronyms were not personal names and he thought it already muddled the issue from the start.

Secondly, he added that *codesuri* had been generally accepted and done no harm so he wondered why this should not be allowable.

Thirdly, he happened to be a secretary of an organization known as OPTIMA, which was an acronym, and there were at least three species generally known and correctly named *optimae*. He pointed out that this would not be allowed under the proposal and thought it was ridiculous.

**Challis** agreed with Greuter, adding that he had brought up several points that she was going to make, only [he had done so] more cogently.

**Demoulin** had spent a lot of time on Art. 60 and agreed entirely with Greuter that this was absolutely unacceptable. He felt that there were already too many things that were supposed to be corrected by the back door and there was no reason at all to put acronyms in the Recommendation dealing with personal names.

**Prop. A** was **withdrawn**.

### Recommendation 60H

**Prop. A** (31: 68: 8: 0).

**McNeill** introduced Rec. 60H, which had received a heavy but not destructive “no” vote in the mail vote: 31 in favour, 68 against. The proposal was recommending greater use of the diaeresis.

**Gereau** was absolutely against the proposal. He agreed with everyone who had said that the diaeresis was a nuisance in databases and everywhere else, and thought that its use should be completely discouraged or disallowed, but acknowledged that was not on the floor.

**Kellermann** did not understand why databases could not cope with that in the names of plants because they have to cope with umlauts and accents in the names of authors.

**Wiersema**, as someone who was heavily involved in a database that would use such names and would have to insert this diaeresis, felt that this would create problems for sorting and searching because it would be necessary to search on two alternatives rather than one in order to find it.

**McNeill** noted that the Section had just decreed earlier in the morning that it was not part of the spelling of the name.

**Wiersema** thought it was preferable to discourage its use rather than encourage it.

**Alvarado** thought that the diaeresis was a problem because not all browsers supported all sorts of those alternative writings.

**Knapp** deemed that she was not hearing anything particularly different, so moved to a vote.

**Prop. A** was **rejected**.

### Recommendation 60I (new)

**Prop. A** (34: 52: 22: 0) was **withdrawn**.

### Article 61

**Prop. A** (10: 54: 40: 0) was ruled referred to the **Editorial Committee**.

**Prop. B** (9: 67: 33: 0).

**Gandhi** explained that in the Example given, initially the subgeneric name was spelled as *Petrophytum*, but when Rydberg elevated it to generic rank, he changed the ending to *Petrophyton*, which was quite a common procedure in those days, changing either an *um* ending to an *on* ending or an *on* ending to an *um* ending. This led to the problem of how to treat Rydberg’s generic name: as a status novus or as a new generic name? It involved not only IPNI but also ING. He reported that finally all involved had agreed, with discussion, to treat Rydberg’s generic name as a status novus. He believed in such cases having an Example would be quite useful.

**McNeill** noted that it had a quite heavy defeat in the mail vote. He suggested that it really did not particularly belong in this position and would need to be reworded and it could be referred to the Editorial Committee, as it was just a Note.

**Gereau** felt that the presentation of the Note was far too confused, beyond the remit of the Editorial Committee to clarify. He added that the entire meaning was already implicit in Art. 7.4, so felt there was no need for it whatsoever.

**Gandhi** asked if he could respond to that.

**Knapp** said he could not and that there was not going to be an argument about it, she was not going to let people get into discussion here on the floor and she moved to a vote.

**Prop. B** was **rejected**.

### Article 62

**Prop. A** (21: 60: 25: 0).

**McNeill** moved on to Art. 62 Prop. A, which was to delete provisions that had been in the *Code* for about 12 years, perhaps 18, establishing the names ending in *ites* as masculine. He elucidated that it was established particularly for fossil plant names, which generally were masculine, and it was to make clear that they all were, but there were one or two recent nonfossil plant names that were generally treated as feminine. He outlined that the proposers wanted to get rid of the provision although, in fact, the tradition of some being feminine was rather changing on account of the existence of this Article. He reported that the mail vote was rather negative: 21 in favour, 60 against, 25 referring to the Editorial Committee, although he noted that in the last case it would be a little difficult for the Editorial Committee to do anything.

**Christine Barker** gave the background that until 1975 established usage dictated the gender of generic names ending in *ites*, but a Recommendation came in in 1975 that these names should be treated as masculine. The Recommendation became an Article in 1988 but, as mentioned, the discussions were focused on the names of fossil taxa without much consideration of the effect on the names of extant plants. She noted that since the ruling had come in, it had been largely overlooked and littleapplied to the names of extant plants.

She argued that it was in conflict with current practice in a lot of cases as there were about 300 *ites* names, including some wellestablished and economically important ones that were treated as feminine and were documented extensively in the literature with feminine endings. For example: *Aleurites moluccana*; *Balanites aegyptiaca*, the desert date; *Odontites verna* and *Galactites tomentosa*, which were both common European taxa. She felt that enforcement of this overlooked ruling would be disruptive to the names of extant plants and acceptance of this proposal would allow established usage to dictate gender.

**Knapp** asked for comments and identified someone “who was a fossil person”. [Laughter.] She corrected herself to “a person interested in fossils”.

**Herendeen** wondered if she had called him a fossil. As he understood it, the proposal as written would cause significant disruption to fossil plant names, so if it were to be accepted an exception would be needed for fossil plants. He proposed an amendment that added “fossil plant names excepted”, or something to that effect, so that it did not apply to names of fossil plants. [This was accepted as a **friendly amendment**.]

**Demoulin** spoke in support of the proposal as he had never been happy with the rule and could not get *Rozites caperata* out of his mind.

**Greuter** agreed that the community had been slow to become aware of this rule. However, as the Rapporteurs correctly commented, in the last 10, 12 years it had been increasingly implemented. He noted that it had been adopted in floras, school floras and in gardens in labelling. He suggested that what we were now asked to do was to tell all those who had been applying the *Code*, hey, come back, bear the expenditure of making new labels, whereas those who had not been applying it were laughing. He did not think that was wise, he thought it inappropriate.

**McNeill** had thought it was actually 18 years but corrected that to 24 years since this was a rule. He thought that the Section should be well aware that this was a very long time for a rule to be in and then to be changed.

**Davidse** strongly endorsed what Greuter had said, adding that many databases had in fact changed the names and he hated this pingpong back and forth. He suggested sticking with the rule that was being implemented, although in some cases very slowly.

**Wiersema** pointed out that the remedy of conserving gender of any of those special cases that would be disrupted by this was still available so he thought we should stick with what had been established and fix those other cases through conservation.

It was just pointed out to **Herendeen** that the proposal had “fossil plants” and it also covered fossil algae and fungi, so the amendment would need to be modified so that it was not specifically about fossil plants.

**Knapp** thought that was editorial.

**Gandhi** thought that within the IPNI plant database several generic names had the masculine ending and some not and based on the conclusion from the Section, they would be changed accordingly.

**Christine Barker** wished to add that it was very easy to change the endings in the databases, but in the literature there were many instances of feminine endings. It was through following the *Code* and altering the endings in IPNI that this all came to light. She had encountered some backlash from Martin Sands, who published a monograph of *Balanites* in 2001. She explained that he had taken *Balanites* as feminine, so he was wondering why the endings had been changed in IPNI.

**McNeill** noted that the change was because she had, very sensibly, followed the *Code*.

**Kirk** felt that the effect of changing orthographies to the accumulated literature was nothing compared with the changes in taxonomy and misapplications, so it was a non-entity.

**Knapp** queried as to whether he was speaking for or against the proposal.

**Kirk** clarified that he was referring to the question that said that it would be a problem for incorrect orthographies in the literature.

**Knapp** thought that was more of a comment, and asked again whether he was speaking for or against the proposal.

**Kirk** was against the pingpong.

**Barrie** called the question. [There was a sufficient majority in favour of voting.]

**Prop. A** was **rejected**.

**Demoulin’s proposal**

**Demoulin** moved a proposal from the floor, a small amendment to Art. 62, that he thought in Australia would be considered an important one. He explained the background that Art. 62 had been introduced in Berlin as approximately the only thing that came out of the Orthographic Committee appointed in Sydney, which had generated tons of papers but a single proposal that made it. In the original proposal, Laurie Johnson, who was one of the most active and, he thought, rational members of that Committee, always insisted on treating the case of *Eucalyptus* and asserting that *Eucalyptus* should retain its feminine gender attributed by botanical tradition.

But this wording, which had unanimous support in Berlin, had been changed because Paul Silva had some personal ideas about what botanical tradition was and did not want to retain the Berlin wording. His proposal was to find some way to respect Laurie Johnson’s wish and emend the entry. He assured the Section that this would not change anything in practice, simply the way it was worded, as he felt that nobody understood the Article anymore. He suggested changing the wording of the phrase dealing with *Eucalyptus* to something like: “*Eucalyptus*, even if this is a relatively young tradition, has a botanical tradition that gives it, as the author originally did, the feminine gender”.

**Knapp** added that this was regarding Ex. 1.

**Demoulin** agreed, pointing out that it was a voted Example at the top of Art. 62. He noted that he had had to do a lot of research to understand how this ununderstandable thing had crept into the *Code*. He clarified the proposal to replace the words “*Eucalyptus* L’Hér., which lacks a botanical tradition” by “*Eucalyptus* L’Hér., which had a botanical tradition, even if limited in time”. He felt this was an important thing, as Laurie would never have accepted it.

**McNeill** noted that it might need editorial attention, but thought that the core amendment was clear. [The **proposal** was **seconded** and **supported** by three others.]

**Thiele** called the question.

**Knapp** deemed it necessary to have to have one bit of discussion.

**May** commented that in discussions about this when it came up in the Committee for Fungi, they were very confused about what “botanical tradition” meant because how can a name that was described around 1800 lack a tradition onwards from there? The confusion was about whether it was lacking a botanical tradition because it did not have a classical usage. He suggested that the Example did need to be reworded and thought it should be referred to the Editorial Committee to sort that out, because it was not just about the gender.

**Glen** felt that one could, of course, say that *Eucalyptus*, being feminine, was in a very much longer botanical tradition, thinking of all the classical names of mainly Mediterranean trees that were masculine in form and feminine in gender. He had no problem with *Eucalyptus* being feminine.

**Funk** called the question. [There was a sufficient majority in favour of voting.]

**Knapp** asked all those in favour of voting, to please raise their hands and all those against voting at this time, to please not raise their hands. [Laughter. Someone’s mobile telephone rang]

**Knapp** asked all those in favour of answering the phone… [Laughter.] She moved to a vote on changing the wording of Art. 62 voted Ex. 1 to be slightly nicer to the Australians… [Laughter.]

**McNeill** suggested it was to be slightly nicer to botanical tradition.

**Demoulin’s proposal** was referred to the **Editorial Committee**.

## Tenth session

Friday, 22nd July, 2011, 13:30–16:30

**McNeill**, before starting, emphasized that if people had proposals that they were planning to make in the “other business” section to make sure they had given him a piece of paper saying what it addressed, in order to order them sensibly.

**Funk** had two Special Committees she wished to propose that related to Division III, so she thought this was an appropriate place. The first of these would be a Special Committee to compose by-laws for the Nomenclature Section and the second one would be one to examine institutional votes.

**Knapp** thought they should probably be taken as separate proposals. The first would be a proposal to establish a Special Committee to write by-laws for the Nomenclature Section. She explained that by-laws were the rules on voting that governed meetings. [The **motion** was **seconded** and **supported** by three others.]

**Barrie** noted that there had never actually been any written procedures before; there had always been these traditions as set down. He thought that this time the Bureau had done an excellent job of explaining exactly what was going to happen at the beginning, so everyone knew what the procedures would be. However, he also thought it would be a good idea to look at the procedures and see if something could be put down in writing so everyone would know beforehand what was going on. He added that there were several proposals coming up that would also address the issue. He would much prefer to go to a Committee than have those proposals passed, so he was in support of the Committee.

**Knapp** moved to a vote on whether the Section would like to have a Special Committee, established to write down procedures in a place where everyone could look at them at one time, which was to draft by-laws. She noted that it was a procedural vote so it required a 50% majority, because it was not a proposal to change the *Code*.

A new **Special Committee** was established [the Special Committee on By-laws for the Nomenclature Section].

**Knapp** passed to the other proposal, which was to establish a Special Committee on Institutional Votes. [The **motion** was **seconded** and **supported** by three others.] She invited Funk to say a little bit about what the Committee might do.

**Funk** outlined that there had been a lot of discussion over the last three or four years about how institutional votes were assigned: do we need that large of a number for anybody; what were the criteria that should be used? It seemed to her that it would be a really good time to set up a Committee where anybody who was interested in being considered to be on those Committees would be able to sign up offering a very open format where people could bring their ideas to the table and the Sections might reformat how the issue was dealt with.

**Sebsebe Demissew** thought it was a very good idea and that it was time that the institutional voting system was revised. He thanked Funk for raising the issue.

**Knapp** added that at the end of the meeting she would highlight the Special Committees for which there were signup sheets, and people would be invited to come down and sign up.

A new **Special Committee** was established [the Special Committee on Institutional Votes].

### Division III

**Prop. A** (77: 6: 5: 11).

**McNeill** proceeded with the first proposal in Division III from Stotler and Isoviita. He reported that they were proposing that the Committee for *Bryophyta* change its name to the Committee for Bryophytes. He explained that *Bryophyta* taken these days apparently applied to what otherwise would be called *Musci*, whereas the *Marchantiophyta* were the liverworts, but apparently the English word “bryophytes” had not narrowed its scope in the same way as *Bryophyta* had, so it would be a welcome change, of which the Committee for *Bryophyta*, which would be now Bryophytes, was unanimous in support.

**Prop. A** was **accepted**.

**Herendeen’s proposal**

**McNeill** decided to deal with one of the proposals from the Floor at the same time, because it was so cognate, and that was that there was a proposal that the Committee for Fossil Plants change its name to the Committee on Fossils.

**Herendeen** explained that for a variety of reasons the Committee found it necessary to change the name. He added that there had been consultation among the members of the Committee here and by e-mail and all were in agreement that Committee on Fossils was the most succinct appropriate name for the Committee.

**Knapp** asked for four seconders and noted great support for “our fossil”. [Laughter. The **proposal** was **seconded** and **supported** by three others.]

**Herendeen’s proposal** was **accepted**.

**Prop. B** (93: 14: 2: 0).

**Knapp** moved to Prop. B, which was a proposal from Landrum to insert a new paragraph under the first paragraph that “Changes in the *Code* require 60% or higher positive vote of the Nomenclature Section of the International Botanical Congress”. She suggested that in the light of the fact that a Committee had just been set up to address the issue, it would seem wise that it be referred to that Special Committee.

**Prop. B** was referred to the new **Special Committee** on By-laws for the Nomenclature Section.

**Prop. C** (81: 28: 1: 0).

**McNeill** commented that the second proposal was a similar procedural issue, that approval of actions by Committees as recommended by the General Committee would require a 60% or higher positive vote in the Nomenclature Section of the International Botanical Congress, which again would seem as though it should go to the Committee on By-laws.

**Prop. C** was referred to the new **Special Committee** on By-laws for the Nomenclature Section.

**Prop. D** (18: 85: 2: 1) was ruled **rejected**.

**Landrum’s proposal**

**McNeill** noted that there had been a more than 75% majority “no” vote on Prop. D, but that there was an amendment that the proposer had submitted even though he was not present. He thought that this would be the time to take the amended version. He explained that it was dealing with who was entitled to vote at a Section, and thought it might be that it should be referred to the Special Committee on Institutional votes.

**Lewis** said that Les Landrum had asked him to put in the amendment to his own proposal so that it was on the floor, but given what had just taken place he was sure the proposer would be happy for this also to be passed to the new Special Committee.

**Buck** queried as to whether, if the Section voted “no”, the proposal was just killed; he wanted to know if “yes” sent it to Special Committee, “no” killed it.

**Knapp** presumed that the Special Committee would probably consider the issue anyway.

**Buck** wondered what a “no” vote meant then.

**Knapp** suggested that a “no” vote meant you didn’t care. [Laughter.]

**Demoulin** thought it should mean that you did not want that proposal.

**Knapp** went on that a “no” vote meant that it should not be passed to the Special Committee.

**McNeill** added that the proposal was then dead… or approved here.

**Knapp** continued that a “yes” vote meant the Section would like the proposal, although it had been defeated in the mail vote…

**McNeill** realized that the proposal being discussed was something other than what was heavily defeated in the mail vote, as it was apparently a new version replacing the one that was defeated in the mail vote.

**Knapp** thought that it was substantially different from the proposal that Landrum had put in, so suggested that four seconders were needed to put this from the floor.

**Turland** felt it was not really relevant to the Special Committee.

[The **proposal** was **seconded** and **supported** by three others.]

**Knapp** clarified that the discussion was not about the proposal that was defeated in the mail vote, but about a new proposal on Div.III.4(b), that the final vote at the Nomenclature Section would be by all officially enrolled members and that the Nomenclature Section would be conducted over the World Wide Web.

**Barkworth** thought that this would also need to go to the Special Committee, the one on by-laws rather than the one on institutional votes.

**Greuter** wondered if anyone could tell what “the final vote” meant in this context.

**Knapp** felt obliged to say, as the proposer was not present, that it was a question that was not answerable.

**Demoulin** commented that there had been a proposal heavily defeated by the mail vote not to have any more institutional votes. He felt that was a very important issue and it was now being replaced by a quite different proposal dealing with introducing virtual meetings. He thought it was certainly totally appropriate to refer the new concept to a Special Committee, but he thought it also important to rule that the proposal deleting the traditional votes was rejected.

**Knapp** pointed out that it *was* rejected by the mail vote.

**McNeill** clarified that that portion of it was rejected but there was an addition to try to make it more palatable by having electronic participation. He went on to say that the one that was defeated had been defeated and could be forgotten. The new proposal incorporated part of that [defeated proposal] but added an additional component relating to electronic participation.

**Prud’homme van Reine** confirmed that the Section had the right to say “no” to this as well.

**Knapp** agreed.

**McNeill** confirmed there would be a chance.

**Knapp** asked if there were any objections to voting to send the proposal to a Special Committee.

**McNeill** pointed out that he thought some people wanted to actually reject it outright.

**Knapp** explained that if there were no objections to voting to send it to a Special Committee a “yes” vote would mean the proposal would go to the Special Committee from this Section, as commended by this Section. A “no” vote would mean the Section did not commend the proposal and would prefer not to send it to the new Special Committee, but it would be a mandate from the Section as to which way the Special Committee should act.

**Thiele** was unclear, because in the previous votes to send proposals to a Committee he did not regard that as an endorsement of those proposals to the Committee.

**Knapp** concluded that it would not be considered an endorsement, it would be that the proposal would be sent to the Committee or not.

**Thiele** suggested that if the majority voted “no” to sending it to the Committee, then the proposal should be voted on.

**Knapp** thought that would be perfectly in order, and thanked him for the suggestion. [There was a simple majority against sending the proposal to the Committee.]

**Landrum’s proposal** was **rejected**.

**Prop. E** (35: 54: 12: 4) was referred to the new **Special Committee** on By-laws for the Nomenclature Section.

**Prop. F** (57: 36: 6: 5).

**McNeill** moved on to Div. III Prop. F, by Hawksworth & al., which was to amend Div.III.2 to provide for the election of the Permanent Nomenclature Committee for Fungi by an International Mycological Congress as opposed to by an International Botanical Congress. He reported that this had been considered by the Committee for Fungi and received a 71% “yes” vote: 10 in favour and 4 against. It also was supported in the mail vote 57 to 36.

**Applequist** asked the proposers if this was really how they wanted to do business, why were they here? She wondered, once the title of the *Code* had been changed and all the provisions put into it that the mycologists wanted and the botanists did not, what reason there was to believe that “we were ever going to see any of you guys again”?

**McNeill** pointed out that the discussion was solely about the way in which the Committee was established, not discussing where decisions on mycological matters come; that followed in Prop. G and H, not in Prop. F.

**Demoulin** had a general suggestion concerning the three proposals on mycological nomenclature. He thought that they should be reconsidered in the light of what had already been decided at the Congress. He thought that the change in title of the *Code* should lead to a reconsideration of how to handle the governance of mycological nomenclature. He suggested that the IAPT meeting next might consider this, since it was now a *Code* for three equally ranked groups and the Nomenclature Section was something that was really independent from the Congress. For plants there were the classical [Botanical] Congresses, for fungi the Mycological Congresses and for algae the Phycological Congresses. In his opinion the Nomenclature Section should be associated with something else in the framework of a BioCode, but at the moment, given the title change, he thought it may change the opinion of some of the fundamentalist mycologists if the Nomenclature Sections would take turns between the three congresses. He offered this thought for the proposers and the IAPT people, but at the moment he considered the proposals premature and would vote against them.

**Hawksworth** thought one way forward for the three proposals might be to have a Special Committee. He proposed a Special Committee on the Governance of Mycological Nomenclature.

**McNeill** asked if he would want it to be as broad as Demoulin had suggested, which would involve examining the authority of the *Code*, in other words, permitting a different Congress to ratify changes.

**Hawksworth** felt that if it was the wish of the Section that it was broadened out, it could be part of a bigger exploration.

**McNeill** clarified that the Special Committee would just be concerned with the mycological component, not phycological and other things.

**Hawksworth** confirmed that was correct.

**Knapp** summarized that a Special Committee had been proposed on the governance of mycological nomenclature. [The **motion** was **seconded** and **supported** by three others.]

**Gereau** felt that if part of the mandate of such a Special Committee was to be to consider mechanisms by which Mycological Congresses could effect changes in the common *Code*, then the answer was absolutely not.

**Greuter** suggested that it should be a subcommittee of the Committee just appointed, so as not to have completely unrelated procedures drawn up and to force them to keep in contact. [This was **accepted** as a **friendly amendment**.]

**Knapp** clarified that it had been amended to be a subcommittee on governance of mycological nomenclature, which would be reporting to the Special Committee on By-laws for the Nomenclature Section.

A new **Subcommittee** of a **Special Committee** was established [the Subcommittee on Governance of the *Code* With Respect to Fungi, to report to the new Special Committee on By-laws for the Nomenclature Section].

**Prop. F, G** (27: 64: 3: 6) and **H** (28: 61: 3: 7) were **withdrawn** on the understanding that they would be considered by the new Subcommittee on Governance of the *Code* With Respect to Fungi, within the Special Committee on By-laws for the Nomenclature Section.

### Article H.1

**Prop. A** (20: 73: 7: 2) was **withdrawn**.

### Article H.2

**Prop. A** (19: 72: 8: 1) was **withdrawn**.

### Article H.6

**Prop. A** (18: 69: 11: 2) was **withdrawn**.

**McNeill** noted that the three proposals on the hybrid Appendix had been withdrawn as they had substantial negative votes in the mail vote and the proposer did not think it was good use of our time to fight a losing battle.

**Knapp** and **McNeill** thanked him for that.

### Appendix III

**Prop. A** (45: 7: 53: 0) was referred to the **Editorial Committee**.

**Prop. B** (54: 6: 43: 0).

**McNeill** introduced Prop. B and C, which were quite independent of anything considered before and added some new terms and abbreviations, because there were a number of generic names of which the gender was conserved and there was a suggestion that that should be indicated in the Appendices. He reported that Prop. B had good support in the mail vote.

**Greuter** noted that “gend.” was not a Latin abbreviation. He moved an amendment to say “gen. masc.” or “gen. fem.” or “gen. neut.” [followed by] “cons.”

**Knapp** thought that was probably editorial.

**McNeill** agreed, adding that it was very important, as it was not conserving the gender, but conserving the gender of the name.

**Prop. B** was **accepted**.

**Prop. C** (52: 5: 46: 0).

**McNeill** indicated that Prop. C was suggesting explaining in the introduction to App. III, as opposed to simply in the Glossary, what nomen illegitimum, “nom. illeg.”, meant. The proposal was to add another sentence to the introduction of App. III, explaining what a nomen illegitimum was.

**Demoulin** was not sure that it was correctly worded and suggested referring that to the Editorial Committee.

**McNeill** agreed that the wording could be modified, but it was a matter of the principle of whether it should be included there or whether it was deemed sufficient to have it in the Glossary.

**Prop. C** was **accepted**.

**Prop. D** (30: 18: 57: 0).

**McNeill** considered that App. III Prop. D and E were editorial consequences of Art. 14 Prop. A, which had been accepted.

**Perry** questioned if the proposals were really editorial.

**McNeill** acknowledged what she was saying and concluded that it was implicit but not explicit.

**Perry** wondered whether they really were editorial, because it involved retroactively conserving names.

**McNeill** clarified that it made clear that the decision taken on Art. 14 Prop. A applied to existing conserved names as well as to ones in the future, which, as the *Code* was generally retroactive, might be presumed, but this made it very explicit. He added that it would be, incidentally, extremely valuable to have because there were names in there for which, without such provision, conservation would be useless.

**Prop. D** was **accepted**.

**Prop. E** (31: 18: 56: 0) was **accepted**.

### Appendix IV

**Prop. A** (52: 2: 45: 0) was **accepted**.

### Appendix VII

**McNeill** noted that earlier in the day there had been a proposal to refer all the proposals in App. VII to the Editorial Committee. He wondered if that person still wanted to make that proposal.

**Barrie** was not sure if McNeill was referring to him, but he would certainly like to see all of the proposals referred to the Editorial Committee en masse. [The **motion** was **seconded** and **supported** by many more than three others. Laughter at the large numbers of seconders.]

**Buck** was in favour of this but wanted to make an amendment to exclude Prop. Q from that, because he thought it was actually erroneous: “monotypic genus … for … a single binomial is validly published”. He argued that there could be 20 synonyms, with many validly published names, but there was only one taxonomically accepted species in the genus; that was typically what it meant by monotypic. As an editor and as an author he had changed that to monospecific because it was obviously not talking about one type.

**Knapp** was sure that the Editorial Committee would take that in mind. She also pointed out that suggesting that the proposals were all referred to the Editorial Committee did not mean they were boring or useless. It meant that they were something that was very useful to have in the *Code* but the Section thought that they could be dealt with by an Editorial Committee.

**McNeill** responded to Buck’s comment by saying that the Editorial Committee had been, and would continue to be, very careful with the previous editions of the Glossary that there was a precise concordance between the wording of the *Code* with regard to that term and the wording of the Glossary. Even if a word had wide parlance in the particular sense in taxonomy, if that was not how the *Code* used it, that was not how it would appear in the Glossary.

**Prop. A** (10: 12: 87: 0), **B** (17: 5: 88: 0), **C** (7: 17: 87: 0), **D** (13: 1: 94: 0), **E** (6: 14: 89: 0), **F** (11: 10: 89: 0), **G** (5: 16: 89: 0), **H** (14: 0: 95: 0), **I** (8: 12: 89: 0), **J** (20: 0: 89: 0), **K** (10: 10: 89: 0), **L** (8: 11: 91: 0), **M** (6: 13: 91: 0), **N** (11: 9: 89: 0), **O** (20: 0: 88: 0), **P** (12: 9: 87: 0), **Q** (7: 9: 93: 0), **R** (7: 11: 91: 0), **S** (12: 1: 96: 0), **T** (7: 1: 101: 0), **U** (10: 10: 89: 0), **V** (7: 11: 91: 0), **W** (8: 12: 88: 0), **X** (7: 6: 97: 0), **Y** (9: 11: 88: 0), **Z** (9: 10: 90: 0), **AA** (16: 37: 50: 0) and **BB** (13: 32: 55: 0) were all referred to the **Editorial Committee**.

### Other proposals
Sanctiotypification debate

[*The following discussion about the set of new proposals relating to sanctiotypification took place during the Sixth Session on Wednesday afternoon.*]

**McNeill** noted that consideration of a series of proposals dealing with the typification of sanctioned names had been deferred. He reported that there had been meetings discussing the nature of the current Art. 7.8 on typification of sanctioned names and there was an agreement.

**Demoulin** was the chairman of the Committee for Fungi and considered himself one of the few persons who had followed the whole story since its beginning. He offered some background to the issue and would then let one of the main proposers, Redhead, explain the details of what was planned—he emphasised that it would not be too much detail.

At the Sydney Congress there was a major event for fungal nomenclature, which was to delete the later startingpoint. The proposal itself did not deal with typification, but the people who met informally at that time, including Hawksworth, Demoulin and Korf, felt that the issue should be addressed because otherwise too much opposition for the new proposal would come from people like Rolf Singer, who had been used to typifying agaric names according to Fries’s volume one of the *Systema*, with an 1821 startingpoint.

Thus Korf raised a proposal from the floor that was supposed to be a compromise between those like Singer, who had been typifying according to Fries 1821, and people like Donk, who always considered that the main emphasis should be to the real original author. It was supposed to be a gentlemen’s agreement that, with the new system, as little as possible of what had been done before would be changed. That is, if agaricologists had been typifying according to Fries, that typification would stay. For other mycologists, those [names] for which there was a special status of protection if they had been accepted in volumes two and three of the *Systema*, but nothing about typifications of names were validly published somewhere else, and so they were not typified with any inference of Fries, they would keep their types as they were and everybody could keep doing things the way they were used to doing them.

But the wording was rather vague; it was allowing everybody to do whatever they wanted. There had been some attempt to make it more accurate for the next Congress in Berlin, with a lot of discussion inside the fungal Committee [the then Committee for Fungi and Lichens], but because there were those two schools that went back to the ’50s of typifying according to original authors or Fries, no consensus was reached and Singer was still present at the Berlin Congress. So there was no agreement and no major change came from the Committee. The Editorial Committee did some modification but of course not major modification.

Then for 20 years things did not move until this winter, when independently Perry, as somebody who was very accurate in trying to find things in the *Code* that should be straightened up, saw that it was not possible to keep this rather vague rule and made proposals. And, on the other hand, a group of mycologists led by Redhead also made proposals and eventually alternative proposals. The result was that there are three alternative proposals for the main vote in some issues. None was accepted. Some were rather heavily rejected. Others were moderately rejected but with a possibility to take up discussion.

Since all proposers were present, as well as people who knew the whole story, such as Hawksworth and himself, and they had strong objections to some of the aspects of the published proposals, it was decided to try to find out if it was possible to come to an agreement. He added that Singer was dead and Korf no longer attended Congresses, so many people had forgotten what it was all about and much more precise instruction was necessary.

He felt that there should still remain some gentlemen’s agreement that introducing the term sanctiotype should not be considered as encouraging using this possibility for things where it had never been used. That is, ascomycete names in volumes two and three of the *Systema*, for example. He was sorry about that, as he would have liked to be able to do with the vocabulary available, but there seemed no way out, and while he felt the introduction of a new word—sanctiotype—was unfortunate, he believed it was necessary for the situation.

**Redhead** thanked Demoulin for the background. He went on to add, for those who were not familiar with fungal taxonomy and history, that the existence of the proposals went back to the fact that the starting date for fungi was not 1753. It used to be 1821 with Fries’s *Systema*, for certain fungi, and Persoon’s *Synopsis* in 1801. The *Systema* spanned several years, which was a problem, and at one point the dates were artificially moved forward to 1821. Then the span of years was allowed, and then it was finally decided that the startingpoint for fungi would go back to 1753. To stabilize the decisions made in nomenclature in fungal taxonomy, it was decided that the names in these two original starting-point publications would be sanctioned names. Then there were questions on how to deal with typification and existing typification. So there was an Article, Art. 15, on sanctioning and there was also an Article on the typification in the sanctioning works. These, for the most part, affect only fungal names.

So the wording of Art. 7.8 was a bit vague in places and it allowed for dual interpretation. Norvell and Pennycook and himself had published a series of proposals to modify the Article. Perry had published another set of alternative proposals and he and his colleagues had also published an alternative set of proposals. One was to just delete Art. 7.8 and one was to modify it with the introduction of a new type of term. It was pointed out to them by the Rapporteur that they had all actually incorrectly attributed certain generic features to the Articles on species and vice versa. So they had got together to try to rework that with Perry and others like Demoulin and Hawksworth, and they had revised what had been submitted and the formal proposals.

The upshot was that they were withdrawing all of the original proposals and replacing them with a new set of proposals. Should they be approved they intended to refer all the accompanying Examples, some of which were independent proposals, to the Editorial Committee.

So the first part was the typification of names in the sanctioned works, and he outlined that they had come up with a particular wording, which allowed mycologists to typify things in sanctioning works that may include elements that were not in the protologues of the names that were sanctioned. They had run into problems in doing so in that there was a conflict with the definition of protologue and original material. Typifications that were allowed via the sanctioning works could not be lectotypes. They had had to come up with a new term, and arrived at sanctiotype—it was either sanctotype or sanctiotype—so they had defined that in Art. 9, and they had added various notes to further define that.

Then they had had to get to the level of genera and subdivisions [of genera] and had moved to Art. 10 to cover those situations. They had defined them in such a way that they thought it would work better than it was now and they had reached agreement.

He did not think there was any dissent amongst the mycologists and did not think Perry was against it, so they presented it as a kind of a package to the Section and he threw the floor open for discussion.

**McNeill** commented that in the process of editorial and Congress clarification of Articles, Art. 7.8 moved to a situation in which its only reasonable interpretation was that typification was entirely from the sanctioned work—actually, as Demoulin put it, the Singer position—which was clearly unacceptable to the broad mycological community. He noted that the key proposal that Redhead was making was these changes to Art. 7.8. and, apart from the introduction of a new term, everything else fell from that. He suggested that if there was any discussion, he imagined it should concentrate on that.

**Redhead** thought for procedural purposes—because it was a substantive change—it should be put forward as substitutes and would require the relevant number of seconders. [The **motions** were **seconded** and **supported** by three others.]

**Buck** was sorry but, since it was just proposed and he had not had a chance to see it all, he had a quick question based on what he had read. It seemed as if you could have both the sanctiotype and a lectotype for a single species. He wanted to know, if you did have both and they turned out to be different, did one have precedence over the other? He suggested it would be nice to print the new proposals out and distribute them so the Section could read it and digest it overnight.

**Demoulin** agreed that it would be necessary that people could read everything.

**Nic Lughadha** thought that all of the non-mycologists were disposed to try and help mycologists to the extent that they could, but that they did need to at least have an attempt to understand, and to do that they did need to be able to read all the proposals, and not like that [i.e. only on the screen]. She noted that the session was near a tea break and asked if it was possible to get it printed so that it could be looked at. It was very difficult to just say “Yes, this is all fine because the mycologists say so”.

[*It was decided that copies would be made to enable the Section to read and consider the new proposals. Here the record reverts to the normal sequence of events.*]

**McNeill** returned to the one major piece of published business that was withdrawn and replaced with a new set of proposals, and he understood Lorelei Norvell was going to speak to that. These were the proposals on the typification of sanctioned names, which had had much discussion by core groups of people over the last few days.

**Norvell** introduced herself. [Computer buzzing sound.]

**Knapp** concluded that it meant that she had to answer the question now! [Buzzer.] Now!

**Norvell** [Laughing while being interrupted by repeated buzzing and with the screen still blank] suggested just hooking up her Mac while the proposals were being searched for, because they were on that.

**Knapp** solved the issue of buzzing and blank screen by wiggling a cord.

**Norvell’s set of proposals**

**Norvell** outlined what the group that had proposed the so-called sanctiotypification proposals were withdrawing or addressing. She noted that the new set of proposals would affect Art. 7 Prop. H–I, Art. 9 Prop. J–M, Art. 10 Prop. C and Art. 15 Prop. A–C. She confirmed that Perry was also going to withdraw Art. 7 Prop. J and Art. 9 Prop. I. All of those proposals would be replaced by the new package displayed on the board. The people who worked on the solution were: Redhead, Pennycook, herself, Perry, Greuter, Demoulin and Hawksworth.

She reported that essentially what they were proposing to do was eliminate Art. 7.8, reword it and insert it after Art. 8.1 as Art. 8.1*bis*: “The type of the name of a species or infraspecific taxon adopted in one of the works specified in Art. 13.1(d) and thereby sanctioned (Art. 15), may be selected from among the elements associated with the name in the protologue and/or the sanctioning treatment.”

She planned to go through the whole package and then it could be taken either en masse or individually depending on the wishes of the Section.

She moved on to the second item to add a sentence to Art. 9.2, which would read: “For sanctioned names, a lectotype may be selected from among elements associated with either or both the protologue and the sanctioning treatment.”

She moved down to subgeneric names, where the proposal was to remove [Alternative 1], as it was for their purposes and she had forgotten to take it out. She outlined that essentially what was being proposed was to amend Art. 10.2, such that a colon and “*(a)*” would be inserted after “unless” about four lines down, and then after a semicolon the following would be added: “*(b)* the name was sanctioned, in which case the type may also be chosen from among the types of species names included in the sanctioning treatment”. The comment “or associated with a name in a sanctioning treatment” would be appended at the end of that, which she noted may be somewhat redundant.

She went on to the next amendment to Art. 10.5, to which would be added “The author who first designates a type of a name of a genus or subdivision of a genus must be followed, but the choice may be superseded if *(a)* it can be shown that it is in serious conflict with the protologue (or, for a sanctioned name, typified under Art. 8.1*bis*, with the sanctioning treatment)”—the new part that had been introduced—“and another element was available which is not in conflict with the protologue (or sanctioning treatment)”.

They proposed to add a new Article, Art. 48.1*bis*: “Where a sanctioning author accepted an earlier name but did not include, even implicitly, any element associated with its protologue, or when the protologue did not include the subsequently designated type of the sanctioned name, the sanctioning author is considered to have created a later homonym, treated as conserved under Art. 15.1”.

She explained that the reason they were doing this was that, as the Section knew, there was a change in starting dates, and up until that time mycologists were referring to the works of Elias Fries, and the starting date there would have been 1821, and things not covered by Fries—rusts, smuts and *Gasteromycetes*—were then also referred to Persoon, an 1801 publication. When the starting date was thrown back to 1753, mycologists had to ferret out all of the original works that those two authors had cited and it caused a fair amount of a kerfuffle—it affected about 4500 names, so it was no minor thing.

She assured the Section that mycologists really did deal with this daily and were looking for a way to make it very clear how to lectotypify. She added that the proposals were called the sanctiotypification proposals because we were going to introduce a new term called sanctiotype. She had noticed that there was a decided abhorrence of new terms in the Section and so they thought perhaps it would be kinder to everybody to work within the confines of the existing *Code* and that was what the document represented.

**Knapp** thanked her. [Applause.]

**McNeill** asked to return to Art. 10.5, because he thought there was a slight problem with the wording as the portion “and another element was available…” was no longer in the Article—it had been deleted earlier in the week.

**Norvell** proffered a tendency to regard an awful lot of amendments as friendly at this point.

**McNeill** noted it was not friendly, it was just a fact as it was not possible to amend something that was not in the *Code*.

**Norvell** thought that was very friendly! [Laughing.]

**Knapp** suggested it was not quite as friendly as proposing it yourself, though. She opened the floor for comment. [The **set of proposals** was **seconded** and **supported** by three others.]

**Gereau** had been unable to understand regarding sanctiotypes why, if there was no original material associated with the protologue available for lectotypification that was not in conflict with the protologue, one would not simply choose a neotype and give preference to selecting that from the sanctioning work. He thought the new proposals were an improvement, working somewhat more within the confines of the *Code*, but he felt it was essentially expanding the definition of original material so that other materials were available for lectotypification, and he simply did not see why it could not still be called a neotype, because that was what it was, and give preference in standard practice to selection of the neotype from the sanctioning work where necessary.

**McNeill** added a point of clarification about what was in the existing *Code*: the present wording of Art. 7.8, taken as literally set out, said for the purposes of typification of a sanctioned name, an element from the context of the sanctioning work must be taken as lectotype. In other words, he thought that the new proposal was actually being broader and using the protologue more. He added that, in effect, under the existing *Code*, the original material was the material in the sanctioning work, which turned out not to be what most mycologists actually wanted. He interpreted this as a change that was actually moving more back to the protologue than the current wording of the *Code*.

**Knapp** instructed the microphone runner not to give Redhead the microphone unless she said he could have it. She allowed him to have it. [Laughter.]

**Redhead** pointed out that if you looked at what was being replaced, which was Art. 7.8, it had fuzzy wording, which had allowed people to go in several directions. The intent was to make this more precise as to what may be selected and what may not be selected, because you may have material in the sanctioning work that had been designated as type, but it was not in the protologue, and if this allowed you to typify them effectively on that, then what was the material? If it was not in the protologue then it could not be a lectotype, unless the definition of lectotype was changed, which was basically what was being done. He explained that that was how they had come up with the alternative term “sanctiotype”, but in this case the preference was to modify slightly the definition of what a lectotype was rather than introduce a new term, and then eliminate Art. 7.8 and replace it with the changes that were here.

**Knapp** suggested that it was necessary, as a Section, to decide on whether to vote on the proposals individually or to vote on them as a package. She chose to use the Chair’s prerogative and moved to a vote on whether to consider the proposals as a package and vote on them once or whether to consider them one by one and vote on each one. She added that as there were two alternatives the vote would be a simple majority. [There was a majority in favour of voting on the proposals as a package. Laughter.]

**Norvell’s set of proposals** was **accepted**.

[*Discussion of a new proposal by Prud’homme van Reine, concerning Art. 8.4, occurred here and has been moved to the normal order in the Second Session on Monday afternoon.*]

**Wiersema’s set of proposals**

**McNeill** moved on to an amendment to Art. 18.3, a proposal that he had initiated but Wiersema was going to present on his behalf.

**Wiersema** explained that the proposal came about because 12 sets of proposals involving some 86 family names that were deemed to be illegitimate came to *Taxon* for editing, and the reason they were illegitimate was because of the current provisions of Art. 18.3 and 6.4, because at the time that these families were created the genera upon which they were based were themselves illegitimate. So a name of a family based on an illegitimate generic name was illegitimate unless it was conserved. These families were not conserved at that time, and in fact most of them were not conserved now. They were mainly bryophyte, algal and fungal families and one gymnosperm family included.

The package also dealt with Art. 6.4, which stated “A name which according to this *Code* was illegitimate when published cannot become legitimate later unless it is conserved”. These family names, not having been conserved, would remain illegitimate unless something was done about it. The new wording was suggested in order to resolve the issue, by making conservation of the genus name also create the conserved family name or override illegitimacy of the family name.

**McNeill** explained that all of those names were based on what were now conserved generic names.

**Wiersema** agreed that the generic names were now conserved but that did not affect the family names, which remained illegitimate unless the suggested wording was added. The only way that this could cause a problem was if people were actually aware of this and had dismissed the use of these family names and adopted later ones, but since no one had ever been aware of it he thought that they were using most of these family names. He concluded by saying that for the family names to remain available the suggested action was necessary.

[The **set of proposals** was **seconded** and **supported** by three others.]

**Van Rijckevorsel** was missing something in the wording. He suggested that perhaps it could be attended to editorially but it seemed redundant because a generic name that was conserved could not be illegitimate.

**McNeill** responded that it was based on an illegitimate generic name and that name had since been conserved and was now not illegitimate, but the point was although the rules were retroactive the status of a name was not retroactive, so when the family name was published it was illegitimate.

**Van Rijckevorsel** understood that, but felt that the way it was phrased it did not read that way. He thought it was redundant because “is illegitimate” and “is conserved” are in present tense.

**Funk** suggested that it be referred to the Editorial Committee as she did not think the Section wanted to sit here and argue about present tense.

**Knapp** suggested that the Section could argue about the past though, as “we’re very good at that”. [Laughter.]

**Wiersema** pointed out that Art. 19.5 dealt with names of subdivisions of families in the same way, so that the situation could not arise where the family name had been fixed but the name of the subdivision of the family that was based on the same type as the family name could end up still being illegitimate if the changes were not made.

**Barrie** had two points. He felt that this was an extremely efficient way of handling the problem and it should be passed. The other was that the issue could not simply be referred to the Editorial Committee because the changes had to be voted in and then the Editorial Committee would clean up the language.

**Knapp** assured him that she was not going to let it just be referred to the Editorial Committee.

**Barrie** replied that he trusted her.

**Cameron** suggested that the word “or” simply be removed before “the generic name” in the Art. 18.3 amendment, because to him it would read correctly and would take account of the concern that some people had. He felt that that was simply a grammatical amendment and knew it would be addressed by the Editorial Committee.

**Knapp** asked if he was proposing that as a formal amendment.

**Cameron** was just wondering whether it would influence people’s view if the Editorial Committee accepted that that was a possible way of amending it.

**Knapp** recognized the woman who was waving her arm around.

**Funk** tried again. She apologized for suggesting the proposal go to the Editorial Committee but wondered if it was possible to just get a sense if everybody approved the essence of what was trying to be accomplished here, in a vote and it could then go to the Editorial Committee.

**Knapp** thanked her and moved to a vote on the package of additions. She assured everyone that the Editorial Committee would take account of details of tense and things with this proposal from the floor.

**Wiersema’s set of proposals** was **accepted**.

**Lendemer’s proposal**

**McNeill** introduced a new proposal to make a modification to the new component of Art. 14 that had been accepted on Thursday with regard to the conserved list of names.

**Knapp** asked for the microphone for the man in red and commented that he had learned that if he wore a red jumper she would be able to find him.

**Buck** [?] suggested that this was just like Nancy Reagan.

**Lendemer** agreed it was just like Nancy Reagan. He intended the new proposal to be a new Article within Art. 14. He noted that the numbering was subjective and available for alteration by the Editorial Committee. He felt it was mainly the content and essence of the text being approved by the Section that he and the mycological community were looking for. He added that the proposal had been passed out as hard copy, although there were a few minor modifications. He outlined that what had changed was the removal of Art. 59, because lichens were already exempted from Art. 59 as the new version that was passed yesterday and he did not want to go and open that can of worms yet again. He had also added “taxonomically” after “with them” to qualify that it would apply to lichenized fungi and the fungi that have traditionally been associated with them taxonomically, and there was a reason for that, which he would explain at the end.

He gave a short explanation of why the changes were needed. On Thursday the Section had passed sweeping changes to the rules that govern pleomorphic fungi. Unfortunately, because lichens were not exempted from the new rules, when they had been before, there was a significant potential for broad destabilization of lichen nomenclature at all ranks, including the most common species and genera, such as *Parmelia*, *Physcia*, *Usnea* and some of the most common species in these genera. He explained that it stemmed from the fact that lichenologists never considered the state of a type—whether or not it was an anamorph or a teleomorph—when determining priority. Nor did they consider sterility, but he dismissed that as another mess. The point he wished to make was that Art. 59 had never applied to lichens and the new Art. 59 did not apply to lichens, but all of the subsequent Articles that were proposed to deal with containing the mess made by removing the old version of Art. 59 did not exempt lichens, when they should have.

He had included “other taxonomically similar fungi” because there were some groups of fungi that were not lichenized but were traditionally treated by lichenologists and not by mycologists, and he felt it was important to realize that dual nomenclature had never happened in these groups. Just because other groups of mycologists used dual nomenclature did not mean that lichenologists did and the *Mycocaliciaceae* was an excellent example. This was a group that was embedded within an otherwise lichenized group and they were treated with lichens traditionally but were not lichenized fungi and had never had dual nomenclature applied, had never had separate anamorph and teleomorph names. He had sent the amended version of the proposal with the Articles that had been passed yesterday to the lichen LISTSERV with 482 recipients. Of course, it was midnight in most of the world where lichenologists reside, but he did have correspondence with a colleague from the Field Museum, who supported it.

[The **proposal** was **seconded** and **supported** by three others.]

**Reveal** suggested a friendly amendment that at the end, where it said Art. “14”, it would refer to Art. “14(new.1)”. [This was considered a **friendly amendment**.]

**Hawksworth** thought that a lot of it was not actually necessary because the lichen thalli were not pleomorphic in the sense that it was used. He felt that it was a misunderstanding that some people evidently had. He was very much against having the reference to Art. 14(new.1). He suggested that there may well be cases where lists included lichenized and non-lichenized species that may need to be protected, so he could not see that caused any problems at all. He added that really the only thing, if those were concerned, was Art. 57.2, where that could easily be dealt with just editorially; he felt it did not necessarily need a separate Art. 14(new.2).

**Redhead** agree with Hawksworth that it was unnecessary for most of the Articles. He suggested that the concerns about the non-lichenized fungi could be amended or taken care of by simply amending the new Art. 57.2 by saying “non-lichenized pleomorphic fungi”.

**McNeill** asked if he was proposing that as an amendment.

**Redhead** confirmed that he was. He was worried about the suggested wording, fearing that it was very fuzzy in saying “fungi traditionally associated with them”. He thought that with lichenized fungi it was a very slippery slope with very imprecise wording, so it would not be possible to know which fungi would apply or not apply there. He felt that was opening the door to something that should never have been…

**McNeill** asked Redhead to confirm that his amendment was to replace what was on the board by an addition to Art. 57.2.

**Redhead** confirmed that that was correct and it would say “in non-lichenized pleomorphic fungi.” [The **amendment** was **seconded**.]

**Knapp** pointed out that as an addition to Art. 57.2 it was not actually an amendment to the proposal, it was a suggested different way to deal with the issue.

**Buck** [?] suggested it was a separate proposal.

**McNeill** pointed out that Madam Chairman could rule it as a separate proposal and give notice that it was going to happen.

**Knapp** thought that it could not be treated as an amendment to Lendemer’s proposal, because that was to add something to Art. 14 and the proposed amendment to deal with the same thing was something added to Art. 57. It was decided that it would be much simpler to deal with them separately.

**Price** wished to clarify something, because she believed that on Thursday the addition of the lichens was proposed and voted down by the Section, and now it was being re-proposed. She wanted to make sure that this was actually acceptable, because it was proposed on Thursday and was refused, which was the reason why the new proposal had been introduced.

**Knapp** agreed that that was correct and added that at the point when Redhead was going to make the proposal, she was going to tell him that it was not possible to revisit something that had been voted against, unless the proposal to revisit it came from the losing side.

**McNeill** did not think it was revisiting it…

**Unknown speaker** called the question on the Lendemer proposal. [There was a sufficient majority in favour of voting.]

**Lendemer’s proposal** was **accepted**.

**McNeill** noted that therefore the other proposal became irrelevant.

[*Discussion of a new proposal by Cameron & Prud’homme van Reine, concerning Art. 32, occurred here and has been moved to the normal order in the Eighth Session on Tuesday afternoon. A short discussion of a new proposal by Herendeen, concerning Art. 11.8, took place here and has been moved to the normal order under Art. 11 in the Third Session on Tuesday morning. Discussion of a new proposal by Marhold, concerning Art. 37bis, occurred here and has been moved to the normal order in the Seventh Session on Thursday morning.*]

**Barrie & al.’s proposal**

**McNeill** thought that this was the suitable point to introduce another proposal from the floor to establish a Special Committee on the registration of names other than fungi in the names of Barrie, Stevens, Funk, Cafferty and Greuter. [The **proposal** was **seconded** and **supported** by three others.]

**Barrie** added that the reason they were proposing this was because it had become pretty obvious to many that, as electronic publication was coming in, there would need to be a mechanism for keeping track of the names, and the best one they could come up with was registration. With the Special Committee they wanted to look into the possibilities of registration, how to construct it, the mechanism and where to put it, and report to the next Congress. At that point he figured the Section should have a better idea of what was going on, and there would also be the experience of the mycologists to see how theirs [their system of registration] had worked.

**Marhold** commented that that had been his alternative suggestion after the result he had expected [of his new proposal on Art. 37*bis*], but he wondered whether it was possible to put into the mandate of the Special Committee to try to establish some voluntary registration, which was actually the argument by the fungal community—that they were able to come to this Congress and say “Hey, look, it works”—because he suggested that if the Committee came to the next Congress with just the proposal and then there was the same argument of “we do not see it running”, then 12 years would already have been lost. He felt that another six were going to be lost and did not want to lose another 12 years.

**Barrie & al.’s proposal** was **accepted** and a new **Special Committee** was established [the Special Committee on Registration of Algal and Plant Names (including fossils)].

[*Discussion of a new proposal by Nagamasu, concerning Art. 60.1, occurred here and has been moved to the normal order in the Ninth Session on Friday morning. Discussion of a new proposal by Davidse & Ulloa, concerning Art. 60.6, occurred here and has been moved to the normal order in the Ninth Session on Friday morning. Discussion of a new proposal by Hawksworth, to amend the conservation of names under Art. 14 to all ranks, occurred here and has been moved to the normal order in the Fourth Session on Tuesday afternoon. Discussion of a new proposal by Veldkamp, concerning Rec. 32B, occurred here and has been moved to the normal order in the Fourth Session on Tuesday afternoon. Discussion of a new proposal by Reveal, concerning Art. 46, occurred here and has been moved to the normal order in the Seventh Session on Thursday morning. Discussion of a new proposal by Norvell, to amend the title of the Code, occurred here and has been moved to the original discussion about the title in the Second Session on Monday afternoon.*]

**Hawksworth’s proposal**

**McNeill** moved on to the last proposal he had, which was to approve the formation of a Special Committee on the *BioCode*. [The **proposal** was **seconded** and **supported** by three others.]

**Hawksworth** noted that there was a situation where proposals to further develop the *BioCode* were progressing. He reported that there was a lot of progress made at the International Congress of Systematic and Evolutionary Biology in Berlin in February and the revised draft was out for discussion. He felt the Section needed a mechanism to have an authoritative botanical view put into that discussion about the document and what, if anything, should be done with it.

**Gereau** suggested that in the published documentation on the development of the *BioCode* there were a number of provisions that were counter to the direction in which many members of the Section, in his discussions, wanted to go. He thought the Section should have nothing to with it and was completely against the establishment of this Committee.

**Hawksworth’s proposal** was **rejected**.

**Knapp** used the Chair’s privilege to add a point of information and pointed out that if people wanted to comment on the *BioCode* there was a mechanism that had been set up through the International Commission on Zoological Nomenclature, where there was a website and a commentary page.

**Dorr** moved to adjourn the Nomenclature Section for the Congress.

**Knapp** said no, there was still business to do.

**McNeill** agreed that it was premature, there was still some business, although he had no more proposals from the floor regarding the *Code*, or indeed any procedural matters, but of course there remained the normal business of Committee reports.

### Report of the Nominating Committee

**McNeill** noted that the report of the Nominating Committee had been distributed and he invited the Chair, Barbara Briggs, to say something to the background and introduction.

**Briggs** wished to say that the main work towards the recommendations had been done by the secretaries and members of the relevant Committees, and that was acknowledged, although the names in the nominations were not always completely identical with their lists. She added that one name, that of Buck for the Editorial Committee, was omitted by mistake from the typed copy and the electronic version given to the Rapporteurs and had been written on the distributed hard copies. The Committee had made a few comments on the matters they thought important, but as that was all in the distributed hard copies she did not feel the need to speak about them.

**Redhead** noted that, in the Committee for Fungi, “Tony May” should be changed to “Tom May”.

**Knapp** assured the Section that the name Committee for *Bryophyta* would be changed to Committee for Bryophytes, following the earlier decision once all decisions were ratified by the International Botanical Congress.

**Karen Wilson** had a question of clarification: under the General Committee, was “Francisco” Zuloaga intended to be Fernando Zuloaga?

**Knapp** confirmed this was the case.

**Briggs** apologized for the typos.

The **Report** of the Nominating Committee was **approved** unanimously.

### Reports of the Permanent Nomenclature Committees

Committee for Algae

**McNeill** asked the secretaries of the Permanent Nomenclature Committees to be quite brief in highlighting the main elements of their six years’ work.

**Prud’homme van Reine** was not expecting to have to give a report.

**Knapp** reassured him that he did not have to say anything.

**Prud’homme van Reine** was pleased and responded “Okay, fine!” [Laughter.]

The **Report** of the permanent Committee for Algae was **approved**.

Committee for Fungi

**Norvell** summarized the six years thus: “Much controversy, many votes, everything settled, we’re happy”.

The **Report** of the permanent Committee for Fungi was **approved**.

Committee for Bryophyta (Committee for Bryophytes)

**Klazenga** was also not expecting to give a report either. He noted that they had had very few proposals, only 10 or so and there was nothing really controversial.

The **Report** of the permanent Committee for *Bryophyta* was **approved**.

Committee for Fossil Plants (Committee on Fossils)

**Herendeen** felt that he could not be creative: the Committee had published two reports and their activities were reported in those reports. He had nothing more to add.

The **Report** of the permanent Committee for Fossil Plants was **approved**.

Committee for Vascular Plants

**Applequist** reported that in the past six years, during which Dick Brummitt was serving as secretary of the Committee, the Committee for Vascular Plants had processed over 300 proposals to conserve or reject names, in addition to a small number of requests for rulings on nomina subnuda and confusable names.

The **Report** of the permanent Committee for Vascular Plants was **approved**.

Editorial Committee

**Turland** had just a few words on the Editorial Committee as was the tradition. He stated that it was the report of the Editorial Committee on the *Vienna Code*, the red *Code*, which was produced according to the decisions made at Vienna and had been approved at the start of the [Melbourne] Section as the basis for discussions.

He wanted to remember Guanghua Zhu from the Missouri Botanical Garden, who was appointed by the Vienna Nomenclature Section to the Editorial Committee, but sadly died in November of 2005 before the Editorial Committee meeting.

The Editorial Committee had met in St Louis in January 2006 for one week. John McNeill, the Rapporteur, had very carefully prepared an edited and marked-up copy of the *St Louis Code* with all of the Vienna changes tracked, which served as a basis for their deliberations. Eventually this led to a final PDF—so he noted that they were ahead of the game with PDFs—submitted to the printer on exactly the 25th of July 2006 and, according to the letter from the distributor inside John McNeill’s copy, it was mailed on the 21st of September 2006, which he supposed must be the publication date. It was published as *Regnum Vegetabile* volume 146.

He wished to acknowledge in particular: Paul Silva, who produced the first draft of the Glossary, now App. VII; also Dan Nicolson, who provided the information for the Appendices; Gea Zijlstra, Vincent Demoulin and Paul Silva, who checked the Appendix entries for bryophytes, fungi, and algae, respectively; also the members of the Special Committee on Suprageneric Names, who checked and submitted the corrections for App. IIB; also all of those who submitted ideas for Examples and editorial improvements in the *Code*.

The **Report** of the Editorial Committee was **approved**.

General Committee

**McNeill** introduced the final report of the General Committee, which was distributed at the Section but was also already [electronically] published in *Taxon* [Taxon 60: 1211–1214. 2011].

**Barrie** noted that everyone should have had a copy of it in their Congress packet. He wished to point out a few lines that were omitted from the printed copy, although they were in the published copy. These were from Report 61 for the Committee for Vascular Plants. One was that *Brugmansia aurea* was a name that came up for consideration and whether or not the description was adequate. The General Committee approved the Vascular Plant Committee’s recommendation that this was an adequate description, but there were several others that were still under debate: *Monorobea esculenta* and *Agave noah*, which failed to get majority votes in the Committee, so they were still sitting in the General Committee, not referred back or approved, so they continued to be under consideration.

He also wished to say first that Dan Nicolson—who had been the Chairman and had resigned in November 2010—deserved to be mentioned and remembered for all his contributions to botanical nomenclature over the years and to note that he was certainly missed. [Applause.]

There were 20 reports published by the other Permanent Committees, but only 17 of them included proposals to conserve or reject or rulings under Art. 32.5 on nomina subnuda or Art. 53.5 on confusability. The other three were simply recommendations on the proposals to amend the *Code*, and the General Committee did not review those.

The one thing that he wished to point out was that the General Committee was going to work with the Editorial Board of *Taxon* to see that criteria were set up for publishing proposals under Art. 32.5, nomina subnuda, and Art. 53.5, confusability. Obviously Art. 53.5 was new from Vienna, so it was a novelty, but confusability had been in the *Code* for a long time. He was not sure which original *Code* it appeared in, but it had been there for quite some time. People were permitted to send in name sets and ask if they were confusable, but it was extremely infrequent in the past and the tradition had built up of merely mailing them to the secretaries of the relevant Committees involved. While they worked very well as communication between the author and the Committee, the problem was that the rest of the botanical community had no idea that these issues were being adjudicated. So from now on the plan was to try to get them published instead of simply done through correspondence.

The **Report** of the General Committee was **received**.

**Barrie** made the recommendations as voted by the Committee in the report and submitted them to the Nomenclature Section.

The **Report** of the General Committee was **approved**.

### Other business

**McNeill** had only one further item of business. In order that the work of the past week not be lost and run into the sand he moved the following motion: “The Section instructs the Rapporteur-général to present a resolution to the Resolutions Committee of the XVIII International Botanical Congress, to the effect that that Congress approve the decisions of the Nomenclature Section.” [The **motion** was **seconded**.]

[The **motion** was **approved**.]

**McNeill** noted that that concluded the formal business. He thought that there were a lot of people to express enormous appreciation to.

**Knapp** started by first thanking Pauline Ladiges and all her team of students and microphone handlers, and the University of Melbourne for providing a really fabulous place to hold the Nomenclature Section that made having the deliberations much, much easier. [Applause.] She had one more thing that she wished to add personally, because when John had asked her to chair the meeting she had said “No, no, I don’t know anything about nomenclature”, and he said “No, you don’t need to know about nomenclature. All you need to do is be able to keep order”. She said “I’m not sure I can do that either”. But having done this for the whole week, her admiration for her colleague Dan Nicolson was huge and she wanted the Section to also thank him not only for all the years of service he gave to botanical nomenclature but also for having done this job, which she was not sure anybody could do more than once—although Hervé Burdet did. She wanted to say that her appreciation for Dan had increased 100-fold.

She also wished to thank all of the participants for behaving themselves and felt that a round of applause was well earned. [Applause.]

The last bit of information that she imparted was that the sign-up sheets for the Special Committees that had been established were available at the front of the lecture theatre: Special Committee on Institutional Votes; Special Committee on Harmonization of Nomenclature of *Cyanophyta/Cyanobacteria*; Special Committee on Publications Using a Largely Mechanical Method of Selection of Types (Art. 10.5) (especially under the *American Code*); Special Committee on By-laws for the Nomenclature Section (with a Subcommittee on Governance of the *Code* With Respect to Fungi); and Special Committee on Registration of Algal and Plant Names (including fossils).

**Karen Wilson** proposed a vote of thanks to Sandy as President, to John as Rapporteur-général, to Nick as his Vice-rapporteur, and to the Recorders, Brendan Lepschi, who had unfortunately already had to leave, and Anna Monro, who had been labouring away valiantly. She felt that the whole group had formed a very good team and had helped the Section get through all of the business. She concluded that it had ended up being a momentous Nomenclature Section, in fact, because there were some big changes made. She thought that everyone was going to greatly appreciate what went on in Melbourne in July 2011. [Extended applause.]

## Appendix A

**Institutional votes**

Preliminary notice of institutional votes at the Nomenclature Section of the XVIII International Botanical Congress, Melbourne, together with a list of the institutional votes at the XVII International Botanical Congress, Vienna, was provided by McNeill & Turland (in Taxon 58: 971–973. 2009). A revised list of institutional votes was provided in 2011 prior to the Melbourne Congress by Turland & McNeill on the IAPT website (http://www.iapt-taxon.org/downloads/institutional_votes_XVIII_International_Botanical_Congress.pdf). Summarizing from the latter document, since the Vienna Congress, 27 requests were received for new vote allocations or changes to existing allocations. These requests were considered by the Bureau of Nomenclature of the Melbourne Congress, which recommended 43 changes to the Vienna list, not only in response to the requests received but also where it was known that institutions had been merged with others or were no longer active. The General Committee approved the revised list (22 for: 0 against: 6 not responding). No attempt was made at a comprehensive revision of institutional votes, and it was acknowledged that many inconsistencies remained.

The following list includes all institutions that were entitled to vote at the Melbourne Congress, Institutions that were added to the list since the Vienna Congress are preceded by a “#” symbol. An asterisk (*) denotes the institutions the institutional votes of which were exercised by delegates or vice-delegates at the Nomenclature Section in Melbourne.

Institutions are identified by the name of the city in which each is located, followed by the standard herbarium code used in *Index herbariorum* (http://sweetgum.nybg.org/ih/). The number of votes allotted to each institution is given in the right-hand column. The “+” symbol is used when two or more herbaria are treated together as a single institution in the wide sense of the term (Div.III.4(b)(2)(second sentence)), e.g., CAN + CANA + CANL + CANM are treated as a single institution with five votes.

**Institutional votes.**

	Aarhus (AAU)	4
	Aberdeen (ABD)	1
	Abidjan (UCJ)	1
*	Addis Ababa (ETH)	1
*	Adelaide (AD)	4
	Akureyri (AMNH)	1
	Albany (NYS)	1
*	Albuquerque (UNM)	1
#	Alice Springs (NT)	1
*	Allahabad (BSA)	1
	Alma-Ata (AA)	1
	Ames (ISC)	2
	Amherst (MASS)	1
*	Ankara (ANK)	2
*#	Ankara (HUB)	2
*	Ann Arbor (MICH)	4
	Antananarivo (TAN)	1
	Aquila (AQUI)	1
	Arcata (HSC)	1
*#	Armidale (NE)	1
	Ashkhabad (ASH)	1
	Asunción (FCQ)	1
	Athens, Greece (ATHU)	2
	Athens, USA (GA)	1
	Auckland (AK)	2
*	Auckland (PDD)	1
*	Austin (TEX)	4
#	Aveiro (AVE)	1
*	Baam (CBS)	4
	Baghdad (BAG)	1
	Baku (BAK)	1
	Bangi (UKMB)	1
*	Bangkok (BKF)	2
	Barcelona (BC)	2
#	Barcelona (BCN)	2
	Bari (BI)	1
	Basel (BAS)	1
	Baton Rouge (LSU + LSUM)	2
	Beijing (CAF)	1
	Beijing (CMMI)	1
	Beijing (HMAS)	5
	Beijing (PE)	7
	Belem (IAN)	1
	Belem (MG)	1
*	Beltsville (BARC)	1
*	Beltsville (BPI)	3
	Beograd (BEO)	1
*	Bergen (BG)	2
*	Berkeley (UC)	5
*	Berlin (B)	7
*#	Berlin (BHU)	1
	Berlin (BHUPM)	1
	Bern (BERN)	1
	Birmingham (BIRM)	1
	Blacksburg (VPI)	1
*	Bloomington (IND)	1
*	Bmo (BRNM)	1
*	Bmo (BRNU)	1
	Bogor (BO)	4
*	Bogotá (COL)	3
	Bologna (BOLO)	1
	Bombay (BLAT)	1
	Bonn (BONN)	1
	Boulder (COLO)	1
	Brasilia (IBGE)	1
	Brasilia (UB)	1
*	Bratislava (BRA)	1
*	Bratislava (SAV)	1
*	Bratislava (SLO)	2
	Brooklyn (BKL)	1
	Bruxelles (BRVU)	1
	Bucureşţi (BUCA + BUCM)	2
	Budapest (BP)	5
	Buenos Aires (BA)	3
	Buenos Aires (BAA)	1
*	Buenos Aires (BAF)	2
	Buenos Aires (BAFC)	1
	Buffalo (BUF)	1
	Burdwan (BURD)	1
*	Burlington (VT)	1
*#	Cairns (CNS)	2
	Cairo (CAI)	2
	Cairo (CAIM)	1
*	Calcutta (CAL)	5
	Calgary (UAC)	1
	Cambridge, UK (AAS)	1
	Cambridge, UK (CGE)	1
	Cambridge, UK (CGG)	1
*	Cambridge, USA (GH)	6
	Camerino (CAME)	1
*	Campinas (UEC)	2
*	Canberra (CANB)	6
*	Cape Town (BOL)	2
*	Cape Town (NBG)	2
*	Caracas (VEN)	2
	Carbondale (SIU)	1
	Cardiff (NMW)	1
*	Castelar (BAB)	1
*	Catania (CAT)	1
	Cayenne (CAY)	1
*	Champaign (ILLS)	1
	Changsha (HNNU)	1
	Chapel Hill (NCU)	2
	Chengdu (CDBI)	2
	Chengdu (SZ)	1
*	Chicago (F)	5
	Chongqing (HWA)	1
	Chongqing (SM)	1
*	Christchurch (CANU)	1
*	Christchurch (CHR)	3
	Claremont (RSA)	2
	Clermont-Ferrand (CLF)	1
*	Cluj-Napoca (CL)	1
#	Coffs Harbour (CFSHB)	1
*	Coimbatore (MH)	2
	Coimbra (COI)	3
*	College Park (MARY)	1
	College Station (TAES)	1
	Columbus (OS)	2
*	Como (PERTH)	3
*	Concepción (CONC)	1
*	Copenhagen (C)	7
*	Córdoba (CORD)	2
*	Corrientes (CTES)	1
*	Corvallis (OSC)	1
	Curitiba (MBM)	1
	Dacca (DACB)	1
*	Darwin (DNA)	1
	Davis (DAV)	1
	Dehra Dun (DD)	2
	Delaware (OWU)	1
	Dublin (DBN)	1
*	Dublin (TCD)	1
#	Durango (CIIDIR)	1
*#	Durban (NH)	1
*#	Durban (UDW)	1
	Durham (DUKE)	2
*	East Lansing (MSC)	2
*	Edinburgh (E)	6
	Edmonton (ALTA)	1
	Edmonton (UAMH)	1
*	Egham (IMI)	4
	Fairbanks (ALA)	1
	Fairfax (GMUF)	1
*	Firenze (FI + FT)	4
	Fort Worth (BRIT)	3
*	Frankfurt (FR)	1
	Fuzhou (FNU)	1
	Gainesville (FLAS)	2
	Gatersleben (GAT)	2
*	Genève (G)	7
	Gent (GENT)	1
	Georgetown (BRG)	1
	Giessen (GI)	1
	Göteborg (GB)	4
	Göttingen (GOET)	1
	Grahamstown (GRA)	1
	Granada (GDA)	1
	Graz (GJO)	1
	Graz (GZU)	2
	Guadalajara (GUADA)	1
	Guangzhou (IBSC)	4
	Guangzhou (SYS)	1
	Guelph (OAC)	1
	Guilin (IBK)	2
	Guiyang (HGAS)	1
*	Habana (HAC)	2
*	Habana (HAJB)	3
	Halle (HAL)	2
	Hamburg (HBG)	3
	Hangzhou (HZU)	1
	Hanoi (HN)	2
	Hanoi (HNU)	1
*	Harare (SRGH)	2
	Harbin (NEFI)	1
*	Helsinki (H)	7
	Hiroshima (HIRO)	2
*	Hobart (HO)	1
	Hong Kong (CUHK)	1
	Honolulu (BISH)	2
	Iaşi (I)	1
	Ibadan (FHI)	1
*	Indooroopilli (BRI)	4
	Innsbruck (IB)	1
*	Islamabad (ISL)	1
	Islamabad (RAW)	1
*	Istanbul (ISTE)	2
*	Istanbul (ISTF)	1
*	Ithaca (BH)	3
	Ithaca (CUP)	1
*	Izmir (EGE)	1
	Jaca (JACA)	1
	Jaipur (RUBL)	1
*	Jena (JE)	3
	Jerusalem (HUJ)	2
	Johannesburg (J)	1
*	Kampala (MHU)	1
	Kanazawa (KANA)	1
*	Karachi (KUH)	2
	Karlsruhe (KR)	1
	Kathmandu (KATH)	1
*	Kew (K)	7
*	Kiev (KW)	5
*#	Kimberley (KMG)	1
	Kingston (IJ)	1
	Kishinev (CHIS)	1
	Knoxville (TENN)	1
	Kórnik (KOR)	1
	Kraków (KRA)	2
	Kraków (KRAM)	3
	Kuala Lumpur (KEP)	1
	Kuala Lumpur (KLU)	1
	Kuching (SAR)	1
	Kunming (KUN)	5
	Kunming (PYU)	1
	Kunming (SWFC)	1
	Kunming (YUKU)	1
*#	KwaDlangezwa (ZULU)	1
*	Kyoto (KYO)	2
	La Laguna (TFC)	1
	La Paz (LPB)	1
	La Plata (LP)	1
	La Plata (LPS)	1
	Lae (LAE)	1
	Lafayette (LAF)	1
	Lanzhou (NWTC)	1
	Laramie (RM)	1
	Lausanne (LAU)	1
*	Lawai (PTBG)	1
*	Lawrence (KANU)	1
*	Leeds (LDS)	1
	Legon (GC)	1
	Leicester (LTR)	1
*	Leiden (L)	7
	Leipzig (LZ)	1
*	Leningrad (LE)	7
	Leningrad (LECB)	1
*	Leningrad (WIR)	1
*	Liège (LG)	2
	Lima (USM)	2
	Linz (LI)	1
*	Lisboa (LISC)	2
	Lisboa (LISI)	1
	Lisboa (LISU)	2
	Lisle (MOR)	1
	Liverpool (LIV)	2
	Ljubljana (LJU)	1
	Lódz (LOD)	1
*	Logan (UTC)	1
	London, Canada (UWO)	1
*	London, UK (BM)	7
	Los Angeles (LA)	1
	Los Baños (CAHUP)	1
	Louvain-la-Neuve (MUCL)	1
	Lublin (LBL)	1
	Lucknow (LWG)	2
	Lund (LD)	6
	Lushan (LBG)	1
	Luxembourg (LUX)	1
	Lvov (LW)	1
*#	Lydenburg (BNRH)	1
*#	Lydenburg (LYD)	1
	Madison (CFMR)	1
	Madison (WIS)	2
*	Madrid (MA)	4
*	Madrid (MAF)	1
	Magadan (MAG)	1
	Mainz (MJG)	1
	Málaga (MGC)	1
	Manaus (INPA)	1
	Manchester (MANCH)	1
	Mangilao (GUAM)	1
	Manhattan (KSC)	1
	Manila (PNH)	1
*	Maputo (LMA)	1
*#	Maputo (LMU)	1
	Maracay (MY)	1
	Marseille (MARSSJ)	1
	Matsumoto (SHIN)	1
	Mayaguez (MSM)	1
	Medellín (HUA)	1
*	Meise (BR)	6
*#	Melbourne (LTB)	1
*	Melbourne (MEL)	6
*	Melbourne (MELU)	2
	Mérida (CICY)	1
	Mesa de Cavacas (PORT)	1
	Mexico City (ENCB)	3
*	Mexico City (FCME)	1
*	Mexico City (MEXU)	5
	Mexico City (UAMIZ)	1
	Miami (FTG)	2
	Milwaukee (MIL)	1
*	Minsk (MSK)	1
	Missoula (MONTU)	1
*	Montecillos (CHAPA)	1
	Monterey (UNL)	1
	Montevideo (MVM)	1
*	Montpellier (MPU)	2
*	Montreal (MT)	2
	Montreal (MTMG)	1
*	Morgantown (WVA)	1
	Moscow, Russia (MHA)	2
*	Moscow, Russia (MW)	2
	Moscow, USA (ID)	1
*	München (M)	5
*	München (MSB)	1
	Murcia (MUB)	1
*	Nairobi (EA)	2
	Nairobi (NAI)	1
	Nanjing (N)	1
	Nanjing (NAS)	2
	Nanjing (NF)	1
	Napoli (NAP)	1
	Neuchâtel (NEU)	1
	New Delhi (HCIO)	1
	New Haven (YU)	1
	New Orleans (NO)	1
*	New York (NY)	7
	Nichinan (NICH)	2
	Norman (OKL)	1
	Novosibirsk (NS)	1
	Novosibirsk (NSK)	1
	Oeiras (LISE)	1
#	Oldenburg (OLD)	1
	Oslo (O)	5
*	Ottawa (CAN + CANA + CANL + CANM)	5
*	Ottawa (DAO)	3
*	Ottawa (DAOM)	3
*	Oulu (OULU)	1
	Oviedo (FCO)	1
	Oxford, UK (FHO)	1
	Oxford, UK (OXF)	1
	Oxford, USA (MU)	1
*	Padova (PAD)	2
*	Palermo (PAL)	3
*	Panama (PMA)	1
*	Paris (P)	4
*	Paris (PC)	2
*	Patras (UPA)	2
*	Pátzcuaro (IEB)	1
	Pavia (PAV)	2
	Peoria (NRRL)	2
	Peradeniya (PDA)	1
*#	Perth (KPBG)	1
	Perugia (PERU)	1
	Philadelphia (ANSP)	1
	Philadelphia (PH)	2
*#	Pietermaritzburg (NU)	1
	Pisa (PI)	2
	Pittsburgh (CM)	2
	Plovdiv (SOA)	1
	Porto (PO)	1
	Porto Alegre (HAS)	1
	Porto Alegre (ICN)	1
*	Potchefstroom (PUC)	1
*	Poznao (POZ + POZG)	2
*	Praha (PR + PRM)	2
*	Praha (PRC)	3
*	Pretoria (PRE)	6
	Pretoria (PREM)	2
*	Pretoria (PRU)	1
	Provo (BRY)	1
	Pullman (WS)	1
	Pullman (WSP)	1
	Pune (AMH + AHMA)	1
*	Pune (BSI)	1
	Quebec (QFA)	1
	Quito (QCA)	1
*	Quito (QCNE)	1
	Rabat (RAB)	1
	Raleigh (NCSC)	1
*	Reading (RNG)	2
	Recife (URM)	1
	Regensburg (REG)	2
	Reykjavik (ICEL)	1
	Riga (RIG)	1
	Rio de Janeiro (HB)	1
	Rio de Janeiro (R)	2
*	Rio de Janeiro (RB)	5
	Riverside (UCR)	1
	Rockville (ATCC)	1
	Roma (RO)	1
*#	Rotorua (NZFRI)	1
	Rydalmere (DAR)	1
	S Miguel Tucuman (LIL)	3
	Saint Andrews (STA)	1
	Saint Augustine (TRIN)	1
*	Saint Louis (MO)	7
	Saint Paul (MIN)	2
	Salaspils (LATV)	1
	Salt Lake City (UT)	1
	Saltillo (ANSM)	1
	San Diego (SD)	1
	San Francisco (CAS)	4
	San Francisco (SFSU)	1
	San Isidro (SI)	3
	San Jose (CR)	1
	Sandakan (SAN)	1
	Santa Barbara (SBBG)	1
	Santiago (SGO)	1
	Santo Domingo (INB)	1
	Santo Domingo (JBSD)	1
	São Leoopoldo (PACA)	1
*	São Paulo (SP)	4
*	São Paulo (SPF)	2
	Sapporo (SAP)	1
	Sarajevo (SARA)	1
	Saskatoon (SASK)	1
	Sassari (SS)	1
	Seattle (WTU)	2
	Sendai (TUS)	1
	Seoul (SNU)	2
	Sevilla (SEV)	2
	Shanghai (FUS)	1
	Shanghai (HSNU)	1
	Shanghai (SHM)	1
	Shenyang (IFP)	2
*	Shillong (ASSAM)	1
*	Singapore (SING)	2
	Sofia (SOM)	3
	Stevens Point (UWSP)	1
	Stockholm (S)	6
	Stockholm (SBT)	1
	Storrs (CONN)	1
	Stuttgart (STU)	2
	Suva (SUVA)	1
	Sverdlovsk (SVER)	1
*	Sydney (NSW)	6
*#	Sydney (SYD)	1
	Taipei (HAST)	1
	Taipei (NTUF)	1
	Taipei (TAI)	1
	Taipei (TAIF)	1
	Tallahassee (FSU)	1
	Tampa (USF)	1
	Tartu (TAA)	2
*	Tartu (TU)	2
	Tashkent (TASH)	2
	Tbilisi (TBI)	2
	Tegucigalpa (EAP)	1
*	Tehran (IRAN)	2
	Tehran (TARI)	1
	Tel Aviv (TELA)	1
*	Tempe (ASU)	1
	Thessaloniki (TAU)	1
	Tiruchirapalli (RHT)	1
	Tokyo (MAK)	2
	Tokyo (TI)	4
	Tokyo (TNS)	5
	Tomsk (TK)	1
	Torino (TO)	1
*	Toronto (TRT)	1
	Toronto (TRTC)	1
	Torufi (TRN)	1
	Tottori (TMI)	1
	Toulouse (TL)	1
*#	Townsville (JCT)	1
	Trieste (TSB)	2
	Tripoli (ULT)	1
	Trondheim (TRH)	2
	Trujillo (HUT)	1
	Tsukuba (TKB)	1
	Tübingen (TUB)	1
	Tucson (ARIZ)	1
*	Turku (TUR)	2
	Ulan Bator (UBA)	1
	Umeå (UME)	2
	University Park (PAC)	1
	Uppsala (UPS)	4
*	Urbana (ILL)	1
	Urumqi (XJA)	1
	Urumqi (XJBI)	1
	Urumqi (XJU)	1
*	Utrecht (U)	1
*	Vácrátót (VBI)	1
#	València (VAL)	2
	Vancouver (UBC)	2
	Victoria (DAVFP)	1
	Victoria (V)	1
	Vilnius (BILAS)	1
	Vladivostok (VLA)	1
*	Wageningen (WAG)	4
	Warszawa (WA)	3
*	Washington (NA)	1
*	Washington (US)	7
	Waterloo (WAT)	1
*	Wellington (WELT)	1
*	Wellington (WELTU)	1
*	Wien (W)	3
*	Wien (WU)	5
*	Windhoek (WIND)	1
*	Wisley (WSY)	1
	Wolfville (ACAD)	1
	Wrocław (WRSL)	1
	Wuchang (WH)	1
	Wuhan (CCNU)	1
	Wuhan (HBI)	1
	Wuhan (HIB)	1
*	Xalapa (XAL)	2
	Xining (HNWP)	2
	Xishuangbanna (HITBC)	1
	Yangling (WUK)	2
	Yaounde (YA)	1
*	Yerevan (ERE)	3
	Zagreb (ZA)	1
	Zapopan (IBUG)	2
	Zomba (MAL)	1
	Zürich (Z + ZT)	3

## Appendix B

List of registered members of the Nomenclature Section

This list is also an index to speakers as recorded in the preceding report, with page references to all relevant entries.

Acevedo, P., U.S.A. ... 168

Adams, P., Australia

Agosti, D., Switzerland

Aitchison, W., Australia

Alfaro, D., Australia

Alvarado Reyes, E., Sweden

Anderson, W., U.S.A. ... 205

Applequist, W., U.S.A. ... 34, 78, 133, 135, 175, 178, 189, 191, 207, 210, 228, 234, 238, 260, 276

Barfuss, M. H. J., Austria

Barker, C. [Christine], U.K. ... 253, 254

Barker, R., Australia

Barker, W. [Bill], Australia ... 35, 56, 58, 65, 128, 129, 134, 138, 140

Barkworth, M., U.S.A.

Barrett, R., Australia

Barrie, F. R., U.S.A. ... 12, 13, 26, 31, 37, 40, 41, 45-47, 51, 56, 61, 63, 64, 67, 70, 72, 76, 77, 109, 112, 114, 116, 123, 137, 143, 146, 148, 150, 166, 183, 188, 200, 211, 212, 215, 218, 229-233, 235, 236, 238, 244-246, 254, 256, 263, 270, 273, 276, 277

Batty, E., Australia

Bayly, M., Australia ... 117

Bell, K., Australia

Bellu, F., Italy

Belyaeva, I., U.K.

Bester, S. P., South Africa

Boyne, R., Australia ... 45, 201

Breitwieser, I., New Zealand ... 209

Briggs, B., Australia ... 139, 150, 275

Brown, A., Australia

Brown, G., Australia

Brownsey, P., New Zealand

Buck, W. R., U.S.A. ... 25, 43, 48, 49, 56, 58, 63, 84, 110, 115, 120, 139, 147, 164, 173, 183, 228, 233, 234, 258, 263, 266, 271, 272

Butcher, R., Australia

Cafferty, S., U.K. ... 120, 136, 166, 178, 227

Cameron, D., Australia ... 148, 149, 210, 270

Cantrill, D., Australia ... 106

Casanova, M., Australia

Challis, K., U.K. ... 98, 118, 124, 143, 155, 166, 177, 194, 245, 249, 251

Chang, Ching-Sung, China

Chang, Ho-Ming, China

Chao, Yi-Shan, Taiwan

Coetzee, J. C., South Africa

Collins, B., Australia ... 8

Conway, S., Australia

Cosgrove, M., Australia

Cowley, K., Australia

Crane, J. L., U.S.A. ... 107

Croft, J., Australia

David, J., U.K. ... 29, 34

Davidse, G., U.S.A. ... 115, 184, 205, 250, 253

De Lange, P., New Zealand

Demoulin, V., Belgium ... 20, 22, 25, 29, 30, 37, 41, 51, 58, 64, 67, 75, 79, 88, 91, 95, 97, 124-126, 141, 142, 151, 152, 161, 163, 164, 173, 185, 187, 190, 197, 202, 204, 217, 222, 223, 227, 229, 230, 239, 241, 242, 244, 250, 251, 253-255, 258-260, 262, 264, 266

Deshmukh, Y., India

Dhabe, A., India ... 137

Dixon, D., Australia ... 125

Dong, H., China

Dönmez, A. A., Turkey

Dorr, L., U.S.A. ... 63, 103, 108, 110, 120, 135, 136, 161, 167, 179, 220, 221, 230, 237, 248, 274

Downing, T., Australia

Dressler, S., Germany ... 128, 168, 195

Drinnan, A., Australia

Dunk, C., Australia

Duretto, M., Australia

Feuillet, C., U.S.A.

Flann, C., Netherlands ... 107, 124, 211

Ford-Werntz, D., U.S.A. ... 34, 212

Fortunato, R., Argentina ... 176, 212

Funk, V. A., U.S.A. ... 16, 27, 54, 83, 87, 95, 96, 111, 145, 176, 177, 231, 237, 240, 243, 255, 256, 270

Gallagher, C., Australia

Gams, W., Netherlands ... 20, 24, 35, 40, 104, 164, 174, 220, 222, 226, 227

Gandhi, K., U.S.A. ... 16, 20, 31, 40, 53, 56, 58, 59, 61, 72, 81, 106, 122, 131, 135, 136, 146, 148, 151, 154, 155, 160, 168, 175, 184, 189, 191, 192, 195, 196, 199, 204, 205, 211, 214, 215, 226, 245, 252, 254

Geltman, D., Russia ... 211

Gereau, R., U.S.A. ... 14, 21, 34, 37, 49, 50, 51, 59, 65, 75, 87, 92, 95, 98, 125, 135, 143, 146, 149, 176, 177, 184, 188, 190, 193, 195, 201, 215, 218, 236, 241, 244, 249, 251, 252, 260, 268, 274

Gillespie, L., Canada

Giulietti Harley, A. M., Brazil

Glen, H., South Africa ... 18, 21, 81, 103, 111, 112, 120, 151, 194, 216, 255

Glen, M., South Africa

Gott, B., Australia

Govaerts, R., U.K. ... 37, 143, 147

Greuter, W., Switzerland ... 13, 18-20, 24, 25, 31-34, 36, 38, 43, 46, 52, 60, 67, 73, 79, 89-91, 109, 115, 118-120, 124, 134, 139-146, 155, 157, 158, 168, 176-178, 186, 191, 194, 195, 197, 198, 210, 212, 223, 229, 231, 233, 234, 236-238, 242-247, 250, 253, 258, 261, 262

Harley, R. M., U.K. ... 21, 29, 30, 72, 98, 136, 138, 151

Hawksworth, D. L., Spain ... 19, 21, 22, 26, 45, 66, 111, 112, 155, 165, 169, 172, 178, 205, 218, 224, 229, 230, 232, 234, 237-239, 260, 272, 274

Hawksworth, P., U.K.

Head, M., Canada ... 23, 118

Herendeen, P. S., U.S.A. ... 21, 22, 24, 26, 27, 32, 33, 44, 46, 62, 80, 102, 108, 109, 113, 120, 125, 131, 151, 161, 164, 180, 200, 253, 257, 276

Hirai, R. Y., Brazil

Hoch, P., U.S.A.

Hollowell, V., U.S.A. ... 116

Holmes-Anderson, H., Australia

Howlett, B., Australia

Hsu, Tsai-Wen, Taiwan

Huang, Shing-Fan, Taiwan

Huang, Tseng-Chieng, Taiwan

Ingle, P., India

James, L., Australia

Janarthanam, M., India ... 102, 114, 115, 121, 136, 150, 169, 176

Janssen, T., Belgium ... 57

Jiao, G., China

Jin, H., China

Johnston, P., New Zealand ... 226

Kellermann, J., Australia ... 47, 55, 86, 105, 111, 119, 136, 137, 179, 209, 216, 246, 251

Kilian, N., Germany

Kirk, P., U.K. ... 21, 29, 40, 46, 47, 81, 103, 116, 118, 119, 130, 138, 151, 160, 161, 169, 173, 178, 223, 226, 233, 235, 254

Klazenga, N., Australia ... 275

Klopper, R., South Africa

Knapp, S., U.K. ... 7-19, 21, 23-28, 30, 31, 34-36, 38-40, 42, 43, 46-48, 50, 54-57, 59, 60, 62-68, 71-76, 78, 80-87, 90, 92, 94, 97, 99, 100, 103, 107-111, 113-115, 117-122, 124, 125, 127-132, 134, 136-138, 140, 143, 145-147, 149, 151-155, 158-161, 165, 166, 169, 171, 172, 176-179, 183, 184, 186-190, 192, 194-198, 200, 202, 204-206, 208-210, 212-216, 218, 220, 221, 223, 224, 226-234, 236-238, 240-263, 266-275

Knox, E., U.S.A. ... 16, 18

Kodela, P., Australia

Köster, N., Germany

Kvacek, J., Czech Republic

Ladiges, P., Australia ... 18, 25, 102, 118, 126, 209

Lendemer, J., U.S.A. ... 57, 101, 109, 110, 119, 122, 125, 127, 130, 136, 137, 139, 170, 175, 177, 179, 223, 224, 230, 234, 239, 271

Lepschi, B. J., Australia

Levin, G., U.S.A. ... 35, 85, 167, 201, 213

Lewis, E., Australia

Lewis, G., U.K. ... 18, 96, 201, 204, 207, 258

Liu, H., Taiwan

Löhne, C., Germany

Loizeau, P.-A., Switzerland

Luckow, M., U.S.A. ... 12, 14, 16, 208, 225

Mabberley, D., U.K. ... 203, 209, 210

Magill, R., U.S.A. ... 111, 142, 168, 247

Malécot, V., France ... 10, 22, 27, 30, 37, 55-57, 90, 119, 131, 133, 190, 207

Marhold, K., Slovakia ... 43, 56, 57, 107, 110, 114, 137, 148, 174, 197, 218, 234, 235, 241, 273

Maslin, B. R., Australia ... 201

May, T., Australia ... 24, 43, 45, 107, 111, 119, 126, 130, 137, 165, 169, 170, 174, 225, 227, 228, 234, 237, 239, 242, 255

McNeill, J., U.K. ... 8-19, 21-31, 33-44, 46-53, 55-82, 84-94, 96-99, 108, 109, 111-113, 115-118, 120-126, 128-148, 150-162, 164-172, 174, 177-195, 197-200, 204, 205, 207-210, 212-224, 228, 230, 232-264, 266, 268-277

Meagher, D., Australia

Miles, W., Canada

Miller, J., U.S.A. ... 81, 117, 139

Milne, P., Australia

Monro, A. M., Australia

Murphy, D., Australia

Muscat, K., Australia

Myles, B., New Zealand

Nagamasu, H., Japan

Nelson, G., Australia ... 117, 165, 208

Nic Lughadha, E., U.K. ... 43, 83, 113, 116, 129, 130, 131, 139, 178, 183, 189, 195, 205, 210, 213, 217, 266

Nicholas, A., South Africa ... 205, 208, 212

Norvell, L. L., U.S.A. ... 25, 26, 30, 35, 41, 76, 77, 104, 123, 165, 172, 176-180, 231, 233, 265-269, 275

Ohlsen, D., Australia

Olde, P., Australia

O’leary, M., Australia

Orchard, T., Australia ... 114, 204, 211

Passalacqua, N. G., Italy ... 18

Paton, A., U.K. ... 106, 112, 127, 138, 169, 177, 180, 217

Penev, L., Bulgaria ... 106, 120, 123-125, 130, 133, 135, 175, 180

Pennycook, S., New Zealand ... 77

Perrie, L., New Zealand

Perry, G., Australia ... 39, 50, 51, 146, 189-191, 262, 264

Peterson, P., U.S.A.

Potgieter, C., South Africa ... 139, 206, 211

Prado, J., Brazil ... 49, 125, 128, 183

Price, M. J., Switzerland ... 131, 132, 228, 272

Prud’homme van Reine, W. F., Netherlands ... 19, 22-24, 28-30, 32, 38, 41, 42, 44-46, 48, 50, 64, 79, 118, 120, 121, 147-149, 217, 218, 225, 226, 231, 249, 259, 273, 275

Puşcas, M., Romania

Rainer, H., Austria

Rangan, H., Australia

Redhead, S., Canada ... 28, 30, 32, 46, 47, 50, 56, 57, 64, 74, 75, 77-80, 84-86, 88-90, 111, 113, 136, 148, 150, 151, 165, 170, 171, 176, 179, 182, 186, 187, 195, 204, 206, 217, 220-224, 228, 230, 232, 235, 236, 239, 240, 264-266, 269, 272, 275

Reier, Ü., Estonia

Reveal, J. L., U.S.A. ... 26, 39, 45, 53, 61, 62, 68, 71, 72, 74-76, 78, 89, 90, 92-94, 105, 121, 129, 131, 144, 154, 161, 163, 180-182, 186-188, 192, 194-196, 209, 240, 272

Rico Arce, M. L., U.K. ... 16, 150, 168, 177, 203, 212, 213

Saarela, J. M., Canada ... 137, 138, 169

Schrire, B., U.K. ... 201, 206

Sebsebe Demissew, Ethiopia ... 14, 166, 202, 208, 256

Séneca, A., Portugal

Sennikov, A., Finland ... 34, 35, 38, 40, 42, 52, 57, 66, 77, 81, 95, 105, 122, 124, 127, 135, 140, 144, 146, 147, 160, 161, 167, 193, 234

Shepherd, K., Australia

Short, P., Australia

Showalter, A., U.S.A.

Simon, B., Australia

Smith, G. F., South Africa ... 166, 204, 211

Söderström, L., Norway

Soreng, R., U.S.A. ... 37, 108, 109, 112, 113, 120, 137, 184

Stajsic, V., Australia

Stevens, P., U.S.A. ... 110, 127, 140, 170, 183, 196, 218, 250

Stuessy, T., Austria ... 79, 81, 169, 174

Sundue, M., U.S.A.

Syme, A., Australia

Talent, N., Canada ... 57, 87, 96

Thiele, K., Australia ... 12, 13, 15, 47, 85, 103, 112, 117, 169, 208, 255, 259

Thornhill, A., Australia

Turland, N. J., U.S.A. ... 33-36, 39, 42, 53, 69, 71, 73-77, 98, 115, 116, 120, 128-132, 134, 136, 138, 140, 144, 147, 156, 158-160, 162, 181, 182, 188, 194, 201, 203, 205-207, 215, 217, 243, 244, 258, 276

Udovicic, F., Australia

Ulloa Ulloa, C., U.S.A. ... 35, 84, 174, 249, 250, 274

Van Rijckevorsel, P., Netherlands ... 12, 13, 15, 27, 34, 43, 46, 52, 67, 68, 72, 79, 92, 98, 128, 137, 142, 143, 150, 158, 192, 200, 201, 204, 205, 231, 232, 245, 246, 248, 249, 270

Veldkamp, J., Netherlands ... 39, 52, 125, 150, 152, 241, 246, 247

Wagner, W., U.S.A.

Walsh, N., Australia ... 169, 170, 235

Wang, Liangmin, China

Ward, J., New Zealand

Ward, T., U.S.A.

Wege, J., Australia

West, J., Australia ... 7, 203

Wheeler, A., Australia

Whitbread, G., Australia ... 116, 117, 140

Wiersema, J. H., U.S.A. ... 22, 40, 59, 65, 66, 68, 70-73, 76, 91, 119, 148, 156, 161, 176, 188, 190, 207, 208, 216, 223, 230, 234, 245, 247, 251, 253, 269, 270

Wilson, A. [Annette], Australia ... 21, 43, 79, 83, 133, 213

Wilson, K. L. [Karen], Australia ... 25, 99, 100, 108, 109, 113-117, 120, 121, 123, 130, 132, 142, 153, 197, 203, 213, 275, 278

Wilson, P. [Peter], Australia ... 117, 165

Woodland, D., U.S.A.

Wu, Ming-Jou, China

Yadav, S. R., India

Yang, Shiow-Yu, Taiwan

Yin, Xiaofeng, U.S.A.

Zhang, Li, China

Zhang, Xianchun, China

Zuo, Benrong, China

